# Racial differences in RAD51 expression are regulated by miRNA-214-5P and its inhibition synergizes with olaparib in triple-negative breast cancer

**DOI:** 10.1186/s13058-023-01615-6

**Published:** 2023-04-20

**Authors:** Chinnadurai Mani, Ganesh Acharya, Karunakar Saamarthy, Damieanus Ochola, Srinidhi Mereddy, Kevin Pruitt, Upender Manne, Komaraiah Palle

**Affiliations:** 1 Department of Cell Biology and Biochemistry, Department of Surgery, Texas Tech University Health Sciences Center, 3601 4th Street, Lubbock, TX 79430 USA; 2 Department of Cellular and Molecular Biology, University of Washington, 1400 NE Campus Parkway, Seattle, WA 98195 USA; 3 Department of Immunology and Infectious Diseases, Texas Tech University Health Sciences Center, 3601 4th Street, Lubbock, TX 79430 USA; 4 Department of Pathology, University of Alabama at Birmingham, Birmingham, AL 35294 USA; 5 Department of Surgery, Texas Tech University Health Sciences Center, Lubbock, TX 79430 USA

**Keywords:** miRNA, RAD51, Racial disparity, Olaparib, HR deficiency

## Abstract

**Background:**

Triple-negative breast cancer (TNBC) affects young women and is the most aggressive subtype of breast cancer (BC). TNBCs disproportionally affect women of African-American (AA) descent compared to other ethnicities. We have identified DNA repair gene RAD51 as a poor prognosis marker in TNBC and its posttranscriptional regulation through microRNAs (miRNAs). This study aims to delineate the mechanisms leading to RAD51 upregulation and develop novel therapeutic combinations to effectively treat TNBCs and reduce disparity in clinical outcomes.

**Methods:**

Analysis of TCGA data for BC cohorts using the UALCAN portal and PrognoScan identified the overexpression of RAD51 in TNBCs. miRNA sequencing identified significant downregulation of RAD51-targeting miRNAs miR-214-5P and miR-142-3P. RT-PCR assays were used to validate the levels of miRNAs and RAD51, and immunohistochemical and immunoblotting techniques were used similarly for RAD51 protein levels in TNBC tissues and cell lines. Luciferase assays were performed under the control of RAD51 3’-UTR to confirm that miR-214-5P regulates RAD51 expression. To examine the effect of miR-214-5P-mediated downregulation of RAD51 on homologous recombination (HR) in TNBC cells, Dr-GFP reporter assays were performed. To assess the levels of olaparib-induced DNA damage responses in miR-214-5P, transfected cells, immunoblots, and immunofluorescence assays were used. Furthermore, COMET assays were used to measure DNA lesions and colony assays were performed to assess the sensitivity of BRCA-proficient TNBC cells to olaparib.

**Results:**

In-silico analysis identified upregulation of RAD51 as a poor prognostic marker in TNBCs. miRNA-seq data showed significant downregulation of miR-214-5P and miR-142-3P in TNBC cell lines derived from AA women compared to Caucasian-American (CA) women. miR-214-5P mimics downregulated RAD51 expression and induces HR deficiency as measured by Dr-GFP assays in these cell lines. Based on these results, we designed a combination treatment of miR-214-5P and olaparib in HR-proficient AA TNBC cell lines using clonogenic survival assays. The combination of miR-214-5P and olaparib showed synergistic lethality compared to individual treatments in these cell lines.

**Conclusions:**

Our studies identified a novel epigenetic regulation of RAD51 in TNBCs by miR-214-5P suggesting a novel combination therapies involving miR-214-5P and olaparib to treat HR-proficient TNBCs and to reduce racial disparity in therapeutic outcomes.

**Supplementary Information:**

The online version contains supplementary material available at 10.1186/s13058-023-01615-6.

## Background

Triple-negative breast cancer (TNBC) constitutes 15–20% of all breast cancers (BC), occurs at a young age (< 40 years), is more aggressive, and contributes to a relatively higher proportion of BC-related deaths in women. TNBCs are negative for both the estrogen receptor (ER) and progesterone receptor (PR) and do not overexpress epidermal growth factor receptor 2 (EGFR-2 or HER-2), which classify and define therapeutic modalities. Consequently, no targeted therapies are currently available for these patients [1–4]. Thus, most of these patients undergo cytotoxic chemotherapy; however, the responses are short-lived and the majority of patients develop resistance and undergo relapse [5–7]. Moreover, TNBC disproportionally affects women of African-American (AA) descent compared to other ethnicities and show the worst clinical outcomes in this population [8, 9]. The health statistics data indicate that the occurrence of TNBCs is over two times higher for AA women compared to women of European or Caucasian ancestry (EA) [10, 11]. Although socioeconomic [12] and comorbidity factors contribute to differences in clinical outcomes, the higher prevalence of TNBCs in young AA women, combined with higher death rates, suggests different molecular factors influence this disease [13].

RAD51 is a key downstream factor to BRCA1/2-mediated DNA repair [14] and facilitates the homology search to repair DNA double-strand breaks (DSBs) [15] in coordination with ATM/ATR-mediated DNA damage checkpoint responses [16]. Moreover, *RAD51* upregulation is evident in many cancers [17], including BCs [18], and is implicated in the development of tumor resistance to chemotherapeutics [19, 20]. Our previous studies showed that *RAD51* is upregulated in TNBCs, and its expression is significantly higher in AA TNBCs compared to EA TNBC patients and correlates with a poor prognosis relative to EA TNBC patients [21]. However, the mechanisms behind the upregulation of RAD51 in these patients have not been identified, particularly those contributing to the observed racial disparity.

The Food and Drug Administration (FDA) has approved PARP inhibitors (PARPi) olaparib [22] and talazoparib [23] for the treatment of BRCA-deficient BC [24, 25]. Although PARP inhibitors have shown promise, their use and effectiveness are limited to only 15–20% of TNBC patients, particularly those with *BRCA* mutations or homologous recombination (HR) DNA repair deficiency (HRD). In these patients, PARPi effectiveness is attributed to synthetic lethality [26], a process wherein the simultaneous perturbation of two compensatory genes or pathways causes cell death [27]. Therefore, the majority (~ 80%) of the TNBC patients do not benefit from PARPi-targeted therapies. The increased expression of RAD51 observed in TNBCs, particularly in AA patients, may facilitate increased DNA repair and resistance, leading to therapeutic relapse and poor outcomes [9, 28]. Moreover, currently available data on biological and genetic differences do not explain the high mortality rate for AA women nor decode the genetic and epigenetic mechanisms that cause these discrepancies.

MicroRNAs (miRNAs) are short, non-coding RNAs with a length of 18–23 nucleotides that act as epigenetic regulators of gene expression [29]. About 2000 miRNAs have been discovered in humans, and they regulate one-third of the human genome [30]. miRNAs are associated with numerous human diseases and are being investigated as clinical diagnostics and therapeutic targets, particularly involving cancer progression and their influence on therapeutic responses [30]. These observations prompted us to perform miRNA-seq analysis to find differentially regulated miRNAs in DNA repair-proficient TNBC cell lines representing both AA and EA ethnicities, particularly those targeting the *RAD51* gene. Our present study identified the loss of miRNA-214-5P (miR-214-5P) in TNBCs, particularly in AA TNBC cell lines and tumors. Therefore, the goals of our current studies are to delineate miRNA-mediated epigenetic mechanisms leading to RAD51 upregulation and develop novel therapeutic combinations based on these biomarkers to effectively treat BRCA-proficient TNBCs and reduce disparity in outcomes.

In this study, our data show that miR-214-5P regulates RAD51 and suggests that this epigenetic regulation of RAD51 contributes to disparities in TNBC’s therapeutic outcomes. Additionally, our data demonstrate that miR-214-5P-mediated downregulation of RAD51 causes HRD, and synergistic lethality with the PARPi, olaparib, in HR-proficient TNBC cells.

## Materials and methods

### TNBC patient cohort and sample collection

To identify the *RAD51* and miRNAs (miR-214-5P and miR-142-3P) differential expression in TNBCs of AA and EA women, we performed RT-PCR on a TNBC cohort. Formalin-fixed, paraffin-embedded (FFPE) TNBC samples were obtained from the Division of Anatomic Pathology in the University of Alabama at Birmingham (UAB). The UAB Institutional Review Board approved the collection and use of all samples in this study (IRB number: 060911009). Before RNA extraction, pathologists macro-dissected all tumors and corresponding normal regions.

### Cell lines, culture method, and reagents

The human TNBC cell lines MDAMB231, MDAMB453, HCC1806, and MDAMB468 were purchased from ATCC, Manassas, VA. These cells were cultured in Dulbecco’s modified Eagle medium (Corning, Manassas, VA) supplemented with 10% fetal bovine serum (Omega Scientific Inc., Tarzana, CA) and 1% penicillin–streptomycin (50  U/mL, 50 μg/mL) (Invitrogen, Eugene, OR). Olaparib (Selleckchem, Houston, TX) was dissolved in DMSO and used at 25 µM for 24 h in western blot, COMET assay and immunofluorescence experiments. For the colony assay, 0–4 µM of Olaparib was used. Primary antibodies for the following were used for western blotting: pH2AX (Cat No: 05636, Millipore), RAD51 (Cat No: 8875, Cell Signaling) and GAPDH (Cat No: 32233, Santa Cruz).

### miRNA sequencing

Total RNA was isolated from AA (MDAMB468 and HCC1806) and EA (MDAMB231 and MDAMB453) TNBC cell lines and outsourced to Admera Health LLC for analysis. miRNA-seq data were trimmed with Cutadapt default parameters and then aligned with miRDeep2 and Bowtie. Quantification of miRNA expression was performed with miRDeep2. DE-Seq2 determined differentially expressed miRNAs. PCA plots and heatmaps were generated using R. miRNA sequencing was performed once, and RT-PCR further confirmed the results in three independent experiments.

### miRNA transfection

Control miRNA (CN-001000-01-20), miR-214-5P (C-301153-01-0020), and miR-142-3P (C-300610-03-0005) were procured from Horizon Discovery Biosciences Limited. miRNAs were transfected using Lipofectamine RNAiMAX (Life Technologies, Eugene, OR) based on the protocol supplied by the manufacturer.

### HR Dr-GFP assay

As previously described [21], the Dr-GFP reporter assay measures HR activity. pDR-GFP and pCBASceI were gifts from Maria Jasin (Addgene plasmids # 26477 and # 26475, respectively), Addgene (Watertown, MA). In brief, MDAMB468 cells were stably transfected with pDr-GFP and selected for puromycin resistance (2 μg/mL). Upon 60% confluence, the stably transfected cells were transfected with the plasmid I-Sce1 and a miR-control or miR-214-5P. Restriction enzyme I-Sce1 cuts the reporter plasmid and initiates GFP expression when the damage is repaired by HR. After transfection of miRNAs for 24 h, GFP-positive cells were measured by flow cytometry using a BD Accuri (BD Biosciences) flow cytometer. A total of three independent experiments were performed.

### Protein expression by western blot

The cells were placed on ice and washed twice with ice-cold PBS, and cell lysates were collected using cytoskeletal (CSK) buffer (10 mM PIPES at pH 6.8, 100 mM NaCl, 300 mM sucrose, 3 mM MgCl2, 1 mM EGTA, 0.1 mM ATP, 0.1% Triton X-100 freshly supplemented with 1 mM dithiothreitol, 1 × protease, and phosphatase inhibitors with EDTA). Protein content was measured with Bradford reagent, and the proteins were equilibrated using CSK buffer with 6 × Laemmli buffer and heated at 100 °C for 15 min. The proteins were resolved on gradient polyacrylamide gels and then transferred onto nitrocellulose membranes using the BioRad Trans-Blot Turbo system. The membranes were blocked with a 2.5% blocking grade blocker (BioRad, USA) in 1 × Tris-buffered saline in 0.1% Tween 20 (TBST) and incubated with the primary antibody overnight on a rocking platform at 4 °C. Membranes were then washed three times with 1 × TBST before being incubated for an hour at room temperature with the secondary antibody. The membranes were then washed three times with 1 × TBST, exposed to Western lightning plus ECL (PerklinElmer, USA), and developed in a dark room with Konica Minolta equipment.

### Cell cycle analysis

After transfection with miR-control or miR-214-5P, cells were trypsinized, collected in tubes, spun down, and washed with ice-cold PBS. Cells were then suspended in ice-cold ethanol and incubated overnight at − 20 °C. After incubation, cells were washed with PBS, stained with propidium iodide (PI) (Invitrogen, Eugene, OR) in the presence of RNAse A (Thermo Scientific), and processed for cell cycle profiles by flow cytometry using a BD Accuri (BD Biosciences) flow cytometer. ModFit LT 5.0 software was used to analyze the cell cycle profiles. A total of three independent experiments were performed.

### Immunohistochemistry

Tissue sections were incubated with RAD51 (Cat No: 8349, Santa Cruz), followed by a specific biotinylated secondary antibody (1:250 dilution) using the kit from Vectastain (PK-6101), and then with conjugated horse radish peroxidase-streptavidin and 3′3-diaminobenzidine HCl chromogen, and tissues were counterstained with hematoxylin. Stained sections were imaged with an AxioCam camera, and RAD51 intensities were analyzed with a Zeiss Axioscope microscope as described previously [31]. A total of three independent experiments were performed.

### RNA isolation and real-time PCR

Using a ZYMO Research (Irvine, CA) Quick-RNA MiniPrep RNA isolation kit, total RNA was extracted from cells transfected with miR-control or miRNA-214-5P. RNA (1 µg) was reverse-transcribed using a high-capacity cDNA reverse transcription kit (Invitrogen, Eugene, OR), as per the manufacturer’s protocol and as described previously [32]. The primers for amplified genes (RAD51 and GAPDH) were purchased from Bio-Rad. Amplification of PCR products was quantified using SYBR green dye (ABI), and fluorescence was monitored on a QuantStudio 12 K Flex detection system. Melting curve analysis was accomplished for each amplicon. Quantitation was done using the 2^−ΔΔCt^ technique with GAPDH as an endogenous control. A total of two independent experiments in triplicate were performed in patients’ samples. A total of three independent experiments were performed in cell lines.

### Immunofluorescence

First, the cells were seeded into FluoroDishes (World Precision Instruments) and incubated overnight for adherence. After treatment, cells were fixed with 4% formaldehyde for 10 min at room temperature. The cells were permeabilized with PBS containing 0.2% Triton X-100 for 3 min. The cells were washed and blocked with 10% goat serum in PBS for 40 min. After three PBS washes, cells were incubated overnight at 4 °C with primary antibodies (RAD51: Cat No: 8349, Santa Cruz or pH2AX: Cat No: 05636, Millipore) in PBS, then incubated with a fluorescent secondary antibody (Molecular Probes, Eugene, OR) for 2 h at room temperature. The cells were mounted with Vectashield containing 4′,6-diamidino-2-phenylindole and analyzed for foci formation using a Nikon Eclipse TE confocal microscope. A total of three independent experiments were performed.

### Comet chip assay

As previously reported [33] and following the manufacturer’s instructions, comet assays were performed under alkaline conditions using Comet Chip Assay Kits (Trevigen, Gaithersburg, MD). The cells transfected with miR-control or miR-214-5P were treated for 24 h with DMSO or 25 μM olaparib, harvested, suspended at a concentration of 1 X 10^5^ cells/ml in PBS combined with 1% low melting point agarose at a ratio of 1:10 (v/v). The Comet chip was immersed in a lysis solution for 30 min and electrophoresed in a horizontal electrophoresis apparatus containing an alkaline solution. The chip was then neutralized with 0.4 M and 20 mM Tris–HCl, pH 7.4, and stained with SYBR gold overnight. The chip was again de-stained with 20 mM Tris–HCl, pH 7.4, to visualize cellular DNA with a Zeiss Axio fluorescence microscope. Fluorescence images were analyzed using the ImageJ comet plugin to demarcate each comet's “head” and “tail” regions. The comet tail areas were measured, and calculations were averaged from three independent experiments.

### Clonogenic survival assay

For high-density and low-density colony formation assays, 5 × 10^3^ cells per well and 5 × 10^2^ cells per well, respectively, were seeded into 6-well culture plates and incubated overnight for adherence. Cells transfected with miR-control or miR-214-5P were then treated with DMSO or various concentrations of olaparib and cultured for colony formation over 7–10 days. After colony formation, the growth medium was removed, and cells were washed with ice-cold PBS three times and then fixed in ice-cold methanol for 5 min. Methanol was removed, and 1% w/v crystal violet (Invitrogen, Eugene, OR) was added for staining. After 10 min, the wells were washed under tap water, and the plates were allowed to dry at room temperature. Colonies were then imaged and counted using ImageJ software. A total of three independent experiments were performed.

### Luciferase promoter assay

Individual GoClone (Luciferase with a RAD51 3’-untranslated region (3’UTR) (Cat No: S807477, Active Motif) constructs were purchased from Switch Gear Genomics. Luciferase assays were performed for MDAMB468 and HCC1806 cells using a LightSwitch Assay System kit (Cat No: LS010, Switch Gear Genomics) according to the manufacturer’s protocol. In brief, TNBC cells were transfected with miR-control or miR-214-5P for 48 h and, for the last 24 h, co-transfected with the vector expressing luciferase mRNA with the RAD51 3’UTR. After transfection/treatment, luciferase activity in the cells was measured using LightSwitch Assay reagents with a Tecan microplate reader. A total of three independent experiments were performed.

### Online databases

The prognostic landscapes of *RAD51* in BCs were identified from the PrognoScan database. The expression profiles of RAD51 in BCs were identified from analysis of the TCGA database using the UALCAN portal.

### Statistical analysis

Student’s t-test was performed to estimate statistical significance using GraphPad Prism 8.0 software.

## Results

### RAD51 is overexpressed in TNBCs, particularly those of AA women, and its high expression correlates with a poor prognosis

**Fig. 1:**
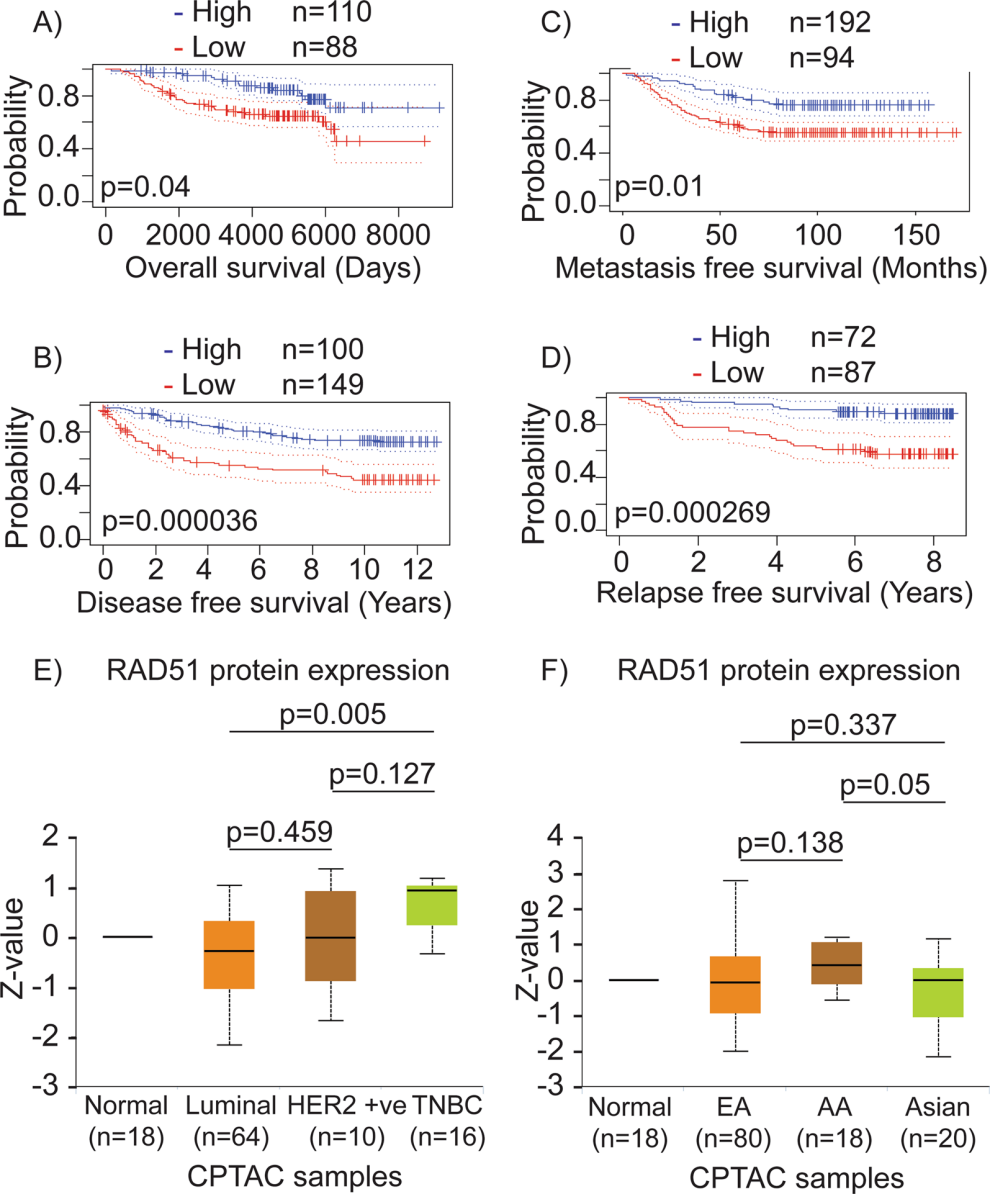
*RAD51* overexpression in TNBC is associated with poor prognosis. **A** Overall survival probability between breast cancer patients with high and low/medium *RAD51* expression. **B** Disease-free survival probability between breast cancer patients with high and low/medium *RAD51* expression. **C** Metastasis-free survival probability between breast cancer patients with high and low/medium *RAD51* expression. **D** Relapse-free survival probability between breast cancer patients with high and low/medium *RAD51* expression. **E** Expression of RAD51 in different subtypes of breast cancer. **F** RAD51 expression in breast cancer patients with diverse racial backgrounds

**Fig. 2:**
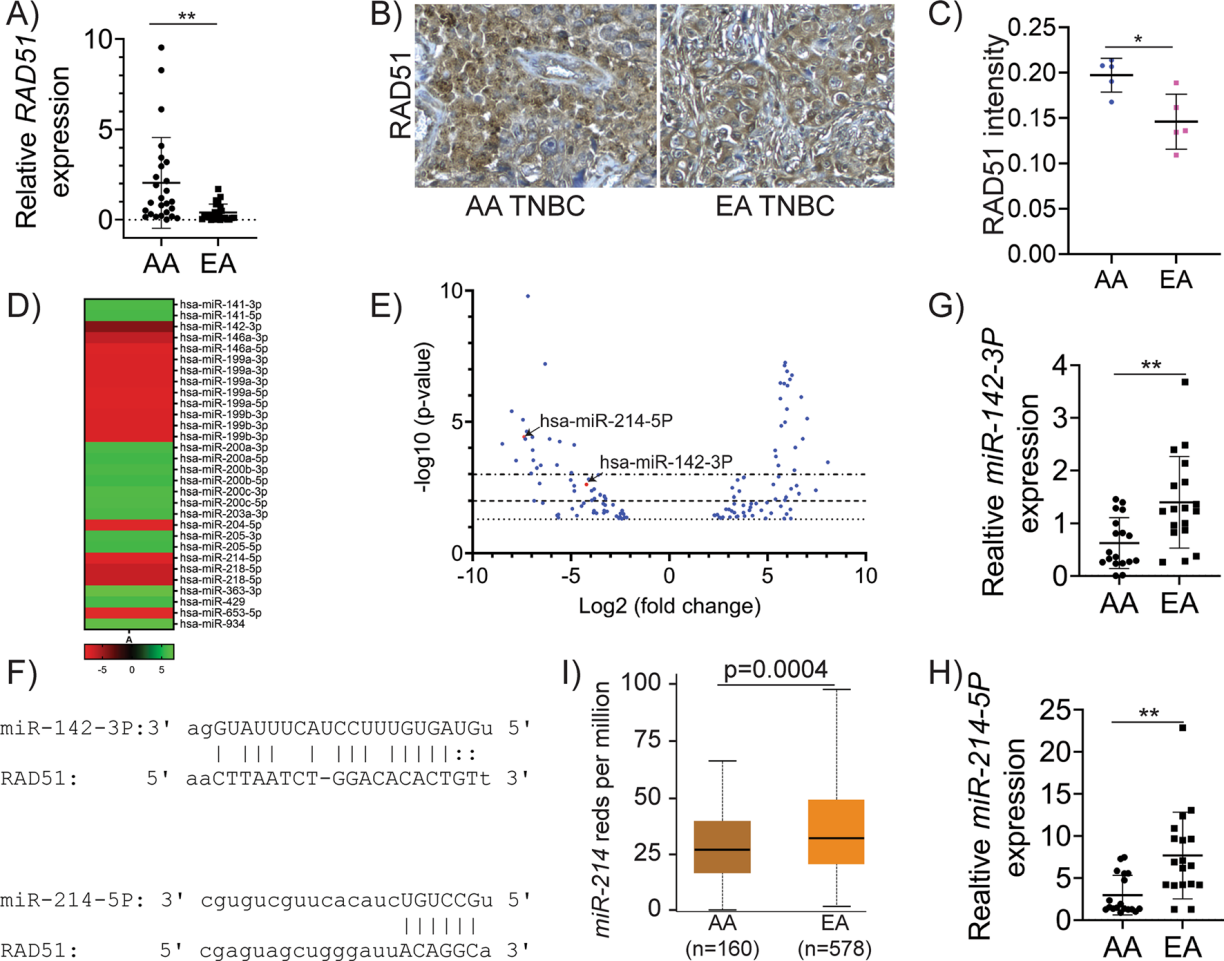
RAD51, miR-214-5P and miR-142-3P are differentially regulated between EA and AA TNBC samples. **A** Expression of *RAD51* in AA (*n* = 26) and EA (*n* = 26) TNBC patients was analyzed by RT-PCR in two independent experiments in triplicate. **B**, **C** Expression of RAD51 in AA (*n* = 5) and EA (*n* = 5) TNBC patients analyzed by IHC in three independent experiments. **D** List of the top 30 miRNAs that were differentially regulated in racially different TNBC cell lines [AA (MDAMB468 and HCC1806) and EA (MDAMB231 and MDAMB453)]. **E** Volcano plot analysis of the miRNA-seq data based on the fold change and p-values in racially different TNBC cell lines [AA (MDAMB468 and HCC1806) and EA (MDAMB231 and MDAMB453)]. **F** Seed sequence in *RAD51* to bind with miR-214-5P and miR-142-3P. **G** Expression of *miR-142-3P* in AA (*n* = 16) and EA (*n* = 16) TNBC patients analyzed by RT-PCR in two independent experiments with triplicates. **H** Expression of *miR-214-5P* in AA (*n* = 16) and EA (*n* = 16) TNBC patients analyzed by RT-PCR in two independent experiments in triplicate. **I** *miR-214* expression in breast cancer patients with different racial backgrounds. (*p < 0.05) and (**p < 0.01)

We recently showed that *RAD51* is upregulated in BCs, particularly in TNBCs [21]. To determine whether *RAD51* could be a prognostic marker for BCs, we utilized RAD51 expression data from PrognoScan for a meta-analysis. PrognoScan uses the minimum p-value approach for the grouping of patients. Patients are separated into two groups (high and low expression) at each cutpoint, and the risk differences between the two groups are assessed using the log-rank test. The best cutpoint with the most pronounced p-value is then chosen to determine high and low expression [34]. Our analysis of RAD51 expression data for BC showed that patients with high *RAD51* levels (*n* = 110) had low overall survival compared to the patients with low *RAD51* (*n* = 88) (Fig. 1A). Similarly, patients with high *RAD51* expression (*n* = 100) also had lower disease-free survival compared to the patients with low *RAD51* expression (*n* = 149) (Fig. 1B). Consistently, BC patients with high *RAD51* levels (*n* = 192) showed lower metastasis-free survival and relapse-free survival (*n* = 72) compared to the patients with low *RAD51* (*n* = 94 and *n* = 87), respectively (Fig. 1C, D). We expanded our analysis to explore the expression of RAD51 protein levels in BC using the UALCAN portal. Interestingly, RAD51 levels were upregulated in the TNBC subtype (*n* = 16), compared to normal breast tissues (*n* = 18) and other subtypes (luminal: *n* = 64; HER2-positive: *n* = 10) of BC (Fig. 1E). Additionally, these data indicate relatively high RAD51 protein levels in TNBCs of AA (*n* = 18) patients compared to those of EAs (*n* = 80) (Fig. 1F). To validate CPTAC data, we analyzed RAD51 expression levels in TNBCs of our bio-specimen repository using RT-PCR and immunohistochemistry (IHC). Consistently, our results show that RAD51 transcript levels were significantly higher in AA TNBC (*n* = 26) samples compared to EA TNBC (*n* = 26) (Fig. 2A). Age at diagnosis and stage of TNBC for the samples used in Fig. 2A are listed in Table 1, showing no distinctive difference between the AA (*n* = 26) and EA (*n* = 26) TNBCs. Similarly, RAD51 protein levels were also significantly higher in AA TNBC (*n* = 5) specimens compared to EA TNBC (*n* = 5) as evidenced by the IHC data (Fig. 2B, C). These data indicate an important role for RAD51 in TNBC progression and prognosis. Additionally, RAD51’s distinct expression in TNBCs of AA and EA ethnicity implicates a role in racial disparities in these patients. However, the mechanisms that influence RAD51 expression in TNBCs, and its distinctive regulation in AA TNBC patients are not known.

**Table 1:** Comparison of age at diagnosis and stage for TNBC samples used in Fig. 2A

Race	AA (*N* = 26)	CA (*N* = 26)	*P* value
Age at diagnosis (years ± SD))	52.69 ± 12.87	50.42 ± 14.15	0.260592
AJCC stage			
I	5 (19.2%)	2 (7.7%)	0.394143
II	13 (46.1%)	19 (73.1%)	0.96158
III	9 (34.6%)	4 (15.4%)	0.689564
IV	0	1 (3.8%)	NA

### RAD51 is epigenetically regulated in TNBCs, and loss of miR-214-5P and miR-142-3P leads to RAD51 upregulation

Recent studies from non-coding RNAs (ncRNAs), including miRNAs, indicate their differential regulation in different cancers and ethnicities, including TNBCs, and their attribution to disease progression and prognosis [35, 36]. To identify differentially regulated miRNAs, particularly those that target and epigenetically regulate RAD51, we performed miRNA-seq in two pairs of cell lines from each AA (MDAMB468 and HCC1806) and EA (MDAMB231 and MDAMB453)-derived TNBCs. A list of the top 30 miRNAs that were differentially regulated in AA vs EA TNBC cell lines are presented in Fig. 2D. The cell line-wise miRNA expression heatmap is shown in supplementary Fig. 1 (Additional file 1: Figure S1) and the list of miRNAs that are differentially expressed in AA TNBCs (MDAMB468 and HCC1806), compared to EA (MDAMB231 and MDAMB453) TNBCs, is presented in Table 2. To distinguish the upregulated and downregulated miRNAs based on the fold change and p-values, we presented a volcano plot analysis of the miRNA-seq data (Fig. 2E). Analysis of these data using the miRNA Target Base portal identified miR-214-5P and miR-142-3P as *RAD51*-targeting miRNAs based on the presence of their seed sequences (Fig. 2F). To validate these cell line data, we analyzed the expression of these miRNAs (miR-214-5P and miR-142-3P) in TNBC samples using RT-PCR. As shown in Fig. 2G, H, the expression of miR-142-3P and miR-214-5P was significantly downregulated in AA TNBCs (*n* = 16) compared to EA TNBCs (*n* = 16), respectively. Additionally, analysis of the TCGA database using the UALCAN portal further validated that miR-214 is significantly downregulated in AA TNBCs (*n* = 160) compared to EA TNBCs (*n* = 578) (Fig. 2I).

**Table 2:** Log-fold difference in the expression of miRNAs in AA TNBC (MDAMB468 and HCC1806) cells compared to EA TNBC (MDAMB231 and MDAMB453) cells

mature/precursor	baseMean	baseMean_Group1	baseMean_Group4	log2FoldChange	lfcSE	stat	*P* value	padj
hsa-miR-146a-5p/hsa-mir-146a	385,070.2	764,904.7	5235.596	− 7.19087	1.12452	− 6.39461	1.61E−10	1.42E−07
hsa-miR-200a-3p/hsa-mir-200a	196,515.4	6498.818	386,532	5.894247	1.085028	5.432343	5.56E−08	1.27 E−05
hsa-miR-218-5p/hsa-mir-218-1	11,387.58	22,493.3	281.8509	− 6.31582	1.166983	− 5.41209	6.23E−08	1.27E−05
hsa-miR-218-5p/hsa-mir-218-2	11,216.89	22,154.62	279.1662	− 6.30769	1.166167	− 5.40891	6.34E−08	1.27E−05
hsa-miR-429/hsa-mir-429	43,083.38	1448.776	84,717.99	5.869651	1.089666	5.386652	7.18E−08	1.27E−05
hsa-miR-200b-3p/hsa-mir-200b	61,842.89	1910.354	121,775.4	5.994171	1.132336	5.293631	1.20E−07	1.77E−05
hsa-miR-200c-3p/hsa-mir-200c	60,606.16	1580.814	119,631.5	6.2416	1.192958	5.232038	1.68E−07	2.12 E−05
hsa-miR-200c-5p/hsa-mir-200c	1077.441	29.91912	2124.962	6.141757	1.189709	5.162403	2.44E−07	2.70E−05
hsa-miR-141-5p/hsa-mir-141	2396.349	81.82585	4710.872	5.84472	1.147315	5.094259	3.50E−07	3.10E−05
hsa-miR-200a-5p/hsa-mir-200a	1359.018	52.76105	2665.275	5.657618	1.108421	5.104214	3.32E−07	3.10E−05
hsa-miR-141-3p/hsa-mir-141	79,769.54	2429.467	157,109.6	6.014875	1.188682	5.060119	4.19E−07	3.37E−05
hsa-miR-934/hsa-mir-934	5255.53	98.75334	10,412.31	6.717429	1.380521	4.865867	1.14E−06	8.40E−05
hsa-miR-200b-5p/hsa-mir-200b	1058.681	41.13295	2076.23	5.654164	1.169169	4.836056	1.32E−06	9.02E−05
hsa-miR-203a-3p/hsa-mir-203a	31,074.34	967.4037	61,181.28	5.98256	1.285927	4.652333	3.28E−06	0.000207
hsa-miR-653-5p/hsa-mir-653	67.20833	134.1523	0.264343	− 8.01717	1.738222	− 4.61228	3.98E−06	0.000235
hsa-miR-363-3p/hsa-mir-363	590.0655	8.915323	1171.216	7.01919	1.568426	4.475308	7.63E−06	0.000422
hsa-miR-204-5p/hsa-mir-204	153.4614	305.6012	1.321714	− 7.44442	1.671318	− 4.45422	8.42E−06	0.000438
hsa-miR-205-3p/hsa-mir-205	1350.024	44.59966	2655.449	5.89028	1.336186	4.40828	1.04E−05	0.000512
hsa-miR-205-5p/hsa-mir-205	397,639.7	15,012.93	780,266.5	5.699674	1.313337	4.339842	1.43E−05	0.000664
hsa-miR-199a-5p/hsa-mir-199a-1	11,260.56	22,373.91	147.2084	− 7.25272	1.715912	− 4.22674	2.37E−05	0.000999
hsa-miR-199a-5p/hsa-mir-199a-2	11,262.56	22,377.91	147.2084	− 7.25298	1.715926	− 4.22686	2.37E−05	0.000999
hsa-miR-199a-3p/hsa-mir-199a-1	129,993.6	257,919.7	2067.465	− 6.96318	1.692385	− 4.11442	3.88E−05	0.001227
hsa-miR-199a-3p/hsa-mir-199a-2	130,303.2	258,538.2	2068.258	− 6.96608	1.692158	− 4.11668	3.84E−05	0.001227
hsa-miR-199a-3p/hsa-mir-199b	129,993.9	257,920.3	2067.465	− 6.96318	1.692385	− 4.11442	3.88E−05	0.001227
hsa-miR-199b-3p/hsa-mir-199a-1	129,993.6	257,919.7	2067.465	− 6.96318	1.692385	− 4.11442	3.88E−05	0.001227
hsa-miR-199b-3p/hsa-mir-199a-2	130,303.2	258,538.2	2068.258	− 6.96608	1.692158	− 4.11668	3.84E−05	0.001227
hsa-miR-199b-3p/hsa-mir-199b	129,993.9	257,920.3	2067.465	− 6.96318	1.692385	− 4.11442	3.88E−05	0.001227
hsa-miR-214-5p/hsa-mir-214	292.7247	581.6662	3.783278	− 7.38049	1.790374	− 4.12232	3.75E−05	0.001227
hsa-miR-146a-3p/hsa-mir-146a	460.4773	908.5305	12.42411	− 6.09102	1.494113	− 4.07668	4.57E−05	0.001304
hsa-miR-375-3p/hsa-mir-375	1884.41	33.44618	3735.374	6.796739	1.666042	4.079573	4.51E−05	0.001304
hsa-miR-653-3p/hsa-mir-653	41.68788	83.11143	0.264343	− 7.32526	1.796643	− 4.07719	4.56E−05	0.001304
hsa-miR-143-3p/hsa-mir-143	2724.786	5336.925	112.6472	− 5.5749	1.385718	− 4.02312	5.74E−05	0.001588
hsa-miR-143-5p/hsa-mir-143	39.83994	79.67988	0	− 8.49774	2.138427	− 3.97383	7.07E−05	0.001897
hsa-miR-125b-1-3p/hsa-mir-125b-1	1683.312	3253.825	112.7993	− 4.83629	1.223446	− 3.95301	7.72E−05	0.002009
hsa-miR-187-3p/hsa-mir-187	743.1946	17.10855	1469.281	6.41598	1.645636	3.898784	9.67E−05	0.002445
hsa-miR-1269a/hsa-mir-1269a	275.6275	546.3731	4.881889	− 6.95012	1.808788	− 3.84242	0.000122	0.002994
hsa-miR-20b-5p/hsa-mir-20b	1989.002	92.82951	3885.175	5.386732	1.404353	3.835739	0.000125	0.002994
hsa-miR-203b-3p/hsa-mir-203b	146.2018	6.559193	285.8445	5.423171	1.464674	3.702646	0.000213	0.004969
hsa-miR-296-3p/hsa-mir-296	86.66459	171.9662	1.362953	− 7.79842	2.157872	− 3.61394	0.000302	0.006672
hsa-miR-296-5p/hsa-mir-296	75.40157	149.1759	1.627296	− 6.91959	1.911848	− 3.61932	0.000295	0.006672
hsa-miR-106a-3p/hsa-mir-106a	19.85376	0	39.70751	8.067004	2.257858	3.572858	0.000353	0.007622
hsa-miR-1910-5p/hsa-mir-1910	27.08289	1.12484	53.04095	5.548199	1.585176	3.500052	0.000465	0.009356
hsa-miR-214-3p/hsa-mir-214	209.3982	413.9146	4.881889	− 6.54065	1.86751	− 3.50234	0.000461	0.009356
hsa-miR-3681-5p/hsa-mir-3681	122.6533	239.8549	5.451813	− 5.71685	1.62839	− 3.51074	0.000447	0.009356
hsa-miR-549a-3p/hsa-mir-549a	27.65922	55.0541	0.264343	− 6.73678	1.957635	− 3.44129	0.000579	0.011386
hsa-miR-518c-3p/hsa-mir-518c	28.56343	0.615645	56.51122	6.366067	1.875582	3.394183	0.000688	0.013243
hsa-miR-489-3p/hsa-mir-489	42.85653	82.45848	3.254592	− 4.97892	1.494946	− 3.3305	0.000867	0.016323
hsa-miR-569/hsa-mir-569	33.94623	67.62812	0.264343	− 7.02781	2.118854	− 3.3168	0.000911	0.016788
hsa-miR-125b-5p/hsa-mir-125b-1	352,899.8	652,357.5	53,442.15	− 3.60958	1.101825	− 3.276	0.001053	0.019016
hsa-miR-125b-5p/hsa-mir-125b-2	350,374.9	647,455	53,294.82	− 3.60268	1.101886	− 3.26956	0.001077	0.019066
hsa-miR-515-5p/hsa-mir-515–1	65.98441	2.663952	129.3049	5.569219	1.723377	3.231573	0.001231	0.020953
hsa-miR-515-5p/hsa-mir-515–2	65.98441	2.663952	129.3049	5.569219	1.723377	3.231573	0.001231	0.020953
hsa-miR-9-3p/hsa-mir-9–1	2663.508	359.6608	4967.356	3.788218	1.180334	3.209446	0.00133	0.021399
hsa-miR-9-3p/hsa-mir-9–2	2663.508	359.6608	4967.356	3.788218	1.180334	3.209446	0.00133	0.021399
hsa-miR-9-3p/hsa-mir-9–3	2646.495	354.2495	4938.741	3.801755	1.181185	3.218594	0.001288	0.021399
hsa-miR-100-5p/hsa-mir-100	105,158.3	198,688.3	11,628.32	− 4.09463	1.293634	− 3.16521	0.00155	0.024491
hsa-miR-1269b/hsa-mir-1269b	359.7143	698.4631	20.96556	− 5.01586	1.59333	− 3.14803	0.001644	0.025124
hsa-miR-877-5p/hsa-mir-877	426.9675	70.2954	783.6396	3.47635	1.104468	3.147533	0.001647	0.025124
hsa-miR-1270/hsa-mir-1270	38.16059	74.95823	1.362953	− 6.59828	2.157066	− 3.05892	0.002221	0.033321
hsa-miR-142-3p/hsa-mir-142	1815.893	3446.32	185.4672	− 4.21142	1.389234	− 3.03147	0.002434	0.035392
hsa-miR-675-3p/hsa-mir-675	32.70534	1.539112	63.87158	5.291564	1.745956	3.030755	0.002439	0.035392
hsa-miR-1271-5p/hsa-mir-1271	18.66766	0.509196	36.82613	6.476359	2.15746	3.001844	0.002683	0.03825
hsa-miR-145-5p/hsa-mir-145	78.11755	150.2546	5.980499	− 4.83892	1.614368	− 2.99741	0.002723	0.03825
hsa-miR-3200-3p/hsa-mir-3200	303.3964	59.28567	547.5072	3.207259	1.090378	2.941418	0.003267	0.045178
hsa-miR-518f-3p/hsa-mir-518f	15.40462	0.509196	30.30004	6.197809	2.127855	2.912703	0.003583	0.048786
hsa-miR-139-5p/hsa-mir-139	63.34801	118.6833	8.012763	− 3.82383	1.315504	− 2.90674	0.003652	0.048972
hsa-miR-1323/hsa-mir-1323	12.55628	0	25.11257	7.456848	2.593212	2.875526	0.004034	0.053279
hsa-miR-1299/hsa-mir-1299	23.68434	45.47703	1.891639	− 4.82833	1.690588	− 2.85601	0.00429	0.055025
hsa-miR-142-5p/hsa-mir-142	186.7363	351.0203	22.45223	− 3.94603	1.379464	− 2.86056	0.004229	0.055025
hsa-miR-3115/hsa-mir-3115	55.07186	8.679377	101.4643	3.563348	1.250287	2.850024	0.004372	0.055269
hsa-miR-3059-5p/hsa-mir-3059	18.12922	0.307822	35.95062	6.597879	2.373368	2.779965	0.005436	0.067765
hsa-miR-10399-5p/hsa-mir-10399	81.04754	149.2417	12.85341	− 3.51729	1.277564	− 2.75313	0.005903	0.072557
hsa-miR-548a-3p/hsa-mir-548a-1	193.779	350.9645	36.59354	− 3.30078	1.219636	− 2.70637	0.006802	0.082467
hsa-miR-148a-3p/hsa-mir-148a	70,335.78	13,013.96	127,657.6	3.29414	1.224889	2.689338	0.007159	0.085622
hsa-miR-675-5p/hsa-mir-675	20.79439	0.615645	40.97314	5.939207	2.213066	2.683701	0.007281	0.085918
hsa-miR-548a-3p/hsa-mir-548a-2	201.0208	362.7221	39.31945	− 3.24294	1.227358	− 2.64221	0.008237	0.093454
hsa-miR-548a-3p/hsa-mir-548a-3	201.0208	362.7221	39.31945	− 3.24294	1.227358	− 2.64221	0.008237	0.093454
hsa-miR-551b-3p/hsa-mir-551b	216.8889	405.6337	28.14406	− 3.81118	1.441589	− 2.64374	0.0082	0.093454
hsa-miR-8065/hsa-mir-8065	28.68836	1.231289	56.14543	5.40265	2.056003	2.627744	0.008595	0.096289
hsa-miR-520f-3p/hsa-mir-520f	11.03321	0.307822	21.75859	5.879669	2.262605	2.598628	0.00936	0.103542
hsa-let-7a-2-3p/hsa-let-7a-2	337.4269	634.4094	40.44445	− 3.93776	1.518214	− 2.59368	0.009495	0.103747
hsa-miR-1305/hsa-mir-1305	12.92854	25.85708	0	− 6.87613	2.662493	− 2.58259	0.009806	0.105835
hsa-miR-100-3p/hsa-mir-100	7954.388	14,496.02	1412.76	− 3.3579	1.346661	− 2.4935	0.012649	0.121625
hsa-miR-1283/hsa-mir-1283–1	82.63708	15.39385	149.8803	3.304565	1.329264	2.486011	0.012918	0.121625
hsa-miR-1283/hsa-mir-1283–2	82.63708	15.39385	149.8803	3.304565	1.329264	2.486011	0.012918	0.121625
hsa-miR-147b-3p/hsa-mir-147b	360.5469	656.4143	64.67945	− 3.35027	1.336215	− 2.50728	0.012166	0.121625
hsa-miR-223-3p/hsa-mir-223	33.57809	3.386046	63.77013	4.164885	1.672477	2.49025	0.012765	0.121625
hsa-miR-517a-3p/hsa-mir-517a	23.1174	2.048307	44.18649	4.4151	1.768983	2.495841	0.012566	0.121625
hsa-miR-517a-3p/hsa-mir-517b	23.1174	2.048307	44.18649	4.4151	1.768983	2.495841	0.012566	0.121625
hsa-miR-517b-3p/hsa-mir-517a	23.1174	2.048307	44.18649	4.4151	1.768983	2.495841	0.012566	0.121625
hsa-miR-517b-3p/hsa-mir-517b	23.1174	2.048307	44.18649	4.4151	1.768983	2.495841	0.012566	0.121625
hsa-miR-6807-5p/hsa-mir-6807	12.60952	0.817018	24.40202	4.973947	1.969838	2.525053	0.011568	0.121625
hsa-miR-873-5p/hsa-mir-873	121.1677	231.0681	11.26736	− 4.35601	1.738581	− 2.5055	0.012228	0.121625
hsa-miR-887-3p/hsa-mir-887	9.489268	18.97854	0	− 6.43192	2.56478	− 2.50779	0.012149	0.121625
hsa-miR-9985/hsa-mir-9985	139.6671	245.3672	33.96702	− 2.87204	1.169878	− 2.45499	0.014089	0.131247
hsa-let-7i-3p/hsa-let-7i	1154.894	2032.462	277.3266	− 2.87853	1.174466	− 2.45093	0.014249	0.131358
hsa-miR-1262/hsa-mir-1262	77.9382	141.4369	14.43947	− 3.26519	1.334324	− 2.44707	0.014402	0.131401
hsa-miR-31-3p/hsa-mir-31	1392.042	190.1047	2593.98	3.769413	1.545296	2.439282	0.014716	0.132899
hsa-miR-1179/hsa-mir-1179	14.15206	26.67682	1.627296	− 4.46217	1.848459	− 2.41399	0.015779	0.141052
hsa-miR-526b-5p/hsa-mir-526b	9.558701	0.307822	18.80958	5.677348	2.358409	2.407279	0.016072	0.142236
hsa-miR-548f-3p/hsa-mir-548f-1	27.80862	2.971774	52.64546	4.128573	1.737472	2.376195	0.017492	0.153273
hsa-miR-551b-5p/hsa-mir-551b	12.7454	24.96212	0.528686	− 4.93349	2.120514	− 2.32655	0.019989	0.173435
hsa-miR-365a-5p/hsa-mir-365a	45.43713	10.73921	80.13506	2.891124	1.250442	2.312081	0.020773	0.178488
hsa-miR-24–2-5p/hsa-mir-24–2	105.9556	22.8392	189.0719	3.034654	1.315665	2.306554	0.02108	0.17938
hsa-miR-3929/hsa-mir-3929	25.50735	1.846934	49.16776	4.685872	2.057709	2.277228	0.022773	0.191612
hsa-miR-6844/hsa-mir-6844	18.4914	2.048307	34.93449	4.081811	1.794784	2.274263	0.02295	0.191612
hsa-miR-140-3p/hsa-mir-140	4510.326	7589.169	1431.483	− 2.40636	1.061473	− 2.267	0.02339	0.191671
hsa-miR-3614-5p/hsa-mir-3614	24.92847	45.58621	4.270724	− 3.31662	1.462529	− 2.26773	0.023346	0.191671
hsa-miR-1-3p/hsa-mir-1–2	54.94792	103.2873	6.60857	− 3.82913	1.69281	− 2.262	0.023698	0.192407
hsa-miR-126-5p/hsa-mir-126	10,120.49	2021.272	18,219.71	3.172311	1.408014	2.25304	0.024257	0.195155
hsa-miR-1468-5p/hsa-mir-1468	28.24948	50.86529	5.633678	− 3.1686	1.415195	− 2.23898	0.025157	0.200576
hsa-miR-221-5p/hsa-mir-221	5497.288	9249.968	1744.609	− 2.407	1.089069	− 2.21015	0.027095	0.214098
hsa-miR-31-5p/hsa-mir-31	15,786.38	2876.469	28,696.29	3.318442	1.510167	2.1974	0.027992	0.217998
hsa-miR-3617-5p/hsa-mir-3617	18.78337	34.57649	2.99025	− 3.8543	1.75805	− 2.19237	0.028352	0.217998
hsa-miR-452-5p/hsa-mir-452	3315.813	6032.818	598.8074	− 3.33089	1.521427	− 2.18932	0.028574	0.217998
hsa-miR-708-5p/hsa-mir-708	131.8611	17.84217	245.8801	3.779845	1.721628	2.195507	0.028127	0.217998
hsa-miR-1-3p/hsa-mir-1–1	48.86644	91.38866	6.344228	− 3.70838	1.702995	− 2.17756	0.029439	0.221081
hsa-miR-4467/hsa-mir-4467	7.191781	0.307822	14.07574	5.187369	2.382762	2.177041	0.029478	0.221081
hsa-let-7i-5p/hsa-let-7i	774,834.4	1,315,182	234,486.4	− 2.48768	1.146871	− 2.1691	0.030075	0.223669
hsa-miR-361-3p/hsa-mir-361	2938.258	4903.773	972.7433	− 2.33464	1.087029	− 2.14773	0.031736	0.23405
hsa-miR-3065-3p/hsa-mir-3065	336.539	104.3396	568.7384	2.44463	1.148159	2.129173	0.03324	0.243119
hsa-miR-183-3p/hsa-mir-183	200.134	66.73376	333.5343	2.321141	1.104056	2.102377	0.03552	0.255573
hsa-miR-3065-5p/hsa-mir-3065	1243.402	421.9095	2064.894	2.290934	1.088658	2.104366	0.035346	0.255573
hsa-miR-145-3p/hsa-mir-145	10.98776	20.61257	1.362953	− 4.70198	2.249047	− 2.09065	0.036559	0.259144
hsa-miR-7156-5p/hsa-mir-7156	24.39573	2.451054	46.34041	4.235608	2.026439	2.090173	0.036602	0.259144
hsa-miR-3911/hsa-mir-3911	15.12915	1.941859	28.31644	3.833215	1.844499	2.078188	0.037692	0.264742
hsa-miR-4485-3p/hsa-mir-4485	60.10178	103.4019	16.80165	− 2.69535	1.302334	− 2.06963	0.038487	0.268196
hsa-miR-877-3p/hsa-mir-877	12.86143	2.048307	23.67456	3.477775	1.68507	2.063876	0.039029	0.269852
hsa-miR-21-3p/hsa-mir-21	10,006.35	17,043.61	2969.089	− 2.52151	1.232564	− 2.04575	0.040781	0.277626
hsa-miR-551a/hsa-mir-551a	73.85858	12.51427	135.2029	3.426449	1.672814	2.048314	0.040529	0.277626
hsa-let-7f-1-3p/hsa-let-7f-1	247.7957	410.897	84.69445	− 2.28801	1.126941	− 2.03028	0.042328	0.279553
hsa-miR-126-3p/hsa-mir-126	21,040.36	5517.03	36,563.69	2.728523	1.343119	2.031483	0.042206	0.279553
hsa-miR-181b-3p/hsa-mir-181b-1	699.2514	1161.287	237.2161	− 2.29353	1.126	− 2.03688	0.041662	0.279553
hsa-miR-199b-5p/hsa-mir-199b	90.61121	19.07346	162.149	3.083968	1.517643	2.032078	0.042146	0.279553
hsa-miR-148a-5p/hsa-mir-148a	65.07982	18.84904	111.3106	2.551743	1.259484	2.026022	0.042763	0.280332
hsa-miR-222-5p/hsa-mir-222	896.629	1469.139	324.1194	− 2.17876	1.078294	− 2.02056	0.043325	0.28193
hsa-miR-3662/hsa-mir-3662	101.9718	28.17863	175.7649	2.626726	1.312947	2.000634	0.045432	0.293483
hsa-miR-503-5p/hsa-mir-503	1790.67	3067.249	514.0899	− 2.57522	1.290267	− 1.99588	0.045946	0.294657
hsa-miR-27b-5p/hsa-mir-27b	936.7314	1578.971	294.4919	− 2.42661	1.224466	− 1.98177	0.047505	0.302463
hsa-miR-577/hsa-mir-577	196.7154	57.98797	335.4428	2.537007	1.298334	1.954047	0.050696	0.320469
hsa-miR-224-3p/hsa-mir-224	10.4482	20.10338	0.793028	− 4.21705	2.178819	− 1.93548	0.052932	0.322863
hsa-miR-518e-5p/hsa-mir-519a-1	9.727018	1.231289	18.22275	3.79334	1.96863	1.926893	0.053993	0.322863
hsa-miR-519a-5p/hsa-mir-519a-1	9.727018	1.231289	18.22275	3.79334	1.96863	1.926893	0.053993	0.322863
hsa-miR-519b-5p/hsa-mir-519a-1	9.727018	1.231289	18.22275	3.79334	1.96863	1.926893	0.053993	0.322863
hsa-miR-519c-5p/hsa-mir-519a-1	9.727018	1.231289	18.22275	3.79334	1.96863	1.926893	0.053993	0.322863
hsa-miR-522-5p/hsa-mir-519a-1	9.727018	1.231289	18.22275	3.79334	1.96863	1.926893	0.053993	0.322863
hsa-miR-523-5p/hsa-mir-519a-1	9.727018	1.231289	18.22275	3.79334	1.96863	1.926893	0.053993	0.322863
hsa-miR-7974/hsa-mir-7974	209.4355	67.53046	351.3405	2.379416	1.230768	1.933278	0.053202	0.322863
hsa-miR-221-3p/hsa-mir-221	202,381	325,054.2	79,707.83	− 2.02787	1.076586	− 1.88361	0.059618	0.354105
hsa-miR-147b-5p/hsa-mir-147b	64.99878	114.3189	15.6787	− 2.80924	1.504791	− 1.86686	0.061921	0.362263
hsa-miR-9-5p/hsa-mir-9–1	15,085.39	5079.234	25,091.56	2.30462	1.237138	1.862864	0.062481	0.362263
hsa-miR-9-5p/hsa-mir-9–2	15,085.26	5079.234	25,091.29	2.304605	1.237137	1.862854	0.062483	0.362263
hsa-miR-9-5p/hsa-mir-9–3	15,098.14	5087.192	25,109.08	2.303369	1.23716	1.86182	0.062628	0.362263
hsa-miR-224-5p/hsa-mir-224	15,183.37	26,361.86	4004.88	− 2.71832	1.481027	− 1.83543	0.066442	0.376182
hsa-miR-27b-3p/hsa-mir-27b	180,678.2	288,713.6	72,642.75	− 1.99074	1.089503	− 1.8272	0.06767	0.376182
hsa-miR-29a-3p/hsa-mir-29a	405,497.4	662,152.4	148,842.5	− 2.15335	1.173262	− 1.83535	0.066453	0.376182
hsa-miR-301b-5p/hsa-mir-301b	84.31175	26.44088	142.1826	2.441269	1.339789	1.822129	0.068435	0.376182
hsa-miR-3125/hsa-mir-3125	9.553788	1.12484	17.98274	3.942609	2.153967	1.830394	0.067191	0.376182
hsa-miR-3613-5p/hsa-mir-3613	1994.329	3168.158	820.4995	− 1.9493	1.068292	− 1.82469	0.068048	0.376182
hsa-miR-4516/hsa-mir-4516	9.167019	0.615645	17.71839	4.699761	2.57107	1.82794	0.067559	0.376182
hsa-miR-518b/hsa-mir-518b	13.72235	1.539112	25.9056	4.03217	2.190825	1.84048	0.065698	0.376182
hsa-miR-424-5p/hsa-mir-424	3963.203	6492.625	1433.781	− 2.17802	1.198582	− 1.81716	0.069192	0.377996
hsa-miR-222-3p/hsa-mir-222	23,426.79	37,163.25	9690.326	− 1.93925	1.0728	− 1.80766	0.07066	0.381306
hsa-miR-4746-5p/hsa-mir-4746	9.126216	16.62514	1.627296	− 3.70083	2.045519	− 1.80924	0.070414	0.381306
hsa-miR-6724-5p/hsa-mir-6724–1	8.373819	1.432663	15.31498	3.384043	1.883512	1.796667	0.072388	0.381332
hsa-miR-6724-5p/hsa-mir-6724–2	8.373819	1.432663	15.31498	3.384043	1.883512	1.796667	0.072388	0.381332
hsa-miR-6724-5p/hsa-mir-6724–3	8.373819	1.432663	15.31498	3.384043	1.883512	1.796667	0.072388	0.381332
hsa-miR-6724-5p/hsa-mir-6724–4	8.373819	1.432663	15.31498	3.384043	1.883512	1.796667	0.072388	0.381332
hsa-miR-1286/hsa-mir-1286	13.65958	2.545979	24.77317	3.341195	1.866995	1.789611	0.073516	0.384983
hsa-miR-140-5p/hsa-mir-140	1690.833	2669.23	712.4361	− 1.90575	1.070763	− 1.7798	0.075108	0.391006
hsa-miR-4664-5p/hsa-mir-4664	10.96004	1.846934	20.07315	3.375196	1.901368	1.775141	0.075875	0.392685
hsa-miR-4454/hsa-mir-4454	4296.955	1342.645	7251.265	2.433357	1.384893	1.757072	0.078906	0.40365
hsa-miR-449a/hsa-mir-449a	155.7767	249.0956	62.4579	− 1.99191	1.13249	− 1.75888	0.078598	0.40365
hsa-miR-448/hsa-mir-448	6.287201	0.307822	12.26658	4.988584	2.847988	1.751617	0.07984	0.406081
hsa-miR-181a-3p/hsa-mir-181a-1	8263.135	13,445.56	3080.706	− 2.12622	1.219749	− 1.74316	0.081305	0.411171
hsa-miR-1301-3p/hsa-mir-1301	156.7831	58.96359	254.6026	2.103069	1.209706	1.738496	0.082123	0.41295
hsa-miR-21-5p/hsa-mir-21	1,598,967	2,551,494	646,438.7	− 1.98074	1.15579	− 1.71376	0.086574	0.421851
hsa-miR-301b-3p/hsa-mir-301b	463.6318	169.2149	758.0486	2.165792	1.260133	1.718701	0.085669	0.421851
hsa-miR-516a-5p/hsa-mir-516a-1	388.7021	132.6729	644.7313	2.284147	1.333593	1.712777	0.086754	0.421851
hsa-miR-516a-5p/hsa-mir-516a-2	388.7021	132.6729	644.7313	2.284147	1.333593	1.712777	0.086754	0.421851
hsa-miR-548bb-5p/hsa-mir-548bb	10.60366	2.249681	18.95763	3.030536	1.765228	1.716796	0.086016	0.421851
hsa-miR-6720-3p/hsa-mir-6720	7.048564	0.615645	13.48148	4.382591	2.546791	1.720828	0.085282	0.421851
hsa-miR-3145-3p/hsa-mir-3145	19.03517	4.309513	33.76083	2.93042	1.721544	1.702205	0.088717	0.426709
hsa-miR-324-5p/hsa-mir-324	1192.395	505.3463	1879.443	1.895625	1.112352	1.704159	0.088351	0.426709
hsa-miR-330-3p/hsa-mir-330	160.3618	67.0992	253.6244	1.920008	1.135324	1.691155	0.090807	0.434402
hsa-miR-130a-3p/hsa-mir-130a	27,373.95	10,946.5	43,801.4	2.000556	1.190147	1.680932	0.092776	0.43596
hsa-miR-3144-3p/hsa-mir-3144	229.8864	73.51191	386.2609	2.393744	1.427206	1.677224	0.093499	0.43596
hsa-miR-3187-3p/hsa-mir-3187	48.51469	17.13433	79.89504	2.224514	1.326701	1.676726	0.093596	0.43596
hsa-miR-548ad-5p/hsa-mir-548ad	90.93064	145.2339	36.62736	− 1.9719	1.175511	− 1.67749	0.093448	0.43596
hsa-miR-548ae-5p/hsa-mir-548ad	90.93064	145.2339	36.62736	− 1.9719	1.175511	− 1.67749	0.093448	0.43596
hsa-miR-3614-3p/hsa-mir-3614	10.23044	18.87482	1.586057	− 3.25526	1.961028	− 1.65998	0.096919	0.449077
hsa-miR-183-5p/hsa-mir-183	26,647.52	12,164.22	41,130.83	1.757582	1.060923	1.656654	0.097589	0.449826
hsa-miR-3691-5p/hsa-mir-3691	22.98417	5.540802	40.42754	2.849993	1.743564	1.634579	0.102137	0.46835
hsa-miR-196a-3p/hsa-mir-196a-2	193.9146	325.5119	62.31728	− 2.38703	1.469886	− 1.62395	0.104386	0.468941
hsa-miR-301a-3p/hsa-mir-301a	5307.049	2292.161	8321.937	1.860393	1.145588	1.623963	0.104384	0.468941
hsa-miR-3136-5p/hsa-mir-3136	12.76592	22.31848	3.213353	− 2.75679	1.690409	− 1.63084	0.102924	0.468941
hsa-miR-3139/hsa-mir-3139	6.733418	1.018392	12.44844	3.691032	2.266389	1.628596	0.103399	0.468941
hsa-miR-10399-3p/hsa-mir-10399	21.94535	38.3395	5.551199	− 2.61707	1.62229	− 1.6132	0.106702	0.471917
hsa-miR-10523-5p/hsa-mir-10523	12.506	3.575895	21.4361	2.599196	1.60809	1.616325	0.106024	0.471917
hsa-miR-10b-3p/hsa-mir-10b	7.160126	1.326214	12.99404	3.392884	2.106081	1.610994	0.107181	0.471917
hsa-miR-4766-5p/hsa-mir-4766	7.459485	1.231289	13.68768	3.384529	2.099517	1.612051	0.106951	0.471917
hsa-miR-503-3p/hsa-mir-503	51.27705	85.55369	17.00042	− 2.2698	1.417648	− 1.6011	0.109355	0.476744
hsa-miR-642a-3p/hsa-mir-642a	12.99015	23.33688	2.643428	− 2.89505	1.808143	− 1.60112	0.109351	0.476744
hsa-miR-193b-3p/hsa-mir-193b	152.9513	68.43694	237.4656	1.794534	1.123119	1.597814	0.110084	0.477572
hsa-miR-6510-3p/hsa-mir-6510	17.67224	5.730651	29.61382	2.345779	1.479838	1.585159	0.11293	0.487528
hsa-miR-450a-5p/hsa-mir-450a-1	115.7198	183.5037	47.93595	− 1.92875	1.222441	− 1.57779	0.114615	0.490021
hsa-miR-450a-5p/hsa-mir-450a-2	115.7198	183.5037	47.93595	− 1.92875	1.222441	− 1.57779	0.114615	0.490021
hsa-miR-210-3p/hsa-mir-210	2859.956	4474.493	1245.418	− 1.84578	1.181289	− 1.56251	0.118168	0.502781
hsa-miR-3616-3p/hsa-mir-3616	21.50045	4.309513	38.69138	3.111649	1.999035	1.556576	0.119571	0.506319
hsa-miR-744-5p/hsa-mir-744	1678.721	811.3951	2546.048	1.649915	1.067938	1.544954	0.122357	0.515649
hsa-miR-29b-1-5p/hsa-mir-29b-1	987.4942	1533.161	441.827	− 1.79228	1.173188	− 1.5277	0.126586	0.530943
hsa-miR-2110/hsa-mir-2110	57.98975	25.55198	90.42752	1.819397	1.199192	1.517186	0.12922	0.53439
hsa-miR-504-5p/hsa-mir-504	17.60346	30.71309	4.493828	− 2.58957	1.706032	− 1.51789	0.129042	0.53439
hsa-miR-92b-3p/hsa-mir-92b	1249.732	1907.297	592.1675	− 1.68873	1.110383	− 1.52086	0.128295	0.53439
hsa-miR-323a-3p/hsa-mir-323a	10.14398	2.048307	18.23965	3.16643	2.098055	1.509221	0.131242	0.54023
hsa-miR-4783-3p/hsa-mir-4783	6.338024	1.326214	11.34983	3.11573	2.082532	1.496126	0.134621	0.551571
hsa-miR-1275/hsa-mir-1275	55.9156	24.86446	86.96673	1.802014	1.212124	1.486659	0.137105	0.559161
hsa-miR-6514-5p/hsa-mir-6514	15.42689	5.411304	25.44248	2.256328	1.523324	1.481187	0.138557	0.56249
hsa-miR-181a-2-3p/hsa-mir-181a-2	1581	2373.786	788.2138	− 1.59144	1.081824	− 1.47107	0.141271	0.570891
hsa-miR-188-5p/hsa-mir-188	136.093	62.46155	209.7245	1.746229	1.189197	1.46841	0.141993	0.571199
hsa-miR-34c-3p/hsa-mir-34c	29.45867	52.04442	6.872913	− 2.80749	1.915271	− 1.46585	0.14269	0.571406
hsa-miR-561-5p/hsa-mir-561	102.1823	31.17892	173.1858	2.485722	1.715558	1.448929	0.147357	0.587438
hsa-miR-149-5p/hsa-mir-149	1360.171	606.1067	2114.235	1.803396	1.25043	1.442221	0.14924	0.592276
hsa-miR-3679-5p/hsa-mir-3679	16.13123	6.026949	26.23551	2.139727	1.495127	1.431134	0.152392	0.599408
hsa-miR-629-3p/hsa-mir-629	18.92228	30.58359	7.260974	− 2.1009	1.466651	− 1.43245	0.152015	0.599408
hsa-miR-424-3p/hsa-mir-424	98.90086	151.8156	45.98617	− 1.7277	1.211982	− 1.42552	0.154008	0.603086
hsa-miR-488-3p/hsa-mir-488	58.86875	97.59879	20.13872	− 2.33089	1.638905	− 1.42223	0.154961	0.604142
hsa-miR-181b-2-3p/hsa-mir-181b-2	105.3508	160.2047	50.4969	− 1.6514	1.164569	− 1.41804	0.15618	0.606226
hsa-miR-345-3p/hsa-mir-345	22.54625	35.67555	9.416956	− 1.94816	1.402138	− 1.38942	0.164704	0.636519
hsa-miR-335-3p/hsa-mir-335	2428.435	1005.079	3851.79	1.938821	1.399964	1.384908	0.166081	0.63905
hsa-miR-708-3p/hsa-mir-708	10.59372	2.154756	19.03268	3.12832	2.266377	1.380317	0.167489	0.641679
hsa-miR-1267/hsa-mir-1267	9.275015	16.43529	2.114743	− 2.69309	1.959973	− 1.37405	0.169427	0.642352
hsa-miR-181d-5p/hsa-mir-181d	505.6307	261.3942	749.8672	1.519342	1.107336	1.372069	0.170042	0.642352
hsa-miR-182-3p/hsa-mir-182	100.2483	50.80767	149.689	1.559283	1.137847	1.370381	0.170568	0.642352
hsa-miR-769-3p/hsa-mir-769	185.9284	93.45395	278.4029	1.571225	1.142741	1.374962	0.169143	0.642352
hsa-miR-556-3p/hsa-mir-556	22.13067	35.10873	9.152613	− 1.96729	1.443322	− 1.36303	0.172874	0.648277
hsa-miR-374b-3p/hsa-mir-374b	301.6185	442.2806	160.9563	− 1.45767	1.088008	− 1.33976	0.180324	0.673362
hsa-miR-135b-5p/hsa-mir-135b	4766.947	2389.802	7144.093	1.579883	1.188798	1.328975	0.183856	0.67343
hsa-miR-18b-5p/hsa-mir-18b	26.94844	10.10052	43.79637	2.10113	1.577261	1.332139	0.182815	0.67343
hsa-miR-22-5p/hsa-mir-22	610.6169	903.3148	317.9191	− 1.51026	1.13301	− 1.33297	0.182542	0.67343
hsa-miR-3127-5p/hsa-mir-3127	12.2281	3.788792	20.6674	2.380179	1.794618	1.326288	0.184744	0.67343
hsa-miR-491-3p/hsa-mir-491	34.73167	57.4442	12.01914	− 2.2241	1.677562	− 1.32579	0.184908	0.67343
hsa-miR-548ar-5p/hsa-mir-548ar	8.194962	14.53953	1.8504	− 2.70854	2.039401	− 1.32811	0.184143	0.67343
hsa-miR-1226-3p/hsa-mir-1226	18.63453	7.778958	29.4901	1.914422	1.453244	1.317343	0.187724	0.680883
hsa-miR-181d-3p/hsa-mir-181d	11.16832	3.990166	18.34646	2.165327	1.660826	1.303766	0.192314	0.693633
hsa-miR-4762-5p/hsa-mir-4762	6.331801	1.231289	11.43231	3.0867	2.370151	1.302322	0.192806	0.693633
hsa-miR-22-3p/hsa-mir-22	7644.675	11,101.72	4187.629	− 1.40672	1.082633	− 1.29935	0.193823	0.694466
hsa-miR-450b-5p/hsa-mir-450b	854.6039	1258.65	450.5577	− 1.47971	1.142518	− 1.29513	0.195275	0.696849
hsa-miR-23b-3p/hsa-mir-23b	28,036.53	41,360.9	14,712.17	− 1.49133	1.15358	− 1.29278	0.196087	0.696935
hsa-miR-197-3p/hsa-mir-197	371.1444	192.8888	549.4001	1.507106	1.173051	1.284775	0.198871	0.704003
hsa-miR-1343-3p/hsa-mir-1343	9.829965	3.469446	16.19048	2.22364	1.744957	1.274323	0.202549	0.711332
hsa-miR-324-3p/hsa-mir-324	329.3509	178.2998	480.4021	1.432276	1.12248	1.275992	0.201958	0.711332
hsa-miR-3611/hsa-mir-3611	28.06127	43.8803	12.24225	− 1.77579	1.398492	− 1.26979	0.20416	0.714156
hsa-miR-105-5p/hsa-mir-105–1	1644.295	645.8716	2642.717	2.032009	1.615564	1.257771	0.208475	0.72353
hsa-miR-105-5p/hsa-mir-105–2	1644.295	645.8716	2642.717	2.032009	1.615564	1.257771	0.208475	0.72353
hsa-miR-1255a/hsa-mir-1255a	38.61958	59.93316	17.306	− 1.74816	1.395822	− 1.25242	0.210415	0.727412
hsa-miR-190b-5p/hsa-mir-190b	754.5586	372.0759	1137.041	1.609782	1.303546	1.234925	0.216858	0.741907
hsa-miR-4636/hsa-mir-4636	6.531221	12.00507	1.057371	− 3.21921	2.617826	− 1.22973	0.2188	0.741907
hsa-miR-4719/hsa-mir-4719	8.060723	1.846934	14.27451	2.939963	2.383285	1.233576	0.217361	0.741907
hsa-miR-671-3p/hsa-mir-671	90.28767	133.9537	46.62166	− 1.55129	1.253567	− 1.2375	0.2159	0.741907
hsa-miR-744-3p/hsa-mir-744	43.30501	21.65674	64.95328	1.603429	1.302764	1.23079	0.218401	0.741907
hsa-miR-1257/hsa-mir-1257	9.6273	3.575895	15.6787	2.182617	1.782371	1.224558	0.220742	0.742256
hsa-miR-335-5p/hsa-mir-335	8537.127	4453.158	12,621.1	1.503096	1.225037	1.22698	0.21983	0.742256
hsa-miR-34c-5p/hsa-mir-34c	1741.157	2813.684	668.6298	− 2.07144	1.69406	− 1.22276	0.221419	0.742256
hsa-miR-3664-3p/hsa-mir-3664	20.20684	8.561404	31.85228	1.902037	1.559969	1.219279	0.222738	0.743862
hsa-let-7 g-3p/hsa-let-7 g	72.80782	106.6842	38.93139	− 1.48077	1.221763	− 1.212	0.225514	0.7503
hsa-miR-376b-3p/hsa-mir-376b	12.38916	3.895241	20.88308	2.434457	2.02714	1.200932	0.229778	0.761622
hsa-miR-328-3p/hsa-mir-328	41.73007	62.91646	20.54369	− 1.65676	1.382513	− 1.19837	0.230772	0.762065
hsa-miR-29c-3p/hsa-mir-29c	55,228.09	78,299.72	32,156.47	− 1.2839	1.07581	− 1.19343	0.232701	0.763322
hsa-miR-374c-5p/hsa-mir-374c	14.07832	21.95304	6.203603	− 1.88457	1.585558	− 1.18859	0.234603	0.763322
hsa-miR-505-5p/hsa-mir-505	33.34703	16.84956	49.8445	1.586826	1.334605	1.188986	0.234445	0.763322
hsa-miR-95-3p/hsa-mir-95	1263.976	623.6875	1904.264	1.609246	1.34994	1.192087	0.233227	0.763322
hsa-miR-10401-3p/hsa-mir-10401	12.02944	4.79566	19.26321	1.983015	1.719005	1.153583	0.248671	0.769631
hsa-miR-10a-5p/hsa-mir-10a	15,024.06	23,404.33	6643.792	− 1.81649	1.588202	− 1.14374	0.252731	0.769631
hsa-miR-127-3p/hsa-mir-127	7.694391	12.70412	2.684667	− 2.25529	1.946406	− 1.1587	0.246581	0.769631
hsa-miR-135b-3p/hsa-mir-135b	292.0133	149.4391	434.5875	1.539	1.331202	1.156098	0.247641	0.769631
hsa-miR-138-5p/hsa-mir-138–1	18,629.51	28,937.76	8321.256	− 1.79789	1.557187	− 1.15458	0.248263	0.769631
hsa-miR-23c/hsa-mir-23c	17.93627	27.55419	8.318345	− 1.7205	1.499552	− 1.14734	0.251241	0.769631
hsa-miR-26a-2-3p/hsa-mir-26a-2	277.3057	403.0456	151.5658	− 1.40303	1.190449	− 1.17857	0.238568	0.769631
hsa-miR-301a-5p/hsa-mir-301a	752.1273	414.6512	1089.603	1.395374	1.185946	1.176591	0.239359	0.769631
hsa-miR-3200-5p/hsa-mir-3200	7.131062	2.036783	12.22534	2.597866	2.256232	1.151418	0.249561	0.769631
hsa-miR-320d/hsa-mir-320d-1	630.3083	342.589	918.0276	1.421212	1.232817	1.152816	0.248986	0.769631
hsa-miR-365a-3p/hsa-mir-365a	5969.479	3533.914	8405.045	1.249999	1.069624	1.168634	0.242551	0.769631
hsa-miR-365a-3p/hsa-mir-365b	5969.765	3534.221	8405.309	1.249918	1.069632	1.16855	0.242585	0.769631
hsa-miR-365b-3p/hsa-mir-365a	5969.479	3533.914	8405.045	1.249999	1.069624	1.168634	0.242551	0.769631
hsa-miR-365b-3p/hsa-mir-365b	5969.765	3534.221	8405.309	1.249918	1.069632	1.16855	0.242585	0.769631
hsa-miR-374b-5p/hsa-mir-374b	2159.9	3040.062	1279.739	− 1.24863	1.082211	− 1.15378	0.248592	0.769631
hsa-miR-548az-5p/hsa-mir-548az	9.451198	3.587419	15.31498	2.054523	1.797583	1.142936	0.253065	0.769631
hsa-miR-548 h-3p/hsa-mir-548 h-4	52.77853	77.55364	28.00343	− 1.44175	1.259813	− 1.14442	0.252451	0.769631
hsa-miR-548z/hsa-mir-548 h-4	52.77853	77.55364	28.00343	− 1.44175	1.259813	− 1.14442	0.252451	0.769631
hsa-miR-873-3p/hsa-mir-873	13.08367	21.85538	4.311964	− 2.39383	2.06872	− 1.15715	0.24721	0.769631
hsa-let-7a-5p/hsa-let-7a-1	957,514.1	1,333,683	581,345.6	− 1.19793	1.060196	− 1.12992	0.258511	0.771112
hsa-let-7a-5p/hsa-let-7a-2	958,252.2	1,334,319	582,185.7	− 1.19654	1.060202	− 1.1286	0.259069	0.771112
hsa-let-7a-5p/hsa-let-7a-3	958,562.8	1,333,436	583,689.5	− 1.19186	1.060211	− 1.12417	0.260939	0.771112
hsa-miR-320b/hsa-mir-320b-1	1009.046	562.6604	1455.431	1.370365	1.21913	1.124052	0.260991	0.771112
hsa-miR-320b/hsa-mir-320b-2	1013.821	567.0649	1460.577	1.364204	1.217937	1.120093	0.262674	0.771112
hsa-miR-320d/hsa-mir-320d-2	615.0254	338.1011	891.9496	1.398635	1.231283	1.135916	0.255992	0.771112
hsa-miR-374a-3p/hsa-mir-374a	4314.988	6023.078	2606.897	− 1.20797	1.069034	− 1.12996	0.258491	0.771112
hsa-miR-548bc/hsa-mir-548bc	58.82536	33.04617	84.60455	1.357735	1.205599	1.126191	0.260085	0.771112
hsa-miR-548x-3p/hsa-mir-548x-2	27.91006	47.09682	8.723313	− 2.35965	2.10323	− 1.12192	0.261898	0.771112
hsa-miR-642a-5p/hsa-mir-642a	41.30546	62.17405	20.43688	− 1.54847	1.371645	− 1.12892	0.258933	0.771112
hsa-miR-654-3p/hsa-mir-654	21.86545	8.00338	35.72752	2.161493	1.931615	1.119008	0.263137	0.771112
hsa-miR-320c/hsa-mir-320c-1	845.5846	474.3303	1216.839	1.358416	1.217601	1.11565	0.264572	0.77276
hsa-miR-320c/hsa-mir-320c-2	851.0585	479.3388	1222.778	1.350285	1.216243	1.110211	0.266908	0.774793
hsa-miR-338-3p/hsa-mir-338	31.61723	13.27366	49.96079	1.938143	1.746148	1.109954	0.267019	0.774793
hsa-miR-3140-3p/hsa-mir-3140	12.37624	20.25865	4.493828	− 2.00417	1.810292	− 1.1071	0.268251	0.775824
hsa-miR-548au-5p/hsa-mir-548au	48.23616	69.19634	27.27597	− 1.37351	1.254488	− 1.09488	0.27357	0.78863
hsa-miR-6500-3p/hsa-mir-6500	7.401427	12.07695	2.725907	− 2.43312	2.229485	− 1.09134	0.275125	0.790537
hsa-miR-10b-5p/hsa-mir-10b	154.1763	71.46027	236.8924	1.735476	1.608963	1.07863	0.280753	0.798744
hsa-miR-30c-1-3p/hsa-mir-30c-1	249.5599	144.9634	354.1563	1.284578	1.193013	1.076752	0.281591	0.798744
hsa-miR-3940-3p/hsa-mir-3940	14.28611	21.88116	6.691049	− 1.67685	1.553119	− 1.07967	0.280291	0.798744
hsa-miR-760/hsa-mir-760	13.03296	6.133398	19.93252	1.683971	1.562195	1.077952	0.281055	0.798744
hsa-miR-548e-5p/hsa-mir-548e	93.84659	54.06149	133.6317	1.309066	1.22285	1.070504	0.284393	0.804114
hsa-let-7f-2-3p/hsa-let-7f-2	341.4222	477.0661	205.7783	− 1.21127	1.140736	− 1.06183	0.288314	0.810024
hsa-miR-6720-5p/hsa-mir-6720	19.01024	7.885407	30.13508	1.95526	1.841289	1.061897	0.288282	0.810024
hsa-let-7f-5p/hsa-let-7f-1	903,120	1,243,486	562,753.9	− 1.1438	1.114008	− 1.02675	0.304541	0.817952
hsa-let-7f-5p/hsa-let-7f-2	920,023.8	1,267,953	572,094.9	− 1.14816	1.115224	− 1.02954	0.303228	0.817952
hsa-miR-1180-3p/hsa-mir-1180	273.1615	166.568	379.7551	1.19195	1.149031	1.037353	0.299572	0.817952
hsa-miR-1307-3p/hsa-mir-1307	832.603	522.7072	1142.499	1.128714	1.101479	1.024726	0.305492	0.817952
hsa-miR-181c-5p/hsa-mir-181c	179.3146	110.9219	247.7074	1.157524	1.107314	1.045344	0.295864	0.817952
hsa-miR-217-5p/hsa-mir-217	23.18449	34.75481	11.61418	− 1.62324	1.551636	− 1.04615	0.295493	0.817952
hsa-miR-3144-5p/hsa-mir-3144	9.615974	3.693868	15.53808	2.040299	1.973714	1.033736	0.30126	0.817952
hsa-miR-450a-1-3p/hsa-mir-450a-1	57.63044	82.06999	33.1909	− 1.29301	1.236413	− 1.04578	0.295665	0.817952
hsa-miR-548ad-5p/hsa-mir-548ay	139.7114	193.579	85.84379	− 1.1664	1.134091	− 1.02849	0.303718	0.817952
hsa-miR-548ae-5p/hsa-mir-548ay	139.7114	193.579	85.84379	− 1.1664	1.134091	− 1.02849	0.303718	0.817952
hsa-miR-548ay-5p/hsa-mir-548ay	139.7114	193.579	85.84379	− 1.1664	1.134091	− 1.02849	0.303718	0.817952
hsa-miR-589-3p/hsa-mir-589	171.4756	100.0877	242.8634	1.28502	1.255131	1.023814	0.305923	0.817952
hsa-miR-652-3p/hsa-mir-652	162.0348	95.54502	228.5246	1.254785	1.198024	1.047379	0.294925	0.817952
hsa-miR-7-5p/hsa-mir-7–1	93,966.42	59,959.41	127,973.4	1.093797	1.063092	1.028883	0.303535	0.817952
hsa-miR-7-5p/hsa-mir-7–2	91,421.84	58,271.87	124,571.8	1.096116	1.062675	1.031469	0.302321	0.817952
hsa-miR-7-5p/hsa-mir-7–3	91,091.14	58,043.21	124,139.1	1.096768	1.062687	1.032071	0.302039	0.817952
hsa-miR-561-3p/hsa-mir-561	86.75176	38.91178	134.5917	1.802672	1.764423	1.021678	0.306933	0.818181
hsa-miR-1285-3p/hsa-mir-1285–1	6.7177	10.75073	2.684667	− 2.01272	1.983254	− 1.01486	0.310173	0.82217
hsa-miR-944/hsa-mir-944	514.3999	232.8805	795.9193	1.773555	1.748002	1.014618	0.310288	0.82217
hsa-miR-501-5p/hsa-mir-501	25.64083	14.01881	37.26285	1.385534	1.376501	1.006562	0.314145	0.829906
hsa-miR-184/hsa-mir-184	14.08876	6.452745	21.72478	1.691476	1.683817	1.004549	0.315114	0.829988
hsa-miR-195-3p/hsa-mir-195	50.90489	71.27922	30.53057	− 1.25337	1.253527	− 0.99987	0.317372	0.83099
hsa-miR-361-5p/hsa-mir-361	6116.888	8288.125	3945.652	− 1.07091	1.069872	− 1.00097	0.31684	0.83099
hsa-miR-1266-5p/hsa-mir-1266	10.15264	4.073566	16.23172	2.004242	2.033199	0.985758	0.324252	0.839469
hsa-miR-138-5p/hsa-mir-138–2	12,500.13	18,576.93	6423.335	− 1.53188	1.560999	− 0.98135	0.326421	0.839469
hsa-miR-3912-3p/hsa-mir-3912	50.64898	70.32118	30.97677	− 1.19889	1.223772	− 0.97967	0.327251	0.839469
hsa-miR-548 h-3p/hsa-mir-548z	56.56518	79.18767	33.94269	− 1.20623	1.227197	− 0.98292	0.325648	0.839469
hsa-miR-548z/hsa-mir-548z	56.56518	79.18767	33.94269	− 1.20623	1.227197	− 0.98292	0.325648	0.839469
hsa-miR-598-3p/hsa-mir-598	2804.823	3943.649	1665.997	− 1.24388	1.263769	− 0.98426	0.324986	0.839469
hsa-miR-6733-5p/hsa-mir-6733	26.97594	15.33349	38.61838	1.354998	1.374732	0.985645	0.324307	0.839469
hsa-miR-643/hsa-mir-643	43.13186	25.59808	60.66565	1.247749	1.288817	0.968136	0.332977	0.851689
hsa-miR-1268a/hsa-mir-1268a	480.145	302.391	657.899	1.121938	1.180399	0.950473	0.341872	0.866659
hsa-miR-193b-5p/hsa-mir-193b	151.2341	96.12943	206.3388	1.098305	1.154122	0.951636	0.341281	0.866659
hsa-miR-23b-5p/hsa-mir-23b	309.053	422.896	195.21	− 1.12184	1.182434	− 0.94875	0.342746	0.866659
hsa-miR-5000-3p/hsa-mir-5000	6.531184	10.33646	2.725907	− 2.19112	2.295766	− 0.95442	0.339873	0.866659
hsa-miR-181c-3p/hsa-mir-181c	267.2222	173.3549	361.0894	1.056	1.116257	0.946018	0.344139	0.867701
hsa-miR-1285-3p/hsa-mir-1285–2	6.255967	9.827266	2.684667	− 1.88252	2.014098	− 0.93467	0.349956	0.869447
hsa-miR-32-3p/hsa-mir-32	391.8009	534.7863	248.8155	− 1.10274	1.17124	− 0.94151	0.346442	0.869447
hsa-miR-545-5p/hsa-mir-545	126.3481	81.84283	170.8533	1.066225	1.13905	0.936066	0.349239	0.869447
hsa-miR-548f-3p/hsa-mir-548f-4	7.565772	2.971774	12.15977	2.061273	2.197756	0.937899	0.348296	0.869447
hsa-miR-573/hsa-mir-573	55.81498	35.26128	76.36868	1.118614	1.196565	0.934854	0.349863	0.869447
hsa-miR-629-5p/hsa-mir-629	4331.147	5894.596	2767.698	− 1.09119	1.169326	− 0.93318	0.350726	0.869447
hsa-miR-1268a/hsa-mir-1268b	496.5911	316.755	676.4273	1.095049	1.185042	0.924059	0.355456	0.87871
hsa-let-7 g-5p/hsa-let-7 g	81,128.59	107,424	54,833.15	− 0.9702	1.085346	− 0.89391	0.371371	0.878966
hsa-miR-1268b/hsa-mir-1268b	498.1429	318.4954	677.7903	1.090031	1.182875	0.92151	0.356784	0.878966
hsa-miR-181a-5p/hsa-mir-181a-1	7922.794	10,773.72	5071.87	− 1.0872	1.204545	− 0.90258	0.366746	0.878966
hsa-miR-181a-5p/hsa-mir-181a-2	7921.296	10,772.08	5070.507	− 1.08737	1.204508	− 0.90275	0.366657	0.878966
hsa-miR-29a-5p/hsa-mir-29a	998.8278	1353.303	644.3527	− 1.06857	1.172841	− 0.9111	0.362243	0.878966
hsa-miR-30e-5p/hsa-mir-30e	89,415.01	59,883.74	118,946.3	0.990073	1.102257	0.898224	0.369066	0.878966
hsa-miR-3688-3p/hsa-mir-3688–2	7.955835	11.86405	4.047621	− 1.6346	1.840337	− 0.88821	0.374429	0.878966
hsa-miR-3934-5p/hsa-mir-3934	45.74403	28.53528	62.95277	1.125346	1.264513	0.889944	0.373496	0.878966
hsa-miR-4517/hsa-mir-4517	8.59632	4.297988	12.89465	1.567515	1.758388	0.89145	0.372688	0.878966
hsa-miR-4683/hsa-mir-4683	9.721609	16.00676	3.436457	− 2.08714	2.347225	− 0.88919	0.373899	0.878966
hsa-miR-4802-5p/hsa-mir-4802	15.86628	8.892275	22.84029	1.356922	1.475347	0.91973	0.357714	0.878966
hsa-miR-4999-5p/hsa-mir-4999	21.90258	12.89396	30.9112	1.278937	1.431859	0.8932	0.37175	0.878966
hsa-miR-548ad-5p/hsa-mir-548d-2	181.3124	242.4333	120.1914	− 1.00563	1.130733	− 0.88936	0.373808	0.878966
hsa-miR-548ae-5p/hsa-mir-548d-2	181.3124	242.4333	120.1914	− 1.00563	1.130733	− 0.88936	0.373808	0.878966
hsa-miR-548ay-5p/hsa-mir-548d-2	181.3124	242.4333	120.1914	− 1.00563	1.130733	− 0.88936	0.373808	0.878966
hsa-miR-548d-5p/hsa-mir-548d-2	181.3124	242.4333	120.1914	− 1.00563	1.130733	− 0.88936	0.373808	0.878966
hsa-miR-5582-3p/hsa-mir-5582	28.63188	17.3818	39.88195	1.205459	1.314952	0.916732	0.359283	0.878966
hsa-miR-636/hsa-mir-636	13.2428	7.246714	19.23888	1.441889	1.583039	0.910836	0.362382	0.878966
hsa-miR-6868-3p/hsa-mir-6868	6.886707	2.770401	11.00301	1.946201	2.138606	0.910032	0.362805	0.878966
hsa-miR-766-5p/hsa-mir-766	28.57754	17.6781	39.47698	1.159183	1.309037	0.885524	0.375874	0.880023
hsa-miR-1322/hsa-mir-598	20.79197	29.88727	11.69666	− 1.44426	1.639969	− 0.88066	0.3785	0.883254
hsa-miR-548n/hsa-mir-548n	103.9473	139.5724	68.32211	− 1.04498	1.188453	− 0.87928	0.37925	0.883254
hsa-miR-34a-5p/hsa-mir-34a	25,397.43	16,404.71	34,390.14	1.067899	1.221634	0.874157	0.382033	0.886036
hsa-miR-548ad-5p/hsa-mir-548d-1	181.7164	241.3084	122.1243	− 0.97666	1.125314	− 0.8679	0.385451	0.886036
hsa-miR-548ae-5p/hsa-mir-548d-1	181.7164	241.3084	122.1243	− 0.97666	1.125314	− 0.8679	0.385451	0.886036
hsa-miR-548ay-5p/hsa-mir-548d-1	181.7164	241.3084	122.1243	− 0.97666	1.125314	− 0.8679	0.385451	0.886036
hsa-miR-548d-5p/hsa-mir-548d-1	181.7164	241.3084	122.1243	− 0.97666	1.125314	− 0.8679	0.385451	0.886036
hsa-miR-32-5p/hsa-mir-32	1712.632	2291.659	1133.605	− 1.01488	1.181559	− 0.85893	0.390377	0.893784
hsa-miR-582-3p/hsa-mir-582	328.4714	445.4368	211.506	− 1.0672	1.243687	− 0.85809	0.390841	0.893784
hsa-miR-10a-3p/hsa-mir-10a	784.9375	1139.195	430.68	− 1.4005	1.659696	− 0.84383	0.398766	0.897419
hsa-miR-1294/hsa-mir-1294	20.3098	28.39426	12.22534	− 1.30562	1.550646	− 0.84198	0.399797	0.897419
hsa-miR-1322/hsa-mir-1322	20.28277	28.86888	11.69666	− 1.39397	1.642385	− 0.84875	0.396022	0.897419
hsa-miR-182-5p/hsa-mir-182	74,566.91	51,949.83	97,183.99	0.903612	1.079535	0.837038	0.402571	0.897419
hsa-miR-1843/hsa-mir-1843	131.6383	90.07063	173.206	0.94114	1.122474	0.838451	0.401777	0.897419
hsa-miR-195-5p/hsa-mir-195	982.2769	1286.601	677.9532	− 0.92533	1.084672	− 0.8531	0.393606	0.897419
hsa-miR-326/hsa-mir-326	69.08455	44.43832	93.73077	1.06472	1.261851	0.843776	0.398794	0.897419
hsa-miR-340-3p/hsa-mir-340	94.35542	63.27584	125.435	0.980851	1.165015	0.841921	0.399832	0.897419
hsa-miR-378a-3p/hsa-mir-378a	5789.911	4044.439	7535.382	0.89768	1.072057	0.837344	0.402399	0.897419
hsa-miR-4504/hsa-mir-4504	12.20669	6.760567	17.65282	1.382724	1.634958	0.845724	0.397706	0.897419
hsa-miR-3176/hsa-mir-3176	11.43428	6.049998	16.81855	1.471308	1.765528	0.833353	0.404646	0.899778
hsa-miR-5187-5p/hsa-mir-5187	8.688372	4.605811	12.77093	1.502185	1.807454	0.831105	0.405914	0.900336
hsa-miR-411-5p/hsa-mir-411	32.15926	16.20813	48.11039	1.578548	1.927806	0.818831	0.412883	0.913503
hsa-miR-3935/hsa-mir-3935	21.42248	13.29671	29.54825	1.176797	1.440955	0.816678	0.414112	0.913939
hsa-miR-196a-5p/hsa-mir-196a-1	41,392.62	26,254.93	56,530.31	1.106431	1.36676	0.809528	0.418212	0.92069
hsa-miR-92b-5p/hsa-mir-92b	63.38539	83.42804	43.34274	− 0.95535	1.190344	− 0.80258	0.422215	0.927198
hsa-miR-138–1-3p/hsa-mir-138–1	111.0802	159.7756	62.3849	− 1.33749	1.674623	− 0.79868	0.424477	0.927233
hsa-miR-409-3p/hsa-mir-409	23.25177	11.0816	35.42194	1.685028	2.113865	0.797131	0.425375	0.927233
hsa-miR-7706/hsa-mir-7706	52.32856	34.60833	70.04879	1.005016	1.256326	0.799964	0.423732	0.927233
hsa-miR-1273c/hsa-mir-1273c	7.065632	10.34799	3.783278	− 1.56013	1.968667	− 0.79248	0.42808	0.928556
hsa-miR-4458/hsa-mir-4458	7.644491	11.85252	3.436457	− 1.6076	2.026204	− 0.7934	0.427543	0.928556
hsa-miR-2682-5p/hsa-mir-2682	38.25345	21.44111	55.06578	1.369633	1.733682	0.790014	0.42952	0.929401
hsa-miR-219a-5p/hsa-mir-219a-1	356.164	242.9249	469.4032	0.953825	1.213549	0.785979	0.43188	0.932228
hsa-miR-767-5p/hsa-mir-767	2531.188	1474.624	3587.752	1.282402	1.638055	0.782881	0.433697	0.933874
hsa-let-7b-3p/hsa-let-7b	236.4514	305.2406	167.6622	− 0.87052	1.146325	− 0.7594	0.447615	0.935965
hsa-miR-105-3p/hsa-mir-105–2	198.2965	117.4817	279.1114	1.244342	1.640275	0.758618	0.448081	0.935965
hsa-miR-125b-2-3p/hsa-mir-125b-2	68.01002	43.56975	92.4503	1.068171	1.408774	0.758228	0.448315	0.935965
hsa-miR-128-3p/hsa-mir-128–2	14,885.92	10,803.4	18,968.44	0.812105	1.063149	0.763868	0.444946	0.935965
hsa-miR-152-5p/hsa-mir-152	34.82033	49.5506	20.09005	− 1.24781	1.635822	− 0.7628	0.445581	0.935965
hsa-miR-196a-5p/hsa-mir-196a-2	51,060.84	33,292.88	68,828.81	1.047797	1.379182	0.759723	0.44742	0.935965
hsa-miR-330-5p/hsa-mir-330	190.037	134.3218	245.7522	0.875459	1.133425	0.772401	0.439877	0.935965
hsa-miR-331-3p/hsa-mir-331	824.9451	599.1427	1050.747	0.810587	1.069295	0.758057	0.448417	0.935965
hsa-miR-34a-3p/hsa-mir-34a	55.75356	35.5603	75.94681	1.097258	1.421846	0.771714	0.440284	0.935965
hsa-miR-3661/hsa-mir-3661	6.402336	9.59132	3.213353	− 1.53528	1.993047	− 0.77032	0.441113	0.935965
hsa-miR-4326/hsa-mir-4326	155.355	203.4375	107.2725	− 0.91138	1.197555	− 0.76103	0.446637	0.935965
hsa-miR-491-5p/hsa-mir-491	73.20547	102.7626	43.64832	− 1.2402	1.622191	− 0.76452	0.444556	0.935965
hsa-miR-6516-3p/hsa-mir-6516	13.0237	7.684034	18.36337	1.275158	1.66231	0.7671	0.443022	0.935965
hsa-miR-105-3p/hsa-mir-105–1	192.426	114.7113	270.1406	1.23156	1.637672	0.752018	0.45204	0.939097
hsa-miR-4791/hsa-mir-4791	32.09469	43.40568	20.7837	− 1.02777	1.365556	− 0.75264	0.451667	0.939097
hsa-miR-1307-5p/hsa-mir-1307	502.7411	349.8357	655.6465	0.908816	1.227386	0.740449	0.459028	0.941067
hsa-miR-365b-5p/hsa-mir-365b	12.11705	6.251371	17.98274	1.458744	1.961402	0.743725	0.457043	0.941067
hsa-miR-579-3p/hsa-mir-579	50.81252	66.7656	34.85944	− 0.93668	1.261238	− 0.74267	0.457681	0.941067
hsa-miR-615-3p/hsa-mir-615	465.2643	318.1291	612.3995	0.941882	1.266507	0.743685	0.457067	0.941067
hsa-miR-6516-5p/hsa-mir-6516	13.63711	8.299678	18.97454	1.194205	1.614036	0.739888	0.459368	0.941067
hsa-miR-660-3p/hsa-mir-660	9.108168	13.72251	4.493828	− 1.46635	1.97073	− 0.74406	0.456839	0.941067
hsa-miR-1278/hsa-mir-1278	17.40616	24.46172	10.35061	− 1.16176	1.575881	− 0.73721	0.460993	0.942215
hsa-miR-548a-5p/hsa-mir-548a-3	9.696283	13.67641	5.716156	− 1.40837	1.915906	− 0.73509	0.462284	0.942675
hsa-miR-128-3p/hsa-mir-128–1	17,353.68	12,863.61	21,843.75	0.763912	1.065259	0.717114	0.473304	0.952018
hsa-miR-16–1-3p/hsa-mir-16–1	201.7565	256.3511	147.1618	− 0.79682	1.113343	− 0.7157	0.474174	0.952018
hsa-miR-26a-5p/hsa-mir-26a-1	90,573.62	113,877.4	67,269.89	− 0.75944	1.059237	− 0.71697	0.473394	0.952018
hsa-miR-26a-5p/hsa-mir-26a-2	90,915.39	114,338.5	67,492.32	− 0.76051	1.059238	− 0.71798	0.472773	0.952018
hsa-miR-542-5p/hsa-mir-542	101.5623	129.8455	73.27905	− 0.82489	1.143372	− 0.72145	0.470632	0.952018
hsa-miR-937-3p/hsa-mir-937	10.74639	6.524621	14.96815	1.239997	1.733423	0.715346	0.474395	0.952018
hsa-miR-96-5p/hsa-mir-96	9529.353	7030.893	12,027.81	0.774636	1.068249	0.725146	0.468362	0.952018
hsa-miR-151b/hsa-mir-151b	104.9427	133.9048	75.98062	− 0.81062	1.145553	− 0.70762	0.479179	0.959003
hsa-miR-494-3p/hsa-mir-494	10.87569	6.642594	15.10878	1.242476	1.761321	0.705423	0.480547	0.959003
hsa-miR-6505-3p/hsa-mir-6505	10.11878	5.434353	14.8032	1.474539	2.093055	0.704492	0.481127	0.959003
hsa-let-7b-5p/hsa-let-7b	224,195	270,318.4	178,071.6	− 0.6022	1.09539	− 0.54975	0.582488	0.962655
hsa-let-7c-5p/hsa-let-7c	27,379.78	20,973.31	33,786.25	0.687853	1.280804	0.537048	0.591235	0.962655
hsa-miR-101-5p/hsa-mir-101–1	27.00122	34.51613	19.48631	− 0.86989	1.394452	− 0.62382	0.532745	0.962655
hsa-miR-106a-5p/hsa-mir-106a	56.85549	44.90142	68.80955	0.609157	1.259804	0.483533	0.628717	0.962655
hsa-miR-106b-3p/hsa-mir-106b	2850.795	3538.711	2162.878	− 0.7107	1.169679	− 0.6076	0.543451	0.962655
hsa-miR-1249-3p/hsa-mir-1249	6.995405	4.309513	9.681298	1.129125	2.154615	0.52405	0.600244	0.962655
hsa-miR-1255b-5p/hsa-mir-1255b-1	9.739942	13.01194	6.467945	− 1.06035	1.762034	− 0.60178	0.547324	0.962655
hsa-miR-1255b-5p/hsa-mir-1255b-2	9.739942	13.01194	6.467945	− 1.06035	1.762034	− 0.60178	0.547324	0.962655
hsa-miR-128–1-5p/hsa-mir-128–1	60.29029	43.99827	76.58231	0.782789	1.261775	0.620387	0.535003	0.962655
hsa-miR-1290/hsa-mir-1290	146.6027	178.2543	114.9512	− 0.64684	1.316245	− 0.49143	0.623122	0.962655
hsa-miR-1293/hsa-mir-1293	67.38108	90.08822	44.67394	− 0.98845	1.684816	− 0.58668	0.557417	0.962655
hsa-miR-132-3p/hsa-mir-132	1191.392	1434.256	948.5272	− 0.59678	1.13087	− 0.52771	0.597698	0.962655
hsa-miR-155-5p/hsa-mir-155	20,179.54	27,288.83	13,070.25	− 1.06193	1.837206	− 0.57801	0.563255	0.962655
hsa-miR-15a-5p/hsa-mir-15a	17,953.11	21,924.33	13,981.89	− 0.64897	1.068889	− 0.60714	0.543755	0.962655
hsa-miR-15b-3p/hsa-mir-15b	5664.526	4461.577	6867.474	0.622428	1.124568	0.553482	0.579933	0.962655
hsa-miR-17-3p/hsa-mir-17	1039.88	803.3329	1276.428	0.667938	1.091022	0.612213	0.540397	0.962655
hsa-miR-17-5p/hsa-mir-17	14,799.53	11,765.34	17,833.72	0.600062	1.091138	0.549942	0.582359	0.962655
hsa-miR-185-3p/hsa-mir-185	20.60039	14.00728	27.1935	0.943091	1.395456	0.67583	0.499149	0.962655
hsa-miR-185-5p/hsa-mir-185	6831.292	5169.517	8493.067	0.716222	1.074256	0.666715	0.504954	0.962655
hsa-miR-186-5p/hsa-mir-186	19,506.93	14,657.85	24,356.01	0.732581	1.104289	0.663395	0.507077	0.962655
hsa-miR-188-3p/hsa-mir-188	12.27192	8.394603	16.14924	0.918239	1.597121	0.574934	0.565336	0.962655
hsa-miR-18a-5p/hsa-mir-18a	5272.08	4122.058	6422.101	0.639638	1.182779	0.540793	0.588651	0.962655
hsa-miR-190a-5p/hsa-mir-190a	5504.39	4126.483	6882.297	0.738126	1.179711	0.625684	0.531522	0.962655
hsa-miR-192-3p/hsa-mir-192	31.82518	24.1078	39.54255	0.689461	1.361986	0.506217	0.612704	0.962655
hsa-miR-2116-5p/hsa-mir-2116	27.30751	35.94607	18.66895	− 0.9148	1.405895	− 0.65069	0.515248	0.962655
hsa-miR-212-5p/hsa-mir-212	15.49311	11.01246	19.97376	0.84923	1.58228	0.536713	0.591466	0.962655
hsa-miR-215-5p/hsa-mir-215	93.87212	113.9946	73.74959	− 0.63564	1.214791	− 0.52325	0.600799	0.962655
hsa-miR-2277-3p/hsa-mir-2277	13.35503	8.892275	17.81778	0.973899	1.555817	0.625973	0.531333	0.962655
hsa-miR-2278/hsa-mir-2278	36.74095	45.96591	27.51598	− 0.71758	1.288155	− 0.55706	0.577484	0.962655
hsa-miR-24–1-5p/hsa-mir-24–1	13.81228	8.584452	19.04011	1.096379	1.703046	0.643775	0.519721	0.962655
hsa-miR-25-3p/hsa-mir-25	83,921.42	102,551.7	65,291.19	− 0.65138	1.101525	− 0.59134	0.55429	0.962655
hsa-miR-25-5p/hsa-mir-25	528.733	657.8691	399.5969	− 0.72256	1.191193	− 0.60659	0.544124	0.962655
hsa-miR-28-3p/hsa-mir-28	7126.309	8848.3	5404.318	− 0.71112	1.084926	− 0.65546	0.512174	0.962655
hsa-miR-29b-3p/hsa-mir-29b-1	103,025.9	127,600.5	78,451.28	− 0.70173	1.233503	− 0.56889	0.569429	0.962655
hsa-miR-29b-3p/hsa-mir-29b-2	103,827.1	128,918.5	78,735.75	− 0.71133	1.233009	− 0.57691	0.564	0.962655
hsa-miR-29c-5p/hsa-mir-29c	870.3984	1068.472	672.3249	− 0.66978	1.149825	− 0.58251	0.560224	0.962655
hsa-miR-30e-3p/hsa-mir-30e	9241.246	6811.34	11,671.15	0.776828	1.122295	0.692178	0.488825	0.962655
hsa-miR-3149/hsa-mir-3149	9.480702	12.79904	6.162363	− 1.04316	1.739256	− 0.59977	0.548656	0.962655
hsa-miR-3171/hsa-mir-3171	7.391202	11.0816	3.700799	− 1.46955	2.461393	− 0.59704	0.550481	0.962655
hsa-miR-331-5p/hsa-mir-331	54.93784	67.8959	41.97979	− 0.70043	1.225997	− 0.57131	0.567788	0.962655
hsa-miR-339-5p/hsa-mir-339	6221	4975.877	7466.122	0.585443	1.065427	0.549492	0.582668	0.962655
hsa-miR-33a-3p/hsa-mir-33a	82.62963	66.27339	98.98587	0.581523	1.168849	0.497518	0.618824	0.962655
hsa-miR-33b-5p/hsa-mir-33b	109.991	138.3751	81.60688	− 0.7452	1.2738	− 0.58502	0.558534	0.962655
hsa-miR-340-5p/hsa-mir-340	9291.107	7529.267	11,052.95	0.553764	1.095903	0.505304	0.613346	0.962655
hsa-miR-342-5p/hsa-mir-342	124.6566	91.26462	158.0486	0.791277	1.135195	0.697041	0.485777	0.962655
hsa-miR-345-5p/hsa-mir-345	896.0998	1127.708	664.4919	− 0.76478	1.21822	− 0.62779	0.530145	0.962655
hsa-miR-34b-5p/hsa-mir-34b	328.1605	453.8345	202.4866	− 1.1597	1.946819	− 0.59569	0.551382	0.962655
hsa-miR-3688-3p/hsa-mir-3688–1	9.135633	12.37324	5.898021	− 1.05762	1.727931	− 0.61207	0.54049	0.962655
hsa-miR-374a-5p/hsa-mir-374a	21,871.42	26,305.88	17,436.96	− 0.59322	1.06226	− 0.55845	0.576535	0.962655
hsa-miR-376a-3p/hsa-mir-376a-1	56.11031	19.70063	92.51999	2.231762	4.568804	0.488478	0.625211	0.962655
hsa-miR-376a-3p/hsa-mir-376a-2	56.11031	19.70063	92.51999	2.231762	4.568804	0.488478	0.625211	0.962655
hsa-miR-378a-5p/hsa-mir-378a	779.3649	623.11	935.6198	0.58637	1.075805	0.545052	0.585718	0.962655
hsa-miR-378c/hsa-mir-378c	889.5461	709.6069	1069.485	0.591443	1.074659	0.550354	0.582077	0.962655
hsa-miR-378f/hsa-mir-378f	34.56291	25.74183	43.38398	0.743821	1.285672	0.578547	0.562895	0.962655
hsa-miR-378 g/hsa-mir-378 g	10.11191	6.536145	13.68768	1.075015	1.662995	0.646433	0.517999	0.962655
hsa-miR-378i/hsa-mir-378i	95.09757	70.42763	119.7675	0.754876	1.235969	0.610756	0.541361	0.962655
hsa-miR-379-5p/hsa-mir-379	39.6981	24.41289	54.98331	1.181671	1.881225	0.628139	0.529913	0.962655
hsa-miR-382-5p/hsa-mir-382	50.48289	32.72409	68.24169	1.069581	1.752716	0.610242	0.541702	0.962655
hsa-miR-3909/hsa-mir-3909	13.06184	16.78921	9.334477	− 0.80138	1.608605	− 0.49818	0.618355	0.962655
hsa-miR-3913-5p/hsa-mir-3913–1	51.16468	64.36338	37.96598	− 0.73032	1.342696	− 0.54392	0.586494	0.962655
hsa-miR-3913-5p/hsa-mir-3913–2	51.16468	64.36338	37.96598	− 0.73032	1.342696	− 0.54392	0.586494	0.962655
hsa-miR-3934-3p/hsa-mir-3934	21.61674	15.56944	27.66403	0.78425	1.520349	0.515836	0.605969	0.962655
hsa-miR-3939/hsa-mir-3939	8.630054	5.422829	11.83728	1.103188	1.745637	0.631969	0.527407	0.962655
hsa-miR-425-5p/hsa-mir-425	9924.872	12,081.9	7767.84	− 0.63738	1.173391	− 0.5432	0.586994	0.962655
hsa-miR-449b-5p/hsa-mir-449b	8.34945	11.02398	5.674917	− 1.02925	1.814584	− 0.56721	0.570573	0.962655
hsa-miR-450a-2-3p/hsa-mir-450a-2	13.1497	17.22926	9.070134	− 0.86958	1.678857	− 0.51796	0.604487	0.962655
hsa-miR-4645-3p/hsa-mir-4645	12.98112	17.07398	8.88827	− 0.97587	1.566191	− 0.62308	0.533229	0.962655
hsa-miR-4652-5p/hsa-mir-4652	11.50506	16.93023	6.079885	− 1.39352	2.27985	− 0.61123	0.541044	0.962655
hsa-miR-4742-3p/hsa-mir-4742	14.92895	11.02398	18.83391	0.769935	1.518666	0.506981	0.612168	0.962655
hsa-miR-4766-3p/hsa-mir-4766	57.07607	41.74859	72.40354	0.793502	1.206804	0.657524	0.510844	0.962655
hsa-miR-4775/hsa-mir-4775	61.36516	75.82741	46.90291	− 0.70614	1.201527	− 0.5877	0.556734	0.962655
hsa-miR-4787-3p/hsa-mir-4787	11.35087	6.346296	16.35544	1.310267	2.107944	0.621585	0.534215	0.962655
hsa-miR-4804-5p/hsa-mir-4804	24.59676	31.45823	17.7353	− 0.83955	1.440091	− 0.58298	0.559904	0.962655
hsa-miR-486-5p/hsa-mir-486–1	226.3837	290.3371	162.4302	− 0.8289	1.504791	− 0.55084	0.581743	0.962655
hsa-miR-486-5p/hsa-mir-486–2	231.2817	298.2829	164.2806	− 0.85164	1.507328	− 0.565	0.572075	0.962655
hsa-miR-499a-5p/hsa-mir-499a	84.18138	66.17301	102.1897	0.61436	1.268549	0.484301	0.628172	0.962655
hsa-miR-499b-5p/hsa-mir-499b	7.238844	10.20696	4.270724	− 1.18378	1.901349	− 0.6226	0.533547	0.962655
hsa-miR-5009-5p/hsa-mir-5009	10.87275	14.22018	7.525317	− 0.93499	1.6434	− 0.56894	0.5694	0.962655
hsa-miR-500a-5p/hsa-mir-500a	184.3349	134.7103	233.9595	0.790755	1.187088	0.66613	0.505328	0.962655
hsa-miR-500a-5p/hsa-mir-500b	184.3349	134.7103	233.9595	0.790755	1.187088	0.66613	0.505328	0.962655
hsa-miR-500b-5p/hsa-mir-500a	94.14795	69.66823	118.6277	0.755888	1.234589	0.612259	0.540366	0.962655
hsa-miR-500b-5p/hsa-mir-500b	94.14795	69.66823	118.6277	0.755888	1.234589	0.612259	0.540366	0.962655
hsa-miR-501-3p/hsa-mir-501	30.04524	21.86964	38.22084	0.776908	1.386079	0.560508	0.575133	0.962655
hsa-miR-514a-3p/hsa-mir-514a-1	14.17204	9.815742	18.52833	0.912459	1.511563	0.603653	0.546075	0.962655
hsa-miR-514a-3p/hsa-mir-514a-2	14.17204	9.815742	18.52833	0.912459	1.511563	0.603653	0.546075	0.962655
hsa-miR-514a-3p/hsa-mir-514a-3	14.17204	9.815742	18.52833	0.912459	1.511563	0.603653	0.546075	0.962655
hsa-miR-542-3p/hsa-mir-542	3322.91	4111.295	2534.525	− 0.69741	1.120649	− 0.62233	0.533726	0.962655
hsa-miR-548ab/hsa-mir-548ab	25.61226	32.65495	18.56957	− 0.83432	1.371196	− 0.60846	0.54288	0.962655
hsa-miR-548ak/hsa-mir-548ak	6.887552	9.686244	4.08886	− 1.40309	2.305738	− 0.60852	0.542841	0.962655
hsa-miR-548ap-3p/hsa-mir-548aa-1	33.25669	41.41772	25.09566	− 0.69843	1.307241	− 0.53428	0.593149	0.962655
hsa-miR-548ap-3p/hsa-mir-548aa-2	33.25669	41.41772	25.09566	− 0.69843	1.307241	− 0.53428	0.593149	0.962655
hsa-miR-548ap-3p/hsa-mir-548t	33.24695	41.92692	24.56697	− 0.74709	1.305289	− 0.57236	0.56708	0.962655
hsa-miR-548aq-3p/hsa-mir-548aq	111.9631	135.3809	88.54536	− 0.60267	1.187679	− 0.50744	0.611847	0.962655
hsa-miR-548aq-3p/hsa-mir-548 h-3	114.2713	137.5356	91.00693	− 0.58719	1.179655	− 0.49776	0.618651	0.962655
hsa-miR-548av-5p/hsa-mir-548 k	91.19695	73.9086	108.4853	0.552938	1.142991	0.483764	0.628553	0.962655
hsa-miR-548ay-3p/hsa-mir-548ay	13.33598	18.65919	8.012763	− 1.18756	1.702031	− 0.69773	0.485345	0.962655
hsa-miR-548b-3p/hsa-mir-548b	55.16244	37.29533	73.02956	0.94968	1.664807	0.570445	0.568376	0.962655
hsa-miR-548c-3p/hsa-mir-548c	8.641294	11.34333	5.93926	− 0.99857	1.76808	− 0.56478	0.572226	0.962655
hsa-miR-548e-3p/hsa-mir-548e	97.87365	75.20903	120.5383	0.684407	1.171932	0.583999	0.559221	0.962655
hsa-miR-548 h-5p/hsa-mir-548 h-1	93.45309	65.53158	121.3746	0.89568	1.685921	0.53127	0.595231	0.962655
hsa-miR-548 h-5p/hsa-mir-548 h-2	93.45309	65.53158	121.3746	0.89568	1.685921	0.53127	0.595231	0.962655
hsa-miR-548 h-5p/hsa-mir-548 h-3	93.45309	65.53158	121.3746	0.89568	1.685921	0.53127	0.595231	0.962655
hsa-miR-548 h-5p/hsa-mir-548 h-4	93.45309	65.53158	121.3746	0.89568	1.685921	0.53127	0.595231	0.962655
hsa-miR-548 h-5p/hsa-mir-548 h-5	93.43135	65.22376	121.6389	0.905583	1.685741	0.537202	0.591128	0.962655
hsa-miR-548 k/hsa-mir-548 k	91.19695	73.9086	108.4853	0.552938	1.142991	0.483764	0.628553	0.962655
hsa-miR-548u/hsa-mir-548u	29.04761	22.50833	35.58689	0.688598	1.418591	0.48541	0.627386	0.962655
hsa-miR-548w/hsa-mir-548w	18.64202	23.29078	13.99326	− 0.75151	1.434861	− 0.52375	0.600452	0.962655
hsa-miR-548x-3p/hsa-mir-548x	311.4521	425.3989	197.5053	− 1.10277	1.902308	− 0.5797	0.562115	0.962655
hsa-miR-5699-5p/hsa-mir-5699	22.45895	29.99099	14.92692	− 0.96581	1.436519	− 0.67233	0.501375	0.962655
hsa-miR-5701/hsa-mir-5701–1	12.17721	15.85421	8.500209	− 0.81896	1.666929	− 0.4913	0.623215	0.962655
hsa-miR-5701/hsa-mir-5701–2	12.17721	15.85421	8.500209	− 0.81896	1.666929	− 0.4913	0.623215	0.962655
hsa-miR-5701/hsa-mir-5701–3	12.17721	15.85421	8.500209	− 0.81896	1.666929	− 0.4913	0.623215	0.962655
hsa-miR-576-3p/hsa-mir-576	94.26394	119.1727	69.35515	− 0.78526	1.153191	− 0.68095	0.495906	0.962655
hsa-miR-579-5p/hsa-mir-579	24.89879	30.39374	19.40384	− 0.67432	1.371689	− 0.4916	0.623005	0.962655
hsa-miR-582-5p/hsa-mir-582	89.63233	111.7504	67.51423	− 0.70784	1.247989	− 0.56718	0.570591	0.962655
hsa-miR-625-3p/hsa-mir-625	1262.715	1584.484	940.9454	− 0.75017	1.218046	− 0.61588	0.537972	0.962655
hsa-miR-625-5p/hsa-mir-625	477.9369	603.6232	352.2506	− 0.77268	1.242508	− 0.62187	0.534028	0.962655
hsa-miR-628-5p/hsa-mir-628	546.5145	650.3422	442.6868	− 0.55659	1.096736	− 0.50749	0.611809	0.962655
hsa-miR-6505-5p/hsa-mir-6505	32.72718	24.66309	40.79127	0.752958	1.493721	0.504082	0.614204	0.962655
hsa-miR-6511a-3p/hsa-mir-6511a-1	10.19998	7.364687	13.03528	0.866614	1.710286	0.506707	0.612361	0.962655
hsa-miR-6511a-3p/hsa-mir-6511a-2	10.19998	7.364687	13.03528	0.866614	1.710286	0.506707	0.612361	0.962655
hsa-miR-6511a-3p/hsa-mir-6511a-3	10.19998	7.364687	13.03528	0.866614	1.710286	0.506707	0.612361	0.962655
hsa-miR-6511a-3p/hsa-mir-6511a-4	10.19998	7.364687	13.03528	0.866614	1.710286	0.506707	0.612361	0.962655
hsa-miR-6511b-3p/hsa-mir-6511b-1	7.064608	4.712259	9.416956	0.96338	1.89437	0.508549	0.611068	0.962655
hsa-miR-6511b-3p/hsa-mir-6511b-2	6.2281	4.096615	8.359584	0.978251	1.966913	0.497354	0.61894	0.962655
hsa-miR-651-3p/hsa-mir-651	9.491626	5.601154	13.3821	1.293089	2.061261	0.627329	0.530443	0.962655
hsa-miR-664a-3p/hsa-mir-664a	129.2991	157.1095	101.4887	− 0.63147	1.126963	− 0.56033	0.575255	0.962655
hsa-miR-664b-3p/hsa-mir-664b	16.22187	22.01066	10.43309	− 1.05464	1.515495	− 0.6959	0.486488	0.962655
hsa-miR-671-5p/hsa-mir-671	1422.612	1811.452	1033.772	− 0.81055	1.198829	− 0.67612	0.498964	0.962655
hsa-miR-6866-5p/hsa-mir-6866	11.37867	8.27663	14.48071	0.815257	1.601104	0.509184	0.610623	0.962655
hsa-miR-6873-3p/hsa-mir-6873	23.94846	16.94448	30.95244	0.896517	1.402839	0.639073	0.522775	0.962655
hsa-miR-765/hsa-mir-765	8.548218	5.39978	11.69666	1.077304	2.049155	0.525731	0.599075	0.962655
hsa-miR-7976/hsa-mir-7976	79.1316	97.43259	60.8306	− 0.6771	1.184312	− 0.57172	0.567511	0.962655
hsa-miR-99b-3p/hsa-mir-99b	1680.298	1266.601	2093.994	0.724945	1.084824	0.66826	0.503967	0.962655
hsa-miR-1292-5p/hsa-mir-1292	20.72219	15.96066	25.48372	0.672953	1.396271	0.481964	0.629831	0.962696
hsa-miR-28-5p/hsa-mir-28	371.0046	438.1522	303.8569	− 0.52541	1.103786	− 0.47601	0.634068	0.962782
hsa-miR-3138/hsa-mir-3138	9.879064	7.045341	12.71279	0.814314	1.712507	0.47551	0.634424	0.962782
hsa-miR-33a-5p/hsa-mir-33a	389.0105	314.4768	463.5443	0.562841	1.194086	0.471357	0.637386	0.962782
hsa-miR-362-5p/hsa-mir-362	175.282	143.1022	207.4617	0.5321	1.125606	0.472724	0.63641	0.962782
hsa-miR-500a-3p/hsa-mir-500a	369.5933	295.6866	443.5001	0.581127	1.222179	0.475485	0.634442	0.962782
hsa-miR-532-3p/hsa-mir-532	123.2331	97.81229	148.6539	0.593239	1.259014	0.471193	0.637503	0.962782
hsa-miR-874-3p/hsa-mir-874	18.74623	22.88803	14.60443	− 0.6993	1.466221	− 0.47694	0.633406	0.962782
hsa-miR-449c-5p/hsa-mir-449c	13.63909	16.94448	10.3337	− 0.8057	1.715817	− 0.46957	0.638662	0.962889
hsa-miR-130a-5p/hsa-mir-130a	149.8525	119.0542	180.6509	0.607961	1.30275	0.466675	0.640733	0.963735
hsa-miR-34b-3p/hsa-mir-34b	62.79119	80.86721	44.71518	− 0.83137	1.785048	− 0.46574	0.641401	0.963735
hsa-miR-574-3p/hsa-mir-574	961.1574	1124.731	797.5842	− 0.49624	1.070091	− 0.46373	0.642839	0.964259
hsa-miR-194-3p/hsa-mir-194–2	15.68892	11.5447	19.83314	0.730885	1.612739	0.453195	0.650408	0.970677
hsa-miR-3064-5p/hsa-mir-3064	71.873	58.50596	85.24005	0.541933	1.188087	0.456139	0.64829	0.970677
hsa-miR-767-3p/hsa-mir-767	102.9178	74.80082	131.0349	0.801715	1.76729	0.453641	0.650087	0.970677
hsa-miR-103a-3p/hsa-mir-103a-1	120,205.5	101,799.9	138,611.1	0.445311	1.088137	0.409242	0.682362	0.971219
hsa-miR-103a-3p/hsa-mir-103a-2	120,842.7	102,096.4	139,589.1	0.451259	1.088076	0.414731	0.678339	0.971219
hsa-miR-12136/hsa-mir-12136	73.34812	87.32055	59.37569	− 0.57981	1.297387	− 0.44691	0.654942	0.971219
hsa-miR-1260a/hsa-mir-1260a	359.9128	416.7341	303.0915	− 0.46316	1.144024	− 0.40485	0.685587	0.971219
hsa-miR-1260b/hsa-mir-1260b	369.4752	429.4152	309.5352	− 0.47554	1.127539	− 0.42175	0.673206	0.971219
hsa-miR-1303/hsa-mir-1303	21.76757	16.31731	27.21783	0.705557	1.664306	0.423935	0.671613	0.971219
hsa-miR-132-5p/hsa-mir-132	244.9572	287.524	202.3905	− 0.50702	1.134503	− 0.44691	0.654939	0.971219
hsa-miR-151b/hsa-mir-151a	6445.772	5427.313	7464.231	0.459975	1.171828	0.392528	0.694668	0.971219
hsa-miR-152-3p/hsa-mir-152	17,118.57	20,501.83	13,735.32	− 0.57774	1.441187	− 0.40088	0.688508	0.971219
hsa-miR-155-3p/hsa-mir-155	9.218129	12.62072	5.815542	− 1.0372	2.357763	− 0.43991	0.660004	0.971219
hsa-miR-15a-3p/hsa-mir-15a	75.28539	87.76606	62.80472	− 0.48062	1.207041	− 0.39818	0.690498	0.971219
hsa-miR-191-5p/hsa-mir-191	36,741.98	42,293.49	31,190.47	− 0.43935	1.07876	− 0.40727	0.683811	0.971219
hsa-miR-194-5p/hsa-mir-194–2	1400.399	1181.963	1618.834	0.452866	1.152613	0.392904	0.69439	0.971219
hsa-miR-2116-3p/hsa-mir-2116	8.376942	10.63276	6.121124	− 0.71809	1.808162	− 0.39714	0.691266	0.971219
hsa-miR-219b-3p/hsa-mir-219b	8.849646	6.737518	10.96177	0.737185	1.745819	0.422257	0.672837	0.971219
hsa-miR-27a-5p/hsa-mir-27a	3577.927	4161.164	2994.691	− 0.47449	1.063937	− 0.44598	0.655613	0.971219
hsa-miR-30c-5p/hsa-mir-30c-1	35,708.99	29,892.2	41,525.79	0.47423	1.09971	0.431232	0.6663	0.971219
hsa-miR-30c-5p/hsa-mir-30c-2	35,738.41	29,921.24	41,555.59	0.473864	1.099632	0.43093	0.666519	0.971219
hsa-miR-3129-3p/hsa-mir-3129	8.624224	11.69725	5.551199	− 0.99326	2.384871	− 0.41648	0.677057	0.971219
hsa-miR-339-3p/hsa-mir-339	793.6751	669.398	917.9521	0.455774	1.073855	0.424428	0.671254	0.971219
hsa-miR-369-3p/hsa-mir-369	10.54551	7.873883	13.21714	0.807253	1.939151	0.416292	0.677196	0.971219
hsa-miR-422a/hsa-mir-422a	7.034243	5.221455	8.847031	0.755034	1.869869	0.40379	0.686367	0.971219
hsa-miR-423-3p/hsa-mir-423	1217.22	1396.156	1038.283	− 0.4279	1.080588	− 0.39599	0.69211	0.971219
hsa-miR-4286/hsa-mir-4286	57.78688	46.8636	68.71017	0.534414	1.260293	0.42404	0.671537	0.971219
hsa-miR-4798-5p/hsa-mir-4798	12.60519	15.40537	9.805016	− 0.74375	1.858656	− 0.40015	0.689043	0.971219
hsa-miR-497-3p/hsa-mir-497	8.205283	10.20696	6.203603	− 0.762	1.804768	− 0.42221	0.672869	0.971219
hsa-miR-548aa/hsa-mir-548aa-1	43.5053	51.56433	35.44627	− 0.51758	1.286906	− 0.40219	0.687547	0.971219
hsa-miR-548aa/hsa-mir-548aa-2	43.5053	51.56433	35.44627	− 0.51758	1.286906	− 0.40219	0.687547	0.971219
hsa-miR-548aa/hsa-mir-548t	43.36339	52.07353	34.65324	− 0.56433	1.284171	− 0.43945	0.660336	0.971219
hsa-miR-548aq-5p/hsa-mir-548aq	15.34165	18.96974	11.71356	− 0.63456	1.607029	− 0.39487	0.692941	0.971219
hsa-miR-548b-5p/hsa-mir-548b	43.35578	51.7284	34.98316	− 0.57737	1.304239	− 0.44269	0.657991	0.971219
hsa-miR-548t-3p/hsa-mir-548aa-1	43.5053	51.56433	35.44627	− 0.51758	1.286906	− 0.40219	0.687547	0.971219
hsa-miR-548t-3p/hsa-mir-548aa-2	43.5053	51.56433	35.44627	− 0.51758	1.286906	− 0.40219	0.687547	0.971219
hsa-miR-548t-3p/hsa-mir-548t	43.36339	52.07353	34.65324	− 0.56433	1.284171	− 0.43945	0.660336	0.971219
hsa-miR-550a-3-5p/hsa-mir-550a-1	6.656108	4.605811	8.706406	0.822748	2.054721	0.400419	0.688848	0.971219
hsa-miR-550a-3-5p/hsa-mir-550a-2	6.656108	4.605811	8.706406	0.822748	2.054721	0.400419	0.688848	0.971219
hsa-miR-550a-5p/hsa-mir-550a-1	6.78828	4.605811	8.970749	0.869868	2.00864	0.433063	0.664969	0.971219
hsa-miR-550a-5p/hsa-mir-550a-2	6.78828	4.605811	8.970749	0.869868	2.00864	0.433063	0.664969	0.971219
hsa-miR-597-5p/hsa-mir-597	19.36322	15.43994	23.2865	0.603845	1.4128	0.427411	0.66908	0.971219
hsa-miR-98-5p/hsa-mir-98	29,938.12	24,838.65	35,037.58	0.496293	1.177255	0.421568	0.67334	0.971219
hsa-miR-151a-5p/hsa-mir-151a	6493.016	5473.138	7512.894	0.457219	1.171922	0.390145	0.69643	0.972145
hsa-let-7a-3p/hsa-let-7a-1	2402.517	2700.391	2104.643	− 0.3589	1.152525	− 0.3114	0.755496	0.975159
hsa-let-7a-3p/hsa-let-7a-3	2372.944	2688.504	2057.384	− 0.38528	1.151592	− 0.33457	0.737952	0.975159
hsa-let-7c-3p/hsa-let-7c	72.48019	60.2152	84.74518	0.493332	1.481465	0.333003	0.739132	0.975159
hsa-let-7d-5p/hsa-let-7d	16,789.4	19,232.32	14,346.48	− 0.4229	1.147565	− 0.36852	0.712485	0.975159
hsa-miR-101–2-5p/hsa-mir-101–2	21.86693	24.98244	18.75143	− 0.40842	1.414311	− 0.28878	0.772752	0.975159
hsa-miR-101-3p/hsa-mir-101–1	34,555.72	31,451.55	37,659.88	0.259903	1.071394	0.242584	0.808328	0.975159
hsa-miR-101-3p/hsa-mir-101–2	35,357.27	32,097.14	38,617.41	0.266812	1.072198	0.248845	0.80348	0.975159
hsa-miR-103a-2-5p/hsa-mir-103a-2	21.60935	19.58539	23.63332	0.277058	1.436882	0.192819	0.847101	0.975159
hsa-miR-106b-5p/hsa-mir-106b	3114.046	2930.708	3297.384	0.170056	1.101915	0.154328	0.877351	0.975159
hsa-miR-107/hsa-mir-107	10,688.04	9747.91	11,628.17	0.254398	1.136587	0.223826	0.822892	0.975159
hsa-miR-12135/hsa-mir-12135	4690.544	5011.755	4369.333	− 0.19758	1.141	− 0.17317	0.862521	0.975159
hsa-miR-1246/hsa-mir-1246	323.7635	286.8804	360.6465	0.32562	1.378542	0.236206	0.813273	0.975159
hsa-miR-1248/hsa-mir-1248	9.438306	10.82261	8.054002	− 0.42194	1.706271	− 0.24729	0.804687	0.975159
hsa-miR-1252-5p/hsa-mir-1252	7.466119	9.116697	5.815542	− 0.54682	2.094914	− 0.26102	0.794076	0.975159
hsa-miR-125a-3p/hsa-mir-125a	318.191	296.0202	340.3618	0.197122	1.175686	0.167665	0.866847	0.975159
hsa-miR-125a-5p/hsa-mir-125a	84,528.55	90,004.06	79,053.04	− 0.18717	1.132152	− 0.16533	0.868687	0.975159
hsa-miR-1276/hsa-mir-1276	9.651318	7.341639	11.961	0.651663	1.877358	0.347117	0.728504	0.975159
hsa-miR-1284/hsa-mir-1284	8.177782	9.887618	6.467945	− 0.63602	1.880478	− 0.33822	0.735197	0.975159
hsa-miR-129-5p/hsa-mir-129–1	52.07205	56.93561	47.2085	− 0.28417	1.54201	− 0.18429	0.853788	0.975159
hsa-miR-129-5p/hsa-mir-129–2	52.07205	56.93561	47.2085	− 0.28417	1.54201	− 0.18429	0.853788	0.975159
hsa-miR-1296-5p/hsa-mir-1296	136.3506	117.8174	154.8839	0.392206	1.154095	0.339839	0.733978	0.975159
hsa-miR-1306-3p/hsa-mir-1306	51.00782	45.12585	56.88979	0.326081	1.244919	0.261929	0.793376	0.975159
hsa-miR-1306-5p/hsa-mir-1306	23.35222	20.73328	25.97117	0.339188	1.367958	0.247952	0.804171	0.975159
hsa-miR-130b-5p/hsa-mir-130b	363.8084	388.3453	339.2714	− 0.19121	1.152764	− 0.16587	0.868259	0.975159
hsa-miR-134-5p/hsa-mir-134	11.05025	8.619025	13.48148	0.676694	2.281615	0.296586	0.766783	0.975159
hsa-miR-135a-5p/hsa-mir-135a-1	14.99308	13.30824	16.67793	0.305614	1.517615	0.201378	0.840403	0.975159
hsa-miR-135a-5p/hsa-mir-135a-2	15.67456	13.30824	18.04088	0.414262	1.505289	0.275204	0.783159	0.975159
hsa-miR-137-3p/hsa-mir-137	5699.61	5099.317	6299.902	0.305199	1.576121	0.19364	0.846458	0.975159
hsa-miR-137-5p/hsa-mir-137	13.76968	15.37959	12.15977	− 0.28116	1.933733	− 0.1454	0.884398	0.975159
hsa-miR-146b-3p/hsa-mir-146b	9.650696	8.785826	10.51557	0.23034	1.687331	0.136511	0.891417	0.975159
hsa-miR-146b-5p/hsa-mir-146b	2018.64	2246.607	1790.674	− 0.32691	1.073358	− 0.30457	0.760693	0.975159
hsa-miR-148b-3p/hsa-mir-148b	25,683.18	23,501.91	27,864.46	0.245699	1.107711	0.221808	0.824463	0.975159
hsa-miR-148b-5p/hsa-mir-148b	336.2858	308.0734	364.4982	0.245355	1.119835	0.219099	0.826573	0.975159
hsa-miR-15b-5p/hsa-mir-15b	20,192.72	18,163.35	22,222.08	0.290989	1.073935	0.270956	0.786425	0.975159
hsa-miR-16–2-3p/hsa-mir-16–2	734.3944	813.0828	655.7059	− 0.30954	1.08625	− 0.28496	0.775673	0.975159
hsa-miR-16-5p/hsa-mir-16–1	1,069,612	1,126,632	1,012,592	− 0.15395	1.059609	− 0.14529	0.884481	0.975159
hsa-miR-16-5p/hsa-mir-16–2	1,068,261	1,123,401	1,013,121	− 0.14906	1.059605	− 0.14067	0.88813	0.975159
hsa-miR-181b-5p/hsa-mir-181b-1	6213.037	6766.708	5659.366	− 0.25803	1.193161	− 0.21625	0.828789	0.975159
hsa-miR-181b-5p/hsa-mir-181b-2	7380.871	8357.396	6404.347	− 0.38418	1.199503	− 0.32029	0.748751	0.975159
hsa-miR-186-3p/hsa-mir-186	42.12615	36.82071	47.4316	0.350295	1.481643	0.236423	0.813104	0.975159
hsa-miR-191-3p/hsa-mir-191	392.5965	441.8949	343.298	− 0.36493	1.086182	− 0.33597	0.73689	0.975159
hsa-miR-193a-3p/hsa-mir-193a	12.82338	15.04872	10.59804	− 0.6011	1.710177	− 0.35148	0.725226	0.975159
hsa-miR-193a-5p/hsa-mir-193a	269.8862	212.9691	326.8032	0.613814	1.658634	0.370072	0.711329	0.975159
hsa-miR-194-5p/hsa-mir-194–1	958.9089	869.2575	1048.56	0.26918	1.153241	0.233411	0.815442	0.975159
hsa-miR-19a-3p/hsa-mir-19a	13,253.77	12,197.19	14,310.34	0.230531	1.124309	0.205042	0.837539	0.975159
hsa-miR-19b-1-5p/hsa-mir-19b-1	146.2036	136.7641	155.6431	0.181367	1.196793	0.151544	0.879546	0.975159
hsa-miR-19b-3p/hsa-mir-19b-1	61,601.14	53,974.42	69,227.86	0.359083	1.071943	0.334983	0.737638	0.975159
hsa-miR-19b-3p/hsa-mir-19b-2	62,157.61	54,702.77	69,612.45	0.347737	1.072116	0.324346	0.745676	0.975159
hsa-miR-20a-5p/hsa-mir-20a	78,862.17	71,078.9	86,645.45	0.285706	1.097234	0.260387	0.794565	0.975159
hsa-miR-2277-5p/hsa-mir-2277	20.63283	16.81499	24.45068	0.511421	1.66285	0.307557	0.75842	0.975159
hsa-miR-2355-5p/hsa-mir-2355	69.03251	61.80314	76.26187	0.309701	1.329993	0.232859	0.815871	0.975159
hsa-miR-23a-5p/hsa-mir-23a	71.85204	64.13895	79.56513	0.306746	1.17425	0.261227	0.793918	0.975159
hsa-miR-24-3p/hsa-mir-24–1	119,140.1	102,555.8	135,724.4	0.404283	1.067961	0.378556	0.705018	0.975159
hsa-miR-24-3p/hsa-mir-24–2	117,716.3	101,285.4	134,147.3	0.405405	1.067902	0.379627	0.704222	0.975159
hsa-miR-2682-3p/hsa-mir-2682	65.27841	56.01214	74.54467	0.42181	1.881545	0.224183	0.822615	0.975159
hsa-miR-26b-3p/hsa-mir-26b	117.0974	125.1882	109.0066	− 0.19347	1.152019	− 0.16794	0.866632	0.975159
hsa-miR-30a-5p/hsa-mir-30a	178,908.2	204,730.6	153,085.8	− 0.41938	1.126075	− 0.37243	0.709575	0.975159
hsa-miR-30b-3p/hsa-mir-30b	63.31813	70.77002	55.86624	− 0.33114	1.198784	− 0.27623	0.782373	0.975159
hsa-miR-30d-3p/hsa-mir-30d	910.1709	963.7144	856.6275	− 0.16827	1.159071	− 0.14518	0.884572	0.975159
hsa-miR-3128/hsa-mir-3128	20.1419	17.91405	22.36975	0.347973	1.468618	0.236939	0.812704	0.975159
hsa-miR-3129-5p/hsa-mir-3129	29.38763	36.32303	22.45223	− 0.68743	1.827578	− 0.37614	0.706811	0.975159
hsa-miR-3130-5p/hsa-mir-3130–2	7.63102	6.026949	9.235091	0.539722	1.905408	0.283258	0.776979	0.975159
hsa-miR-3146/hsa-mir-3146	6.823632	7.790483	5.856781	− 0.35938	2.00462	− 0.17928	0.857721	0.975159
hsa-miR-3164/hsa-mir-3164	10.1022	8.71395	11.49046	0.407359	1.778459	0.229052	0.818829	0.975159
hsa-miR-3173-5p/hsa-mir-3173	25.21982	26.80632	23.63332	− 0.18765	1.347423	− 0.13927	0.889239	0.975159
hsa-miR-3174/hsa-mir-3174	22.14035	24.00135	20.27934	− 0.2793	1.420264	− 0.19665	0.844099	0.975159
hsa-miR-320a-3p/hsa-mir-320a	6030.095	5702.176	6358.015	0.156864	1.141738	0.13739	0.890722	0.975159
hsa-miR-320e/hsa-mir-320e	23.6538	21.00652	26.30108	0.295786	1.518957	0.194729	0.845605	0.975159
hsa-miR-33b-3p/hsa-mir-33b	60.76664	66.07202	55.46127	− 0.2462	1.202101	− 0.2048	0.837725	0.975159
hsa-miR-342-3p/hsa-mir-342	5354.744	4778.463	5931.026	0.311842	1.074909	0.29011	0.771732	0.975159
hsa-miR-3605-3p/hsa-mir-3605	84.54797	89.48956	79.60637	− 0.17352	1.157535	− 0.1499	0.880841	0.975159
hsa-miR-3615/hsa-mir-3615	116.3061	99.25648	133.3558	0.414747	1.244003	0.333397	0.738835	0.975159
hsa-miR-362-3p/hsa-mir-362	97.9328	87.40668	108.4589	0.298299	1.250104	0.238619	0.811401	0.975159
hsa-miR-3657/hsa-mir-3657	17.65141	14.96532	20.33749	0.443204	1.553123	0.285363	0.775366	0.975159
hsa-miR-3667-5p/hsa-mir-3667	6.25207	4.309513	8.194627	0.964984	2.523387	0.382416	0.702153	0.975159
hsa-miR-3684/hsa-mir-3684	27.1156	30.84531	23.38589	− 0.35446	1.417002	− 0.25015	0.802471	0.975159
hsa-miR-376c-3p/hsa-mir-376c	21.05799	17.22653	24.88946	0.55939	1.717075	0.325781	0.74459	0.975159
hsa-miR-378d/hsa-mir-378d-1	155.5216	137.3255	173.7178	0.335491	1.122315	0.298928	0.764995	0.975159
hsa-miR-378d/hsa-mir-378d-2	157.6701	138.4503	176.8899	0.350199	1.119443	0.312834	0.754407	0.975159
hsa-miR-381-3p/hsa-mir-381	16.33364	12.31289	20.3544	0.747727	2.183265	0.342481	0.731989	0.975159
hsa-miR-3928-3p/hsa-mir-3928	12.14766	14.34968	9.945641	− 0.55082	1.680069	− 0.32786	0.74302	0.975159
hsa-miR-3942-5p/hsa-mir-3942	7.188661	7.991856	6.385467	− 0.2666	1.945419	− 0.13704	0.890998	0.975159
hsa-miR-423-5p/hsa-mir-423	2035.507	2268.934	1802.08	− 0.33274	1.085286	− 0.30659	0.759157	0.975159
hsa-miR-425-3p/hsa-mir-425	1127.805	1232.854	1022.757	− 0.2697	1.095245	− 0.24625	0.805491	0.975159
hsa-miR-4448/hsa-mir-4448	38.11153	44.47017	31.7529	− 0.52623	1.450169	− 0.36287	0.7167	0.975159
hsa-miR-4477b/hsa-mir-4477b	7.579884	8.774301	6.385467	− 0.37527	1.895076	− 0.19802	0.843027	0.975159
hsa-miR-4484/hsa-mir-4484	29.07339	32.64615	25.50063	− 0.31249	1.454214	− 0.21488	0.829857	0.975159
hsa-miR-4510/hsa-mir-4510	7.278635	6.156446	8.400824	0.376325	2.206994	0.170515	0.864605	0.975159
hsa-miR-454-5p/hsa-mir-454	348.3187	382.7354	313.902	− 0.28312	1.13371	− 0.24973	0.802797	0.975159
hsa-miR-455-5p/hsa-mir-455	4145.206	4546.749	3743.662	− 0.28001	1.517119	− 0.18457	0.853569	0.975159
hsa-miR-4662a-5p/hsa-mir-4662a	72.84816	58.46319	87.23313	0.585718	1.83182	0.319747	0.74916	0.975159
hsa-miR-4668-5p/hsa-mir-4668	14.84362	12.56309	17.12414	0.468245	1.507368	0.310638	0.756076	0.975159
hsa-miR-4677-3p/hsa-mir-4677	186.7085	214.3672	159.0498	− 0.42394	1.17593	− 0.36052	0.718462	0.975159
hsa-miR-4677-5p/hsa-mir-4677	32.73498	36.40916	29.0608	− 0.29946	1.322366	− 0.22646	0.820846	0.975159
hsa-miR-4742-5p/hsa-mir-4742	9.883544	8.27663	11.49046	0.504254	1.677021	0.300684	0.763655	0.975159
hsa-miR-4797-3p/hsa-mir-4797	8.168027	9.686244	6.64981	− 0.45817	1.898373	− 0.24135	0.809286	0.975159
hsa-miR-484/hsa-mir-484	201.2699	170.95	231.5899	0.433415	1.141077	0.37983	0.704072	0.975159
hsa-miR-497-5p/hsa-mir-497	577.2343	652.1133	502.3553	− 0.37625	1.075999	− 0.34968	0.726582	0.975159
hsa-miR-5008-5p/hsa-mir-5008	10.48326	11.5908	9.375716	− 0.30752	1.854483	− 0.16583	0.868294	0.975159
hsa-miR-500b-3p/hsa-mir-500b	12.20258	10.94058	13.46458	0.276707	1.57459	0.175733	0.860504	0.975159
hsa-miR-5010-3p/hsa-mir-5010	7.142283	6.536145	7.74842	0.286692	1.855457	0.154513	0.877206	0.975159
hsa-miR-502-3p/hsa-mir-502	144.7166	131.8911	157.5421	0.247031	1.229882	0.200857	0.84081	0.975159
hsa-miR-532-5p/hsa-mir-532	3691.382	3455.326	3927.438	0.184558	1.084431	0.170188	0.864862	0.975159
hsa-miR-545-3p/hsa-mir-545	40.92929	34.55071	47.30788	0.449097	1.238301	0.362672	0.71685	0.975159
hsa-miR-548aj-5p/hsa-mir-548aj-2	43.50628	47.55991	39.45265	− 0.25574	1.39406	− 0.18345	0.854443	0.975159
hsa-miR-548aj-5p/hsa-mir-548 g	43.32313	48.06911	38.57714	− 0.29805	1.412233	− 0.21105	0.83285	0.975159
hsa-miR-548aj-5p/hsa-mir-548x	61.89905	71.47513	52.32297	− 0.43394	1.474768	− 0.29424	0.768573	0.975159
hsa-miR-548am-5p/hsa-mir-548c	141.0581	148.5278	133.5884	− 0.15729	1.126058	− 0.13968	0.888909	0.975159
hsa-miR-548am-5p/hsa-mir-548o-2	141.1903	148.5278	133.8527	− 0.15439	1.12575	− 0.13714	0.890919	0.975159
hsa-miR-548ap-5p/hsa-mir-548j	98.10835	107.887	88.32968	− 0.29052	1.168885	− 0.24855	0.803711	0.975159
hsa-miR-548ar-5p/hsa-mir-548aj-2	41.83551	46.94427	36.72674	− 0.33492	1.418328	− 0.23614	0.813325	0.975159
hsa-miR-548ar-5p/hsa-mir-548 g	43.0153	47.45347	38.57714	− 0.27947	1.411618	− 0.19798	0.843064	0.975159
hsa-miR-548ar-5p/hsa-mir-548x	61.59123	70.85949	52.32297	− 0.42147	1.47424	− 0.28589	0.774963	0.975159
hsa-miR-548au-5p/hsa-mir-548c	140.7937	148.5278	133.0597	− 0.16312	1.126685	− 0.14478	0.884885	0.975159
hsa-miR-548au-5p/hsa-mir-548o-2	140.7937	148.5278	133.0597	− 0.16312	1.126685	− 0.14478	0.884885	0.975159
hsa-miR-548aw/hsa-mir-548aw	9.582472	11.63963	7.525317	− 0.63585	1.703054	− 0.37336	0.708883	0.975159
hsa-miR-548c-5p/hsa-mir-548c	141.0581	148.5278	133.5884	− 0.15729	1.126058	− 0.13968	0.888909	0.975159
hsa-miR-548c-5p/hsa-mir-548o-2	141.1903	148.5278	133.8527	− 0.15439	1.12575	− 0.13714	0.890919	0.975159
hsa-miR-548d-3p/hsa-mir-548d-1	14.52317	11.86405	17.18228	0.585921	1.83091	0.320016	0.748956	0.975159
hsa-miR-548d-3p/hsa-mir-548d-2	14.65534	11.86405	17.44663	0.60729	1.829208	0.331996	0.739892	0.975159
hsa-miR-548 g-5p/hsa-mir-548aj-2	43.50628	47.55991	39.45265	− 0.25574	1.39406	− 0.18345	0.854443	0.975159
hsa-miR-548 g-5p/hsa-mir-548 g	43.32313	48.06911	38.57714	− 0.29805	1.412233	− 0.21105	0.83285	0.975159
hsa-miR-548 g-5p/hsa-mir-548x	61.89905	71.47513	52.32297	− 0.43394	1.474768	− 0.29424	0.768573	0.975159
hsa-miR-548j-5p/hsa-mir-548j	98.10835	107.887	88.32968	− 0.29052	1.168885	− 0.24855	0.803711	0.975159
hsa-miR-548 l/hsa-mir-548 l	17.09394	15.86574	18.32213	0.250501	1.562973	0.160272	0.872667	0.975159
hsa-miR-548o-5p/hsa-mir-548c	141.0581	148.5278	133.5884	− 0.15729	1.126058	− 0.13968	0.888909	0.975159
hsa-miR-548o-5p/hsa-mir-548o-2	141.1903	148.5278	133.8527	− 0.15439	1.12575	− 0.13714	0.890919	0.975159
hsa-miR-548q/hsa-mir-548q	15.88009	19.17111	12.58907	− 0.58441	1.563019	− 0.3739	0.70848	0.975159
hsa-miR-548t-5p/hsa-mir-548t	13.41151	14.53953	12.28349	− 0.21731	1.586189	− 0.137	0.89103	0.975159
hsa-miR-548x-5p/hsa-mir-548aj-2	43.50628	47.55991	39.45265	− 0.25574	1.39406	− 0.18345	0.854443	0.975159
hsa-miR-548x-5p/hsa-mir-548 g	43.32313	48.06911	38.57714	− 0.29805	1.412233	− 0.21105	0.83285	0.975159
hsa-miR-548x-5p/hsa-mir-548x	61.89905	71.47513	52.32297	− 0.43394	1.474768	− 0.29424	0.768573	0.975159
hsa-miR-548y/hsa-mir-548y	10.89114	13.852	7.930284	− 0.74182	2.296171	− 0.32307	0.746644	0.975159
hsa-miR-550a-3p/hsa-mir-550a-1	32.59571	28.90346	36.28796	0.301916	1.358317	0.222272	0.824102	0.975159
hsa-miR-550a-3p/hsa-mir-550a-2	32.59571	28.90346	36.28796	0.301916	1.358317	0.222272	0.824102	0.975159
hsa-miR-550a-3p/hsa-mir-550a-3	32.59571	28.90346	36.28796	0.301916	1.358317	0.222272	0.824102	0.975159
hsa-miR-5583-3p/hsa-mir-5583–1	8.710016	10.24154	7.178495	− 0.44676	1.83288	− 0.24375	0.807428	0.975159
hsa-miR-5583-3p/hsa-mir-5583–2	8.710016	10.24154	7.178495	− 0.44676	1.83288	− 0.24375	0.807428	0.975159
hsa-miR-5699-3p/hsa-mir-5699	102.8521	111.4426	94.26152	− 0.23322	1.161812	− 0.20074	0.840906	0.975159
hsa-miR-576-5p/hsa-mir-576	437.0624	495.7814	378.3435	− 0.38743	1.11878	− 0.34629	0.729123	0.975159
hsa-miR-580-3p/hsa-mir-580	44.74551	48.02301	41.46801	− 0.20519	1.278285	− 0.16052	0.872474	0.975159
hsa-miR-589-5p/hsa-mir-589	417.6939	455.0682	380.3196	− 0.25511	1.174669	− 0.21718	0.828071	0.975159
hsa-miR-590-3p/hsa-mir-590	8973.993	9609.955	8338.03	− 0.20468	1.112756	− 0.18394	0.854061	0.975159
hsa-miR-590-5p/hsa-mir-590	988.4661	880.7731	1096.159	0.316749	1.138481	0.278221	0.780843	0.975159
hsa-miR-616-3p/hsa-mir-616	7.559463	8.915323	6.203603	− 0.58282	1.894644	− 0.30762	0.758375	0.975159
hsa-miR-616-5p/hsa-mir-616	8.48404	10.35951	6.60857	− 0.56703	2.16995	− 0.26131	0.793854	0.975159
hsa-miR-619-5p/hsa-mir-619	8.77423	6.950416	10.59804	0.531181	1.844996	0.287904	0.773421	0.975159
hsa-miR-624-3p/hsa-mir-624	7.71797	6.547669	8.88827	0.40851	1.801733	0.226732	0.820632	0.975159
hsa-miR-624-5p/hsa-mir-624	51.92518	58.68155	45.16881	− 0.36465	1.220922	− 0.29867	0.765194	0.975159
hsa-miR-627-3p/hsa-mir-627	23.72702	21.38349	26.07055	0.317015	1.427769	0.222035	0.824286	0.975159
hsa-miR-627-5p/hsa-mir-627	111.2786	100.6634	121.8938	0.283695	1.251422	0.226699	0.820658	0.975159
hsa-miR-628-3p/hsa-mir-628	304.4418	347.5614	261.3221	− 0.4107	1.094032	− 0.3754	0.707364	0.975159
hsa-miR-641/hsa-mir-641	48.25218	54.98768	41.51667	− 0.43718	1.337214	− 0.32693	0.74372	0.975159
hsa-miR-6513-3p/hsa-mir-6513	29.63292	26.38053	32.88532	0.3314	1.311898	0.252611	0.800569	0.975159
hsa-miR-651-5p/hsa-mir-651	674.4088	592.8	756.0176	0.352441	1.210621	0.291125	0.770956	0.975159
hsa-miR-660-5p/hsa-mir-660	7577.031	7169.711	7984.352	0.15515	1.089068	0.142461	0.886716	0.975159
hsa-miR-664b-5p/hsa-mir-664b	10.98617	9.424519	12.54783	0.430785	1.687614	0.255263	0.79852	0.975159
hsa-miR-6716-3p/hsa-mir-6716	6.394974	7.767434	5.022514	− 0.5072	2.137254	− 0.23731	0.812413	0.975159
hsa-miR-766-3p/hsa-mir-766	14.59653	11.63963	17.55344	0.567422	1.538192	0.368889	0.71221	0.975159
hsa-miR-769-5p/hsa-mir-769	2775.12	2502.892	3047.348	0.284004	1.108151	0.256286	0.79773	0.975159
hsa-miR-7705/hsa-mir-7705	15.78664	17.19195	14.38132	− 0.32867	1.584776	− 0.20739	0.835704	0.975159
hsa-miR-92a-1-5p/hsa-mir-92a-1	33.57925	29.50758	37.65092	0.326646	1.364986	0.239303	0.81087	0.975159
hsa-miR-92a-3p/hsa-mir-92a-1	31,445.38	28,988.91	33,901.85	0.225834	1.176556	0.191945	0.847786	0.975159
hsa-miR-92a-3p/hsa-mir-92a-2	29,885.12	27,476.13	32,294.1	0.23306	1.173848	0.198544	0.84262	0.975159
hsa-miR-935/hsa-mir-935	199.862	234.4854	165.2386	− 0.49737	1.439266	− 0.34557	0.729666	0.975159
hsa-miR-93-5p/hsa-mir-93	115,786.1	122,566.8	109,005.3	− 0.16916	1.086056	− 0.15575	0.876228	0.975159
hsa-miR-941/hsa-mir-941–1	955.4885	842.49	1068.487	0.343489	1.083642	0.316976	0.751262	0.975159
hsa-miR-941/hsa-mir-941–2	955.4885	842.49	1068.487	0.343489	1.083642	0.316976	0.751262	0.975159
hsa-miR-941/hsa-mir-941–3	955.4885	842.49	1068.487	0.343489	1.083642	0.316976	0.751262	0.975159
hsa-miR-941/hsa-mir-941–4	955.4885	842.49	1068.487	0.343489	1.083642	0.316976	0.751262	0.975159
hsa-miR-941/hsa-mir-941–5	955.4885	842.49	1068.487	0.343489	1.083642	0.316976	0.751262	0.975159
hsa-miR-95-5p/hsa-mir-95	8.642709	6.464269	10.82115	0.685804	2.096343	0.327143	0.74356	0.975159
hsa-miR-9903/hsa-mir-9903	87.34132	94.31706	80.36558	− 0.24653	1.262231	− 0.19531	0.845148	0.975159
hsa-miR-99a-5p/hsa-mir-99a	3709.971	3402.896	4017.046	0.239097	1.372714	0.174178	0.861725	0.975159
hsa-miR-99b-5p/hsa-mir-99b	15,308.95	14,280.54	16,337.37	0.194081	1.081978	0.179376	0.857642	0.975159
hsa-let-7d-3p/hsa-let-7d	340.5948	322.2245	358.9652	0.152921	1.148178	0.133186	0.894046	0.975624
hsa-miR-29b-2-5p/hsa-mir-29b-2	222.3509	209.7262	234.9756	0.159132	1.192747	0.133416	0.893864	0.975624
hsa-miR-3613-3p/hsa-mir-3613	19.75245	18.12694	21.37795	0.19311	1.49445	0.129218	0.897185	0.976641
hsa-miR-454-3p/hsa-mir-454	31,454.68	32,985.33	29,924.02	− 0.1405	1.076408	− 0.13053	0.896147	0.976641
hsa-miR-1277-5p/hsa-mir-1277	1933.736	2019.329	1848.142	− 0.12719	1.158569	− 0.10978	0.912583	0.978542
hsa-miR-1287-5p/hsa-mir-1287	718.351	689.7904	746.9116	0.115769	1.100965	0.105152	0.916255	0.978542
hsa-miR-151a-3p/hsa-mir-151a	41,943.11	40,215.7	43,670.52	0.118949	1.194015	0.099621	0.920645	0.978542
hsa-miR-192-5p/hsa-mir-192	5630.589	5431.044	5830.133	0.10207	1.139738	0.089556	0.92864	0.978542
hsa-miR-196b-3p/hsa-mir-196b	7.765469	8.311203	7.219735	− 0.19614	2.166496	− 0.09053	0.927862	0.978542
hsa-miR-20a-3p/hsa-mir-20a	444.6156	424.1504	465.0809	0.131714	1.106579	0.119028	0.905253	0.978542
hsa-miR-2355-3p/hsa-mir-2355	123.0826	130.1282	116.037	− 0.15929	1.388076	− 0.11476	0.908637	0.978542
hsa-miR-23a-3p/hsa-mir-23a	122,159.1	118,004.4	126,313.7	0.098181	1.067112	0.092007	0.926693	0.978542
hsa-miR-30a-3p/hsa-mir-30a	17,159.59	17,750.19	16,568.99	− 0.09939	1.098038	− 0.09052	0.927875	0.978542
hsa-miR-30b-5p/hsa-mir-30b	9038.746	9399.166	8678.326	− 0.11494	1.160184	− 0.09907	0.921083	0.978542
hsa-miR-3143/hsa-mir-3143	34.58962	36.29392	32.88532	− 0.1235	1.401393	− 0.08813	0.929775	0.978542
hsa-miR-421/hsa-mir-421	1631.492	1695.254	1567.729	− 0.11278	1.090679	− 0.1034	0.917642	0.978542
hsa-miR-505-3p/hsa-mir-505	400.314	386.2251	414.4029	0.10522	1.186524	0.088679	0.929337	0.978542
hsa-miR-5100/hsa-mir-5100	10.57415	9.92219	11.22612	0.201864	1.648525	0.122451	0.902542	0.978542
hsa-miR-548am-5p/hsa-mir-548am	122.3616	127.7714	116.9517	− 0.13323	1.138036	− 0.11707	0.906803	0.978542
hsa-miR-548au-5p/hsa-mir-548am	122.3616	127.7714	116.9517	− 0.13323	1.138036	− 0.11707	0.906803	0.978542
hsa-miR-548av-3p/hsa-mir-548o	14.91173	14.32663	15.49684	0.133872	1.521588	0.087982	0.929891	0.978542
hsa-miR-548av-3p/hsa-mir-548o-2	14.91173	14.32663	15.49684	0.133872	1.521588	0.087982	0.929891	0.978542
hsa-miR-548c-5p/hsa-mir-548am	122.3616	127.7714	116.9517	− 0.13323	1.138036	− 0.11707	0.906803	0.978542
hsa-miR-548f-3p/hsa-mir-548f-2	15.09137	14.42155	15.76118	0.152263	1.508253	0.100953	0.919588	0.978542
hsa-miR-548f-3p/hsa-mir-548f-3	15.09137	14.42155	15.76118	0.152263	1.508253	0.100953	0.919588	0.978542
hsa-miR-548o-3p/hsa-mir-548o	14.91173	14.32663	15.49684	0.133872	1.521588	0.087982	0.929891	0.978542
hsa-miR-548o-3p/hsa-mir-548o-2	14.91173	14.32663	15.49684	0.133872	1.521588	0.087982	0.929891	0.978542
hsa-miR-548o-5p/hsa-mir-548am	122.3616	127.7714	116.9517	− 0.13323	1.138036	− 0.11707	0.906803	0.978542
hsa-miR-610/hsa-mir-610	7.509562	8.063732	6.955392	− 0.17604	1.837925	− 0.09578	0.923695	0.978542
hsa-miR-664a-5p/hsa-mir-664a	125.4202	120.7952	130.0451	0.104461	1.12771	0.092631	0.926197	0.978542
hsa-miR-93-3p/hsa-mir-93	432.5288	450.6143	414.4434	− 0.12264	1.119202	− 0.10958	0.912745	0.978542
hsa-miR-98-3p/hsa-mir-98	220.0477	230.2378	209.8577	− 0.1347	1.147548	− 0.11738	0.906558	0.978542
hsa-miR-122-5p/hsa-mir-122	315.5732	305.5742	325.5722	0.091572	1.095484	0.083591	0.933382	0.978724
hsa-miR-212-3p/hsa-mir-212	15.03908	15.90304	14.17513	− 0.13509	1.609986	− 0.08391	0.933129	0.978724
hsa-miR-652-5p/hsa-mir-652	55.14162	57.30045	52.9828	− 0.11348	1.317487	− 0.08613	0.931361	0.978724
hsa-miR-570-3p/hsa-mir-570	92.53927	96.16946	88.90909	− 0.09967	1.214014	− 0.0821	0.934566	0.978806
hsa-let-7e-3p/hsa-let-7e	169.7466	174.7557	164.7375	− 0.09242	1.170497	− 0.07895	0.937069	0.980267
hsa-miR-1277-3p/hsa-mir-1277	37.30118	38.76803	35.83433	− 0.09658	1.325253	− 0.07288	0.941905	0.981694
hsa-miR-130b-3p/hsa-mir-130b	4188.927	4070.63	4307.223	0.08183	1.141865	0.071663	0.94287	0.981694
hsa-miR-30c-2-3p/hsa-mir-30c-2	918.1615	888.1475	948.1755	0.092814	1.236216	0.075079	0.940152	0.981694
hsa-miR-584-3p/hsa-mir-584	11.71847	10.90601	12.53092	0.142532	1.959611	0.072735	0.942017	0.981694
hsa-let-7e-5p/hsa-let-7e	77,540.73	79,287.44	75,794.02	− 0.06501	1.111829	− 0.05848	0.95337	0.981817
hsa-miR-1285-5p/hsa-mir-1285–1	12.68939	12.1373	13.24147	0.095472	1.642787	0.058116	0.953656	0.981817
hsa-miR-1304-3p/hsa-mir-1304	321.2176	328.9456	313.4896	− 0.0666	1.136247	− 0.05862	0.953257	0.981817
hsa-miR-144-5p/hsa-mir-144	14.01912	14.18561	13.85264	− 0.09669	1.664952	− 0.05807	0.953692	0.981817
hsa-miR-18a-3p/hsa-mir-18a	93.11314	89.9927	96.23358	0.085464	1.294497	0.066021	0.947361	0.981817
hsa-miR-26a-1-3p/hsa-mir-26a-1	348.0065	356.3291	339.6838	− 0.06577	1.142178	− 0.05758	0.954082	0.981817
hsa-miR-27a-3p/hsa-mir-27a	119,305	122,158.4	116,451.6	− 0.06901	1.094539	− 0.06305	0.949731	0.981817
hsa-miR-5001-3p/hsa-mir-5001	12.02054	11.7576	12.28349	0.095344	1.595023	0.059776	0.952334	0.981817
hsa-miR-7977/hsa-mir-7977	140.4013	143.8698	136.9329	− 0.07838	1.236373	− 0.06339	0.949453	0.981817
hsa-miR-96-3p/hsa-mir-96	29.93643	31.03516	28.8377	− 0.08149	1.342741	− 0.06069	0.951605	0.981817
hsa-miR-5094/hsa-mir-5094	9.956766	9.827266	10.08627	0.098782	1.775264	0.055643	0.955626	0.982263
hsa-miR-216a-5p/hsa-mir-216a	12.69562	12.94279	12.44844	− 0.09115	1.735484	− 0.05252	0.958114	0.982687
hsa-miR-26b-5p/hsa-mir-26b	129,902.8	132,472.3	127,333.3	− 0.05706	1.090264	− 0.05234	0.958259	0.982687
hsa-miR-3605-5p/hsa-mir-3605	20.13105	19.53656	20.72555	0.067853	1.404357	0.048316	0.961464	0.984833
hsa-miR-1287-3p/hsa-mir-1287	6.29491	6.121874	6.467945	0.057442	1.95207	0.029426	0.976525	0.986558
hsa-miR-196b-5p/hsa-mir-196b	8171.852	8014.398	8329.305	0.05569	1.66052	0.033538	0.973246	0.986558
hsa-miR-219b-5p/hsa-mir-219b	25.10071	24.84141	25.36	0.053482	1.361158	0.039292	0.968658	0.986558
hsa-miR-30d-5p/hsa-mir-30d	30,513.05	30,910.74	30,115.35	− 0.03758	1.078708	− 0.03484	0.972211	0.986558
hsa-miR-3157-5p/hsa-mir-3157	12.6517	12.49122	12.81217	0.062364	1.607633	0.038793	0.969056	0.986558
hsa-miR-455-3p/hsa-mir-455	627.1832	638.7289	615.6375	− 0.05098	1.516256	− 0.03362	0.973178	0.986558
hsa-miR-548ad-5p/hsa-mir-548ae-2	158.8643	156.6952	161.0334	0.048487	1.277887	0.037943	0.969733	0.986558
hsa-miR-548ae-5p/hsa-mir-548ae-2	158.8643	156.6952	161.0334	0.048487	1.277887	0.037943	0.969733	0.986558
hsa-miR-574-5p/hsa-mir-574	1685.776	1665.627	1705.924	0.03433	1.075891	0.031908	0.974545	0.986558
hsa-miR-584-5p/hsa-mir-584	5503.856	5578.278	5429.433	− 0.03912	1.280682	− 0.03055	0.975631	0.986558
hsa-miR-7–1-3p/hsa-mir-7–1	499.0899	506.0196	492.1602	− 0.04076	1.079578	− 0.03776	0.969881	0.986558
hsa-miR-942-5p/hsa-mir-942	736.7844	745.7182	727.8505	− 0.03525	1.092484	− 0.03227	0.974257	0.986558
hsa-miR-202-5p/hsa-mir-202	8.849494	8.975675	8.723313	0.050891	2.032876	0.025034	0.980028	0.987508
hsa-miR-219a-1-3p/hsa-mir-219a-1	30.70571	31.21076	30.20065	− 0.03386	1.322775	− 0.0256	0.97958	0.987508
hsa-miR-597-3p/hsa-mir-597	21.43839	21.05262	21.82416	0.033362	1.387187	0.02405	0.980813	0.987508
hsa-miR-10527-5p/hsa-mir-10527	12.09597	12.25527	11.93667	0.033806	1.695803	0.019935	0.984095	0.989335
hsa-miR-502-5p/hsa-mir-502	32.70491	32.19458	33.21523	0.0224	1.308407	0.01712	0.986341	0.989335
hsa-miR-6772-3p/hsa-mir-6772	6.628454	6.524621	6.732288	0.036241	1.969391	0.018402	0.985318	0.989335
hsa-miR-99a-3p/hsa-mir-99a	171.1553	172.228	170.0826	− 0.02321	1.435677	− 0.01617	0.987099	0.989335
hsa-miR-219a-5p/hsa-mir-219a-2	12.92161	13.07229	12.77093	0.023363	1.612827	0.014485	0.988443	0.989561
hsa-miR-4521/hsa-mir-4521	3198.979	3200.78	3197.178	− 0.00138	1.080429	− 0.00128	0.998982	0.998982
hsa-miR-10226/hsa-mir-10226	0	0	0	NA	NA	NA	NA	NA
hsa-miR-10392-3p/hsa-mir-10392	0	0	0	NA	NA	NA	NA	NA
hsa-miR-10392-5p/hsa-mir-10392	0.254598	0.509196	0	− 1.11434	4.959133	− 0.2247	0.82221	NA
hsa-miR-10393-3p/hsa-mir-10393	0.817018	1.634036	0	− 2.88326	4.83881	− 0.59586	0.551267	NA
hsa-miR-10393-5p/hsa-mir-10393	0	0	0	NA	NA	NA	NA	NA
hsa-miR-10394-3p/hsa-mir-10394	0	0	0	NA	NA	NA	NA	NA
hsa-miR-10394-5p/hsa-mir-10394	1.362953	0	2.725907	4.163156	4.684664	0.888677	0.374176	NA
hsa-miR-10395-3p/hsa-mir-10395	3.013109	0.615645	5.410574	2.992938	2.850764	1.049872	0.293777	NA
hsa-miR-10395-5p/hsa-mir-10395	0	0	0	NA	NA	NA	NA	NA
hsa-miR-10396a-3p/hsa-mir-10396a	0	0	0	NA	NA	NA	NA	NA
hsa-miR-10396a-5p/hsa-mir-10396a	0	0	0	NA	NA	NA	NA	NA
hsa-miR-10396a-5p/hsa-mir-10396b	0	0	0	NA	NA	NA	NA	NA
hsa-miR-10396b-3p/hsa-mir-10396b	0	0	0	NA	NA	NA	NA	NA
hsa-miR-10396b-5p/hsa-mir-10396b	0	0	0	NA	NA	NA	NA	NA
hsa-miR-10397-3p/hsa-mir-10397	0	0	0	NA	NA	NA	NA	NA
hsa-miR-10397-5p/hsa-mir-10397	0.132171	0	0.264343	1.823505	4.944331	0.368807	0.712271	NA
hsa-miR-10398-3p/hsa-mir-10398	0	0	0	NA	NA	NA	NA	NA
hsa-miR-10398-5p/hsa-mir-10398	0	0	0	NA	NA	NA	NA	NA
hsa-miR-103a-1-5p/hsa-mir-103a-1	5.033915	4.392913	5.674917	0.313698	2.084777	0.150471	0.880393	NA
hsa-miR-103b/hsa-mir-103b-1	0	0	0	NA	NA	NA	NA	NA
hsa-miR-103b/hsa-mir-103b-2	0	0	0	NA	NA	NA	NA	NA
hsa-miR-10400-3p/hsa-mir-10400	0	0	0	NA	NA	NA	NA	NA
hsa-miR-10400-5p/hsa-mir-10400	2.04443	0	4.08886	4.753613	4.067207	1.168766	0.242498	NA
hsa-miR-10401-5p/hsa-mir-10401	0.681477	0	1.362953	3.012055	4.910469	0.613395	0.539615	NA
hsa-miR-10522-5p/hsa-mir-10522	0.307822	0.615645	0	− 1.51932	4.922146	− 0.30867	0.757573	NA
hsa-miR-10524-5p/hsa-mir-10524	0	0	0	NA	NA	NA	NA	NA
hsa-miR-10525-3p/hsa-mir-10525	0	0	0	NA	NA	NA	NA	NA
hsa-miR-10526-3p/hsa-mir-10526	1.951603	3.374521	0.528686	− 2.11008	3.229899	− 0.6533	0.513566	NA
hsa-miR-11181-3p/hsa-mir-11181	0	0	0	NA	NA	NA	NA	NA
hsa-miR-11181-5p/hsa-mir-11181	0	0	0	NA	NA	NA	NA	NA
hsa-miR-11399/hsa-mir-11399	0.518941	0.509196	0.528686	0.681945	4.885862	0.139575	0.888996	NA
hsa-miR-11400/hsa-mir-11400	0	0	0	NA	NA	NA	NA	NA
hsa-miR-11401/hsa-mir-11401	0.694592	1.12484	0.264343	− 1.12785	4.807996	− 0.23458	0.814537	NA
hsa-miR-1178-3p/hsa-mir-1178	0	0	0	NA	NA	NA	NA	NA
hsa-miR-1178-5p/hsa-mir-1178	0	0	0	NA	NA	NA	NA	NA
hsa-miR-1180-5p/hsa-mir-1180	0.509196	1.018392	0	− 2.1782	4.872426	− 0.44705	0.654841	NA
hsa-miR-1181/hsa-mir-1181	0	0	0	NA	NA	NA	NA	NA
hsa-miR-1182/hsa-mir-1182	0	0	0	NA	NA	NA	NA	NA
hsa-miR-1183/hsa-mir-1183	0	0	0	NA	NA	NA	NA	NA
hsa-miR-1184/hsa-mir-1184–1	0	0	0	NA	NA	NA	NA	NA
hsa-miR-1184/hsa-mir-1184–2	0	0	0	NA	NA	NA	NA	NA
hsa-miR-1184/hsa-mir-1184–3	0	0	0	NA	NA	NA	NA	NA
hsa-miR-1185–1-3p/hsa-mir-1185–1	2.312133	0.923467	3.700799	2.025593	3.379806	0.599322	0.548958	NA
hsa-miR-1185–2-3p/hsa-mir-1185–2	0.153911	0.307822	0	− 0.74599	4.97963	− 0.14981	0.880917	NA
hsa-miR-1185-5p/hsa-mir-1185–1	0.572165	0.615645	0.528686	0.276985	4.848317	0.05713	0.954442	NA
hsa-miR-1185-5p/hsa-mir-1185–2	0.572165	0.615645	0.528686	0.276985	4.848317	0.05713	0.954442	NA
hsa-miR-1193/hsa-mir-1193	0	0	0	NA	NA	NA	NA	NA
hsa-miR-1197/hsa-mir-1197	4.251225	0.307822	8.194627	4.506394	2.956619	1.524171	0.127466	NA
hsa-miR-1199-3p/hsa-mir-1199	0	0	0	NA	NA	NA	NA	NA
hsa-miR-1199-5p/hsa-mir-1199	0	0	0	NA	NA	NA	NA	NA
hsa-miR-1200/hsa-mir-1200	0	0	0	NA	NA	NA	NA	NA
hsa-miR-1202/hsa-mir-1202	0	0	0	NA	NA	NA	NA	NA
hsa-miR-1203/hsa-mir-1203	0	0	0	NA	NA	NA	NA	NA
hsa-miR-1204/hsa-mir-1204	0	0	0	NA	NA	NA	NA	NA
hsa-miR-1205/hsa-mir-1205	0	0	0	NA	NA	NA	NA	NA
hsa-miR-1206/hsa-mir-1206	0	0	0	NA	NA	NA	NA	NA
hsa-miR-1207-3p/hsa-mir-1207	0	0	0	NA	NA	NA	NA	NA
hsa-miR-1207-5p/hsa-mir-1207	0	0	0	NA	NA	NA	NA	NA
hsa-miR-1208/hsa-mir-1208	0	0	0	NA	NA	NA	NA	NA
hsa-miR-12113/hsa-mir-12113	0	0	0	NA	NA	NA	NA	NA
hsa-miR-12114/hsa-mir-12114	1.012159	1.231289	0.793028	− 0.33182	4.610832	− 0.07196	0.94263	NA
hsa-miR-12115/hsa-mir-12115	0	0	0	NA	NA	NA	NA	NA
hsa-miR-12116/hsa-mir-12116	0	0	0	NA	NA	NA	NA	NA
hsa-miR-12117/hsa-mir-12117	0	0	0	NA	NA	NA	NA	NA
hsa-miR-12118/hsa-mir-12118	0	0	0	NA	NA	NA	NA	NA
hsa-miR-12119/hsa-mir-12119	0	0	0	NA	NA	NA	NA	NA
hsa-miR-12120/hsa-mir-12120	0	0	0	NA	NA	NA	NA	NA
hsa-miR-12121/hsa-mir-12121	0	0	0	NA	NA	NA	NA	NA
hsa-miR-12122/hsa-mir-12122	0	0	0	NA	NA	NA	NA	NA
hsa-miR-12123/hsa-mir-12123	0.286083	0.307822	0.264343	0.477194	4.926281	0.096867	0.922832	NA
hsa-miR-12124/hsa-mir-12124	0	0	0	NA	NA	NA	NA	NA
hsa-miR-12125/hsa-mir-12125	0	0	0	NA	NA	NA	NA	NA
hsa-miR-12126/hsa-mir-12126	0	0	0	NA	NA	NA	NA	NA
hsa-miR-12127/hsa-mir-12127	0	0	0	NA	NA	NA	NA	NA
hsa-miR-12128/hsa-mir-12128	0	0	0	NA	NA	NA	NA	NA
hsa-miR-12129/hsa-mir-12129	0.254598	0.509196	0	− 1.11434	4.959133	− 0.2247	0.82221	NA
hsa-miR-12130/hsa-mir-12130	0	0	0	NA	NA	NA	NA	NA
hsa-miR-12131/hsa-mir-12131	0	0	0	NA	NA	NA	NA	NA
hsa-miR-12132/hsa-mir-12132	0	0	0	NA	NA	NA	NA	NA
hsa-miR-12133/hsa-mir-12133	0.54068	0.817018	0.264343	− 0.65628	4.837961	− 0.13565	0.892097	NA
hsa-miR-122-3p/hsa-mir-122	0	0	0	NA	NA	NA	NA	NA
hsa-miR-1224-3p/hsa-mir-1224	0.254598	0.509196	0	− 1.11434	4.959133	− 0.2247	0.82221	NA
hsa-miR-1224-5p/hsa-mir-1224	0	0	0	NA	NA	NA	NA	NA
hsa-miR-1225-3p/hsa-mir-1225	0	0	0	NA	NA	NA	NA	NA
hsa-miR-1225-5p/hsa-mir-1225	0	0	0	NA	NA	NA	NA	NA
hsa-miR-1226-5p/hsa-mir-1226	0	0	0	NA	NA	NA	NA	NA
hsa-miR-1227-3p/hsa-mir-1227	0.264343	0	0.528686	2.396631	4.924788	0.486646	0.626509	NA
hsa-miR-1227-5p/hsa-mir-1227	0	0	0	NA	NA	NA	NA	NA
hsa-miR-1228-3p/hsa-mir-1228	0.396514	0	0.793028	2.767752	4.915475	0.563069	0.573388	NA
hsa-miR-1228-5p/hsa-mir-1228	0	0	0	NA	NA	NA	NA	NA
hsa-miR-1229-3p/hsa-mir-1229	1.517985	0.615645	2.420325	1.893906	3.605705	0.525253	0.599408	NA
hsa-miR-1229-5p/hsa-mir-1229	0	0	0	NA	NA	NA	NA	NA
hsa-miR-122b-3p/hsa-mir-122b	0	0	0	NA	NA	NA	NA	NA
hsa-miR-122b-5p/hsa-mir-122b	3.74133	7.48266	0	− 5.09098	2.985271	− 1.70537	0.088126	NA
hsa-miR-1231/hsa-mir-1231	0.681477	0	1.362953	3.012055	4.910469	0.613395	0.539615	NA
hsa-miR-1233-3p/hsa-mir-1233–1	0	0	0	NA	NA	NA	NA	NA
hsa-miR-1233-3p/hsa-mir-1233–2	0	0	0	NA	NA	NA	NA	NA
hsa-miR-1233-5p/hsa-mir-1233–1	0	0	0	NA	NA	NA	NA	NA
hsa-miR-1233-5p/hsa-mir-1233–2	0	0	0	NA	NA	NA	NA	NA
hsa-miR-1234-3p/hsa-mir-1234	0.264343	0	0.528686	2.396631	4.924788	0.486646	0.626509	NA
hsa-miR-1236-3p/hsa-mir-1236	0.94694	0.307822	1.586057	2.184228	4.790294	0.45597	0.648412	NA
hsa-miR-1236-5p/hsa-mir-1236	3.91657	0.307822	7.525317	4.318439	2.712883	1.591826	0.111424	NA
hsa-miR-1237-3p/hsa-mir-1237	0.763794	1.527587	0	− 2.77189	4.84311	− 0.57234	0.567094	NA
hsa-miR-1237-5p/hsa-mir-1237	0	0	0	NA	NA	NA	NA	NA
hsa-miR-1238-3p/hsa-mir-1238	0.132171	0	0.264343	1.823505	4.944331	0.368807	0.712271	NA
hsa-miR-1238-5p/hsa-mir-1238	0	0	0	NA	NA	NA	NA	NA
hsa-miR-1243/hsa-mir-1243	3.214848	6.429696	0	− 4.85837	3.06281	− 1.58624	0.112684	NA
hsa-miR-124-3p/hsa-mir-124–1	0	0	0	NA	NA	NA	NA	NA
hsa-miR-124-3p/hsa-mir-124–2	0	0	0	NA	NA	NA	NA	NA
hsa-miR-124-3p/hsa-mir-124–3	0	0	0	NA	NA	NA	NA	NA
hsa-miR-1244/hsa-mir-1244–1	0.264343	0	0.528686	2.396631	4.924788	0.486646	0.626509	NA
hsa-miR-1244/hsa-mir-1244–2	0.264343	0	0.528686	2.396631	4.924788	0.486646	0.626509	NA
hsa-miR-1244/hsa-mir-1244–3	0.264343	0	0.528686	2.396631	4.924788	0.486646	0.626509	NA
hsa-miR-1244/hsa-mir-1244–4	0.264343	0	0.528686	2.396631	4.924788	0.486646	0.626509	NA
hsa-miR-1245a/hsa-mir-1245a	0.153911	0.307822	0	− 0.74599	4.97963	− 0.14981	0.880917	NA
hsa-miR-1245b-3p/hsa-mir-1245b	0	0	0	NA	NA	NA	NA	NA
hsa-miR-1245b-5p/hsa-mir-1245b	0.153911	0.307822	0	− 0.74599	4.97963	− 0.14981	0.880917	NA
hsa-miR-124-5p/hsa-mir-124–1	0	0	0	NA	NA	NA	NA	NA
hsa-miR-124-5p/hsa-mir-124–2	0	0	0	NA	NA	NA	NA	NA
hsa-miR-124-5p/hsa-mir-124–3	0	0	0	NA	NA	NA	NA	NA
hsa-miR-1247-3p/hsa-mir-1247	2.114743	0	4.229485	4.913958	3.913804	1.255545	0.209281	NA
hsa-miR-1247-5p/hsa-mir-1247	1.321714	0	2.643428	4.240271	4.639562	0.913938	0.36075	NA
hsa-miR-1249-5p/hsa-mir-1249	1.033899	1.539112	0.528686	− 1.02168	4.581774	− 0.22299	0.823545	NA
hsa-miR-1250-3p/hsa-mir-1250	0	0	0	NA	NA	NA	NA	NA
hsa-miR-1250-5p/hsa-mir-1250	1.688994	1.527587	1.8504	0.412862	3.804911	0.108508	0.913593	NA
hsa-miR-1251-3p/hsa-mir-1251	0	0	0	NA	NA	NA	NA	NA
hsa-miR-1251-5p/hsa-mir-1251	0	0	0	NA	NA	NA	NA	NA
hsa-miR-1252-3p/hsa-mir-1252	0.54068	0.817018	0.264343	− 0.65628	4.837961	− 0.13565	0.892097	NA
hsa-miR-1253/hsa-mir-1253	0	0	0	NA	NA	NA	NA	NA
hsa-miR-1255b-2-3p/hsa-mir-1255b-2	0	0	0	NA	NA	NA	NA	NA
hsa-miR-1256/hsa-mir-1256	0.132171	0	0.264343	1.823505	4.944331	0.368807	0.712271	NA
hsa-miR-1258/hsa-mir-1258	0.773539	1.018392	0.528686	− 0.3819	4.797831	− 0.0796	0.936556	NA
hsa-miR-1261/hsa-mir-1261	0	0	0	NA	NA	NA	NA	NA
hsa-miR-1263/hsa-mir-1263	0.132171	0	0.264343	1.823505	4.944331	0.368807	0.712271	NA
hsa-miR-1264/hsa-mir-1264	0.307822	0.615645	0	− 1.51932	4.922146	− 0.30867	0.757573	NA
hsa-miR-1265/hsa-mir-1265	0.509196	1.018392	0	− 2.1782	4.872426	− 0.44705	0.654841	NA
hsa-miR-1266-3p/hsa-mir-1266	0	0	0	NA	NA	NA	NA	NA
hsa-miR-1271-3p/hsa-mir-1271	0.793028	0	1.586057	3.52828	4.884751	0.722305	0.470107	NA
hsa-miR-1272/hsa-mir-1272	4.555687	0	9.111374	5.973397	2.778382	2.149955	0.031559	NA
hsa-miR-1273 h-3p/hsa-mir-1273 h	0	0	0	NA	NA	NA	NA	NA
hsa-miR-1273 h-5p/hsa-mir-1273 h	0.132171	0	0.264343	1.823505	4.944331	0.368807	0.712271	NA
hsa-miR-127-5p/hsa-mir-127	0	0	0	NA	NA	NA	NA	NA
hsa-miR-1279/hsa-mir-1279	0	0	0	NA	NA	NA	NA	NA
hsa-miR-1281/hsa-mir-1281	0	0	0	NA	NA	NA	NA	NA
hsa-miR-128–2-5p/hsa-mir-128–2	0	0	0	NA	NA	NA	NA	NA
hsa-miR-1282/hsa-mir-1282	0	0	0	NA	NA	NA	NA	NA
hsa-miR-1288-3p/hsa-mir-1288	1.48762	1.12484	1.8504	0.835112	3.585059	0.232942	0.815806	NA
hsa-miR-1288-5p/hsa-mir-1288	0	0	0	NA	NA	NA	NA	NA
hsa-miR-1289/hsa-mir-1289–1	4.657868	6.631069	2.684667	− 1.29682	2.206569	− 0.58771	0.556728	NA
hsa-miR-1289/hsa-mir-1289–2	4.657868	6.631069	2.684667	− 1.29682	2.206569	− 0.58771	0.556728	NA
hsa-miR-129–1-3p/hsa-mir-129–1	1.178065	2.35613	0	− 3.42376	4.366695	− 0.78406	0.433004	NA
hsa-miR-1291/hsa-mir-1291	5.511991	11.02398	0	− 5.63734	2.641501	− 2.13414	0.032831	NA
hsa-miR-129–2-3p/hsa-mir-129–2	0.641367	1.018392	0.264343	− 0.95503	4.817889	− 0.19822	0.842869	NA
hsa-miR-1292-3p/hsa-mir-1292	2.308773	0	4.617546	4.930853	3.488822	1.413329	0.157559	NA
hsa-miR-1295a/hsa-mir-1295a	4.638673	2.462579	6.814767	1.374498	2.839446	0.484073	0.628334	NA
hsa-miR-1295b-3p/hsa-mir-1295b	0	0	0	NA	NA	NA	NA	NA
hsa-miR-1295b-5p/hsa-mir-1295b	0	0	0	NA	NA	NA	NA	NA
hsa-miR-1296-3p/hsa-mir-1296	0.396514	0	0.793028	2.767752	4.915475	0.563069	0.573388	NA
hsa-miR-1297/hsa-mir-1297	0	0	0	NA	NA	NA	NA	NA
hsa-miR-1298-3p/hsa-mir-1298	0.461733	0.923467	0	− 2.09228	4.877731	− 0.42895	0.667962	NA
hsa-miR-1298-5p/hsa-mir-1298	4.925157	9.850314	0	− 5.48703	3.124677	− 1.75603	0.079083	NA
hsa-miR-1301-5p/hsa-mir-1301	0	0	0	NA	NA	NA	NA	NA
hsa-miR-1302/hsa-mir-1302–1	2.11301	1.846934	2.379085	0.439862	3.472539	0.126669	0.899203	NA
hsa-miR-1302/hsa-mir-1302–10	2.11301	1.846934	2.379085	0.439862	3.472539	0.126669	0.899203	NA
hsa-miR-1302/hsa-mir-1302–11	2.11301	1.846934	2.379085	0.439862	3.472539	0.126669	0.899203	NA
hsa-miR-1302/hsa-mir-1302–2	2.11301	1.846934	2.379085	0.439862	3.472539	0.126669	0.899203	NA
hsa-miR-1302/hsa-mir-1302–3	2.11301	1.846934	2.379085	0.439862	3.472539	0.126669	0.899203	NA
hsa-miR-1302/hsa-mir-1302–4	1.980838	1.846934	2.114743	0.277189	3.555509	0.07796	0.937859	NA
hsa-miR-1302/hsa-mir-1302–5	1.980838	1.846934	2.114743	0.277189	3.555509	0.07796	0.937859	NA
hsa-miR-1302/hsa-mir-1302–6	2.11301	1.846934	2.379085	0.439862	3.472539	0.126669	0.899203	NA
hsa-miR-1302/hsa-mir-1302–7	2.11301	1.846934	2.379085	0.439862	3.472539	0.126669	0.899203	NA
hsa-miR-1302/hsa-mir-1302–8	2.11301	1.846934	2.379085	0.439862	3.472539	0.126669	0.899203	NA
hsa-miR-1302/hsa-mir-1302–9	2.11301	1.846934	2.379085	0.439862	3.472539	0.126669	0.899203	NA
hsa-miR-1304-5p/hsa-mir-1304	3.022712	3.055175	2.99025	− 0.11968	2.94532	− 0.04063	0.967587	NA
hsa-miR-1321/hsa-mir-1321	0	0	0	NA	NA	NA	NA	NA
hsa-miR-1324/hsa-mir-1324	0	0	0	NA	NA	NA	NA	NA
hsa-miR-133a-3p/hsa-mir-133a-1	5.140871	7.068389	3.213353	− 1.11852	2.134313	− 0.52407	0.600233	NA
hsa-miR-133a-3p/hsa-mir-133a-2	5.140871	7.068389	3.213353	− 1.11852	2.134313	− 0.52407	0.600233	NA
hsa-miR-133a-5p/hsa-mir-133a-1	0.132171	0	0.264343	1.823505	4.944331	0.368807	0.712271	NA
hsa-miR-133a-5p/hsa-mir-133a-2	0.132171	0	0.264343	1.823505	4.944331	0.368807	0.712271	NA
hsa-miR-133b/hsa-mir-133b	0.153911	0.307822	0	− 0.74599	4.97963	− 0.14981	0.880917	NA
hsa-miR-1343-5p/hsa-mir-1343	1.729103	0.509196	2.94901	2.694291	3.513585	0.766821	0.443188	NA
hsa-miR-134-3p/hsa-mir-134	0	0	0	NA	NA	NA	NA	NA
hsa-miR-135a-2-3p/hsa-mir-135a-2	0.153911	0.307822	0	− 0.74599	4.97963	− 0.14981	0.880917	NA
hsa-miR-135a-3p/hsa-mir-135a-1	0	0	0	NA	NA	NA	NA	NA
hsa-miR-136-3p/hsa-mir-136	2.089537	3.386046	0.793028	− 1.7536	3.549551	− 0.49403	0.621283	NA
hsa-miR-136-5p/hsa-mir-136	0	0	0	NA	NA	NA	NA	NA
hsa-miR-138–2-3p/hsa-mir-138–2	2.859198	0.307822	5.410574	3.809679	2.991934	1.273317	0.202906	NA
hsa-miR-139-3p/hsa-mir-139	4.653886	7.15179	2.155982	− 1.80378	2.213444	− 0.81492	0.415119	NA
hsa-miR-144-3p/hsa-mir-144	0.264343	0	0.528686	2.396631	4.924788	0.486646	0.626509	NA
hsa-miR-1468-3p/hsa-mir-1468	0.153911	0.307822	0	− 0.74599	4.97963	− 0.14981	0.880917	NA
hsa-miR-1469/hsa-mir-1469	0	0	0	NA	NA	NA	NA	NA
hsa-miR-1470/hsa-mir-1470	0	0	0	NA	NA	NA	NA	NA
hsa-miR-1471/hsa-mir-1471	0	0	0	NA	NA	NA	NA	NA
hsa-miR-147a/hsa-mir-147a	2.789854	2.853801	2.725907	− 0.19474	3.01767	− 0.06453	0.948547	NA
hsa-miR-149-3p/hsa-mir-149	1.321714	0	2.643428	4.240271	4.639562	0.913938	0.36075	NA
hsa-miR-150-3p/hsa-mir-150	0	0	0	NA	NA	NA	NA	NA
hsa-miR-150-5p/hsa-mir-150	3.21386	2.462579	3.965142	0.743906	3.016706	0.246595	0.805221	NA
hsa-miR-153-3p/hsa-mir-153–1	4.303704	6.228323	2.379085	− 1.20293	2.416185	− 0.49786	0.618581	NA
hsa-miR-153-3p/hsa-mir-153–2	4.303704	6.228323	2.379085	− 1.20293	2.416185	− 0.49786	0.618581	NA
hsa-miR-153-5p/hsa-mir-153–2	0	0	0	NA	NA	NA	NA	NA
hsa-miR-1537-3p/hsa-mir-1537	6.00071	9.093648	2.907771	− 1.46171	2.169939	− 0.67362	0.500555	NA
hsa-miR-1537-5p/hsa-mir-1537	3.970159	5.825576	2.114743	− 1.27116	2.473109	− 0.51399	0.607258	NA
hsa-miR-1538/hsa-mir-1538	0.945819	0	1.891639	3.662379	4.791539	0.764343	0.444663	NA
hsa-miR-1539/hsa-mir-1539	0	0	0	NA	NA	NA	NA	NA
hsa-miR-154-3p/hsa-mir-154	0.396514	0	0.793028	2.767752	4.915475	0.563069	0.573388	NA
hsa-miR-154-5p/hsa-mir-154	2.3104	2.770401	1.8504	− 0.47217	3.381045	− 0.13965	0.888935	NA
hsa-miR-1587/hsa-mir-1587	0	0	0	NA	NA	NA	NA	NA
hsa-miR-1-5p/hsa-mir-1–1	0.461733	0.923467	0	− 2.09228	4.877731	− 0.42895	0.667962	NA
hsa-miR-1825/hsa-mir-1825	0	0	0	NA	NA	NA	NA	NA
hsa-miR-1827/hsa-mir-1827	0.254598	0.509196	0	− 1.11434	4.959133	− 0.2247	0.82221	NA
hsa-miR-187-5p/hsa-mir-187	1.362953	0	2.725907	4.163156	4.684664	0.888677	0.374176	NA
hsa-miR-18b-3p/hsa-mir-18b	0	0	0	NA	NA	NA	NA	NA
hsa-miR-1908-3p/hsa-mir-1908	1.432663	2.865326	0	− 3.70192	4.026369	− 0.91942	0.357876	NA
hsa-miR-1908-5p/hsa-mir-1908	2.571231	4.085091	1.057371	− 1.67725	2.91883	− 0.57463	0.565542	NA
hsa-miR-1909-3p/hsa-mir-1909	0.132171	0	0.264343	1.823505	4.944331	0.368807	0.712271	NA
hsa-miR-1909-5p/hsa-mir-1909	1.362953	0	2.725907	4.163156	4.684664	0.888677	0.374176	NA
hsa-miR-190a-3p/hsa-mir-190a	6.030086	4.617335	7.442838	0.714222	2.312673	0.30883	0.757451	NA
hsa-miR-190b-3p/hsa-mir-190b	0.418254	0.307822	0.528686	1.050318	4.906666	0.214059	0.830501	NA
hsa-miR-1910-3p/hsa-mir-1910	1.057371	0	2.114743	3.925076	4.861599	0.807363	0.419457	NA
hsa-miR-1911-3p/hsa-mir-1911	0	0	0	NA	NA	NA	NA	NA
hsa-miR-1911-5p/hsa-mir-1911	0.461733	0.923467	0	− 2.09228	4.877731	− 0.42895	0.667962	NA
hsa-miR-1912-3p/hsa-mir-1912	0	0	0	NA	NA	NA	NA	NA
hsa-miR-1912-5p/hsa-mir-1912	0	0	0	NA	NA	NA	NA	NA
hsa-miR-1913/hsa-mir-1913	0	0	0	NA	NA	NA	NA	NA
hsa-miR-1914-3p/hsa-mir-1914	0.528686	0	1.057371	3.044078	4.909871	0.619992	0.535263	NA
hsa-miR-1914-5p/hsa-mir-1914	2.110769	1.231289	2.99025	1.140734	3.24778	0.351235	0.725412	NA
hsa-miR-1915-3p/hsa-mir-1915	0.681477	0	1.362953	3.012055	4.910469	0.613395	0.539615	NA
hsa-miR-1915-5p/hsa-mir-1915	2.02381	0	4.047621	4.770558	3.585675	1.330449	0.18337	NA
hsa-miR-196a-1-3p/hsa-mir-196a-1	2.907771	0	5.815542	5.369694	3.532176	1.520223	0.128455	NA
hsa-miR-1972/hsa-mir-1972–1	0.694592	1.12484	0.264343	− 1.12785	4.807996	− 0.23458	0.814537	NA
hsa-miR-1972/hsa-mir-1972–2	0.694592	1.12484	0.264343	− 1.12785	4.807996	− 0.23458	0.814537	NA
hsa-miR-1973/hsa-mir-1973	2.984382	4.605811	1.362953	− 2.28725	2.913707	− 0.785	0.432455	NA
hsa-miR-197-5p/hsa-mir-197	1.781207	0.307822	3.254592	3.059855	3.506857	0.872535	0.382917	NA
hsa-miR-1976/hsa-mir-1976	0	0	0	NA	NA	NA	NA	NA
hsa-miR-198/hsa-mir-198	0	0	0	NA	NA	NA	NA	NA
hsa-miR-19a-5p/hsa-mir-19a	3.108399	6.216798	0	− 4.80547	3.169073	− 1.51637	0.129427	NA
hsa-miR-19b-2-5p/hsa-mir-19b-2	1.362953	0	2.725907	4.163156	4.684664	0.888677	0.374176	NA
hsa-miR-202-3p/hsa-mir-202	0	0	0	NA	NA	NA	NA	NA
hsa-miR-203a-5p/hsa-mir-203a	0.814768	0.307822	1.321714	1.93264	4.884354	0.39568	0.692341	NA
hsa-miR-203b-5p/hsa-mir-203b	0	0	0	NA	NA	NA	NA	NA
hsa-miR-204-3p/hsa-mir-204	0	0	0	NA	NA	NA	NA	NA
hsa-miR-2052/hsa-mir-2052	0	0	0	NA	NA	NA	NA	NA
hsa-miR-2053/hsa-mir-2053	0	0	0	NA	NA	NA	NA	NA
hsa-miR-2054/hsa-mir-2054	0	0	0	NA	NA	NA	NA	NA
hsa-miR-206/hsa-mir-206	0.681477	0	1.362953	3.012055	4.910469	0.613395	0.539615	NA
hsa-miR-208a-3p/hsa-mir-208a	0	0	0	NA	NA	NA	NA	NA
hsa-miR-208a-5p/hsa-mir-208a	0	0	0	NA	NA	NA	NA	NA
hsa-miR-208b-3p/hsa-mir-208b	0	0	0	NA	NA	NA	NA	NA
hsa-miR-208b-5p/hsa-mir-208b	0	0	0	NA	NA	NA	NA	NA
hsa-miR-20b-3p/hsa-mir-20b	2.04443	0	4.08886	4.753613	4.067207	1.168766	0.242498	NA
hsa-miR-210-5p/hsa-mir-210	1.272989	2.545979	0	− 3.51573	4.761337	− 0.73839	0.460277	NA
hsa-miR-2113/hsa-mir-2113	0.550425	0.307822	0.793028	1.42144	4.897318	0.290249	0.771626	NA
hsa-miR-211-3p/hsa-mir-211	0	0	0	NA	NA	NA	NA	NA
hsa-miR-2114-3p/hsa-mir-2114	1.172303	2.344605	0	− 3.39745	4.400056	− 0.77214	0.440032	NA
hsa-miR-2114-5p/hsa-mir-2114	2.971774	5.943549	0	− 4.7585	3.174364	− 1.49904	0.133863	NA
hsa-miR-2115-3p/hsa-mir-2115	1.670776	0.615645	2.725907	1.999765	3.932211	0.50856	0.611061	NA
hsa-miR-2115-5p/hsa-mir-2115	0.681477	0	1.362953	3.012055	4.910469	0.613395	0.539615	NA
hsa-miR-211-5p/hsa-mir-211	0.307822	0.615645	0	− 1.51932	4.922146	− 0.30867	0.757573	NA
hsa-miR-2117/hsa-mir-2117	0	0	0	NA	NA	NA	NA	NA
hsa-miR-215-3p/hsa-mir-215	1.331976	2.663952	0	− 3.60145	4.174871	− 0.86265	0.388331	NA
hsa-miR-216a-3p/hsa-mir-216a	2.831084	2.143232	3.518935	0.652542	2.60074	0.250906	0.801887	NA
hsa-miR-216b-3p/hsa-mir-216b	0	0	0	NA	NA	NA	NA	NA
hsa-miR-216b-5p/hsa-mir-216b	0	0	0	NA	NA	NA	NA	NA
hsa-miR-217-3p/hsa-mir-217	0	0	0	NA	NA	NA	NA	NA
hsa-miR-218–1-3p/hsa-mir-218–1	1.077378	2.154756	0	− 3.29934	4.82535	− 0.68375	0.494131	NA
hsa-miR-218–2-3p/hsa-mir-218–2	0	0	0	NA	NA	NA	NA	NA
hsa-miR-219a-2-3p/hsa-mir-219a-2	0	0	0	NA	NA	NA	NA	NA
hsa-miR-223-5p/hsa-mir-223	0.813648	0	1.627296	3.418215	4.892272	0.698697	0.484741	NA
hsa-miR-2276-3p/hsa-mir-2276	5.32576	4.712259	5.93926	0.267689	2.082157	0.128563	0.897703	NA
hsa-miR-2276-5p/hsa-mir-2276	0	0	0	NA	NA	NA	NA	NA
hsa-miR-2392/hsa-mir-2392	0	0	0	NA	NA	NA	NA	NA
hsa-miR-2467-3p/hsa-mir-2467	0	0	0	NA	NA	NA	NA	NA
hsa-miR-2467-5p/hsa-mir-2467	0.826763	1.12484	0.528686	− 0.5515	4.504759	− 0.12243	0.902562	NA
hsa-miR-2681-3p/hsa-mir-2681	1.018392	2.036783	0	− 3.19138	4.828483	− 0.66095	0.508645	NA
hsa-miR-2681-5p/hsa-mir-2681	0	0	0	NA	NA	NA	NA	NA
hsa-miR-2861/hsa-mir-2861	0	0	0	NA	NA	NA	NA	NA
hsa-miR-2909/hsa-mir-2909	0	0	0	NA	NA	NA	NA	NA
hsa-miR-297/hsa-mir-297	0	0	0	NA	NA	NA	NA	NA
hsa-miR-298/hsa-mir-298	0.307822	0.615645	0	− 1.51932	4.922146	− 0.30867	0.757573	NA
hsa-miR-299-3p/hsa-mir-299	5.436794	1.539112	9.334477	2.56451	2.308561	1.11087	0.266624	NA
hsa-miR-299-5p/hsa-mir-299	2.553003	2.462579	2.643428	0.182091	3.255723	0.055929	0.955398	NA
hsa-miR-300/hsa-mir-300	0	0	0	NA	NA	NA	NA	NA
hsa-miR-302a-3p/hsa-mir-302a	0	0	0	NA	NA	NA	NA	NA
hsa-miR-302a-5p/hsa-mir-302a	0	0	0	NA	NA	NA	NA	NA
hsa-miR-302b-3p/hsa-mir-302b	0	0	0	NA	NA	NA	NA	NA
hsa-miR-302b-5p/hsa-mir-302b	0	0	0	NA	NA	NA	NA	NA
hsa-miR-302c-3p/hsa-mir-302c	0	0	0	NA	NA	NA	NA	NA
hsa-miR-302c-5p/hsa-mir-302c	0	0	0	NA	NA	NA	NA	NA
hsa-miR-302d-3p/hsa-mir-302d	0	0	0	NA	NA	NA	NA	NA
hsa-miR-302d-5p/hsa-mir-302d	0	0	0	NA	NA	NA	NA	NA
hsa-miR-302e/hsa-mir-302e	0	0	0	NA	NA	NA	NA	NA
hsa-miR-302f/hsa-mir-302f	0	0	0	NA	NA	NA	NA	NA
hsa-miR-3059-3p/hsa-mir-3059	1.057371	0	2.114743	3.925076	4.861599	0.807363	0.419457	NA
hsa-miR-3064-3p/hsa-mir-3064	1.302224	1.018392	1.586057	0.778749	4.201547	0.185348	0.852956	NA
hsa-miR-3074-3p/hsa-mir-3074	2.923146	3.161624	2.684667	− 0.21975	2.573185	− 0.0854	0.931942	NA
hsa-miR-3074-5p/hsa-mir-3074	1.722728	2.652428	0.793028	− 1.36746	3.396669	− 0.40259	0.687251	NA
hsa-miR-3085-3p/hsa-mir-3085	0	0	0	NA	NA	NA	NA	NA
hsa-miR-3085-5p/hsa-mir-3085	0	0	0	NA	NA	NA	NA	NA
hsa-miR-3116/hsa-mir-3116–1	4.11172	0.615645	7.607795	3.45909	2.657819	1.301477	0.193095	NA
hsa-miR-3116/hsa-mir-3116–2	4.11172	0.615645	7.607795	3.45909	2.657819	1.301477	0.193095	NA
hsa-miR-3117-3p/hsa-mir-3117	0	0	0	NA	NA	NA	NA	NA
hsa-miR-3117-5p/hsa-mir-3117	0	0	0	NA	NA	NA	NA	NA
hsa-miR-3118/hsa-mir-3118–1	0	0	0	NA	NA	NA	NA	NA
hsa-miR-3118/hsa-mir-3118–2	0	0	0	NA	NA	NA	NA	NA
hsa-miR-3118/hsa-mir-3118–3	0	0	0	NA	NA	NA	NA	NA
hsa-miR-3118/hsa-mir-3118–4	0	0	0	NA	NA	NA	NA	NA
hsa-miR-3119/hsa-mir-3119–1	0	0	0	NA	NA	NA	NA	NA
hsa-miR-3119/hsa-mir-3119–2	0	0	0	NA	NA	NA	NA	NA
hsa-miR-3120-3p/hsa-mir-3120	0.663107	1.326214	0	− 2.57515	4.851549	− 0.53079	0.595564	NA
hsa-miR-3120-5p/hsa-mir-3120	0	0	0	NA	NA	NA	NA	NA
hsa-miR-3121-3p/hsa-mir-3121	0.901727	1.539112	0.264343	− 1.59554	4.786369	− 0.33335	0.738869	NA
hsa-miR-3121-5p/hsa-mir-3121	0	0	0	NA	NA	NA	NA	NA
hsa-miR-3122/hsa-mir-3122	0	0	0	NA	NA	NA	NA	NA
hsa-miR-3123/hsa-mir-3123	0	0	0	NA	NA	NA	NA	NA
hsa-miR-3124-3p/hsa-mir-3124	0.681477	0	1.362953	3.012055	4.910469	0.613395	0.539615	NA
hsa-miR-3124-5p/hsa-mir-3124	1.345704	1.634036	1.057371	− 0.40128	3.710507	− 0.10815	0.913879	NA
hsa-miR-3126-3p/hsa-mir-3126	3.189738	4.487837	1.891639	− 1.34927	2.559314	− 0.5272	0.598054	NA
hsa-miR-3126-5p/hsa-mir-3126	0.153911	0.307822	0	− 0.74599	4.97963	− 0.14981	0.880917	NA
hsa-miR-3127-3p/hsa-mir-3127	0.418254	0.307822	0.528686	1.050318	4.906666	0.214059	0.830501	NA
hsa-miR-3130-3p/hsa-mir-3130–1	0	0	0	NA	NA	NA	NA	NA
hsa-miR-3130-3p/hsa-mir-3130–2	0	0	0	NA	NA	NA	NA	NA
hsa-miR-3130-5p/hsa-mir-3130–1	5.960245	5.411304	6.509185	0.1868	2.023563	0.092312	0.92645	NA
hsa-miR-3131/hsa-mir-3131	0.286083	0.307822	0.264343	0.477194	4.926281	0.096867	0.922832	NA
hsa-miR-3132/hsa-mir-3132	0.386769	0.509196	0.264343	0.108828	4.90556	0.022185	0.982301	NA
hsa-miR-3133/hsa-mir-3133	5.191338	3.386046	6.996631	1.000755	2.33796	0.428046	0.668617	NA
hsa-miR-3134/hsa-mir-3134	0.132171	0	0.264343	1.823505	4.944331	0.368807	0.712271	NA
hsa-miR-3135a/hsa-mir-3135a	0	0	0	NA	NA	NA	NA	NA
hsa-miR-3135b/hsa-mir-3135b	0	0	0	NA	NA	NA	NA	NA
hsa-miR-3136-3p/hsa-mir-3136	0.980674	1.432663	0.528686	− 0.90089	4.207238	− 0.21413	0.830447	NA
hsa-miR-3137/hsa-mir-3137	0	0	0	NA	NA	NA	NA	NA
hsa-miR-3140-5p/hsa-mir-3140	2.040295	2.758877	1.321714	− 0.8702	3.150058	− 0.27625	0.782358	NA
hsa-miR-3141/hsa-mir-3141	0.264343	0	0.528686	2.396631	4.924788	0.486646	0.626509	NA
hsa-miR-3142/hsa-mir-3142	0	0	0	NA	NA	NA	NA	NA
hsa-miR-3145-5p/hsa-mir-3145	0.153911	0.307822	0	− 0.74599	4.97963	− 0.14981	0.880917	NA
hsa-miR-3147/hsa-mir-3147	0	0	0	NA	NA	NA	NA	NA
hsa-miR-3148/hsa-mir-3148	3.656727	2.96025	4.353203	0.448791	2.469093	0.181764	0.855768	NA
hsa-miR-3150a-3p/hsa-mir-3150a	2.475799	1.432663	3.518935	1.206494	2.745431	0.439455	0.660332	NA
hsa-miR-3150a-5p/hsa-mir-3150a	1.081361	1.634036	0.528686	− 1.07481	4.039233	− 0.26609	0.790168	NA
hsa-miR-3150b-3p/hsa-mir-3150b	4.353203	0	8.706406	5.849767	2.943946	1.987049	0.046917	NA
hsa-miR-3150b-5p/hsa-mir-3150b	0	0	0	NA	NA	NA	NA	NA
hsa-miR-3151-3p/hsa-mir-3151	0	0	0	NA	NA	NA	NA	NA
hsa-miR-3151-5p/hsa-mir-3151	0	0	0	NA	NA	NA	NA	NA
hsa-miR-3152-3p/hsa-mir-3152	2.800577	5.601154	0	− 4.65522	3.657934	− 1.27264	0.203147	NA
hsa-miR-3152-5p/hsa-mir-3152	0	0	0	NA	NA	NA	NA	NA
hsa-miR-3153/hsa-mir-3153	0	0	0	NA	NA	NA	NA	NA
hsa-miR-3154/hsa-mir-3154	0	0	0	NA	NA	NA	NA	NA
hsa-miR-3155a/hsa-mir-3155a	0	0	0	NA	NA	NA	NA	NA
hsa-miR-3155b/hsa-mir-3155a	0	0	0	NA	NA	NA	NA	NA
hsa-miR-3155b/hsa-mir-3155b	0	0	0	NA	NA	NA	NA	NA
hsa-miR-3156-3p/hsa-mir-3156–1	0	0	0	NA	NA	NA	NA	NA
hsa-miR-3156-3p/hsa-mir-3156–2	0	0	0	NA	NA	NA	NA	NA
hsa-miR-3156-5p/hsa-mir-3156–1	0	0	0	NA	NA	NA	NA	NA
hsa-miR-3156-5p/hsa-mir-3156–2	0	0	0	NA	NA	NA	NA	NA
hsa-miR-3156-5p/hsa-mir-3156–3	0	0	0	NA	NA	NA	NA	NA
hsa-miR-3157-3p/hsa-mir-3157	0.726076	0.923467	0.528686	− 0.29599	4.803219	− 0.06162	0.950864	NA
hsa-miR-3158-3p/hsa-mir-3158–1	1.627804	2.462579	0.793028	− 1.30728	3.862996	− 0.33841	0.735054	NA
hsa-miR-3158-3p/hsa-mir-3158–2	1.627804	2.462579	0.793028	− 1.30728	3.862996	− 0.33841	0.735054	NA
hsa-miR-3158-5p/hsa-mir-3158–1	1.210162	0	2.420325	4.044592	4.335033	0.933001	0.350819	NA
hsa-miR-3158-5p/hsa-mir-3158–2	0.681477	0	1.362953	3.012055	4.910469	0.613395	0.539615	NA
hsa-miR-3159/hsa-mir-3159	4.997308	3.386046	6.60857	1.005957	2.661093	0.378024	0.705413	NA
hsa-miR-3160-3p/hsa-mir-3160–1	1.781715	2.770401	0.793028	− 1.4725	3.744246	− 0.39327	0.69412	NA
hsa-miR-3160-3p/hsa-mir-3160–2	1.781715	2.770401	0.793028	− 1.4725	3.744246	− 0.39327	0.69412	NA
hsa-miR-3160-5p/hsa-mir-3160–1	2.179971	1.634036	2.725907	0.615976	3.219218	0.191343	0.848257	NA
hsa-miR-3160-5p/hsa-mir-3160–2	2.179971	1.634036	2.725907	0.615976	3.219218	0.191343	0.848257	NA
hsa-miR-3161/hsa-mir-3161	3.122421	0	6.244842	5.371593	3.165487	1.696924	0.089711	NA
hsa-miR-3162-3p/hsa-mir-3162	0	0	0	NA	NA	NA	NA	NA
hsa-miR-3162-5p/hsa-mir-3162	0.615645	1.231289	0	− 2.50085	4.855042	− 0.5151	0.606481	NA
hsa-miR-3163/hsa-mir-3163	2.629097	3.895241	1.362953	− 2.00189	3.09356	− 0.64711	0.517558	NA
hsa-miR-3165/hsa-mir-3165	2.215946	3.374521	1.057371	− 1.42006	3.070806	− 0.46244	0.643766	NA
hsa-miR-3166/hsa-mir-3166	0.264343	0	0.528686	2.396631	4.924788	0.486646	0.626509	NA
hsa-miR-3167/hsa-mir-3167	0	0	0	NA	NA	NA	NA	NA
hsa-miR-3168/hsa-mir-3168	0	0	0	NA	NA	NA	NA	NA
hsa-miR-3169/hsa-mir-3169	0	0	0	NA	NA	NA	NA	NA
hsa-miR-3170/hsa-mir-3170	0.254598	0.509196	0	− 1.11434	4.959133	− 0.2247	0.82221	NA
hsa-miR-3173-3p/hsa-mir-3173	0.518941	0.509196	0.528686	0.681945	4.885862	0.139575	0.888996	NA
hsa-miR-3175/hsa-mir-3175	0.681477	0	1.362953	3.012055	4.910469	0.613395	0.539615	NA
hsa-miR-3177-3p/hsa-mir-3177	0.651112	0.509196	0.793028	1.053061	4.876474	0.215947	0.829029	NA
hsa-miR-3177-5p/hsa-mir-3177	2.831074	1.432663	4.229485	1.636489	2.859283	0.572342	0.56709	NA
hsa-miR-3178/hsa-mir-3178	0	0	0	NA	NA	NA	NA	NA
hsa-miR-3179/hsa-mir-3179–1	3.421513	6.049998	0.793028	− 2.54615	2.77928	− 0.91612	0.359605	NA
hsa-miR-3179/hsa-mir-3179–2	3.421513	6.049998	0.793028	− 2.54615	2.77928	− 0.91612	0.359605	NA
hsa-miR-3179/hsa-mir-3179–3	3.421513	6.049998	0.793028	− 2.54615	2.77928	− 0.91612	0.359605	NA
hsa-miR-3179/hsa-mir-3179–4	3.421513	6.049998	0.793028	− 2.54615	2.77928	− 0.91612	0.359605	NA
hsa-miR-3180-3p/hsa-mir-3180–1	2.879818	0.307822	5.451813	3.804913	3.391493	1.121899	0.261905	NA
hsa-miR-3180-3p/hsa-mir-3180–2	2.879818	0.307822	5.451813	3.804913	3.391493	1.121899	0.261905	NA
hsa-miR-3180-3p/hsa-mir-3180–3	2.879818	0.307822	5.451813	3.804913	3.391493	1.121899	0.261905	NA
hsa-miR-3180-3p/hsa-mir-3180–4	2.879818	0.307822	5.451813	3.804913	3.391493	1.121899	0.261905	NA
hsa-miR-3180-3p/hsa-mir-3180–5	2.879818	0.307822	5.451813	3.804913	3.391493	1.121899	0.261905	NA
hsa-miR-3180-5p/hsa-mir-3180–1	0.461733	0.923467	0	− 2.09228	4.877731	− 0.42895	0.667962	NA
hsa-miR-3180-5p/hsa-mir-3180–2	0.461733	0.923467	0	− 2.09228	4.877731	− 0.42895	0.667962	NA
hsa-miR-3180-5p/hsa-mir-3180–3	0.461733	0.923467	0	− 2.09228	4.877731	− 0.42895	0.667962	NA
hsa-miR-3180/hsa-mir-3180–1	2.879818	0.307822	5.451813	3.804913	3.391493	1.121899	0.261905	NA
hsa-miR-3180/hsa-mir-3180–2	2.879818	0.307822	5.451813	3.804913	3.391493	1.121899	0.261905	NA
hsa-miR-3180/hsa-mir-3180–3	2.879818	0.307822	5.451813	3.804913	3.391493	1.121899	0.261905	NA
hsa-miR-3180/hsa-mir-3180–4	2.879818	0.307822	5.451813	3.804913	3.391493	1.121899	0.261905	NA
hsa-miR-3180/hsa-mir-3180–5	2.879818	0.307822	5.451813	3.804913	3.391493	1.121899	0.261905	NA
hsa-miR-3181/hsa-mir-3181	0	0	0	NA	NA	NA	NA	NA
hsa-miR-3182/hsa-mir-3182	0	0	0	NA	NA	NA	NA	NA
hsa-miR-3183/hsa-mir-3183	1.458385	2.652428	0.264343	− 2.35188	3.64371	− 0.64546	0.518627	NA
hsa-miR-3184-3p/hsa-mir-3184	0	0	0	NA	NA	NA	NA	NA
hsa-miR-3184-5p/hsa-mir-3184	0	0	0	NA	NA	NA	NA	NA
hsa-miR-3185/hsa-mir-3185	0	0	0	NA	NA	NA	NA	NA
hsa-miR-3186-3p/hsa-mir-3186	0	0	0	NA	NA	NA	NA	NA
hsa-miR-3186-5p/hsa-mir-3186	0	0	0	NA	NA	NA	NA	NA
hsa-miR-3187-5p/hsa-mir-3187	2.884308	2.249681	3.518935	0.558307	2.564503	0.217706	0.827658	NA
hsa-miR-3188/hsa-mir-3188	1.903634	0.817018	2.99025	1.783155	3.149039	0.566254	0.571221	NA
hsa-miR-3189-3p/hsa-mir-3189	0.386769	0.509196	0.264343	0.108828	4.90556	0.022185	0.982301	NA
hsa-miR-3189-5p/hsa-mir-3189	0.307822	0.615645	0	− 1.51932	4.922146	− 0.30867	0.757573	NA
hsa-miR-3190-3p/hsa-mir-3190	0.528686	0	1.057371	3.044078	4.909871	0.619992	0.535263	NA
hsa-miR-3190-5p/hsa-mir-3190	0.254598	0.509196	0	− 1.11434	4.959133	− 0.2247	0.82221	NA
hsa-miR-3191-3p/hsa-mir-3191	0.153911	0.307822	0	− 0.74599	4.97963	− 0.14981	0.880917	NA
hsa-miR-3191-5p/hsa-mir-3191	0	0	0	NA	NA	NA	NA	NA
hsa-miR-3192-3p/hsa-mir-3192	0.264343	0	0.528686	2.396631	4.924788	0.486646	0.626509	NA
hsa-miR-3192-5p/hsa-mir-3192	5.767486	3.48097	8.054002	1.191108	1.998999	0.595852	0.551274	NA
hsa-miR-3193/hsa-mir-3193	1.047626	0.509196	1.586057	1.829656	4.617886	0.396211	0.69195	NA
hsa-miR-3194-3p/hsa-mir-3194	0.509196	1.018392	0	− 2.1782	4.872426	− 0.44705	0.654841	NA
hsa-miR-3194-5p/hsa-mir-3194	4.815914	4.180015	5.451813	0.298407	2.550415	0.117003	0.906857	NA
hsa-miR-3195/hsa-mir-3195	0	0	0	NA	NA	NA	NA	NA
hsa-miR-3196/hsa-mir-3196	0	0	0	NA	NA	NA	NA	NA
hsa-miR-3197/hsa-mir-3197	0	0	0	NA	NA	NA	NA	NA
hsa-miR-3198/hsa-mir-3198–1	2.365367	1.740485	2.99025	0.639749	2.859377	0.223737	0.822962	NA
hsa-miR-3198/hsa-mir-3198–2	2.365367	1.740485	2.99025	0.639749	2.859377	0.223737	0.822962	NA
hsa-miR-3199/hsa-mir-3199–1	4.894239	5.517753	4.270724	− 0.31088	2.111095	− 0.14726	0.882928	NA
hsa-miR-3199/hsa-mir-3199–2	4.894239	5.517753	4.270724	− 0.31088	2.111095	− 0.14726	0.882928	NA
hsa-miR-3201/hsa-mir-3201	0.286083	0.307822	0.264343	0.477194	4.926281	0.096867	0.922832	NA
hsa-miR-3202/hsa-mir-3202–1	0.153911	0.307822	0	− 0.74599	4.97963	− 0.14981	0.880917	NA
hsa-miR-3202/hsa-mir-3202–2	0.153911	0.307822	0	− 0.74599	4.97963	− 0.14981	0.880917	NA
hsa-miR-320a-5p/hsa-mir-320a	3.182893	5.8371	0.528686	− 2.88919	2.765924	− 1.04457	0.296223	NA
hsa-miR-323a-5p/hsa-mir-323a	0	0	0	NA	NA	NA	NA	NA
hsa-miR-323b-3p/hsa-mir-323b	3.083422	0.615645	5.551199	3.152625	3.123134	1.009443	0.312762	NA
hsa-miR-323b-5p/hsa-mir-323b	0	0	0	NA	NA	NA	NA	NA
hsa-miR-325/hsa-mir-325	0	0	0	NA	NA	NA	NA	NA
hsa-miR-328-5p/hsa-mir-328	0	0	0	NA	NA	NA	NA	NA
hsa-miR-329-3p/hsa-mir-329–1	2.707527	0.615645	4.79941	2.907408	2.955286	0.983799	0.325214	NA
hsa-miR-329-3p/hsa-mir-329–2	2.707527	0.615645	4.79941	2.907408	2.955286	0.983799	0.325214	NA
hsa-miR-329-5p/hsa-mir-329–1	0	0	0	NA	NA	NA	NA	NA
hsa-miR-329-5p/hsa-mir-329–2	0	0	0	NA	NA	NA	NA	NA
hsa-miR-337-3p/hsa-mir-337	0	0	0	NA	NA	NA	NA	NA
hsa-miR-337-5p/hsa-mir-337	0.769556	1.539112	0	− 2.81872	4.841262	− 0.58223	0.560413	NA
hsa-miR-338-5p/hsa-mir-338	0	0	0	NA	NA	NA	NA	NA
hsa-miR-346/hsa-mir-346	0	0	0	NA	NA	NA	NA	NA
hsa-miR-3529-3p/hsa-mir-3529	0	0	0	NA	NA	NA	NA	NA
hsa-miR-3529-5p/hsa-mir-3529	0.408509	0.817018	0	− 1.87945	4.892274	− 0.38417	0.700854	NA
hsa-miR-3606-3p/hsa-mir-3606	0	0	0	NA	NA	NA	NA	NA
hsa-miR-3606-5p/hsa-mir-3606	0	0	0	NA	NA	NA	NA	NA
hsa-miR-3609/hsa-mir-3609	3.061079	3.173148	2.94901	− 0.10428	2.497329	− 0.04176	0.966694	NA
hsa-miR-3610/hsa-mir-3610	0	0	0	NA	NA	NA	NA	NA
hsa-miR-3612/hsa-mir-3612	0	0	0	NA	NA	NA	NA	NA
hsa-miR-3616-5p/hsa-mir-3616	0.153911	0.307822	0	− 0.74599	4.97963	− 0.14981	0.880917	NA
hsa-miR-3617-3p/hsa-mir-3617	5.898253	9.0706	2.725907	− 1.95525	2.439825	− 0.80139	0.422906	NA
hsa-miR-3618/hsa-mir-3618	0.386769	0.509196	0.264343	0.108828	4.90556	0.022185	0.982301	NA
hsa-miR-3619-3p/hsa-mir-3619	0.408509	0.817018	0	− 1.87945	4.892274	− 0.38417	0.700854	NA
hsa-miR-3619-5p/hsa-mir-3619	0.509196	1.018392	0	− 2.1782	4.872426	− 0.44705	0.654841	NA
hsa-miR-3620-3p/hsa-mir-3620	0.132171	0	0.264343	1.823505	4.944331	0.368807	0.712271	NA
hsa-miR-3620-5p/hsa-mir-3620	0.836508	0.615645	1.057371	0.924429	4.833162	0.191268	0.848316	NA
hsa-miR-3621/hsa-mir-3621	0	0	0	NA	NA	NA	NA	NA
hsa-miR-3622a-3p/hsa-mir-3622a	2.330513	0.307822	4.353203	3.466641	3.285262	1.05521	0.291329	NA
hsa-miR-3622a-5p/hsa-mir-3622a	0	0	0	NA	NA	NA	NA	NA
hsa-miR-3622b-3p/hsa-mir-3622b	0	0	0	NA	NA	NA	NA	NA
hsa-miR-3622b-5p/hsa-mir-3622b	0	0	0	NA	NA	NA	NA	NA
hsa-miR-363-5p/hsa-mir-363	4.242771	0.307822	8.17772	4.396743	3.081531	1.426805	0.153636	NA
hsa-miR-3646/hsa-mir-3646	0	0	0	NA	NA	NA	NA	NA
hsa-miR-3648/hsa-mir-3648–1	1.282734	2.036783	0.528686	− 1.38477	4.25402	− 0.32552	0.744787	NA
hsa-miR-3648/hsa-mir-3648–2	1.282734	2.036783	0.528686	− 1.38477	4.25402	− 0.32552	0.744787	NA
hsa-miR-3649/hsa-mir-3649	0	0	0	NA	NA	NA	NA	NA
hsa-miR-3650/hsa-mir-3650	0	0	0	NA	NA	NA	NA	NA
hsa-miR-3651/hsa-mir-3651	1.150563	2.036783	0.264343	− 1.96576	4.453029	− 0.44144	0.658893	NA
hsa-miR-3652/hsa-mir-3652	0.813648	0	1.627296	3.418215	4.892272	0.698697	0.484741	NA
hsa-miR-3654/hsa-mir-3654	0	0	0	NA	NA	NA	NA	NA
hsa-miR-3655/hsa-mir-3655	0	0	0	NA	NA	NA	NA	NA
hsa-miR-3658/hsa-mir-3658	0	0	0	NA	NA	NA	NA	NA
hsa-miR-3659/hsa-mir-3659	0	0	0	NA	NA	NA	NA	NA
hsa-miR-3660/hsa-mir-3660	0.132171	0	0.264343	1.823505	4.944331	0.368807	0.712271	NA
hsa-miR-3663-3p/hsa-mir-3663	0.264343	0	0.528686	2.396631	4.924788	0.486646	0.626509	NA
hsa-miR-3663-5p/hsa-mir-3663	0.528686	0	1.057371	3.044078	4.909871	0.619992	0.535263	NA
hsa-miR-3664-5p/hsa-mir-3664	0.396514	0	0.793028	2.767752	4.915475	0.563069	0.573388	NA
hsa-miR-3665/hsa-mir-3665	0	0	0	NA	NA	NA	NA	NA
hsa-miR-3666/hsa-mir-3666	0	0	0	NA	NA	NA	NA	NA
hsa-miR-3667-3p/hsa-mir-3667	1.475625	0.307822	2.643428	2.90711	4.059297	0.716161	0.473892	NA
hsa-miR-3668/hsa-mir-3668	0	0	0	NA	NA	NA	NA	NA
hsa-miR-3670/hsa-mir-3670–1	0	0	0	NA	NA	NA	NA	NA
hsa-miR-3670/hsa-mir-3670–2	0	0	0	NA	NA	NA	NA	NA
hsa-miR-3670/hsa-mir-3670–3	0	0	0	NA	NA	NA	NA	NA
hsa-miR-3670/hsa-mir-3670–4	0	0	0	NA	NA	NA	NA	NA
hsa-miR-3671/hsa-mir-3671	0	0	0	NA	NA	NA	NA	NA
hsa-miR-3672/hsa-mir-3672	0.153911	0.307822	0	− 0.74599	4.97963	− 0.14981	0.880917	NA
hsa-miR-367-3p/hsa-mir-367	0	0	0	NA	NA	NA	NA	NA
hsa-miR-3674/hsa-mir-3674	0	0	0	NA	NA	NA	NA	NA
hsa-miR-3675-3p/hsa-mir-3675	0.528686	0	1.057371	3.044078	4.909871	0.619992	0.535263	NA
hsa-miR-3675-5p/hsa-mir-3675	1.233022	0.615645	1.8504	1.628845	4.277544	0.38079	0.703359	NA
hsa-miR-367-5p/hsa-mir-367	0	0	0	NA	NA	NA	NA	NA
hsa-miR-3677-3p/hsa-mir-3677	0.763794	1.527587	0	− 2.77189	4.84311	− 0.57234	0.567094	NA
hsa-miR-3677-5p/hsa-mir-3677	1.516865	0.307822	2.725907	2.792606	4.127289	0.67662	0.498647	NA
hsa-miR-3678-3p/hsa-mir-3678	0.528686	0	1.057371	3.044078	4.909871	0.619992	0.535263	NA
hsa-miR-3678-5p/hsa-mir-3678	1.516865	0.307822	2.725907	2.792606	4.127289	0.67662	0.498647	NA
hsa-miR-3679-3p/hsa-mir-3679	1.179798	0.509196	1.8504	2.055306	4.413446	0.465692	0.641436	NA
hsa-miR-3680-3p/hsa-mir-3680–1	1.841172	3.682344	0	− 4.06205	3.667407	− 1.10761	0.268031	NA
hsa-miR-3680-3p/hsa-mir-3680–2	1.841172	3.682344	0	− 4.06205	3.667407	− 1.10761	0.268031	NA
hsa-miR-3680-5p/hsa-mir-3680–1	2.355613	1.539112	3.172114	1.094885	3.341007	0.327711	0.74313	NA
hsa-miR-3680-5p/hsa-mir-3680–2	2.355613	1.539112	3.172114	1.094885	3.341007	0.327711	0.74313	NA
hsa-miR-3681-3p/hsa-mir-3681	3.321297	6.642594	0	− 4.90984	3.015522	− 1.62819	0.103485	NA
hsa-miR-3682-3p/hsa-mir-3682	5.465943	6.049998	4.881889	− 0.42639	2.199117	− 0.19389	0.846261	NA
hsa-miR-3682-5p/hsa-mir-3682	0.153911	0.307822	0	− 0.74599	4.97963	− 0.14981	0.880917	NA
hsa-miR-3683/hsa-mir-3683	0	0	0	NA	NA	NA	NA	NA
hsa-miR-3685/hsa-mir-3685	0	0	0	NA	NA	NA	NA	NA
hsa-miR-3686/hsa-mir-3686	0	0	0	NA	NA	NA	NA	NA
hsa-miR-3688-5p/hsa-mir-3688–1	0	0	0	NA	NA	NA	NA	NA
hsa-miR-3688-5p/hsa-mir-3688–2	0	0	0	NA	NA	NA	NA	NA
hsa-miR-3689a-3p/hsa-mir-3689a	0	0	0	NA	NA	NA	NA	NA
hsa-miR-3689a-3p/hsa-mir-3689c	0	0	0	NA	NA	NA	NA	NA
hsa-miR-3689a-5p/hsa-mir-3689a	0.660857	0	1.321714	3.278941	4.90256	0.668822	0.503609	NA
hsa-miR-3689a-5p/hsa-mir-3689b	0.660857	0	1.321714	3.278941	4.90256	0.668822	0.503609	NA
hsa-miR-3689a-5p/hsa-mir-3689e	0.660857	0	1.321714	3.278941	4.90256	0.668822	0.503609	NA
hsa-miR-3689b-3p/hsa-mir-3689b	0	0	0	NA	NA	NA	NA	NA
hsa-miR-3689b-3p/hsa-mir-3689c	0	0	0	NA	NA	NA	NA	NA
hsa-miR-3689b-5p/hsa-mir-3689a	0.660857	0	1.321714	3.278941	4.90256	0.668822	0.503609	NA
hsa-miR-3689b-5p/hsa-mir-3689b	0.660857	0	1.321714	3.278941	4.90256	0.668822	0.503609	NA
hsa-miR-3689b-5p/hsa-mir-3689e	0.660857	0	1.321714	3.278941	4.90256	0.668822	0.503609	NA
hsa-miR-3689c/hsa-mir-3689b	0	0	0	NA	NA	NA	NA	NA
hsa-miR-3689c/hsa-mir-3689c	0	0	0	NA	NA	NA	NA	NA
hsa-miR-3689d/hsa-mir-3689d-1	0	0	0	NA	NA	NA	NA	NA
hsa-miR-3689d/hsa-mir-3689d-2	0	0	0	NA	NA	NA	NA	NA
hsa-miR-3689e/hsa-mir-3689a	0.660857	0	1.321714	3.278941	4.90256	0.668822	0.503609	NA
hsa-miR-3689e/hsa-mir-3689b	0.660857	0	1.321714	3.278941	4.90256	0.668822	0.503609	NA
hsa-miR-3689e/hsa-mir-3689e	0.660857	0	1.321714	3.278941	4.90256	0.668822	0.503609	NA
hsa-miR-3689f/hsa-mir-3689f	0.528686	0	1.057371	3.044078	4.909871	0.619992	0.535263	NA
hsa-miR-3690/hsa-mir-3690–1	0.153911	0.307822	0	− 0.74599	4.97963	− 0.14981	0.880917	NA
hsa-miR-3690/hsa-mir-3690–2	0.153911	0.307822	0	− 0.74599	4.97963	− 0.14981	0.880917	NA
hsa-miR-3691-3p/hsa-mir-3691	4.842277	2.770401	6.914152	1.33127	2.429965	0.547856	0.583791	NA
hsa-miR-3692-3p/hsa-mir-3692	1.734723	3.469446	0	− 3.96706	3.762289	− 1.05443	0.291687	NA
hsa-miR-3692-5p/hsa-mir-3692	0.153911	0.307822	0	− 0.74599	4.97963	− 0.14981	0.880917	NA
hsa-miR-369-5p/hsa-mir-369	4.622533	3.693868	5.551199	0.639585	2.718615	0.235261	0.814006	NA
hsa-miR-370-3p/hsa-mir-370	0.307822	0.615645	0	− 1.51932	4.922146	− 0.30867	0.757573	NA
hsa-miR-370-5p/hsa-mir-370	0	0	0	NA	NA	NA	NA	NA
hsa-miR-3713/hsa-mir-3713	0	0	0	NA	NA	NA	NA	NA
hsa-miR-3714/hsa-mir-3714	0	0	0	NA	NA	NA	NA	NA
hsa-miR-371a-3p/hsa-mir-371a	0	0	0	NA	NA	NA	NA	NA
hsa-miR-371a-5p/hsa-mir-371a	0	0	0	NA	NA	NA	NA	NA
hsa-miR-371b-3p/hsa-mir-371b	0	0	0	NA	NA	NA	NA	NA
hsa-miR-371b-5p/hsa-mir-371b	0	0	0	NA	NA	NA	NA	NA
hsa-miR-372-3p/hsa-mir-372	0	0	0	NA	NA	NA	NA	NA
hsa-miR-372-5p/hsa-mir-372	0	0	0	NA	NA	NA	NA	NA
hsa-miR-373-3p/hsa-mir-373	0.836508	0.615645	1.057371	0.924429	4.833162	0.191268	0.848316	NA
hsa-miR-373-5p/hsa-mir-373	0.132171	0	0.264343	1.823505	4.944331	0.368807	0.712271	NA
hsa-miR-374c-3p/hsa-mir-374c	1.920119	3.575895	0.264343	− 2.79008	3.279672	− 0.85072	0.394925	NA
hsa-miR-375-5p/hsa-mir-375	0	0	0	NA	NA	NA	NA	NA
hsa-miR-376a-2-5p/hsa-mir-376a-2	0.286083	0.307822	0.264343	0.477194	4.926281	0.096867	0.922832	NA
hsa-miR-376a-5p/hsa-mir-376a-1	1.444141	0.509196	2.379085	2.424778	4.098046	0.591691	0.554057	NA
hsa-miR-376b-5p/hsa-mir-376b	4.18254	3.078223	5.286856	0.829434	2.791464	0.297132	0.766365	NA
hsa-miR-376c-5p/hsa-mir-376c	4.18254	3.078223	5.286856	0.829434	2.791464	0.297132	0.766365	NA
hsa-miR-377-3p/hsa-mir-377	1.562584	1.539112	1.586057	0.135288	3.885332	0.03482	0.972223	NA
hsa-miR-377-5p/hsa-mir-377	0	0	0	NA	NA	NA	NA	NA
hsa-miR-378b/hsa-mir-378b	1.754213	2.451054	1.057371	− 0.96896	3.3485	− 0.28937	0.772296	NA
hsa-miR-378e/hsa-mir-378e	2.794344	4.79566	0.793028	− 2.1958	2.865052	− 0.76641	0.443433	NA
hsa-miR-378 h/hsa-mir-378 h	0.945819	0	1.891639	3.662379	4.791539	0.764343	0.444663	NA
hsa-miR-378j/hsa-mir-378j	0.153911	0.307822	0	− 0.74599	4.97963	− 0.14981	0.880917	NA
hsa-miR-379-3p/hsa-mir-379	1.321714	0	2.643428	4.240271	4.639562	0.913938	0.36075	NA
hsa-miR-380-3p/hsa-mir-380	0.286083	0.307822	0.264343	0.477194	4.926281	0.096867	0.922832	NA
hsa-miR-380-5p/hsa-mir-380	1.122591	0.923467	1.321714	0.596069	4.423869	0.134739	0.892818	NA
hsa-miR-381-5p/hsa-mir-381	0	0	0	NA	NA	NA	NA	NA
hsa-miR-382-3p/hsa-mir-382	1.211282	0.307822	2.114743	2.591299	4.361129	0.594181	0.552391	NA
hsa-miR-383-3p/hsa-mir-383	0	0	0	NA	NA	NA	NA	NA
hsa-miR-383-5p/hsa-mir-383	0	0	0	NA	NA	NA	NA	NA
hsa-miR-384/hsa-mir-384	0	0	0	NA	NA	NA	NA	NA
hsa-miR-3907/hsa-mir-3907	0	0	0	NA	NA	NA	NA	NA
hsa-miR-3908/hsa-mir-3908	0	0	0	NA	NA	NA	NA	NA
hsa-miR-3910/hsa-mir-3910–1	0	0	0	NA	NA	NA	NA	NA
hsa-miR-3910/hsa-mir-3910–2	0	0	0	NA	NA	NA	NA	NA
hsa-miR-3912-5p/hsa-mir-3912	5.822818	7.862359	3.783278	− 1.14301	2.026428	− 0.56405	0.572719	NA
hsa-miR-3913-3p/hsa-mir-3913–1	6.083686	10.84566	1.321714	− 2.73824	2.2156	− 1.23589	0.2165	NA
hsa-miR-3913-3p/hsa-mir-3913–2	6.083686	10.84566	1.321714	− 2.73824	2.2156	− 1.23589	0.2165	NA
hsa-miR-3914/hsa-mir-3914–1	0	0	0	NA	NA	NA	NA	NA
hsa-miR-3914/hsa-mir-3914–2	0	0	0	NA	NA	NA	NA	NA
hsa-miR-3915/hsa-mir-3915	0	0	0	NA	NA	NA	NA	NA
hsa-miR-3916/hsa-mir-3916	3.285312	4.191539	2.379085	− 0.65941	2.634545	− 0.25029	0.80236	NA
hsa-miR-3917/hsa-mir-3917	0	0	0	NA	NA	NA	NA	NA
hsa-miR-3918/hsa-mir-3918	0	0	0	NA	NA	NA	NA	NA
hsa-miR-3919/hsa-mir-3919	0.509196	1.018392	0	− 2.1782	4.872426	− 0.44705	0.654841	NA
hsa-miR-3920/hsa-mir-3920	0	0	0	NA	NA	NA	NA	NA
hsa-miR-3921/hsa-mir-3921	0	0	0	NA	NA	NA	NA	NA
hsa-miR-3922-3p/hsa-mir-3922	0	0	0	NA	NA	NA	NA	NA
hsa-miR-3922-5p/hsa-mir-3922	0.681477	0	1.362953	3.012055	4.910469	0.613395	0.539615	NA
hsa-miR-3923/hsa-mir-3923	0	0	0	NA	NA	NA	NA	NA
hsa-miR-3924/hsa-mir-3924	0	0	0	NA	NA	NA	NA	NA
hsa-miR-3925-3p/hsa-mir-3925	0	0	0	NA	NA	NA	NA	NA
hsa-miR-3925-5p/hsa-mir-3925	0.716331	1.432663	0	− 2.70323	4.845928	− 0.55784	0.576957	NA
hsa-miR-3926/hsa-mir-3926–1	0	0	0	NA	NA	NA	NA	NA
hsa-miR-3926/hsa-mir-3926–2	0	0	0	NA	NA	NA	NA	NA
hsa-miR-3927-3p/hsa-mir-3927	1.474505	0	2.94901	4.345329	4.021035	1.08065	0.279853	NA
hsa-miR-3927-5p/hsa-mir-3927	0	0	0	NA	NA	NA	NA	NA
hsa-miR-3928-5p/hsa-mir-3928	0.681477	0	1.362953	3.012055	4.910469	0.613395	0.539615	NA
hsa-miR-3936/hsa-mir-3936	0.264343	0	0.528686	2.396631	4.924788	0.486646	0.626509	NA
hsa-miR-3937/hsa-mir-3937	0	0	0	NA	NA	NA	NA	NA
hsa-miR-3938/hsa-mir-3938	0	0	0	NA	NA	NA	NA	NA
hsa-miR-3940-5p/hsa-mir-3940	0.509196	1.018392	0	− 2.1782	4.872426	− 0.44705	0.654841	NA
hsa-miR-3941/hsa-mir-3941	0.461733	0.923467	0	− 2.09228	4.877731	− 0.42895	0.667962	NA
hsa-miR-3942-3p/hsa-mir-3942	0.528686	0	1.057371	3.044078	4.909871	0.619992	0.535263	NA
hsa-miR-3943/hsa-mir-3943	0.763794	1.527587	0	− 2.77189	4.84311	− 0.57234	0.567094	NA
hsa-miR-3944-3p/hsa-mir-3944	2.423695	1.634036	3.213353	1.008375	2.728028	0.369635	0.711654	NA
hsa-miR-3944-5p/hsa-mir-3944	3.255712	0.307822	6.203603	4.02281	2.86415	1.404539	0.160158	NA
hsa-miR-3945/hsa-mir-3945	0	0	0	NA	NA	NA	NA	NA
hsa-miR-3960/hsa-mir-3960	1.362953	0	2.725907	4.163156	4.684664	0.888677	0.374176	NA
hsa-miR-3972/hsa-mir-3972	0	0	0	NA	NA	NA	NA	NA
hsa-miR-3973/hsa-mir-3973	0	0	0	NA	NA	NA	NA	NA
hsa-miR-3974/hsa-mir-3974	0	0	0	NA	NA	NA	NA	NA
hsa-miR-3975/hsa-mir-3975	0	0	0	NA	NA	NA	NA	NA
hsa-miR-3976/hsa-mir-3976	0	0	0	NA	NA	NA	NA	NA
hsa-miR-3977/hsa-mir-3977	0	0	0	NA	NA	NA	NA	NA
hsa-miR-3978/hsa-mir-3978	0	0	0	NA	NA	NA	NA	NA
hsa-miR-409-5p/hsa-mir-409	1.386933	0.923467	1.8504	1.06221	4.066831	0.261189	0.793947	NA
hsa-miR-410-3p/hsa-mir-410	0.264343	0	0.528686	2.396631	4.924788	0.486646	0.626509	NA
hsa-miR-410-5p/hsa-mir-410	0	0	0	NA	NA	NA	NA	NA
hsa-miR-411-3p/hsa-mir-411	0.793028	0	1.586057	3.52828	4.884751	0.722305	0.470107	NA
hsa-miR-412-3p/hsa-mir-412	0.132171	0	0.264343	1.823505	4.944331	0.368807	0.712271	NA
hsa-miR-412-5p/hsa-mir-412	1.047626	0.509196	1.586057	1.829656	4.617886	0.396211	0.69195	NA
hsa-miR-4251/hsa-mir-4251	0	0	0	NA	NA	NA	NA	NA
hsa-miR-4252/hsa-mir-4252	0	0	0	NA	NA	NA	NA	NA
hsa-miR-4253/hsa-mir-4253	0	0	0	NA	NA	NA	NA	NA
hsa-miR-4254/hsa-mir-4254	0	0	0	NA	NA	NA	NA	NA
hsa-miR-4255/hsa-mir-4255	0	0	0	NA	NA	NA	NA	NA
hsa-miR-4256/hsa-mir-4256	0	0	0	NA	NA	NA	NA	NA
hsa-miR-4257/hsa-mir-4257	0	0	0	NA	NA	NA	NA	NA
hsa-miR-4258/hsa-mir-4258	0	0	0	NA	NA	NA	NA	NA
hsa-miR-4259/hsa-mir-4259	0	0	0	NA	NA	NA	NA	NA
hsa-miR-4260/hsa-mir-4260	0	0	0	NA	NA	NA	NA	NA
hsa-miR-4261/hsa-mir-4261	0	0	0	NA	NA	NA	NA	NA
hsa-miR-4262/hsa-mir-4262	0	0	0	NA	NA	NA	NA	NA
hsa-miR-4263/hsa-mir-4263	0	0	0	NA	NA	NA	NA	NA
hsa-miR-4264/hsa-mir-4264	0	0	0	NA	NA	NA	NA	NA
hsa-miR-4265/hsa-mir-4265	0	0	0	NA	NA	NA	NA	NA
hsa-miR-4266/hsa-mir-4266	0	0	0	NA	NA	NA	NA	NA
hsa-miR-4267/hsa-mir-4267	0	0	0	NA	NA	NA	NA	NA
hsa-miR-4268/hsa-mir-4268	0	0	0	NA	NA	NA	NA	NA
hsa-miR-4269/hsa-mir-4269	0	0	0	NA	NA	NA	NA	NA
hsa-miR-4270/hsa-mir-4270	0	0	0	NA	NA	NA	NA	NA
hsa-miR-4271/hsa-mir-4271	0	0	0	NA	NA	NA	NA	NA
hsa-miR-4272/hsa-mir-4272	0	0	0	NA	NA	NA	NA	NA
hsa-miR-4273/hsa-mir-4273	0	0	0	NA	NA	NA	NA	NA
hsa-miR-4274/hsa-mir-4274	0	0	0	NA	NA	NA	NA	NA
hsa-miR-4275/hsa-mir-4275	0	0	0	NA	NA	NA	NA	NA
hsa-miR-4276/hsa-mir-4276	0	0	0	NA	NA	NA	NA	NA
hsa-miR-4277/hsa-mir-4277	0	0	0	NA	NA	NA	NA	NA
hsa-miR-4278/hsa-mir-4278	0	0	0	NA	NA	NA	NA	NA
hsa-miR-4279/hsa-mir-4279	0	0	0	NA	NA	NA	NA	NA
hsa-miR-4280/hsa-mir-4280	0	0	0	NA	NA	NA	NA	NA
hsa-miR-4281/hsa-mir-4281	0	0	0	NA	NA	NA	NA	NA
hsa-miR-4282/hsa-mir-4282	0	0	0	NA	NA	NA	NA	NA
hsa-miR-4283/hsa-mir-4283–1	0	0	0	NA	NA	NA	NA	NA
hsa-miR-4283/hsa-mir-4283–2	0	0	0	NA	NA	NA	NA	NA
hsa-miR-4284/hsa-mir-4284	1.630666	1.634036	1.627296	− 0.1373	3.245486	− 0.04231	0.966255	NA
hsa-miR-4285/hsa-mir-4285	0	0	0	NA	NA	NA	NA	NA
hsa-miR-4287/hsa-mir-4287	0	0	0	NA	NA	NA	NA	NA
hsa-miR-4288/hsa-mir-4288	0	0	0	NA	NA	NA	NA	NA
hsa-miR-4289/hsa-mir-4289	0	0	0	NA	NA	NA	NA	NA
hsa-miR-4290/hsa-mir-4290	0	0	0	NA	NA	NA	NA	NA
hsa-miR-4291/hsa-mir-4291	0	0	0	NA	NA	NA	NA	NA
hsa-miR-4292/hsa-mir-4292	0	0	0	NA	NA	NA	NA	NA
hsa-miR-4293/hsa-mir-4293	0	0	0	NA	NA	NA	NA	NA
hsa-miR-4294/hsa-mir-4294	0	0	0	NA	NA	NA	NA	NA
hsa-miR-4295/hsa-mir-4295	0	0	0	NA	NA	NA	NA	NA
hsa-miR-4296/hsa-mir-4296	0	0	0	NA	NA	NA	NA	NA
hsa-miR-4297/hsa-mir-4297	0	0	0	NA	NA	NA	NA	NA
hsa-miR-4298/hsa-mir-4298	0	0	0	NA	NA	NA	NA	NA
hsa-miR-4299/hsa-mir-4299	0	0	0	NA	NA	NA	NA	NA
hsa-miR-4300/hsa-mir-4300	0	0	0	NA	NA	NA	NA	NA
hsa-miR-4301/hsa-mir-4301	1.617551	0.509196	2.725907	2.456594	4.044713	0.607359	0.543612	NA
hsa-miR-4302/hsa-mir-4302	0	0	0	NA	NA	NA	NA	NA
hsa-miR-4303/hsa-mir-4303	0	0	0	NA	NA	NA	NA	NA
hsa-miR-4304/hsa-mir-4304	0	0	0	NA	NA	NA	NA	NA
hsa-miR-4305/hsa-mir-4305	0	0	0	NA	NA	NA	NA	NA
hsa-miR-4306/hsa-mir-4306	0.835388	0.307822	1.362953	1.665763	4.892293	0.340487	0.73349	NA
hsa-miR-4307/hsa-mir-4307	0	0	0	NA	NA	NA	NA	NA
hsa-miR-4308/hsa-mir-4308	0	0	0	NA	NA	NA	NA	NA
hsa-miR-4309/hsa-mir-4309	0	0	0	NA	NA	NA	NA	NA
hsa-miR-4310/hsa-mir-4310	0	0	0	NA	NA	NA	NA	NA
hsa-miR-4311/hsa-mir-4311	0	0	0	NA	NA	NA	NA	NA
hsa-miR-4312/hsa-mir-4312	0	0	0	NA	NA	NA	NA	NA
hsa-miR-4313/hsa-mir-4313	0	0	0	NA	NA	NA	NA	NA
hsa-miR-431-3p/hsa-mir-431	0	0	0	NA	NA	NA	NA	NA
hsa-miR-4314/hsa-mir-4314	0	0	0	NA	NA	NA	NA	NA
hsa-miR-4315/hsa-mir-4315–1	0	0	0	NA	NA	NA	NA	NA
hsa-miR-4315/hsa-mir-4315–2	0	0	0	NA	NA	NA	NA	NA
hsa-miR-431-5p/hsa-mir-431	0.153911	0.307822	0	− 0.74599	4.97963	− 0.14981	0.880917	NA
hsa-miR-4316/hsa-mir-4316	0	0	0	NA	NA	NA	NA	NA
hsa-miR-4317/hsa-mir-4317	0	0	0	NA	NA	NA	NA	NA
hsa-miR-4318/hsa-mir-4318	0	0	0	NA	NA	NA	NA	NA
hsa-miR-4319/hsa-mir-4319	0	0	0	NA	NA	NA	NA	NA
hsa-miR-4320/hsa-mir-4320	0	0	0	NA	NA	NA	NA	NA
hsa-miR-4321/hsa-mir-4321	0	0	0	NA	NA	NA	NA	NA
hsa-miR-4322/hsa-mir-4322	0	0	0	NA	NA	NA	NA	NA
hsa-miR-4323/hsa-mir-4323	0	0	0	NA	NA	NA	NA	NA
hsa-miR-432-3p/hsa-mir-432	0	0	0	NA	NA	NA	NA	NA
hsa-miR-4324/hsa-mir-4324	0	0	0	NA	NA	NA	NA	NA
hsa-miR-4325/hsa-mir-4325	0	0	0	NA	NA	NA	NA	NA
hsa-miR-432-5p/hsa-mir-432	1.935626	3.078223	0.793028	− 1.62015	3.64061	− 0.44502	0.656304	NA
hsa-miR-4327/hsa-mir-4327	0	0	0	NA	NA	NA	NA	NA
hsa-miR-4328/hsa-mir-4328	0	0	0	NA	NA	NA	NA	NA
hsa-miR-4329/hsa-mir-4329	0	0	0	NA	NA	NA	NA	NA
hsa-miR-4330/hsa-mir-4330	0	0	0	NA	NA	NA	NA	NA
hsa-miR-433-3p/hsa-mir-433	0	0	0	NA	NA	NA	NA	NA
hsa-miR-433-5p/hsa-mir-433	0	0	0	NA	NA	NA	NA	NA
hsa-miR-4418/hsa-mir-4418	0	0	0	NA	NA	NA	NA	NA
hsa-miR-4420/hsa-mir-4420	0.386769	0.509196	0.264343	0.108828	4.90556	0.022185	0.982301	NA
hsa-miR-4421/hsa-mir-4421	2.475186	3.587419	1.362953	− 1.86976	3.146946	− 0.59415	0.552411	NA
hsa-miR-4422/hsa-mir-4422	1.516865	0.307822	2.725907	2.792606	4.127289	0.67662	0.498647	NA
hsa-miR-4423-3p/hsa-mir-4423	0.132171	0	0.264343	1.823505	4.944331	0.368807	0.712271	NA
hsa-miR-4423-5p/hsa-mir-4423	0.550425	0.307822	0.793028	1.42144	4.897318	0.290249	0.771626	NA
hsa-miR-4424/hsa-mir-4424	1.823415	2.853801	0.793028	− 1.4681	3.382847	− 0.43398	0.6643	NA
hsa-miR-4425/hsa-mir-4425	5.070512	4.689211	5.451813	0.129669	2.520784	0.05144	0.958975	NA
hsa-miR-4426/hsa-mir-4426	0	0	0	NA	NA	NA	NA	NA
hsa-miR-4427/hsa-mir-4427	0.264343	0	0.528686	2.396631	4.924788	0.486646	0.626509	NA
hsa-miR-4428/hsa-mir-4428	1.386933	0.923467	1.8504	1.06221	4.066831	0.261189	0.793947	NA
hsa-miR-4429/hsa-mir-4429	2.014065	0.509196	3.518935	2.898018	3.354378	0.863951	0.387615	NA
hsa-miR-4430/hsa-mir-4430	0	0	0	NA	NA	NA	NA	NA
hsa-miR-4431/hsa-mir-4431	0	0	0	NA	NA	NA	NA	NA
hsa-miR-4432/hsa-mir-4432	0	0	0	NA	NA	NA	NA	NA
hsa-miR-4433a-3p/hsa-mir-4433a	0	0	0	NA	NA	NA	NA	NA
hsa-miR-4433a-5p/hsa-mir-4433a	0	0	0	NA	NA	NA	NA	NA
hsa-miR-4433b-3p/hsa-mir-4433b	0	0	0	NA	NA	NA	NA	NA
hsa-miR-4433b-5p/hsa-mir-4433b	0	0	0	NA	NA	NA	NA	NA
hsa-miR-4434/hsa-mir-4434	0	0	0	NA	NA	NA	NA	NA
hsa-miR-4435/hsa-mir-4435–1	2.951767	3.788792	2.114743	− 0.69035	2.763534	− 0.24981	0.802737	NA
hsa-miR-4435/hsa-mir-4435–2	2.951767	3.788792	2.114743	− 0.69035	2.763534	− 0.24981	0.802737	NA
hsa-miR-4436a/hsa-mir-4436a	0	0	0	NA	NA	NA	NA	NA
hsa-miR-4436b-3p/hsa-mir-4436b-1	0	0	0	NA	NA	NA	NA	NA
hsa-miR-4436b-3p/hsa-mir-4436b-2	0	0	0	NA	NA	NA	NA	NA
hsa-miR-4436b-5p/hsa-mir-4436b-1	0	0	0	NA	NA	NA	NA	NA
hsa-miR-4436b-5p/hsa-mir-4436b-2	0	0	0	NA	NA	NA	NA	NA
hsa-miR-4437/hsa-mir-4437	0	0	0	NA	NA	NA	NA	NA
hsa-miR-4438/hsa-mir-4438	0	0	0	NA	NA	NA	NA	NA
hsa-miR-4439/hsa-mir-4439	0.307822	0.615645	0	− 1.51932	4.922146	− 0.30867	0.757573	NA
hsa-miR-4440/hsa-mir-4440	1.991713	2.35613	1.627296	− 0.70777	3.052637	− 0.23186	0.81665	NA
hsa-miR-4441/hsa-mir-4441	0	0	0	NA	NA	NA	NA	NA
hsa-miR-4442/hsa-mir-4442	0	0	0	NA	NA	NA	NA	NA
hsa-miR-4443/hsa-mir-4443	0	0	0	NA	NA	NA	NA	NA
hsa-miR-4444/hsa-mir-4444–1	0.153911	0.307822	0	− 0.74599	4.97963	− 0.14981	0.880917	NA
hsa-miR-4444/hsa-mir-4444–2	0.153911	0.307822	0	− 0.74599	4.97963	− 0.14981	0.880917	NA
hsa-miR-4445-3p/hsa-mir-4445	0	0	0	NA	NA	NA	NA	NA
hsa-miR-4445-5p/hsa-mir-4445	0.307822	0.615645	0	− 1.51932	4.922146	− 0.30867	0.757573	NA
hsa-miR-4446-3p/hsa-mir-4446	3.661981	0.509196	6.814767	3.856544	3.18922	1.209244	0.226569	NA
hsa-miR-4446-5p/hsa-mir-4446	0.254598	0.509196	0	− 1.11434	4.959133	− 0.2247	0.82221	NA
hsa-miR-4447/hsa-mir-4447	0.615645	1.231289	0	− 2.50085	4.855042	− 0.5151	0.606481	NA
hsa-miR-4449/hsa-mir-4449	0.254598	0.509196	0	− 1.11434	4.959133	− 0.2247	0.82221	NA
hsa-miR-4450/hsa-mir-4450	1.189543	0	2.379085	4.089116	4.831661	0.846317	0.397376	NA
hsa-miR-4451/hsa-mir-4451	0	0	0	NA	NA	NA	NA	NA
hsa-miR-4452/hsa-mir-4452	0	0	0	NA	NA	NA	NA	NA
hsa-miR-4453/hsa-mir-4453	0	0	0	NA	NA	NA	NA	NA
hsa-miR-4455/hsa-mir-4455	0	0	0	NA	NA	NA	NA	NA
hsa-miR-4456/hsa-mir-4456	0	0	0	NA	NA	NA	NA	NA
hsa-miR-4457/hsa-mir-4457	1.342334	0	2.684667	4.202922	4.165197	1.009057	0.312947	NA
hsa-miR-4460/hsa-mir-4460	0	0	0	NA	NA	NA	NA	NA
hsa-miR-4462/hsa-mir-4462	0	0	0	NA	NA	NA	NA	NA
hsa-miR-4463/hsa-mir-4463	0.132171	0	0.264343	1.823505	4.944331	0.368807	0.712271	NA
hsa-miR-4464/hsa-mir-4464	0	0	0	NA	NA	NA	NA	NA
hsa-miR-4465/hsa-mir-4465	0	0	0	NA	NA	NA	NA	NA
hsa-miR-4466/hsa-mir-4466	0	0	0	NA	NA	NA	NA	NA
hsa-miR-4468/hsa-mir-4468	0	0	0	NA	NA	NA	NA	NA
hsa-miR-4469/hsa-mir-4469	0.396514	0	0.793028	2.767752	4.915475	0.563069	0.573388	NA
hsa-miR-4470/hsa-mir-4470	5.413322	3.078223	7.74842	1.320044	2.313468	0.570591	0.568277	NA
hsa-miR-4471/hsa-mir-4471	0.307822	0.615645	0	− 1.51932	4.922146	− 0.30867	0.757573	NA
hsa-miR-4472/hsa-mir-4472–1	0	0	0	NA	NA	NA	NA	NA
hsa-miR-4472/hsa-mir-4472–2	0	0	0	NA	NA	NA	NA	NA
hsa-miR-4473/hsa-mir-4473	2.725907	0	5.451813	5.173154	3.717745	1.391476	0.164081	NA
hsa-miR-4474-3p/hsa-mir-4474	3.733424	1.527587	5.93926	1.984655	2.628971	0.754917	0.450299	NA
hsa-miR-4474-5p/hsa-mir-4474	0.509196	1.018392	0	− 2.1782	4.872426	− 0.44705	0.654841	NA
hsa-miR-4475/hsa-mir-4475	0	0	0	NA	NA	NA	NA	NA
hsa-miR-4476/hsa-mir-4476	0	0	0	NA	NA	NA	NA	NA
hsa-miR-4477a/hsa-mir-4477a	0.286083	0.307822	0.264343	0.477194	4.926281	0.096867	0.922832	NA
hsa-miR-4478/hsa-mir-4478	0	0	0	NA	NA	NA	NA	NA
hsa-miR-4479/hsa-mir-4479	0.858248	0.923467	0.793028	0.075134	4.793669	0.015673	0.987495	NA
hsa-miR-4480/hsa-mir-4480	0.936075	0.509196	1.362953	1.297386	4.871427	0.266326	0.789988	NA
hsa-miR-4481/hsa-mir-4481	0	0	0	NA	NA	NA	NA	NA
hsa-miR-4482-3p/hsa-mir-4482	4.007502	0.307822	7.707181	4.405043	2.75489	1.598991	0.109823	NA
hsa-miR-4482-5p/hsa-mir-4482	0.813648	0	1.627296	3.418215	4.892272	0.698697	0.484741	NA
hsa-miR-4483/hsa-mir-4483	1.671283	3.078223	0.264343	− 2.59127	3.872991	− 0.66906	0.503456	NA
hsa-miR-4485-5p/hsa-mir-4485	0.641367	1.018392	0.264343	− 0.95503	4.817889	− 0.19822	0.842869	NA
hsa-miR-4486/hsa-mir-4486	0	0	0	NA	NA	NA	NA	NA
hsa-miR-4487/hsa-mir-4487	0	0	0	NA	NA	NA	NA	NA
hsa-miR-4488/hsa-mir-4488	0.153911	0.307822	0	− 0.74599	4.97963	− 0.14981	0.880917	NA
hsa-miR-4489/hsa-mir-4489	0.945819	0	1.891639	3.662379	4.791539	0.764343	0.444663	NA
hsa-miR-4490/hsa-mir-4490	0	0	0	NA	NA	NA	NA	NA
hsa-miR-4491/hsa-mir-4491	0	0	0	NA	NA	NA	NA	NA
hsa-miR-4492/hsa-mir-4492	0	0	0	NA	NA	NA	NA	NA
hsa-miR-4493/hsa-mir-4493	0	0	0	NA	NA	NA	NA	NA
hsa-miR-4494/hsa-mir-4494	0	0	0	NA	NA	NA	NA	NA
hsa-miR-4495/hsa-mir-4495	0	0	0	NA	NA	NA	NA	NA
hsa-miR-4496/hsa-mir-4496	0	0	0	NA	NA	NA	NA	NA
hsa-miR-4497/hsa-mir-4497	0	0	0	NA	NA	NA	NA	NA
hsa-miR-4498/hsa-mir-4498	0	0	0	NA	NA	NA	NA	NA
hsa-miR-4499/hsa-mir-4499	0	0	0	NA	NA	NA	NA	NA
hsa-miR-449b-3p/hsa-mir-449b	0	0	0	NA	NA	NA	NA	NA
hsa-miR-449c-3p/hsa-mir-449c	0	0	0	NA	NA	NA	NA	NA
hsa-miR-4500/hsa-mir-4500	0	0	0	NA	NA	NA	NA	NA
hsa-miR-4501/hsa-mir-4501	1.3852	2.770401	0	− 3.6603	4.585312	− 0.79827	0.424716	NA
hsa-miR-4502/hsa-mir-4502	2.420325	0	4.840649	5.045554	3.365694	1.499113	0.133844	NA
hsa-miR-4503/hsa-mir-4503	0.132171	0	0.264343	1.823505	4.944331	0.368807	0.712271	NA
hsa-miR-4505/hsa-mir-4505	0	0	0	NA	NA	NA	NA	NA
hsa-miR-4506/hsa-mir-4506	0	0	0	NA	NA	NA	NA	NA
hsa-miR-4507/hsa-mir-4507	0	0	0	NA	NA	NA	NA	NA
hsa-miR-4508/hsa-mir-4508	2.725907	0	5.451813	5.173154	3.717745	1.391476	0.164081	NA
hsa-miR-4509/hsa-mir-4509–1	0	0	0	NA	NA	NA	NA	NA
hsa-miR-4509/hsa-mir-4509–2	0	0	0	NA	NA	NA	NA	NA
hsa-miR-4509/hsa-mir-4509–3	0	0	0	NA	NA	NA	NA	NA
hsa-miR-450b-3p/hsa-mir-450b	3.28068	5.198407	1.362953	− 2.44851	2.895165	− 0.84572	0.397707	NA
hsa-miR-4511/hsa-mir-4511	5.511521	10.23001	0.793028	− 3.25329	2.318393	− 1.40325	0.160541	NA
hsa-miR-4512/hsa-mir-4512	0.153911	0.307822	0	− 0.74599	4.97963	− 0.14981	0.880917	NA
hsa-miR-4513/hsa-mir-4513	0	0	0	NA	NA	NA	NA	NA
hsa-miR-4514/hsa-mir-4514	0	0	0	NA	NA	NA	NA	NA
hsa-miR-4515/hsa-mir-4515	0	0	0	NA	NA	NA	NA	NA
hsa-miR-4518/hsa-mir-4518	0.681477	0	1.362953	3.012055	4.910469	0.613395	0.539615	NA
hsa-miR-4519/hsa-mir-4519	0.593905	0.923467	0.264343	− 0.86911	4.823254	− 0.18019	0.857002	NA
hsa-miR-451a/hsa-mir-451a	3.51593	3.777268	3.254592	− 0.32886	2.425916	− 0.13556	0.892169	NA
hsa-miR-451b/hsa-mir-451b	0	0	0	NA	NA	NA	NA	NA
hsa-miR-4520–2-3p/hsa-mir-4520–2	2.513507	1.326214	3.700799	1.599716	2.999844	0.533266	0.593849	NA
hsa-miR-4520-3p/hsa-mir-4520–1	2.898026	0.509196	5.286856	3.606541	3.271623	1.102371	0.270301	NA
hsa-miR-4520-5p/hsa-mir-4520–1	0.264343	0	0.528686	2.396631	4.924788	0.486646	0.626509	NA
hsa-miR-4520-5p/hsa-mir-4520–2	0.264343	0	0.528686	2.396631	4.924788	0.486646	0.626509	NA
hsa-miR-4522/hsa-mir-4522	0.307822	0.615645	0	− 1.51932	4.922146	− 0.30867	0.757573	NA
hsa-miR-4523/hsa-mir-4523	3.957657	2.545979	5.369335	1.111972	2.578494	0.431249	0.666288	NA
hsa-miR-452-3p/hsa-mir-452	0	0	0	NA	NA	NA	NA	NA
hsa-miR-4524a-3p/hsa-mir-4524a	2.510041	5.020082	0	− 4.51444	3.335272	− 1.35355	0.175881	NA
hsa-miR-4524a-5p/hsa-mir-4524a	0.153911	0.307822	0	− 0.74599	4.97963	− 0.14981	0.880917	NA
hsa-miR-4524b-3p/hsa-mir-4524b	0	0	0	NA	NA	NA	NA	NA
hsa-miR-4524b-5p/hsa-mir-4524b	0	0	0	NA	NA	NA	NA	NA
hsa-miR-4525/hsa-mir-4525	3.038219	2.557503	3.518935	0.366842	2.517616	0.14571	0.88415	NA
hsa-miR-4526/hsa-mir-4526	0	0	0	NA	NA	NA	NA	NA
hsa-miR-4527/hsa-mir-4527	0.769556	1.539112	0	− 2.81872	4.841262	− 0.58223	0.560413	NA
hsa-miR-4528/hsa-mir-4528	0	0	0	NA	NA	NA	NA	NA
hsa-miR-4529-3p/hsa-mir-4529	0.132171	0	0.264343	1.823505	4.944331	0.368807	0.712271	NA
hsa-miR-4529-5p/hsa-mir-4529	0	0	0	NA	NA	NA	NA	NA
hsa-miR-4530/hsa-mir-4530	0	0	0	NA	NA	NA	NA	NA
hsa-miR-4531/hsa-mir-4531	0	0	0	NA	NA	NA	NA	NA
hsa-miR-4533/hsa-mir-4533	0	0	0	NA	NA	NA	NA	NA
hsa-miR-4534/hsa-mir-4534	0	0	0	NA	NA	NA	NA	NA
hsa-miR-4535/hsa-mir-4535	0	0	0	NA	NA	NA	NA	NA
hsa-miR-4536-3p/hsa-mir-4536–1	0.56242	1.12484	0	− 2.35102	4.862644	− 0.48349	0.62875	NA
hsa-miR-4536-3p/hsa-mir-4536–2	0.56242	1.12484	0	− 2.35102	4.862644	− 0.48349	0.62875	NA
hsa-miR-4536-5p/hsa-mir-4536–1	1.829177	2.865326	0.793028	− 1.49226	3.303976	− 0.45166	0.651517	NA
hsa-miR-4536-5p/hsa-mir-4536–2	1.829177	2.865326	0.793028	− 1.49226	3.303976	− 0.45166	0.651517	NA
hsa-miR-4537/hsa-mir-4537	0	0	0	NA	NA	NA	NA	NA
hsa-miR-4538/hsa-mir-4538	0	0	0	NA	NA	NA	NA	NA
hsa-miR-4539/hsa-mir-4539	0	0	0	NA	NA	NA	NA	NA
hsa-miR-4540/hsa-mir-4540	0	0	0	NA	NA	NA	NA	NA
hsa-miR-4632-3p/hsa-mir-4632	0.132171	0	0.264343	1.823505	4.944331	0.368807	0.712271	NA
hsa-miR-4632-5p/hsa-mir-4632	0	0	0	NA	NA	NA	NA	NA
hsa-miR-4633-3p/hsa-mir-4633	0	0	0	NA	NA	NA	NA	NA
hsa-miR-4633-5p/hsa-mir-4633	0	0	0	NA	NA	NA	NA	NA
hsa-miR-4634/hsa-mir-4634	0	0	0	NA	NA	NA	NA	NA
hsa-miR-4635/hsa-mir-4635	0.826763	1.12484	0.528686	− 0.5515	4.504759	− 0.12243	0.902562	NA
hsa-miR-4637/hsa-mir-4637	1.319981	1.846934	0.793028	− 0.90304	4.166385	− 0.21675	0.828407	NA
hsa-miR-4638-3p/hsa-mir-4638	0.286083	0.307822	0.264343	0.477194	4.926281	0.096867	0.922832	NA
hsa-miR-4638-5p/hsa-mir-4638	0	0	0	NA	NA	NA	NA	NA
hsa-miR-4639-3p/hsa-mir-4639	0	0	0	NA	NA	NA	NA	NA
hsa-miR-4639-5p/hsa-mir-4639	1.322844	1.018392	1.627296	0.619295	3.878599	0.15967	0.873141	NA
hsa-miR-4640-3p/hsa-mir-4640	2.440944	0	4.881889	5.018156	3.401403	1.475319	0.140127	NA
hsa-miR-4640-5p/hsa-mir-4640	5.952375	1.12484	10.77991	3.228853	2.09669	1.539976	0.123566	NA
hsa-miR-4641/hsa-mir-4641	2.198341	0.307822	4.08886	3.38492	3.663309	0.924006	0.355483	NA
hsa-miR-4642/hsa-mir-4642	0	0	0	NA	NA	NA	NA	NA
hsa-miR-4643/hsa-mir-4643	0.153911	0.307822	0	− 0.74599	4.97963	− 0.14981	0.880917	NA
hsa-miR-4644/hsa-mir-4644	0.681477	0	1.362953	3.012055	4.910469	0.613395	0.539615	NA
hsa-miR-4645-5p/hsa-mir-4645	0	0	0	NA	NA	NA	NA	NA
hsa-miR-4646-3p/hsa-mir-4646	0	0	0	NA	NA	NA	NA	NA
hsa-miR-4646-5p/hsa-mir-4646	0	0	0	NA	NA	NA	NA	NA
hsa-miR-4647/hsa-mir-4647	0	0	0	NA	NA	NA	NA	NA
hsa-miR-4648/hsa-mir-4648	1.453885	0	2.907771	4.376967	4.474595	0.978182	0.327984	NA
hsa-miR-4649-3p/hsa-mir-4649	0	0	0	NA	NA	NA	NA	NA
hsa-miR-4649-5p/hsa-mir-4649	0	0	0	NA	NA	NA	NA	NA
hsa-miR-4650-3p/hsa-mir-4650–1	0	0	0	NA	NA	NA	NA	NA
hsa-miR-4650-3p/hsa-mir-4650–2	0	0	0	NA	NA	NA	NA	NA
hsa-miR-4650-5p/hsa-mir-4650–1	0	0	0	NA	NA	NA	NA	NA
hsa-miR-4650-5p/hsa-mir-4650–2	0	0	0	NA	NA	NA	NA	NA
hsa-miR-4651/hsa-mir-4651	0.153911	0.307822	0	− 0.74599	4.97963	− 0.14981	0.880917	NA
hsa-miR-4652-3p/hsa-mir-4652	1.452153	1.846934	1.057371	− 0.61922	4.015232	− 0.15422	0.877438	NA
hsa-miR-4653-3p/hsa-mir-4653	1.377188	1.432663	1.321714	0.024385	3.685762	0.006616	0.994721	NA
hsa-miR-4653-5p/hsa-mir-4653	0.153911	0.307822	0	− 0.74599	4.97963	− 0.14981	0.880917	NA
hsa-miR-4654/hsa-mir-4654	0	0	0	NA	NA	NA	NA	NA
hsa-miR-4655-3p/hsa-mir-4655	0.254598	0.509196	0	− 1.11434	4.959133	− 0.2247	0.82221	NA
hsa-miR-4655-5p/hsa-mir-4655	0	0	0	NA	NA	NA	NA	NA
hsa-miR-4656/hsa-mir-4656	0	0	0	NA	NA	NA	NA	NA
hsa-miR-4657/hsa-mir-4657	0	0	0	NA	NA	NA	NA	NA
hsa-miR-4658/hsa-mir-4658	0	0	0	NA	NA	NA	NA	NA
hsa-miR-4659a-3p/hsa-mir-4659a	4.226019	3.693868	4.758171	0.425169	2.787167	0.152545	0.878757	NA
hsa-miR-4659a-5p/hsa-mir-4659a	0.814768	0.307822	1.321714	1.93264	4.884354	0.39568	0.692341	NA
hsa-miR-4659b-3p/hsa-mir-4659b	0	0	0	NA	NA	NA	NA	NA
hsa-miR-4659b-5p/hsa-mir-4659b	0	0	0	NA	NA	NA	NA	NA
hsa-miR-4660/hsa-mir-4660	1.266757	1.740485	0.793028	− 0.79738	3.810955	− 0.20923	0.834267	NA
hsa-miR-4661-3p/hsa-mir-4661	0	0	0	NA	NA	NA	NA	NA
hsa-miR-4661-5p/hsa-mir-4661	0.773539	1.018392	0.528686	− 0.3819	4.797831	− 0.0796	0.936556	NA
hsa-miR-4662a-3p/hsa-mir-4662a	0.132171	0	0.264343	1.823505	4.944331	0.368807	0.712271	NA
hsa-miR-4662b/hsa-mir-4662b	0	0	0	NA	NA	NA	NA	NA
hsa-miR-4663/hsa-mir-4663	0	0	0	NA	NA	NA	NA	NA
hsa-miR-4664-3p/hsa-mir-4664	5.787589	0.307822	11.26736	4.904348	2.460866	1.992936	0.046268	NA
hsa-miR-4665-3p/hsa-mir-4665	0	0	0	NA	NA	NA	NA	NA
hsa-miR-4665-5p/hsa-mir-4665	0	0	0	NA	NA	NA	NA	NA
hsa-miR-4666a-3p/hsa-mir-4666a	0	0	0	NA	NA	NA	NA	NA
hsa-miR-4666a-5p/hsa-mir-4666a	0.286083	0.307822	0.264343	0.477194	4.926281	0.096867	0.922832	NA
hsa-miR-4666b/hsa-mir-4666b	0	0	0	NA	NA	NA	NA	NA
hsa-miR-4667-3p/hsa-mir-4667	0.396514	0	0.793028	2.767752	4.915475	0.563069	0.573388	NA
hsa-miR-4667-5p/hsa-mir-4667	0.936075	0.509196	1.362953	1.297386	4.871427	0.266326	0.789988	NA
hsa-miR-4668-3p/hsa-mir-4668	0.550425	0.307822	0.793028	1.42144	4.897318	0.290249	0.771626	NA
hsa-miR-4669/hsa-mir-4669	0.132171	0	0.264343	1.823505	4.944331	0.368807	0.712271	NA
hsa-miR-466/hsa-mir-466	0	0	0	NA	NA	NA	NA	NA
hsa-miR-4670-3p/hsa-mir-4670	0.817018	1.634036	0	− 2.88326	4.83881	− 0.59586	0.551267	NA
hsa-miR-4670-5p/hsa-mir-4670	0.132171	0	0.264343	1.823505	4.944331	0.368807	0.712271	NA
hsa-miR-4671-3p/hsa-mir-4671	4.422618	8.052208	0.793028	− 2.91753	2.552796	− 1.14288	0.253089	NA
hsa-miR-4671-5p/hsa-mir-4671	0.264343	0	0.528686	2.396631	4.924788	0.486646	0.626509	NA
hsa-miR-4672/hsa-mir-4672	0	0	0	NA	NA	NA	NA	NA
hsa-miR-4673/hsa-mir-4673	0.264343	0	0.528686	2.396631	4.924788	0.486646	0.626509	NA
hsa-miR-4674/hsa-mir-4674	0	0	0	NA	NA	NA	NA	NA
hsa-miR-4675/hsa-mir-4675	0	0	0	NA	NA	NA	NA	NA
hsa-miR-4676-3p/hsa-mir-4676	4.691736	4.096615	5.286856	0.451589	2.394781	0.188572	0.850428	NA
hsa-miR-4676-5p/hsa-mir-4676	0	0	0	NA	NA	NA	NA	NA
hsa-miR-4678/hsa-mir-4678	0	0	0	NA	NA	NA	NA	NA
hsa-miR-4679/hsa-mir-4679–1	0.153911	0.307822	0	− 0.74599	4.97963	− 0.14981	0.880917	NA
hsa-miR-4679/hsa-mir-4679–2	0.153911	0.307822	0	− 0.74599	4.97963	− 0.14981	0.880917	NA
hsa-miR-4680-3p/hsa-mir-4680	3.039197	5.814051	0.264343	− 3.48409	2.857783	− 1.21916	0.222784	NA
hsa-miR-4680-5p/hsa-mir-4680	0.254598	0.509196	0	− 1.11434	4.959133	− 0.2247	0.82221	NA
hsa-miR-4681/hsa-mir-4681	0	0	0	NA	NA	NA	NA	NA
hsa-miR-4682/hsa-mir-4682	0.153911	0.307822	0	− 0.74599	4.97963	− 0.14981	0.880917	NA
hsa-miR-4684-3p/hsa-mir-4684	4.443887	7.566061	1.321714	− 2.24736	2.418274	− 0.92932	0.352722	NA
hsa-miR-4684-5p/hsa-mir-4684	0	0	0	NA	NA	NA	NA	NA
hsa-miR-4685-3p/hsa-mir-4685	0.132171	0	0.264343	1.823505	4.944331	0.368807	0.712271	NA
hsa-miR-4685-5p/hsa-mir-4685	0	0	0	NA	NA	NA	NA	NA
hsa-miR-4686/hsa-mir-4686	0	0	0	NA	NA	NA	NA	NA
hsa-miR-4687-3p/hsa-mir-4687	0.132171	0	0.264343	1.823505	4.944331	0.368807	0.712271	NA
hsa-miR-4687-5p/hsa-mir-4687	0	0	0	NA	NA	NA	NA	NA
hsa-miR-4688/hsa-mir-4688	0.681477	0	1.362953	3.012055	4.910469	0.613395	0.539615	NA
hsa-miR-4689/hsa-mir-4689	0.704337	0.615645	0.793028	0.648104	4.838855	0.133937	0.893452	NA
hsa-miR-4690-3p/hsa-mir-4690	1.70563	2.048307	1.362953	− 0.97765	3.532176	− 0.27678	0.781946	NA
hsa-miR-4690-5p/hsa-mir-4690	0.153911	0.307822	0	− 0.74599	4.97963	− 0.14981	0.880917	NA
hsa-miR-4691-3p/hsa-mir-4691	0.793028	0	1.586057	3.52828	4.884751	0.722305	0.470107	NA
hsa-miR-4691-5p/hsa-mir-4691	0.264343	0	0.528686	2.396631	4.924788	0.486646	0.626509	NA
hsa-miR-4692/hsa-mir-4692	0	0	0	NA	NA	NA	NA	NA
hsa-miR-4693-3p/hsa-mir-4693	0	0	0	NA	NA	NA	NA	NA
hsa-miR-4693-5p/hsa-mir-4693	0	0	0	NA	NA	NA	NA	NA
hsa-miR-4694-3p/hsa-mir-4694	1.344584	1.326214	1.362953	− 0.26664	3.861573	− 0.06905	0.944951	NA
hsa-miR-4694-5p/hsa-mir-4694	0	0	0	NA	NA	NA	NA	NA
hsa-miR-4695-3p/hsa-mir-4695	0.153911	0.307822	0	− 0.74599	4.97963	− 0.14981	0.880917	NA
hsa-miR-4695-5p/hsa-mir-4695	0	0	0	NA	NA	NA	NA	NA
hsa-miR-4696/hsa-mir-4696	0	0	0	NA	NA	NA	NA	NA
hsa-miR-4697-3p/hsa-mir-4697	0	0	0	NA	NA	NA	NA	NA
hsa-miR-4697-5p/hsa-mir-4697	0	0	0	NA	NA	NA	NA	NA
hsa-miR-4698/hsa-mir-4698	0	0	0	NA	NA	NA	NA	NA
hsa-miR-4699-3p/hsa-mir-4699	0.386769	0.509196	0.264343	0.108828	4.90556	0.022185	0.982301	NA
hsa-miR-4699-5p/hsa-mir-4699	0.518941	0.509196	0.528686	0.681945	4.885862	0.139575	0.888996	NA
hsa-miR-4700-3p/hsa-mir-4700	0	0	0	NA	NA	NA	NA	NA
hsa-miR-4700-5p/hsa-mir-4700	0	0	0	NA	NA	NA	NA	NA
hsa-miR-4701-3p/hsa-mir-4701	0	0	0	NA	NA	NA	NA	NA
hsa-miR-4701-5p/hsa-mir-4701	0	0	0	NA	NA	NA	NA	NA
hsa-miR-4703-3p/hsa-mir-4703	0	0	0	NA	NA	NA	NA	NA
hsa-miR-4703-5p/hsa-mir-4703	0	0	0	NA	NA	NA	NA	NA
hsa-miR-4704-3p/hsa-mir-4704	2.114743	0	4.229485	4.913958	3.913804	1.255545	0.209281	NA
hsa-miR-4704-5p/hsa-mir-4704	0	0	0	NA	NA	NA	NA	NA
hsa-miR-4705/hsa-mir-4705	4.582762	9.165524	0	− 5.36727	3.215009	− 1.66944	0.09503	NA
hsa-miR-4706/hsa-mir-4706	0.254598	0.509196	0	− 1.11434	4.959133	− 0.2247	0.82221	NA
hsa-miR-4707-3p/hsa-mir-4707	0.132171	0	0.264343	1.823505	4.944331	0.368807	0.712271	NA
hsa-miR-4707-5p/hsa-mir-4707	0	0	0	NA	NA	NA	NA	NA
hsa-miR-4708-3p/hsa-mir-4708	0.651112	0.509196	0.793028	1.053061	4.876474	0.215947	0.829029	NA
hsa-miR-4708-5p/hsa-mir-4708	0	0	0	NA	NA	NA	NA	NA
hsa-miR-4709-3p/hsa-mir-4709	0.980674	1.432663	0.528686	− 0.90089	4.207238	− 0.21413	0.830447	NA
hsa-miR-4709-5p/hsa-mir-4709	0	0	0	NA	NA	NA	NA	NA
hsa-miR-4710/hsa-mir-4710	0	0	0	NA	NA	NA	NA	NA
hsa-miR-4711-3p/hsa-mir-4711	0	0	0	NA	NA	NA	NA	NA
hsa-miR-4711-5p/hsa-mir-4711	0	0	0	NA	NA	NA	NA	NA
hsa-miR-4712-3p/hsa-mir-4712	0	0	0	NA	NA	NA	NA	NA
hsa-miR-4712-5p/hsa-mir-4712	0.307822	0.615645	0	− 1.51932	4.922146	− 0.30867	0.757573	NA
hsa-miR-4713-3p/hsa-mir-4713	0.153911	0.307822	0	− 0.74599	4.97963	− 0.14981	0.880917	NA
hsa-miR-4713-5p/hsa-mir-4713	0.461733	0.923467	0	− 2.09228	4.877731	− 0.42895	0.667962	NA
hsa-miR-4714-3p/hsa-mir-4714	2.316633	3.575895	1.057371	− 1.4953	3.022873	− 0.49466	0.620839	NA
hsa-miR-4714-5p/hsa-mir-4714	0	0	0	NA	NA	NA	NA	NA
hsa-miR-4715-3p/hsa-mir-4715	0	0	0	NA	NA	NA	NA	NA
hsa-miR-4715-5p/hsa-mir-4715	0	0	0	NA	NA	NA	NA	NA
hsa-miR-4716-3p/hsa-mir-4716	0.153911	0.307822	0	− 0.74599	4.97963	− 0.14981	0.880917	NA
hsa-miR-4716-5p/hsa-mir-4716	0	0	0	NA	NA	NA	NA	NA
hsa-miR-4717-3p/hsa-mir-4717	1.771462	0.817018	2.725907	1.640826	3.534734	0.464201	0.642504	NA
hsa-miR-4717-5p/hsa-mir-4717	0	0	0	NA	NA	NA	NA	NA
hsa-miR-4718/hsa-mir-4718	0	0	0	NA	NA	NA	NA	NA
hsa-miR-4720-3p/hsa-mir-4720	0	0	0	NA	NA	NA	NA	NA
hsa-miR-4720-5p/hsa-mir-4720	0.254598	0.509196	0	− 1.11434	4.959133	− 0.2247	0.82221	NA
hsa-miR-4721/hsa-mir-4721	0	0	0	NA	NA	NA	NA	NA
hsa-miR-4722-3p/hsa-mir-4722	0	0	0	NA	NA	NA	NA	NA
hsa-miR-4722-5p/hsa-mir-4722	1.495125	0	2.99025	4.295677	4.079183	1.053073	0.292308	NA
hsa-miR-4723-3p/hsa-mir-4723	0	0	0	NA	NA	NA	NA	NA
hsa-miR-4723-5p/hsa-mir-4723	0	0	0	NA	NA	NA	NA	NA
hsa-miR-4724-3p/hsa-mir-4724	0	0	0	NA	NA	NA	NA	NA
hsa-miR-4724-5p/hsa-mir-4724	0	0	0	NA	NA	NA	NA	NA
hsa-miR-4725-3p/hsa-mir-4725	0.663107	1.326214	0	− 2.57515	4.851549	− 0.53079	0.595564	NA
hsa-miR-4725-5p/hsa-mir-4725	0	0	0	NA	NA	NA	NA	NA
hsa-miR-4726-3p/hsa-mir-4726	0	0	0	NA	NA	NA	NA	NA
hsa-miR-4726-5p/hsa-mir-4726	0.132171	0	0.264343	1.823505	4.944331	0.368807	0.712271	NA
hsa-miR-4727-3p/hsa-mir-4727	0	0	0	NA	NA	NA	NA	NA
hsa-miR-4727-5p/hsa-mir-4727	0.663107	1.326214	0	− 2.57515	4.851549	− 0.53079	0.595564	NA
hsa-miR-4728-3p/hsa-mir-4728	1.781207	0.307822	3.254592	3.059855	3.506857	0.872535	0.382917	NA
hsa-miR-4728-5p/hsa-mir-4728	0	0	0	NA	NA	NA	NA	NA
hsa-miR-4729/hsa-mir-4729	1.362953	0	2.725907	4.163156	4.684664	0.888677	0.374176	NA
hsa-miR-4730/hsa-mir-4730	0.439994	0.615645	0.264343	− 0.29614	4.868166	− 0.06083	0.951493	NA
hsa-miR-4731-3p/hsa-mir-4731	1.693023	3.386046	0	− 3.94939	4.265932	− 0.9258	0.354551	NA
hsa-miR-4731-5p/hsa-mir-4731	0.56242	1.12484	0	− 2.35102	4.862644	− 0.48349	0.62875	NA
hsa-miR-4732-3p/hsa-mir-4732	0	0	0	NA	NA	NA	NA	NA
hsa-miR-4732-5p/hsa-mir-4732	0	0	0	NA	NA	NA	NA	NA
hsa-miR-4733-3p/hsa-mir-4733	0	0	0	NA	NA	NA	NA	NA
hsa-miR-4733-5p/hsa-mir-4733	0.814768	0.307822	1.321714	1.93264	4.884354	0.39568	0.692341	NA
hsa-miR-4734/hsa-mir-4734	0	0	0	NA	NA	NA	NA	NA
hsa-miR-4735-3p/hsa-mir-4735	0	0	0	NA	NA	NA	NA	NA
hsa-miR-4735-5p/hsa-mir-4735	0.132171	0	0.264343	1.823505	4.944331	0.368807	0.712271	NA
hsa-miR-4736/hsa-mir-4736	0	0	0	NA	NA	NA	NA	NA
hsa-miR-4737/hsa-mir-4737	0.153911	0.307822	0	− 0.74599	4.97963	− 0.14981	0.880917	NA
hsa-miR-4738-3p/hsa-mir-4738	1.233022	0.615645	1.8504	1.628845	4.277544	0.38079	0.703359	NA
hsa-miR-4738-5p/hsa-mir-4738	0	0	0	NA	NA	NA	NA	NA
hsa-miR-4739/hsa-mir-4739	0.132171	0	0.264343	1.823505	4.944331	0.368807	0.712271	NA
hsa-miR-4740-3p/hsa-mir-4740	0	0	0	NA	NA	NA	NA	NA
hsa-miR-4740-5p/hsa-mir-4740	0.254598	0.509196	0	− 1.11434	4.959133	− 0.2247	0.82221	NA
hsa-miR-4741/hsa-mir-4741	0.704337	0.615645	0.793028	0.648104	4.838855	0.133937	0.893452	NA
hsa-miR-4743-3p/hsa-mir-4743	0	0	0	NA	NA	NA	NA	NA
hsa-miR-4743-5p/hsa-mir-4743	0.153911	0.307822	0	− 0.74599	4.97963	− 0.14981	0.880917	NA
hsa-miR-4744/hsa-mir-4744	0.769556	1.539112	0	− 2.81872	4.841262	− 0.58223	0.560413	NA
hsa-miR-4745-3p/hsa-mir-4745	0	0	0	NA	NA	NA	NA	NA
hsa-miR-4745-5p/hsa-mir-4745	0.945819	0	1.891639	3.662379	4.791539	0.764343	0.444663	NA
hsa-miR-4746-3p/hsa-mir-4746	0	0	0	NA	NA	NA	NA	NA
hsa-miR-4747-3p/hsa-mir-4747	0	0	0	NA	NA	NA	NA	NA
hsa-miR-4747-5p/hsa-mir-4747	1.071616	2.143232	0	− 3.27232	4.508741	− 0.72577	0.467978	NA
hsa-miR-4748/hsa-mir-4748	2.457429	2.758877	2.155982	− 0.40605	2.716027	− 0.1495	0.881159	NA
hsa-miR-4749-3p/hsa-mir-4749	0	0	0	NA	NA	NA	NA	NA
hsa-miR-4749-5p/hsa-mir-4749	0	0	0	NA	NA	NA	NA	NA
hsa-miR-4750-3p/hsa-mir-4750	0	0	0	NA	NA	NA	NA	NA
hsa-miR-4750-5p/hsa-mir-4750	0.813648	0	1.627296	3.418215	4.892272	0.698697	0.484741	NA
hsa-miR-4751/hsa-mir-4751	0	0	0	NA	NA	NA	NA	NA
hsa-miR-4752/hsa-mir-4752	0.132171	0	0.264343	1.823505	4.944331	0.368807	0.712271	NA
hsa-miR-4753-3p/hsa-mir-4753	3.467196	5.612678	1.321714	− 1.83673	2.638072	− 0.69624	0.486278	NA
hsa-miR-4753-5p/hsa-mir-4753	0.307822	0.615645	0	− 1.51932	4.922146	− 0.30867	0.757573	NA
hsa-miR-4754/hsa-mir-4754	2.838579	0.307822	5.369335	3.841245	2.976009	1.290737	0.196795	NA
hsa-miR-4755-3p/hsa-mir-4755	0	0	0	NA	NA	NA	NA	NA
hsa-miR-4755-5p/hsa-mir-4755	0.660857	0	1.321714	3.278941	4.90256	0.668822	0.503609	NA
hsa-miR-4756-3p/hsa-mir-4756	0	0	0	NA	NA	NA	NA	NA
hsa-miR-4756-5p/hsa-mir-4756	0	0	0	NA	NA	NA	NA	NA
hsa-miR-4757-3p/hsa-mir-4757	0.132171	0	0.264343	1.823505	4.944331	0.368807	0.712271	NA
hsa-miR-4757-5p/hsa-mir-4757	0.132171	0	0.264343	1.823505	4.944331	0.368807	0.712271	NA
hsa-miR-4758-3p/hsa-mir-4758	0.813648	0	1.627296	3.418215	4.892272	0.698697	0.484741	NA
hsa-miR-4758-5p/hsa-mir-4758	0.132171	0	0.264343	1.823505	4.944331	0.368807	0.712271	NA
hsa-miR-4759/hsa-mir-4759	0	0	0	NA	NA	NA	NA	NA
hsa-miR-4760-3p/hsa-mir-4760	0	0	0	NA	NA	NA	NA	NA
hsa-miR-4760-5p/hsa-mir-4760	2.379085	0	4.758171	5.082556	3.763125	1.350621	0.176817	NA
hsa-miR-4761-3p/hsa-mir-4761	5.23319	1.231289	9.235091	2.779787	2.407023	1.154865	0.248146	NA
hsa-miR-4761-5p/hsa-mir-4761	0	0	0	NA	NA	NA	NA	NA
hsa-miR-4762-3p/hsa-mir-4762	1.992833	2.663952	1.321714	− 0.84329	3.227261	− 0.2613	0.793858	NA
hsa-miR-4763-3p/hsa-mir-4763	0	0	0	NA	NA	NA	NA	NA
hsa-miR-4763-5p/hsa-mir-4763	0	0	0	NA	NA	NA	NA	NA
hsa-miR-4764-3p/hsa-mir-4764	0	0	0	NA	NA	NA	NA	NA
hsa-miR-4764-5p/hsa-mir-4764	0.132171	0	0.264343	1.823505	4.944331	0.368807	0.712271	NA
hsa-miR-4765/hsa-mir-4765	1.189543	0	2.379085	4.089116	4.831661	0.846317	0.397376	NA
hsa-miR-4767/hsa-mir-4767	0	0	0	NA	NA	NA	NA	NA
hsa-miR-4768-3p/hsa-mir-4768	0.386769	0.509196	0.264343	0.108828	4.90556	0.022185	0.982301	NA
hsa-miR-4768-5p/hsa-mir-4768	1.83204	2.036783	1.627296	− 0.41791	3.470051	− 0.12043	0.90414	NA
hsa-miR-4769-3p/hsa-mir-4769	0.509196	1.018392	0	− 2.1782	4.872426	− 0.44705	0.654841	NA
hsa-miR-4769-5p/hsa-mir-4769	0.461733	0.923467	0	− 2.09228	4.877731	− 0.42895	0.667962	NA
hsa-miR-4770/hsa-mir-4770	0	0	0	NA	NA	NA	NA	NA
hsa-miR-4771/hsa-mir-4771–1	0	0	0	NA	NA	NA	NA	NA
hsa-miR-4771/hsa-mir-4771–2	0	0	0	NA	NA	NA	NA	NA
hsa-miR-4772-3p/hsa-mir-4772	0.153911	0.307822	0	− 0.74599	4.97963	− 0.14981	0.880917	NA
hsa-miR-4772-5p/hsa-mir-4772	0.153911	0.307822	0	− 0.74599	4.97963	− 0.14981	0.880917	NA
hsa-miR-4773/hsa-mir-4773–1	1.077378	2.154756	0	− 3.29934	4.82535	− 0.68375	0.494131	NA
hsa-miR-4773/hsa-mir-4773–2	1.077378	2.154756	0	− 3.29934	4.82535	− 0.68375	0.494131	NA
hsa-miR-4774-3p/hsa-mir-4774	0	0	0	NA	NA	NA	NA	NA
hsa-miR-4774-5p/hsa-mir-4774	1.345704	1.634036	1.057371	− 0.40128	3.710507	− 0.10815	0.913879	NA
hsa-miR-4776-3p/hsa-mir-4776–1	0	0	0	NA	NA	NA	NA	NA
hsa-miR-4776-3p/hsa-mir-4776–2	0	0	0	NA	NA	NA	NA	NA
hsa-miR-4776-5p/hsa-mir-4776–1	0.132171	0	0.264343	1.823505	4.944331	0.368807	0.712271	NA
hsa-miR-4776-5p/hsa-mir-4776–2	0.132171	0	0.264343	1.823505	4.944331	0.368807	0.712271	NA
hsa-miR-4777-3p/hsa-mir-4777	0	0	0	NA	NA	NA	NA	NA
hsa-miR-4777-5p/hsa-mir-4777	0.286083	0.307822	0.264343	0.477194	4.926281	0.096867	0.922832	NA
hsa-miR-4778-3p/hsa-mir-4778	0.307822	0.615645	0	− 1.51932	4.922146	− 0.30867	0.757573	NA
hsa-miR-4778-5p/hsa-mir-4778	0	0	0	NA	NA	NA	NA	NA
hsa-miR-4779/hsa-mir-4779	0	0	0	NA	NA	NA	NA	NA
hsa-miR-4780/hsa-mir-4780	0	0	0	NA	NA	NA	NA	NA
hsa-miR-4781-3p/hsa-mir-4781	1.961348	2.865326	1.057371	− 1.19854	3.217551	− 0.3725	0.70952	NA
hsa-miR-4781-5p/hsa-mir-4781	0	0	0	NA	NA	NA	NA	NA
hsa-miR-4782-3p/hsa-mir-4782	0	0	0	NA	NA	NA	NA	NA
hsa-miR-4782-5p/hsa-mir-4782	0	0	0	NA	NA	NA	NA	NA
hsa-miR-4783-5p/hsa-mir-4783	0.132171	0	0.264343	1.823505	4.944331	0.368807	0.712271	NA
hsa-miR-4784/hsa-mir-4784	0.307822	0.615645	0	− 1.51932	4.922146	− 0.30867	0.757573	NA
hsa-miR-4785/hsa-mir-4785	1.953336	1.527587	2.379085	0.766267	3.606779	0.212452	0.831755	NA
hsa-miR-4786-3p/hsa-mir-4786	0.254598	0.509196	0	− 1.11434	4.959133	− 0.2247	0.82221	NA
hsa-miR-4786-5p/hsa-mir-4786	0.769556	1.539112	0	− 2.81872	4.841262	− 0.58223	0.560413	NA
hsa-miR-4787-5p/hsa-mir-4787	0	0	0	NA	NA	NA	NA	NA
hsa-miR-4788/hsa-mir-4788	0.307822	0.615645	0	− 1.51932	4.922146	− 0.30867	0.757573	NA
hsa-miR-4789-3p/hsa-mir-4789	0.681477	0	1.362953	3.012055	4.910469	0.613395	0.539615	NA
hsa-miR-4789-5p/hsa-mir-4789	0	0	0	NA	NA	NA	NA	NA
hsa-miR-4790-3p/hsa-mir-4790	0	0	0	NA	NA	NA	NA	NA
hsa-miR-4790-5p/hsa-mir-4790	0	0	0	NA	NA	NA	NA	NA
hsa-miR-4793-3p/hsa-mir-4793	1.059621	1.326214	0.793028	− 0.3881	4.101081	− 0.09463	0.924607	NA
hsa-miR-4793-5p/hsa-mir-4793	0	0	0	NA	NA	NA	NA	NA
hsa-miR-4794/hsa-mir-4794	0.254598	0.509196	0	− 1.11434	4.959133	− 0.2247	0.82221	NA
hsa-miR-4795-3p/hsa-mir-4795	0.970929	1.941859	0	− 3.13569	4.676087	− 0.67058	0.502488	NA
hsa-miR-4795-5p/hsa-mir-4795	0.307822	0.615645	0	− 1.51932	4.922146	− 0.30867	0.757573	NA
hsa-miR-4796-3p/hsa-mir-4796	0	0	0	NA	NA	NA	NA	NA
hsa-miR-4796-5p/hsa-mir-4796	0.879987	1.231289	0.528686	− 0.70455	4.780177	− 0.14739	0.882824	NA
hsa-miR-4797-5p/hsa-mir-4797	0.593905	0.923467	0.264343	− 0.86911	4.823254	− 0.18019	0.857002	NA
hsa-miR-4798-3p/hsa-mir-4798	0	0	0	NA	NA	NA	NA	NA
hsa-miR-4799-3p/hsa-mir-4799	0	0	0	NA	NA	NA	NA	NA
hsa-miR-4799-5p/hsa-mir-4799	0	0	0	NA	NA	NA	NA	NA
hsa-miR-4800-3p/hsa-mir-4800	0.153911	0.307822	0	− 0.74599	4.97963	− 0.14981	0.880917	NA
hsa-miR-4800-5p/hsa-mir-4800	0.153911	0.307822	0	− 0.74599	4.97963	− 0.14981	0.880917	NA
hsa-miR-4801/hsa-mir-4801	0	0	0	NA	NA	NA	NA	NA
hsa-miR-4802-3p/hsa-mir-4802	1.794322	1.432663	2.155982	0.533264	3.07491	0.173424	0.862318	NA
hsa-miR-4803/hsa-mir-4803	5.487577	10.97515	0	− 5.6437	2.798866	− 2.01642	0.043756	NA
hsa-miR-4804-3p/hsa-mir-4804	2.861955	4.096615	1.627296	− 1.54195	2.705418	− 0.56995	0.568713	NA
hsa-miR-483-3p/hsa-mir-483	0.641367	1.018392	0.264343	− 0.95503	4.817889	− 0.19822	0.842869	NA
hsa-miR-483-5p/hsa-mir-483	0	0	0	NA	NA	NA	NA	NA
hsa-miR-485-3p/hsa-mir-485	5.768597	3.078223	8.45897	1.486274	2.565316	0.579373	0.562338	NA
hsa-miR-485-5p/hsa-mir-485	0	0	0	NA	NA	NA	NA	NA
hsa-miR-486-3p/hsa-mir-486-1	1.266757	1.740485	0.793028	− 0.79738	3.810955	− 0.20923	0.834267	NA
hsa-miR-486-3p/hsa-mir-486-2	0.439994	0.615645	0.264343	− 0.29614	4.868166	− 0.06083	0.951493	NA
hsa-miR-487a-3p/hsa-mir-487a	0.132171	0	0.264343	1.823505	4.944331	0.368807	0.712271	NA
hsa-miR-487a-5p/hsa-mir-487a	0.286083	0.307822	0.264343	0.477194	4.926281	0.096867	0.922832	NA
hsa-miR-487b-3p/hsa-mir-487b	1.540844	1.231289	1.8504	0.659801	3.90167	0.169107	0.865712	NA
hsa-miR-487b-5p/hsa-mir-487b	0	0	0	NA	NA	NA	NA	NA
hsa-miR-488-5p/hsa-mir-488	1.954466	2.545979	1.362953	− 1.21733	3.756351	− 0.32407	0.745884	NA
hsa-miR-489-5p/hsa-mir-489	0	0	0	NA	NA	NA	NA	NA
hsa-miR-490-3p/hsa-mir-490	0.615645	1.231289	0	− 2.50085	4.855042	− 0.5151	0.606481	NA
hsa-miR-490-5p/hsa-mir-490	0.307822	0.615645	0	− 1.51932	4.922146	− 0.30867	0.757573	NA
hsa-miR-492/hsa-mir-492	1.077991	0	2.155982	3.866341	4.539122	0.851782	0.394335	NA
hsa-miR-493-3p/hsa-mir-493	0.615645	1.231289	0	− 2.50085	4.855042	− 0.5151	0.606481	NA
hsa-miR-493-5p/hsa-mir-493	0.307822	0.615645	0	− 1.51932	4.922146	− 0.30867	0.757573	NA
hsa-miR-494-5p/hsa-mir-494	0	0	0	NA	NA	NA	NA	NA
hsa-miR-495-3p/hsa-mir-495	1.739968	0.307822	3.172114	3.165097	3.833738	0.82559	0.409037	NA
hsa-miR-495-5p/hsa-mir-495	0.704337	0.615645	0.793028	0.648104	4.838855	0.133937	0.893452	NA
hsa-miR-496/hsa-mir-496	1.057371	0	2.114743	3.925076	4.861599	0.807363	0.419457	NA
hsa-miR-498-3p/hsa-mir-498	0	0	0	NA	NA	NA	NA	NA
hsa-miR-498-5p/hsa-mir-498	0.660857	0	1.321714	3.278941	4.90256	0.668822	0.503609	NA
hsa-miR-4999-3p/hsa-mir-4999	1.434396	1.018392	1.8504	0.997068	4.048294	0.246293	0.805455	NA
hsa-miR-499a-3p/hsa-mir-499a	0	0	0	NA	NA	NA	NA	NA
hsa-miR-499b-3p/hsa-mir-499b	0.509196	1.018392	0	− 2.1782	4.872426	− 0.44705	0.654841	NA
hsa-miR-5000-5p/hsa-mir-5000	0.572165	0.615645	0.528686	0.276985	4.848317	0.05713	0.954442	NA
hsa-miR-5001-5p/hsa-mir-5001	0.835388	0.307822	1.362953	1.665763	4.892293	0.340487	0.73349	NA
hsa-miR-5002-3p/hsa-mir-5002	0	0	0	NA	NA	NA	NA	NA
hsa-miR-5002-5p/hsa-mir-5002	0	0	0	NA	NA	NA	NA	NA
hsa-miR-5003-3p/hsa-mir-5003	0	0	0	NA	NA	NA	NA	NA
hsa-miR-5003-5p/hsa-mir-5003	1.700988	2.344605	1.057371	− 0.89936	3.451975	− 0.26054	0.79445	NA
hsa-miR-5004-3p/hsa-mir-5004	0	0	0	NA	NA	NA	NA	NA
hsa-miR-5004-5p/hsa-mir-5004	0.660857	0	1.321714	3.278941	4.90256	0.668822	0.503609	NA
hsa-miR-5006-3p/hsa-mir-5006	0.528686	0	1.057371	3.044078	4.909871	0.619992	0.535263	NA
hsa-miR-5006-5p/hsa-mir-5006	0.132171	0	0.264343	1.823505	4.944331	0.368807	0.712271	NA
hsa-miR-5007-3p/hsa-mir-5007	0.264343	0	0.528686	2.396631	4.924788	0.486646	0.626509	NA
hsa-miR-5007-5p/hsa-mir-5007	0.153911	0.307822	0	− 0.74599	4.97963	− 0.14981	0.880917	NA
hsa-miR-5008-3p/hsa-mir-5008	0.681477	0	1.362953	3.012055	4.910469	0.613395	0.539615	NA
hsa-miR-5009-3p/hsa-mir-5009	0.307822	0.615645	0	− 1.51932	4.922146	− 0.30867	0.757573	NA
hsa-miR-5010-5p/hsa-mir-5010	1.987071	2.652428	1.321714	− 0.80833	3.210321	− 0.25179	0.801202	NA
hsa-miR-5011-3p/hsa-mir-5011	0	0	0	NA	NA	NA	NA	NA
hsa-miR-5011-5p/hsa-mir-5011	0	0	0	NA	NA	NA	NA	NA
hsa-miR-504-3p/hsa-mir-504	0.153911	0.307822	0	− 0.74599	4.97963	− 0.14981	0.880917	NA
hsa-miR-5047/hsa-mir-5047	0	0	0	NA	NA	NA	NA	NA
hsa-miR-506-3p/hsa-mir-506	0.153911	0.307822	0	− 0.74599	4.97963	− 0.14981	0.880917	NA
hsa-miR-506-5p/hsa-mir-506	0.132171	0	0.264343	1.823505	4.944331	0.368807	0.712271	NA
hsa-miR-507/hsa-mir-507	0.286083	0.307822	0.264343	0.477194	4.926281	0.096867	0.922832	NA
hsa-miR-508-3p/hsa-mir-508	3.916464	4.925157	2.907771	− 0.65872	2.862542	− 0.23012	0.818001	NA
hsa-miR-508-5p/hsa-mir-508	0.264343	0	0.528686	2.396631	4.924788	0.486646	0.626509	NA
hsa-miR-5087/hsa-mir-5087	0.747816	1.231289	0.264343	− 1.27767	4.800308	− 0.26616	0.790112	NA
hsa-miR-5088-3p/hsa-mir-5088	0.572165	0.615645	0.528686	0.276985	4.848317	0.05713	0.954442	NA
hsa-miR-5088-5p/hsa-mir-5088	0.572165	0.615645	0.528686	0.276985	4.848317	0.05713	0.954442	NA
hsa-miR-5089-3p/hsa-mir-5089	0.509196	1.018392	0	− 2.1782	4.872426	− 0.44705	0.654841	NA
hsa-miR-5089-5p/hsa-mir-5089	0.509196	1.018392	0	− 2.1782	4.872426	− 0.44705	0.654841	NA
hsa-miR-5090/hsa-mir-5090	0.835388	0.307822	1.362953	1.665763	4.892293	0.340487	0.73349	NA
hsa-miR-5091/hsa-mir-5091	4.433635	5.612678	3.254592	− 0.91704	2.266717	− 0.40457	0.685796	NA
hsa-miR-5092/hsa-mir-5092	0	0	0	NA	NA	NA	NA	NA
hsa-miR-509-3-5p/hsa-mir-509-3	0	0	0	NA	NA	NA	NA	NA
hsa-miR-5093/hsa-mir-5093	0	0	0	NA	NA	NA	NA	NA
hsa-miR-509-3p/hsa-mir-509-1	4.076608	6.038473	2.114743	− 1.32884	2.465041	− 0.53907	0.589837	NA
hsa-miR-509-3p/hsa-mir-509-2	4.076608	6.038473	2.114743	− 1.32884	2.465041	− 0.53907	0.589837	NA
hsa-miR-509-3p/hsa-mir-509-3	4.076608	6.038473	2.114743	− 1.32884	2.465041	− 0.53907	0.589837	NA
hsa-miR-509-5p/hsa-mir-509-1	0.682597	0.307822	1.057371	1.697766	4.891693	0.347071	0.728538	NA
hsa-miR-509-5p/hsa-mir-509-2	0.682597	0.307822	1.057371	1.697766	4.891693	0.347071	0.728538	NA
hsa-miR-510-3p/hsa-mir-510	0	0	0	NA	NA	NA	NA	NA
hsa-miR-510-5p/hsa-mir-510	0	0	0	NA	NA	NA	NA	NA
hsa-miR-511-3p/hsa-mir-511	0	0	0	NA	NA	NA	NA	NA
hsa-miR-511-5p/hsa-mir-511	0.286083	0.307822	0.264343	0.477194	4.926281	0.096867	0.922832	NA
hsa-miR-512-3p/hsa-mir-512-1	1.189543	0	2.379085	4.089116	4.831661	0.846317	0.397376	NA
hsa-miR-512-3p/hsa-mir-512-2	1.189543	0	2.379085	4.089116	4.831661	0.846317	0.397376	NA
hsa-miR-512-5p/hsa-mir-512-1	0.396514	0	0.793028	2.767752	4.915475	0.563069	0.573388	NA
hsa-miR-512-5p/hsa-mir-512-2	0.396514	0	0.793028	2.767752	4.915475	0.563069	0.573388	NA
hsa-miR-513a-3p/hsa-mir-513a-1	0	0	0	NA	NA	NA	NA	NA
hsa-miR-513a-3p/hsa-mir-513a-2	0	0	0	NA	NA	NA	NA	NA
hsa-miR-513a-5p/hsa-mir-513a-1	0.660857	0	1.321714	3.278941	4.90256	0.668822	0.503609	NA
hsa-miR-513a-5p/hsa-mir-513a-2	0.660857	0	1.321714	3.278941	4.90256	0.668822	0.503609	NA
hsa-miR-513b-3p/hsa-mir-513b	0.153911	0.307822	0	− 0.74599	4.97963	− 0.14981	0.880917	NA
hsa-miR-513b-5p/hsa-mir-513b	0.132171	0	0.264343	1.823505	4.944331	0.368807	0.712271	NA
hsa-miR-513c-3p/hsa-mir-513c	0.153911	0.307822	0	− 0.74599	4.97963	− 0.14981	0.880917	NA
hsa-miR-513c-5p/hsa-mir-513c	2.377352	1.846934	2.907771	0.717875	3.330726	0.215531	0.829353	NA
hsa-miR-514a-5p/hsa-mir-514a-1	1.364073	0.307822	2.420325	2.68904	3.810815	0.705634	0.480416	NA
hsa-miR-514a-5p/hsa-mir-514a-2	1.364073	0.307822	2.420325	2.68904	3.810815	0.705634	0.480416	NA
hsa-miR-514a-5p/hsa-mir-514a-3	1.364073	0.307822	2.420325	2.68904	3.810815	0.705634	0.480416	NA
hsa-miR-514b-3p/hsa-mir-514b	0	0	0	NA	NA	NA	NA	NA
hsa-miR-514b-5p/hsa-mir-514b	0	0	0	NA	NA	NA	NA	NA
hsa-miR-515-3p/hsa-mir-515-1	4.119053	0.307822	7.930284	4.460097	2.979138	1.49711	0.134365	NA
hsa-miR-515-3p/hsa-mir-515-2	4.119053	0.307822	7.930284	4.460097	2.979138	1.49711	0.134365	NA
hsa-miR-516a-3p/hsa-mir-516a-1	0	0	0	NA	NA	NA	NA	NA
hsa-miR-516a-3p/hsa-mir-516a-2	0	0	0	NA	NA	NA	NA	NA
hsa-miR-516a-3p/hsa-mir-516b-1	0	0	0	NA	NA	NA	NA	NA
hsa-miR-516a-3p/hsa-mir-516b-2	0	0	0	NA	NA	NA	NA	NA
hsa-miR-516b-3p/hsa-mir-516a-1	0	0	0	NA	NA	NA	NA	NA
hsa-miR-516b-3p/hsa-mir-516a-2	0	0	0	NA	NA	NA	NA	NA
hsa-miR-516b-3p/hsa-mir-516b-1	0	0	0	NA	NA	NA	NA	NA
hsa-miR-516b-3p/hsa-mir-516b-2	0	0	0	NA	NA	NA	NA	NA
hsa-miR-516b-5p/hsa-mir-516b-1	2.96963	0	5.93926	5.323423	3.158305	1.685532	0.091886	NA
hsa-miR-516b-5p/hsa-mir-516b-2	1.077991	0	2.155982	3.866341	4.539122	0.851782	0.394335	NA
hsa-miR-517-5p/hsa-mir-517a	0.264343	0	0.528686	2.396631	4.924788	0.486646	0.626509	NA
hsa-miR-517-5p/hsa-mir-517b	0.264343	0	0.528686	2.396631	4.924788	0.486646	0.626509	NA
hsa-miR-517-5p/hsa-mir-517c	0.264343	0	0.528686	2.396631	4.924788	0.486646	0.626509	NA
hsa-miR-517c-3p/hsa-mir-517c	5.673626	0.509196	10.83806	4.667309	2.773304	1.682942	0.092386	NA
hsa-miR-5186/hsa-mir-5186	0	0	0	NA	NA	NA	NA	NA
hsa-miR-5187-3p/hsa-mir-5187	4.143053	2.652428	5.633678	1.114946	2.252819	0.494911	0.620663	NA
hsa-miR-5188/hsa-mir-5188	0.858248	0.923467	0.793028	0.075134	4.793669	0.015673	0.987495	NA
hsa-miR-5189-3p/hsa-mir-5189	0.254598	0.509196	0	− 1.11434	4.959133	− 0.2247	0.82221	NA
hsa-miR-5189-5p/hsa-mir-5189	0	0	0	NA	NA	NA	NA	NA
hsa-miR-518a-3p/hsa-mir-518a-1	0.528686	0	1.057371	3.044078	4.909871	0.619992	0.535263	NA
hsa-miR-518a-3p/hsa-mir-518a-2	0.528686	0	1.057371	3.044078	4.909871	0.619992	0.535263	NA
hsa-miR-518a-5p/hsa-mir-518a-1	2.136482	0.307822	3.965142	3.480727	3.58348	0.971326	0.331386	NA
hsa-miR-518a-5p/hsa-mir-518a-2	2.136482	0.307822	3.965142	3.480727	3.58348	0.971326	0.331386	NA
hsa-miR-518a-5p/hsa-mir-527	2.136482	0.307822	3.965142	3.480727	3.58348	0.971326	0.331386	NA
hsa-miR-518c-5p/hsa-mir-518c	0.396514	0	0.793028	2.767752	4.915475	0.563069	0.573388	NA
hsa-miR-518d-3p/hsa-mir-518d	0	0	0	NA	NA	NA	NA	NA
hsa-miR-518d-5p/hsa-mir-518d	2.399705	0	4.79941	5.071506	3.401346	1.491029	0.135954	NA
hsa-miR-518d-5p/hsa-mir-520c	2.399705	0	4.79941	5.071506	3.401346	1.491029	0.135954	NA
hsa-miR-518d-5p/hsa-mir-526a-1	2.399705	0	4.79941	5.071506	3.401346	1.491029	0.135954	NA
hsa-miR-518d-5p/hsa-mir-526a-2	2.399705	0	4.79941	5.071506	3.401346	1.491029	0.135954	NA
hsa-miR-518e-3p/hsa-mir-518e	0.264343	0	0.528686	2.396631	4.924788	0.486646	0.626509	NA
hsa-miR-518e-5p/hsa-mir-518e	4.293584	0.615645	7.971524	3.628842	2.599303	1.396083	0.16269	NA
hsa-miR-518e-5p/hsa-mir-519b	4.293584	0.615645	7.971524	3.628842	2.599303	1.396083	0.16269	NA
hsa-miR-518e-5p/hsa-mir-519c	4.293584	0.615645	7.971524	3.628842	2.599303	1.396083	0.16269	NA
hsa-miR-518e-5p/hsa-mir-522	4.293584	0.615645	7.971524	3.628842	2.599303	1.396083	0.16269	NA
hsa-miR-518e-5p/hsa-mir-523	4.293584	0.615645	7.971524	3.628842	2.599303	1.396083	0.16269	NA
hsa-miR-518f-5p/hsa-mir-518f	2.664048	0	5.328096	5.225204	3.293241	1.586645	0.112593	NA
hsa-miR-5190/hsa-mir-5190	0	0	0	NA	NA	NA	NA	NA
hsa-miR-5191/hsa-mir-5191	0	0	0	NA	NA	NA	NA	NA
hsa-miR-5192/hsa-mir-5192	0	0	0	NA	NA	NA	NA	NA
hsa-miR-5193/hsa-mir-5193	0	0	0	NA	NA	NA	NA	NA
hsa-miR-5194/hsa-mir-5194	0	0	0	NA	NA	NA	NA	NA
hsa-miR-5195-3p/hsa-mir-5195	0	0	0	NA	NA	NA	NA	NA
hsa-miR-5195-5p/hsa-mir-5195	0	0	0	NA	NA	NA	NA	NA
hsa-miR-5196-3p/hsa-mir-5196	0.763794	1.527587	0	− 2.77189	4.84311	− 0.57234	0.567094	NA
hsa-miR-5196-5p/hsa-mir-5196	0	0	0	NA	NA	NA	NA	NA
hsa-miR-5197-3p/hsa-mir-5197	0	0	0	NA	NA	NA	NA	NA
hsa-miR-5197-5p/hsa-mir-5197	0	0	0	NA	NA	NA	NA	NA
hsa-miR-519a-2-5p/hsa-mir-519a-2	0.814768	0.307822	1.321714	1.93264	4.884354	0.39568	0.692341	NA
hsa-miR-519a-2-5p/hsa-mir-520b	0.814768	0.307822	1.321714	1.93264	4.884354	0.39568	0.692341	NA
hsa-miR-519a-3p/hsa-mir-519a-1	5.981467	2.545979	9.416956	1.92256	2.272991	0.845828	0.397648	NA
hsa-miR-519a-3p/hsa-mir-519a-2	5.981467	2.545979	9.416956	1.92256	2.272991	0.845828	0.397648	NA
hsa-miR-519a-5p/hsa-mir-518e	4.293584	0.615645	7.971524	3.628842	2.599303	1.396083	0.16269	NA
hsa-miR-519a-5p/hsa-mir-519b	4.293584	0.615645	7.971524	3.628842	2.599303	1.396083	0.16269	NA
hsa-miR-519a-5p/hsa-mir-519c	4.293584	0.615645	7.971524	3.628842	2.599303	1.396083	0.16269	NA
hsa-miR-519a-5p/hsa-mir-522	4.293584	0.615645	7.971524	3.628842	2.599303	1.396083	0.16269	NA
hsa-miR-519a-5p/hsa-mir-523	4.293584	0.615645	7.971524	3.628842	2.599303	1.396083	0.16269	NA
hsa-miR-519b-3p/hsa-mir-519b	1.211282	0.307822	2.114743	2.591299	4.361129	0.594181	0.552391	NA
hsa-miR-519b-5p/hsa-mir-518e	4.293584	0.615645	7.971524	3.628842	2.599303	1.396083	0.16269	NA
hsa-miR-519b-5p/hsa-mir-519b	4.293584	0.615645	7.971524	3.628842	2.599303	1.396083	0.16269	NA
hsa-miR-519b-5p/hsa-mir-519c	4.293584	0.615645	7.971524	3.628842	2.599303	1.396083	0.16269	NA
hsa-miR-519b-5p/hsa-mir-522	4.293584	0.615645	7.971524	3.628842	2.599303	1.396083	0.16269	NA
hsa-miR-519b-5p/hsa-mir-523	4.293584	0.615645	7.971524	3.628842	2.599303	1.396083	0.16269	NA
hsa-miR-519c-3p/hsa-mir-519c	4.892592	1.326214	8.45897	2.774443	2.493044	1.112874	0.265763	NA
hsa-miR-519c-5p/hsa-mir-518e	4.293584	0.615645	7.971524	3.628842	2.599303	1.396083	0.16269	NA
hsa-miR-519c-5p/hsa-mir-519b	4.293584	0.615645	7.971524	3.628842	2.599303	1.396083	0.16269	NA
hsa-miR-519c-5p/hsa-mir-519c	4.293584	0.615645	7.971524	3.628842	2.599303	1.396083	0.16269	NA
hsa-miR-519c-5p/hsa-mir-522	4.293584	0.615645	7.971524	3.628842	2.599303	1.396083	0.16269	NA
hsa-miR-519c-5p/hsa-mir-523	4.293584	0.615645	7.971524	3.628842	2.599303	1.396083	0.16269	NA
hsa-miR-519d-3p/hsa-mir-519d	4.383396	0.307822	8.45897	4.551232	2.93481	1.550776	0.120955	NA
hsa-miR-519d-5p/hsa-mir-519d	0.814768	0.307822	1.321714	1.93264	4.884354	0.39568	0.692341	NA
hsa-miR-519e-3p/hsa-mir-519e	0.132171	0	0.264343	1.823505	4.944331	0.368807	0.712271	NA
hsa-miR-519e-5p/hsa-mir-519e	2.289273	0.307822	4.270724	3.546249	3.202827	1.107225	0.268197	NA
hsa-miR-520a-3p/hsa-mir-520a	0	0	0	NA	NA	NA	NA	NA
hsa-miR-520a-5p/hsa-mir-520a	1.321714	0	2.643428	4.240271	4.639562	0.913938	0.36075	NA
hsa-miR-520b-3p/hsa-mir-520b	0.9252	0	1.8504	3.740436	4.871648	0.767797	0.442608	NA
hsa-miR-520b-3p/hsa-mir-520c	1.321714	0	2.643428	4.240271	4.639562	0.913938	0.36075	NA
hsa-miR-520b-5p/hsa-mir-519a-2	0.814768	0.307822	1.321714	1.93264	4.884354	0.39568	0.692341	NA
hsa-miR-520b-5p/hsa-mir-520b	0.814768	0.307822	1.321714	1.93264	4.884354	0.39568	0.692341	NA
hsa-miR-520c-3p/hsa-mir-520c	1.321714	0	2.643428	4.240271	4.639562	0.913938	0.36075	NA
hsa-miR-520c-5p/hsa-mir-518d	2.399705	0	4.79941	5.071506	3.401346	1.491029	0.135954	NA
hsa-miR-520c-5p/hsa-mir-520c	2.399705	0	4.79941	5.071506	3.401346	1.491029	0.135954	NA
hsa-miR-520c-5p/hsa-mir-526a-1	2.399705	0	4.79941	5.071506	3.401346	1.491029	0.135954	NA
hsa-miR-520c-5p/hsa-mir-526a-2	2.399705	0	4.79941	5.071506	3.401346	1.491029	0.135954	NA
hsa-miR-520d-3p/hsa-mir-520d	0.132171	0	0.264343	1.823505	4.944331	0.368807	0.712271	NA
hsa-miR-520d-5p/hsa-mir-520d	1.321714	0	2.643428	4.240271	4.639562	0.913938	0.36075	NA
hsa-miR-520e-3p/hsa-mir-520e	0	0	0	NA	NA	NA	NA	NA
hsa-miR-520e-5p/hsa-mir-520e	0	0	0	NA	NA	NA	NA	NA
hsa-miR-520f-3p/hsa-mir-520c	1.321714	0	2.643428	4.240271	4.639562	0.913938	0.36075	NA
hsa-miR-520f-5p/hsa-mir-520f	0.264343	0	0.528686	2.396631	4.924788	0.486646	0.626509	NA
hsa-miR-520 g-3p/hsa-mir-520 g	4.80165	0.615645	8.987656	3.820002	2.77031	1.378908	0.167923	NA
hsa-miR-520 g-5p/hsa-mir-520 g	1.057371	0	2.114743	3.925076	4.861599	0.807363	0.419457	NA
hsa-miR-520 g-5p/hsa-mir-520 h	1.057371	0	2.114743	3.925076	4.861599	0.807363	0.419457	NA
hsa-miR-520 h/hsa-mir-520 g	4.80165	0.615645	8.987656	3.820002	2.77031	1.378908	0.167923	NA
hsa-miR-520 h/hsa-mir-520 h	0.528686	0	1.057371	3.044078	4.909871	0.619992	0.535263	NA
hsa-miR-521/hsa-mir-521–1	1.444141	0.509196	2.379085	2.424778	4.098046	0.591691	0.554057	NA
hsa-miR-521/hsa-mir-521–2	1.444141	0.509196	2.379085	2.424778	4.098046	0.591691	0.554057	NA
hsa-miR-522-3p/hsa-mir-522	1.972826	0.509196	3.436457	2.968116	3.682127	0.806087	0.420193	NA
hsa-miR-522-5p/hsa-mir-518e	4.293584	0.615645	7.971524	3.628842	2.599303	1.396083	0.16269	NA
hsa-miR-522-5p/hsa-mir-519b	4.293584	0.615645	7.971524	3.628842	2.599303	1.396083	0.16269	NA
hsa-miR-522-5p/hsa-mir-519c	4.293584	0.615645	7.971524	3.628842	2.599303	1.396083	0.16269	NA
hsa-miR-522-5p/hsa-mir-522	4.293584	0.615645	7.971524	3.628842	2.599303	1.396083	0.16269	NA
hsa-miR-522-5p/hsa-mir-523	4.293584	0.615645	7.971524	3.628842	2.599303	1.396083	0.16269	NA
hsa-miR-523-3p/hsa-mir-523	0.396514	0	0.793028	2.767752	4.915475	0.563069	0.573388	NA
hsa-miR-523-5p/hsa-mir-518e	4.293584	0.615645	7.971524	3.628842	2.599303	1.396083	0.16269	NA
hsa-miR-523-5p/hsa-mir-519b	4.293584	0.615645	7.971524	3.628842	2.599303	1.396083	0.16269	NA
hsa-miR-523-5p/hsa-mir-519c	4.293584	0.615645	7.971524	3.628842	2.599303	1.396083	0.16269	NA
hsa-miR-523-5p/hsa-mir-522	4.293584	0.615645	7.971524	3.628842	2.599303	1.396083	0.16269	NA
hsa-miR-523-5p/hsa-mir-523	4.293584	0.615645	7.971524	3.628842	2.599303	1.396083	0.16269	NA
hsa-miR-524-3p/hsa-mir-524	0.132171	0	0.264343	1.823505	4.944331	0.368807	0.712271	NA
hsa-miR-524-5p/hsa-mir-524	2.501512	0.509196	4.493828	3.365644	3.41628	0.985178	0.324537	NA
hsa-miR-525-3p/hsa-mir-525	0.264343	0	0.528686	2.396631	4.924788	0.486646	0.626509	NA
hsa-miR-525-5p/hsa-mir-525	2.523252	0.817018	4.229485	2.47312	3.052447	0.810209	0.41782	NA
hsa-miR-526a-3p/hsa-mir-526a-1	0	0	0	NA	NA	NA	NA	NA
hsa-miR-526a-5p/hsa-mir-518d	2.399705	0	4.79941	5.071506	3.401346	1.491029	0.135954	NA
hsa-miR-526a-5p/hsa-mir-520c	2.399705	0	4.79941	5.071506	3.401346	1.491029	0.135954	NA
hsa-miR-526a-5p/hsa-mir-526a-1	2.399705	0	4.79941	5.071506	3.401346	1.491029	0.135954	NA
hsa-miR-526a-5p/hsa-mir-526a-2	2.399705	0	4.79941	5.071506	3.401346	1.491029	0.135954	NA
hsa-miR-526b-3p/hsa-mir-526b	0.9252	0	1.8504	3.740436	4.871648	0.767797	0.442608	NA
hsa-miR-527/hsa-mir-518a-1	2.136482	0.307822	3.965142	3.480727	3.58348	0.971326	0.331386	NA
hsa-miR-527/hsa-mir-518a-2	2.136482	0.307822	3.965142	3.480727	3.58348	0.971326	0.331386	NA
hsa-miR-527/hsa-mir-527	2.136482	0.307822	3.965142	3.480727	3.58348	0.971326	0.331386	NA
hsa-miR-539-3p/hsa-mir-539	4.294704	0.923467	7.665942	3.0384	2.79982	1.085213	0.277827	NA
hsa-miR-539-5p/hsa-mir-539	0	0	0	NA	NA	NA	NA	NA
hsa-miR-541-3p/hsa-mir-541	0	0	0	NA	NA	NA	NA	NA
hsa-miR-541-5p/hsa-mir-541	0	0	0	NA	NA	NA	NA	NA
hsa-miR-543/hsa-mir-543	1.321714	0	2.643428	4.240271	4.639562	0.913938	0.36075	NA
hsa-miR-544a/hsa-mir-544a	0.132171	0	0.264343	1.823505	4.944331	0.368807	0.712271	NA
hsa-miR-544b/hsa-mir-544b	1.3852	2.770401	0	− 3.6603	4.585312	− 0.79827	0.424716	NA
hsa-miR-548ac/hsa-mir-548ac	1.272989	2.545979	0	− 3.51573	4.761337	− 0.73839	0.460277	NA
hsa-miR-548ad-3p/hsa-mir-548ad	0.763794	1.527587	0	− 2.77189	4.84311	− 0.57234	0.567094	NA
hsa-miR-548ae-3p/hsa-mir-548ae-1	1.588307	1.326214	1.8504	0.625567	3.510726	0.178187	0.858576	NA
hsa-miR-548ae-3p/hsa-mir-548ae-2	1.588307	1.326214	1.8504	0.625567	3.510726	0.178187	0.858576	NA
hsa-miR-548ag/hsa-mir-548ag-1	0.56242	1.12484	0	− 2.35102	4.862644	− 0.48349	0.62875	NA
hsa-miR-548ag/hsa-mir-548ag-2	1.687261	3.374521	0	− 3.93506	3.7827	− 1.04028	0.298211	NA
hsa-miR-548ah-3p/hsa-mir-548ah	3.478203	2.462579	4.493828	0.918052	2.943498	0.311891	0.755123	NA
hsa-miR-548ah-3p/hsa-mir-548p	4.273482	3.788792	4.758171	0.417633	2.457359	0.169952	0.865048	NA
hsa-miR-548ah-5p/hsa-mir-548ah	2.311013	0.615645	4.006382	2.646138	3.107344	0.851576	0.39445	NA
hsa-miR-548ai/hsa-mir-548ai	0.528686	0	1.057371	3.044078	4.909871	0.619992	0.535263	NA
hsa-miR-548ai/hsa-mir-570	0.528686	0	1.057371	3.044078	4.909871	0.619992	0.535263	NA
hsa-miR-548aj-3p/hsa-mir-548aj-1	4.414182	8.299678	0.528686	− 3.38698	2.538286	− 1.33436	0.182086	NA
hsa-miR-548aj-3p/hsa-mir-548aj-2	4.414182	8.299678	0.528686	− 3.38698	2.538286	− 1.33436	0.182086	NA
hsa-miR-548al/hsa-mir-548al	2.06841	0.923467	3.213353	1.756191	3.18943	0.550629	0.581888	NA
hsa-miR-548am-3p/hsa-mir-548am	2.389347	2.663952	2.114743	− 0.20752	3.009242	− 0.06896	0.945022	NA
hsa-miR-548an/hsa-mir-548an	0.153911	0.307822	0	− 0.74599	4.97963	− 0.14981	0.880917	NA
hsa-miR-548ao-3p/hsa-mir-548ao	3.123436	4.925157	1.321714	− 1.72052	3.106506	− 0.55384	0.579685	NA
hsa-miR-548ao-5p/hsa-mir-548ao	0	0	0	NA	NA	NA	NA	NA
hsa-miR-548ap-3p/hsa-mir-548ap	0.396514	0	0.793028	2.767752	4.915475	0.563069	0.573388	NA
hsa-miR-548ap-5p/hsa-mir-548ap	1.730223	0.817018	2.643428	1.80718	3.446425	0.524364	0.600026	NA
hsa-miR-548ar-3p/hsa-mir-548ar	1.936239	0.923467	2.94901	1.627115	3.26659	0.498108	0.618408	NA
hsa-miR-548as-3p/hsa-mir-548as	0	0	0	NA	NA	NA	NA	NA
hsa-miR-548as-5p/hsa-mir-548as	1.408673	1.231289	1.586057	0.446623	4.040201	0.110545	0.911977	NA
hsa-miR-548at-3p/hsa-mir-548at	0.461733	0.923467	0	− 2.09228	4.877731	− 0.42895	0.667962	NA
hsa-miR-548at-5p/hsa-mir-548at	3.107929	5.422829	0.793028	− 2.37776	2.749865	− 0.86468	0.387214	NA
hsa-miR-548au-3p/hsa-mir-548au	0.264343	0	0.528686	2.396631	4.924788	0.486646	0.626509	NA
hsa-miR-548av-3p/hsa-mir-548ah	3.478203	2.462579	4.493828	0.918052	2.943498	0.311891	0.755123	NA
hsa-miR-548av-3p/hsa-mir-548av	1.452153	1.846934	1.057371	− 0.61922	4.015232	− 0.15422	0.877438	NA
hsa-miR-548av-3p/hsa-mir-548p	3.478203	2.462579	4.493828	0.918052	2.943498	0.311891	0.755123	NA
hsa-miR-548av-5p/hsa-mir-548av	0	0	0	NA	NA	NA	NA	NA
hsa-miR-548ax/hsa-mir-548ax	1.297121	1.231289	1.362953	− 0.14368	4.290063	− 0.03349	0.973283	NA
hsa-miR-548az-3p/hsa-mir-548az	0.264343	0	0.528686	2.396631	4.924788	0.486646	0.626509	NA
hsa-miR-548ba/hsa-mir-548ba	1.231289	2.462579	0	− 3.49059	4.792344	− 0.72837	0.466388	NA
hsa-miR-548bb-3p/hsa-mir-548bb	0	0	0	NA	NA	NA	NA	NA
hsa-miR-548f-3p/hsa-mir-548f-5	2.353372	0.923467	3.783278	1.926455	3.050902	0.631438	0.527754	NA
hsa-miR-548f-5p/hsa-mir-548f-1	1.794322	1.432663	2.155982	0.533264	3.07491	0.173424	0.862318	NA
hsa-miR-548 g-3p/hsa-mir-548f-5	2.353372	0.923467	3.783278	1.926455	3.050902	0.631438	0.527754	NA
hsa-miR-548 g-3p/hsa-mir-548 g	0.439994	0.615645	0.264343	− 0.29614	4.868166	− 0.06083	0.951493	NA
hsa-miR-548i/hsa-mir-548i-1	3.694434	2.853801	4.535067	0.743999	2.424025	0.306927	0.758899	NA
hsa-miR-548i/hsa-mir-548i-2	3.694434	2.853801	4.535067	0.743999	2.424025	0.306927	0.758899	NA
hsa-miR-548i/hsa-mir-548i-3	3.694434	2.853801	4.535067	0.743999	2.424025	0.306927	0.758899	NA
hsa-miR-548i/hsa-mir-548i-4	3.694434	2.853801	4.535067	0.743999	2.424025	0.306927	0.758899	NA
hsa-miR-548j-3p/hsa-mir-548j	2.608478	3.895241	1.321714	− 1.36769	2.962036	− 0.46174	0.644268	NA
hsa-miR-548 m/hsa-mir-548 m	0	0	0	NA	NA	NA	NA	NA
hsa-miR-548p/hsa-mir-548p	5.056765	4.297988	5.815542	0.52251	2.322284	0.224998	0.821981	NA
hsa-miR-548 s/hsa-mir-548 s	2.608478	3.895241	1.321714	− 1.36769	2.962036	− 0.46174	0.644268	NA
hsa-miR-548v/hsa-mir-548v	0.264343	0	0.528686	2.396631	4.924788	0.486646	0.626509	NA
hsa-miR-549a-5p/hsa-mir-549a	5.742175	11.48435	0	− 5.70864	2.714798	− 2.10279	0.035484	NA
hsa-miR-550a-3-5p/hsa-mir-550a-3	5.722896	3.268072	8.17772	1.255886	2.415201	0.519992	0.603069	NA
hsa-miR-550b-2-5p/hsa-mir-550b-1	0	0	0	NA	NA	NA	NA	NA
hsa-miR-550b-2-5p/hsa-mir-550b-2	0	0	0	NA	NA	NA	NA	NA
hsa-miR-550b-3p/hsa-mir-550b-1	0	0	0	NA	NA	NA	NA	NA
hsa-miR-550b-3p/hsa-mir-550b-2	0	0	0	NA	NA	NA	NA	NA
hsa-miR-552-3p/hsa-mir-552	0.153911	0.307822	0	− 0.74599	4.97963	− 0.14981	0.880917	NA
hsa-miR-552-5p/hsa-mir-552	0.805023	0.817018	0.793028	0.293348	4.583511	0.064001	0.94897	NA
hsa-miR-553/hsa-mir-553	0	0	0	NA	NA	NA	NA	NA
hsa-miR-554/hsa-mir-554	0	0	0	NA	NA	NA	NA	NA
hsa-miR-555/hsa-mir-555	0	0	0	NA	NA	NA	NA	NA
hsa-miR-556-5p/hsa-mir-556	5.815466	6.749043	4.881889	− 0.58519	2.040122	− 0.28684	0.774235	NA
hsa-miR-5571-3p/hsa-mir-5571	0	0	0	NA	NA	NA	NA	NA
hsa-miR-5571-5p/hsa-mir-5571	0	0	0	NA	NA	NA	NA	NA
hsa-miR-5572/hsa-mir-5572	0	0	0	NA	NA	NA	NA	NA
hsa-miR-5579-3p/hsa-mir-5579	0.264343	0	0.528686	2.396631	4.924788	0.486646	0.626509	NA
hsa-miR-5579-5p/hsa-mir-5579	0	0	0	NA	NA	NA	NA	NA
hsa-miR-557/hsa-mir-557	0	0	0	NA	NA	NA	NA	NA
hsa-miR-5580-3p/hsa-mir-5580	0.408509	0.817018	0	− 1.87945	4.892274	− 0.38417	0.700854	NA
hsa-miR-5580-5p/hsa-mir-5580	1.568817	2.344605	0.793028	− 1.19144	3.556829	− 0.33497	0.737645	NA
hsa-miR-5581-3p/hsa-mir-5581	1.333709	0.817018	1.8504	1.304328	3.778996	0.345152	0.72998	NA
hsa-miR-5581-5p/hsa-mir-5581	0.132171	0	0.264343	1.823505	4.944331	0.368807	0.712271	NA
hsa-miR-5582-5p/hsa-mir-5582	0	0	0	NA	NA	NA	NA	NA
hsa-miR-5583-5p/hsa-mir-5583-1	0.286083	0.307822	0.264343	0.477194	4.926281	0.096867	0.922832	NA
hsa-miR-5583-5p/hsa-mir-5583-2	0.286083	0.307822	0.264343	0.477194	4.926281	0.096867	0.922832	NA
hsa-miR-5584-3p/hsa-mir-5584	0	0	0	NA	NA	NA	NA	NA
hsa-miR-5584-5p/hsa-mir-5584	0.408509	0.817018	0	− 1.87945	4.892274	− 0.38417	0.700854	NA
hsa-miR-5585-3p/hsa-mir-5585	1.091106	1.12484	1.057371	0.114164	4.042909	0.028238	0.977472	NA
hsa-miR-5585-5p/hsa-mir-5585	0	0	0	NA	NA	NA	NA	NA
hsa-miR-5586-3p/hsa-mir-5586	0	0	0	NA	NA	NA	NA	NA
hsa-miR-5586-5p/hsa-mir-5586	0.153911	0.307822	0	− 0.74599	4.97963	− 0.14981	0.880917	NA
hsa-miR-5587-3p/hsa-mir-5587	0	0	0	NA	NA	NA	NA	NA
hsa-miR-5587-5p/hsa-mir-5587	0.307822	0.615645	0	− 1.51932	4.922146	− 0.30867	0.757573	NA
hsa-miR-5588-3p/hsa-mir-5588	0	0	0	NA	NA	NA	NA	NA
hsa-miR-5588-5p/hsa-mir-5588	0.793028	0	1.586057	3.52828	4.884751	0.722305	0.470107	NA
hsa-miR-5589-3p/hsa-mir-5589	0	0	0	NA	NA	NA	NA	NA
hsa-miR-5589-5p/hsa-mir-5589	0	0	0	NA	NA	NA	NA	NA
hsa-miR-558/hsa-mir-558	0	0	0	NA	NA	NA	NA	NA
hsa-miR-5590-3p/hsa-mir-5590	0	0	0	NA	NA	NA	NA	NA
hsa-miR-5590-5p/hsa-mir-5590	0	0	0	NA	NA	NA	NA	NA
hsa-miR-5591-3p/hsa-mir-5591	0.132171	0	0.264343	1.823505	4.944331	0.368807	0.712271	NA
hsa-miR-5591-5p/hsa-mir-5591	0	0	0	NA	NA	NA	NA	NA
hsa-miR-559/hsa-mir-559	1.606677	0	3.213353	4.474732	3.896794	1.148311	0.25084	NA
hsa-miR-562/hsa-mir-562	0	0	0	NA	NA	NA	NA	NA
hsa-miR-563/hsa-mir-563	0	0	0	NA	NA	NA	NA	NA
hsa-miR-564/hsa-mir-564	0	0	0	NA	NA	NA	NA	NA
hsa-miR-567/hsa-mir-567	0	0	0	NA	NA	NA	NA	NA
hsa-miR-5680/hsa-mir-5680	0.132171	0	0.264343	1.823505	4.944331	0.368807	0.712271	NA
hsa-miR-5681a/hsa-mir-5681a	0	0	0	NA	NA	NA	NA	NA
hsa-miR-5681b/hsa-mir-5681b	0	0	0	NA	NA	NA	NA	NA
hsa-miR-5682/hsa-mir-5682	1.3852	2.770401	0	− 3.6603	4.585312	− 0.79827	0.424716	NA
hsa-miR-5683/hsa-mir-5683	1.956858	0.923467	2.99025	1.549827	3.341891	0.463758	0.642821	NA
hsa-miR-5684/hsa-mir-5684	0.396514	0	0.793028	2.767752	4.915475	0.563069	0.573388	NA
hsa-miR-5685/hsa-mir-5685	0	0	0	NA	NA	NA	NA	NA
hsa-miR-5687/hsa-mir-5687	2.07403	3.883717	0.264343	− 2.91071	3.192754	− 0.91166	0.361948	NA
hsa-miR-5688/hsa-mir-5688	0	0	0	NA	NA	NA	NA	NA
hsa-miR-5689/hsa-mir-5689	0.805023	0.817018	0.793028	0.293348	4.583511	0.064001	0.94897	NA
hsa-miR-568/hsa-mir-568	0	0	0	NA	NA	NA	NA	NA
hsa-miR-5690/hsa-mir-5690	3.248725	4.605811	1.891639	− 1.41175	2.517474	− 0.56078	0.574949	NA
hsa-miR-5691/hsa-mir-5691	0	0	0	NA	NA	NA	NA	NA
hsa-miR-5692a/hsa-mir-5692a-1	0	0	0	NA	NA	NA	NA	NA
hsa-miR-5692a/hsa-mir-5692a-2	0	0	0	NA	NA	NA	NA	NA
hsa-miR-5692b/hsa-mir-5692b	0	0	0	NA	NA	NA	NA	NA
hsa-miR-5692b/hsa-mir-5692c-1	0.132171	0	0.264343	1.823505	4.944331	0.368807	0.712271	NA
hsa-miR-5692b/hsa-mir-5692c-2	0.132171	0	0.264343	1.823505	4.944331	0.368807	0.712271	NA
hsa-miR-5692c/hsa-mir-5692c-1	0.132171	0	0.264343	1.823505	4.944331	0.368807	0.712271	NA
hsa-miR-5692c/hsa-mir-5692c-2	0.132171	0	0.264343	1.823505	4.944331	0.368807	0.712271	NA
hsa-miR-5693/hsa-mir-5693	0.641367	1.018392	0.264343	− 0.95503	4.817889	− 0.19822	0.842869	NA
hsa-miR-5694/hsa-mir-5694	0	0	0	NA	NA	NA	NA	NA
hsa-miR-5695/hsa-mir-5695	3.019849	3.883717	2.155982	− 0.9086	2.529016	− 0.35927	0.719392	NA
hsa-miR-5696/hsa-mir-5696	5.715382	3.682344	7.74842	1.097053	1.996496	0.549489	0.58267	NA
hsa-miR-5697/hsa-mir-5697	2.969995	5.411304	0.528686	− 2.76901	2.809655	− 0.98553	0.324363	NA
hsa-miR-5698/hsa-mir-5698	1.3852	2.770401	0	− 3.6603	4.585312	− 0.79827	0.424716	NA
hsa-miR-5700/hsa-mir-5700	0.254598	0.509196	0	− 1.11434	4.959133	− 0.2247	0.82221	NA
hsa-miR-5702/hsa-mir-5702	4.032621	2.96025	5.104992	0.793045	2.249113	0.352603	0.724386	NA
hsa-miR-5703/hsa-mir-5703	0	0	0	NA	NA	NA	NA	NA
hsa-miR-5704/hsa-mir-5704	0	0	0	NA	NA	NA	NA	NA
hsa-miR-5705/hsa-mir-5705	0	0	0	NA	NA	NA	NA	NA
hsa-miR-570-5p/hsa-mir-548ai	0.528686	0	1.057371	3.044078	4.909871	0.619992	0.535263	NA
hsa-miR-570-5p/hsa-mir-570	0.528686	0	1.057371	3.044078	4.909871	0.619992	0.535263	NA
hsa-miR-5706/hsa-mir-5706	0	0	0	NA	NA	NA	NA	NA
hsa-miR-5707/hsa-mir-5707	0	0	0	NA	NA	NA	NA	NA
hsa-miR-5708/hsa-mir-5708	0	0	0	NA	NA	NA	NA	NA
hsa-miR-571/hsa-mir-571	0	0	0	NA	NA	NA	NA	NA
hsa-miR-572/hsa-mir-572	0	0	0	NA	NA	NA	NA	NA
hsa-miR-5739/hsa-mir-5739	0	0	0	NA	NA	NA	NA	NA
hsa-miR-575/hsa-mir-575	0	0	0	NA	NA	NA	NA	NA
hsa-miR-5787/hsa-mir-5787	0	0	0	NA	NA	NA	NA	NA
hsa-miR-578/hsa-mir-578	0.509196	1.018392	0	− 2.1782	4.872426	− 0.44705	0.654841	NA
hsa-miR-580-5p/hsa-mir-580	0.439994	0.615645	0.264343	− 0.29614	4.868166	− 0.06083	0.951493	NA
hsa-miR-581/hsa-mir-581	4.954355	6.654118	3.254592	− 1.1905	2.190514	− 0.54348	0.5868	NA
hsa-miR-583/hsa-mir-583	0	0	0	NA	NA	NA	NA	NA
hsa-miR-585-3p/hsa-mir-585	0.572165	0.615645	0.528686	0.276985	4.848317	0.05713	0.954442	NA
hsa-miR-585-5p/hsa-mir-585	0	0	0	NA	NA	NA	NA	NA
hsa-miR-586/hsa-mir-586	2.604965	5.209931	0	− 4.55798	3.256565	− 1.39963	0.161625	NA
hsa-miR-587/hsa-mir-587	0	0	0	NA	NA	NA	NA	NA
hsa-miR-588/hsa-mir-588	4.451899	8.903799	0	− 5.33714	2.784319	− 1.91686	0.055256	NA
hsa-miR-591/hsa-mir-591	0	0	0	NA	NA	NA	NA	NA
hsa-miR-592/hsa-mir-592	1.14433	1.231289	1.057371	− 0.05088	4.392583	− 0.01158	0.990758	NA
hsa-miR-593-3p/hsa-mir-593	0	0	0	NA	NA	NA	NA	NA
hsa-miR-593-5p/hsa-mir-593	0	0	0	NA	NA	NA	NA	NA
hsa-miR-595/hsa-mir-595	0	0	0	NA	NA	NA	NA	NA
hsa-miR-596/hsa-mir-596	0	0	0	NA	NA	NA	NA	NA
hsa-miR-598-5p/hsa-mir-598	0.615645	1.231289	0	− 2.50085	4.855042	− 0.5151	0.606481	NA
hsa-miR-599/hsa-mir-599	0	0	0	NA	NA	NA	NA	NA
hsa-miR-600/hsa-mir-600	0.858248	0.923467	0.793028	0.075134	4.793669	0.015673	0.987495	NA
hsa-miR-601/hsa-mir-601	0	0	0	NA	NA	NA	NA	NA
hsa-miR-602/hsa-mir-602	0.286083	0.307822	0.264343	0.477194	4.926281	0.096867	0.922832	NA
hsa-miR-603/hsa-mir-603	0	0	0	NA	NA	NA	NA	NA
hsa-miR-604/hsa-mir-604	0	0	0	NA	NA	NA	NA	NA
hsa-miR-605-3p/hsa-mir-605	0	0	0	NA	NA	NA	NA	NA
hsa-miR-605-5p/hsa-mir-605	0.286083	0.307822	0.264343	0.477194	4.926281	0.096867	0.922832	NA
hsa-miR-6068/hsa-mir-6068	0	0	0	NA	NA	NA	NA	NA
hsa-miR-6069/hsa-mir-6069	0	0	0	NA	NA	NA	NA	NA
hsa-miR-606/hsa-mir-606	0	0	0	NA	NA	NA	NA	NA
hsa-miR-6070/hsa-mir-6070	0	0	0	NA	NA	NA	NA	NA
hsa-miR-6071/hsa-mir-6071	0	0	0	NA	NA	NA	NA	NA
hsa-miR-6072/hsa-mir-6072	0	0	0	NA	NA	NA	NA	NA
hsa-miR-6073/hsa-mir-6073	0	0	0	NA	NA	NA	NA	NA
hsa-miR-6074/hsa-mir-6074	0	0	0	NA	NA	NA	NA	NA
hsa-miR-6075/hsa-mir-6075	0	0	0	NA	NA	NA	NA	NA
hsa-miR-6076/hsa-mir-6076	0	0	0	NA	NA	NA	NA	NA
hsa-miR-6077/hsa-mir-6077	0	0	0	NA	NA	NA	NA	NA
hsa-miR-6078/hsa-mir-6078	0	0	0	NA	NA	NA	NA	NA
hsa-miR-6079/hsa-mir-6079	0	0	0	NA	NA	NA	NA	NA
hsa-miR-607/hsa-mir-607	2.046057	2.770401	1.321714	− 0.92902	3.546993	− 0.26192	0.793385	NA
hsa-miR-6080/hsa-mir-6080	0	0	0	NA	NA	NA	NA	NA
hsa-miR-6081/hsa-mir-6081	0	0	0	NA	NA	NA	NA	NA
hsa-miR-6082/hsa-mir-6082	0	0	0	NA	NA	NA	NA	NA
hsa-miR-6083/hsa-mir-6083	0	0	0	NA	NA	NA	NA	NA
hsa-miR-6084/hsa-mir-6084	0	0	0	NA	NA	NA	NA	NA
hsa-miR-6085/hsa-mir-6085	0	0	0	NA	NA	NA	NA	NA
hsa-miR-6086/hsa-mir-6086	0	0	0	NA	NA	NA	NA	NA
hsa-miR-6088/hsa-mir-6088	0	0	0	NA	NA	NA	NA	NA
hsa-miR-6089/hsa-mir-6089-1	0	0	0	NA	NA	NA	NA	NA
hsa-miR-6089/hsa-mir-6089-2	0	0	0	NA	NA	NA	NA	NA
hsa-miR-608/hsa-mir-608	0	0	0	NA	NA	NA	NA	NA
hsa-miR-6090/hsa-mir-6090	0	0	0	NA	NA	NA	NA	NA
hsa-miR-609/hsa-mir-609	0.56242	1.12484	0	− 2.35102	4.862644	− 0.48349	0.62875	NA
hsa-miR-611/hsa-mir-611	0	0	0	NA	NA	NA	NA	NA
hsa-miR-6124/hsa-mir-6124	0	0	0	NA	NA	NA	NA	NA
hsa-miR-6125/hsa-mir-6125	0	0	0	NA	NA	NA	NA	NA
hsa-miR-6126/hsa-mir-6126	0	0	0	NA	NA	NA	NA	NA
hsa-miR-6127/hsa-mir-6127	0	0	0	NA	NA	NA	NA	NA
hsa-miR-6128/hsa-mir-6128	0	0	0	NA	NA	NA	NA	NA
hsa-miR-6129/hsa-mir-6129	0	0	0	NA	NA	NA	NA	NA
hsa-miR-612/hsa-mir-612	0	0	0	NA	NA	NA	NA	NA
hsa-miR-6130/hsa-mir-6130	0.461733	0.923467	0	− 2.09228	4.877731	− 0.42895	0.667962	NA
hsa-miR-6131/hsa-mir-6131	0	0	0	NA	NA	NA	NA	NA
hsa-miR-6132/hsa-mir-6132	0	0	0	NA	NA	NA	NA	NA
hsa-miR-6133/hsa-mir-6133	0	0	0	NA	NA	NA	NA	NA
hsa-miR-6134/hsa-mir-6134	0	0	0	NA	NA	NA	NA	NA
hsa-miR-613/hsa-mir-613	0	0	0	NA	NA	NA	NA	NA
hsa-miR-614/hsa-mir-614	0	0	0	NA	NA	NA	NA	NA
hsa-miR-615-5p/hsa-mir-615	5.741773	4.404437	7.07911	0.573208	2.206465	0.259786	0.795029	NA
hsa-miR-6165/hsa-mir-6165	0.264343	0	0.528686	2.396631	4.924788	0.486646	0.626509	NA
hsa-miR-617/hsa-mir-617	0	0	0	NA	NA	NA	NA	NA
hsa-miR-618/hsa-mir-618	0.663107	1.326214	0	− 2.57515	4.851549	− 0.53079	0.595564	NA
hsa-miR-619-3p/hsa-mir-619	0	0	0	NA	NA	NA	NA	NA
hsa-miR-620/hsa-mir-620	0	0	0	NA	NA	NA	NA	NA
hsa-miR-621/hsa-mir-621	0	0	0	NA	NA	NA	NA	NA
hsa-miR-622/hsa-mir-622	0.681477	0	1.362953	3.012055	4.910469	0.613395	0.539615	NA
hsa-miR-623/hsa-mir-623	0	0	0	NA	NA	NA	NA	NA
hsa-miR-626/hsa-mir-626	0	0	0	NA	NA	NA	NA	NA
hsa-miR-630/hsa-mir-630	0	0	0	NA	NA	NA	NA	NA
hsa-miR-631/hsa-mir-631	0	0	0	NA	NA	NA	NA	NA
hsa-miR-632/hsa-mir-632	0	0	0	NA	NA	NA	NA	NA
hsa-miR-633/hsa-mir-633	0	0	0	NA	NA	NA	NA	NA
hsa-miR-634/hsa-mir-634	0	0	0	NA	NA	NA	NA	NA
hsa-miR-635/hsa-mir-635	0	0	0	NA	NA	NA	NA	NA
hsa-miR-637/hsa-mir-637	0	0	0	NA	NA	NA	NA	NA
hsa-miR-638/hsa-mir-638	0	0	0	NA	NA	NA	NA	NA
hsa-miR-639/hsa-mir-639	0	0	0	NA	NA	NA	NA	NA
hsa-miR-640/hsa-mir-640	0	0	0	NA	NA	NA	NA	NA
hsa-miR-642b-3p/hsa-mir-642b	0.439994	0.615645	0.264343	− 0.29614	4.868166	− 0.06083	0.951493	NA
hsa-miR-642b-5p/hsa-mir-642b	2.837353	4.617335	1.057371	− 1.89537	3.216299	− 0.5893	0.55566	NA
hsa-miR-644a/hsa-mir-644a	0	0	0	NA	NA	NA	NA	NA
hsa-miR-645/hsa-mir-645	0	0	0	NA	NA	NA	NA	NA
hsa-miR-646/hsa-mir-646	0	0	0	NA	NA	NA	NA	NA
hsa-miR-647/hsa-mir-647	0	0	0	NA	NA	NA	NA	NA
hsa-miR-648/hsa-mir-648	0	0	0	NA	NA	NA	NA	NA
hsa-miR-6499-3p/hsa-mir-6499	0.264343	0	0.528686	2.396631	4.924788	0.486646	0.626509	NA
hsa-miR-6499-5p/hsa-mir-6499	2.643428	0	5.286856	5.233351	3.637867	1.438577	0.15027	NA
hsa-miR-649/hsa-mir-649	0	0	0	NA	NA	NA	NA	NA
hsa-miR-6500-5p/hsa-mir-6500	0	0	0	NA	NA	NA	NA	NA
hsa-miR-6501-3p/hsa-mir-6501	0.132171	0	0.264343	1.823505	4.944331	0.368807	0.712271	NA
hsa-miR-6501-5p/hsa-mir-6501	4.465627	7.873883	1.057371	− 2.58225	2.442927	− 1.05703	0.290498	NA
hsa-miR-6502-3p/hsa-mir-6502	0.254598	0.509196	0	− 1.11434	4.959133	− 0.2247	0.82221	NA
hsa-miR-6502-5p/hsa-mir-6502	1.936096	3.872193	0	− 4.12096	3.702838	− 1.11292	0.265743	NA
hsa-miR-6503-3p/hsa-mir-6503	0	0	0	NA	NA	NA	NA	NA
hsa-miR-6503-5p/hsa-mir-6503	0	0	0	NA	NA	NA	NA	NA
hsa-miR-6504-3p/hsa-mir-6504	0	0	0	NA	NA	NA	NA	NA
hsa-miR-6504-5p/hsa-mir-6504	0	0	0	NA	NA	NA	NA	NA
hsa-miR-6506-3p/hsa-mir-6506	0	0	0	NA	NA	NA	NA	NA
hsa-miR-6506-5p/hsa-mir-6506	0.848503	1.432663	0.264343	− 1.48069	4.468472	− 0.33136	0.740369	NA
hsa-miR-6507-3p/hsa-mir-6507	0	0	0	NA	NA	NA	NA	NA
hsa-miR-6507-5p/hsa-mir-6507	5.451813	0	10.90363	6.185673	3.117509	1.984172	0.047237	NA
hsa-miR-6508-3p/hsa-mir-6508	0.593905	0.923467	0.264343	− 0.86911	4.823254	− 0.18019	0.857002	NA
hsa-miR-6508-5p/hsa-mir-6508	0.681477	0	1.362953	3.012055	4.910469	0.613395	0.539615	NA
hsa-miR-6509-3p/hsa-mir-6509	0.817018	1.634036	0	− 2.88326	4.83881	− 0.59586	0.551267	NA
hsa-miR-6509-5p/hsa-mir-6509	0	0	0	NA	NA	NA	NA	NA
hsa-miR-650/hsa-mir-650	0	0	0	NA	NA	NA	NA	NA
hsa-miR-6510-5p/hsa-mir-6510	0	0	0	NA	NA	NA	NA	NA
hsa-miR-6511a-5p/hsa-mir-6511a-1	1.253642	0.615645	1.891639	1.505597	3.896978	0.38635	0.699237	NA
hsa-miR-6511a-5p/hsa-mir-6511a-2	1.253642	0.615645	1.891639	1.505597	3.896978	0.38635	0.699237	NA
hsa-miR-6511a-5p/hsa-mir-6511a-3	1.253642	0.615645	1.891639	1.505597	3.896978	0.38635	0.699237	NA
hsa-miR-6511a-5p/hsa-mir-6511a-4	1.253642	0.615645	1.891639	1.505597	3.896978	0.38635	0.699237	NA
hsa-miR-6511a-5p/hsa-mir-6511b-1	1.253642	0.615645	1.891639	1.505597	3.896978	0.38635	0.699237	NA
hsa-miR-6511a-5p/hsa-mir-6511b-2	1.253642	0.615645	1.891639	1.505597	3.896978	0.38635	0.699237	NA
hsa-miR-6511b-5p/hsa-mir-6511a-1	1.253642	0.615645	1.891639	1.505597	3.896978	0.38635	0.699237	NA
hsa-miR-6511b-5p/hsa-mir-6511a-2	1.253642	0.615645	1.891639	1.505597	3.896978	0.38635	0.699237	NA
hsa-miR-6511b-5p/hsa-mir-6511a-3	1.253642	0.615645	1.891639	1.505597	3.896978	0.38635	0.699237	NA
hsa-miR-6511b-5p/hsa-mir-6511a-4	1.253642	0.615645	1.891639	1.505597	3.896978	0.38635	0.699237	NA
hsa-miR-6511b-5p/hsa-mir-6511b-1	1.253642	0.615645	1.891639	1.505597	3.896978	0.38635	0.699237	NA
hsa-miR-6511b-5p/hsa-mir-6511b-2	1.253642	0.615645	1.891639	1.505597	3.896978	0.38635	0.699237	NA
hsa-miR-6512-3p/hsa-mir-6512	0	0	0	NA	NA	NA	NA	NA
hsa-miR-6512-5p/hsa-mir-6512	0	0	0	NA	NA	NA	NA	NA
hsa-miR-6513-5p/hsa-mir-6513	2.388227	2.35613	2.420325	− 0.00229	2.771723	− 0.00083	0.999341	NA
hsa-miR-6514-3p/hsa-mir-6514	0.264343	0	0.528686	2.396631	4.924788	0.486646	0.626509	NA
hsa-miR-6515-3p/hsa-mir-6515	0.528686	0	1.057371	3.044078	4.909871	0.619992	0.535263	NA
hsa-miR-6515-5p/hsa-mir-6515	3.90044	2.249681	5.551199	1.384397	2.541374	0.544744	0.58593	NA
hsa-miR-6529-3p/hsa-mir-6529	0	0	0	NA	NA	NA	NA	NA
hsa-miR-6529-5p/hsa-mir-6529	0	0	0	NA	NA	NA	NA	NA
hsa-miR-654-5p/hsa-mir-654	0	0	0	NA	NA	NA	NA	NA
hsa-miR-655-3p/hsa-mir-655	0.572165	0.615645	0.528686	0.276985	4.848317	0.05713	0.954442	NA
hsa-miR-655-5p/hsa-mir-655	0	0	0	NA	NA	NA	NA	NA
hsa-miR-656-3p/hsa-mir-656	0	0	0	NA	NA	NA	NA	NA
hsa-miR-656-5p/hsa-mir-656	0	0	0	NA	NA	NA	NA	NA
hsa-miR-657/hsa-mir-657	0	0	0	NA	NA	NA	NA	NA
hsa-miR-658/hsa-mir-658	0.153911	0.307822	0	− 0.74599	4.97963	− 0.14981	0.880917	NA
hsa-miR-659-3p/hsa-mir-659	0.813648	0	1.627296	3.418215	4.892272	0.698697	0.484741	NA
hsa-miR-659-5p/hsa-mir-659	5.288513	4.902109	5.674917	0.156221	2.054895	0.076024	0.9394	NA
hsa-miR-661/hsa-mir-661	0	0	0	NA	NA	NA	NA	NA
hsa-miR-662/hsa-mir-662	1.586057	0	3.172114	4.501727	4.332487	1.039063	0.298775	NA
hsa-miR-663a/hsa-mir-663a	0.264343	0	0.528686	2.396631	4.924788	0.486646	0.626509	NA
hsa-miR-663b/hsa-mir-663b	0.396514	0	0.793028	2.767752	4.915475	0.563069	0.573388	NA
hsa-miR-665/hsa-mir-665	0	0	0	NA	NA	NA	NA	NA
hsa-miR-668-3p/hsa-mir-668	0	0	0	NA	NA	NA	NA	NA
hsa-miR-668-5p/hsa-mir-668	0	0	0	NA	NA	NA	NA	NA
hsa-miR-670-3p/hsa-mir-670	0	0	0	NA	NA	NA	NA	NA
hsa-miR-670-5p/hsa-mir-670	0	0	0	NA	NA	NA	NA	NA
hsa-miR-6715a-3p/hsa-mir-6715a	0.461733	0.923467	0	− 2.09228	4.877731	− 0.42895	0.667962	NA
hsa-miR-6715b-3p/hsa-mir-6715b	0	0	0	NA	NA	NA	NA	NA
hsa-miR-6715b-5p/hsa-mir-6715b	0	0	0	NA	NA	NA	NA	NA
hsa-miR-6716-5p/hsa-mir-6716	0	0	0	NA	NA	NA	NA	NA
hsa-miR-6717-5p/hsa-mir-6717	0	0	0	NA	NA	NA	NA	NA
hsa-miR-6718-5p/hsa-mir-6718	0	0	0	NA	NA	NA	NA	NA
hsa-miR-6719-3p/hsa-mir-6719	0	0	0	NA	NA	NA	NA	NA
hsa-miR-6721-5p/hsa-mir-6721	0.153911	0.307822	0	− 0.74599	4.97963	− 0.14981	0.880917	NA
hsa-miR-6722-3p/hsa-mir-6722	0	0	0	NA	NA	NA	NA	NA
hsa-miR-6722-5p/hsa-mir-6722	0	0	0	NA	NA	NA	NA	NA
hsa-miR-6726-3p/hsa-mir-6726	0.396514	0	0.793028	2.767752	4.915475	0.563069	0.573388	NA
hsa-miR-6726-5p/hsa-mir-6726	0.254598	0.509196	0	− 1.11434	4.959133	− 0.2247	0.82221	NA
hsa-miR-6727-3p/hsa-mir-6727	0	0	0	NA	NA	NA	NA	NA
hsa-miR-6727-5p/hsa-mir-6727	0.396514	0	0.793028	2.767752	4.915475	0.563069	0.573388	NA
hsa-miR-6728-3p/hsa-mir-6728	0	0	0	NA	NA	NA	NA	NA
hsa-miR-6728-5p/hsa-mir-6728	1.864644	2.143232	1.586057	− 0.26637	3.269483	− 0.08147	0.935067	NA
hsa-miR-6729-3p/hsa-mir-6729	0	0	0	NA	NA	NA	NA	NA
hsa-miR-6729-5p/hsa-mir-6729	0	0	0	NA	NA	NA	NA	NA
hsa-miR-6730-3p/hsa-mir-6730	1.362953	0	2.725907	4.163156	4.684664	0.888677	0.374176	NA
hsa-miR-6730-5p/hsa-mir-6730	1.496245	0.307822	2.684667	2.849076	3.684969	0.773162	0.439427	NA
hsa-miR-6731-3p/hsa-mir-6731	0	0	0	NA	NA	NA	NA	NA
hsa-miR-6731-5p/hsa-mir-6731	2.440944	0	4.881889	5.018156	3.401403	1.475319	0.140127	NA
hsa-miR-6732-3p/hsa-mir-6732	1.4865	0.817018	2.155982	1.377862	3.350069	0.411294	0.680857	NA
hsa-miR-6732-5p/hsa-mir-6732	0	0	0	NA	NA	NA	NA	NA
hsa-miR-6733-3p/hsa-mir-6733	0.418254	0.307822	0.528686	1.050318	4.906666	0.214059	0.830501	NA
hsa-miR-6734-3p/hsa-mir-6734	0.418254	0.307822	0.528686	1.050318	4.906666	0.214059	0.830501	NA
hsa-miR-6734-5p/hsa-mir-6734	2.832204	2.451054	3.213353	0.422747	2.559576	0.165163	0.868816	NA
hsa-miR-6735-3p/hsa-mir-6735	1.272989	2.545979	0	− 3.51573	4.761337	− 0.73839	0.460277	NA
hsa-miR-6735-5p/hsa-mir-6735	0.153911	0.307822	0	− 0.74599	4.97963	− 0.14981	0.880917	NA
hsa-miR-6736-3p/hsa-mir-6736	0.132171	0	0.264343	1.823505	4.944331	0.368807	0.712271	NA
hsa-miR-6736-5p/hsa-mir-6736	0.153911	0.307822	0	− 0.74599	4.97963	− 0.14981	0.880917	NA
hsa-miR-6737-3p/hsa-mir-6737	0.132171	0	0.264343	1.823505	4.944331	0.368807	0.712271	NA
hsa-miR-6737-5p/hsa-mir-6737	0	0	0	NA	NA	NA	NA	NA
hsa-miR-6738-3p/hsa-mir-6738	0	0	0	NA	NA	NA	NA	NA
hsa-miR-6738-5p/hsa-mir-6738	0.54068	0.817018	0.264343	− 0.65628	4.837961	− 0.13565	0.892097	NA
hsa-miR-6739-3p/hsa-mir-6739	0.660857	0	1.321714	3.278941	4.90256	0.668822	0.503609	NA
hsa-miR-6739-5p/hsa-mir-6739	1.332589	0.509196	2.155982	2.185436	3.862236	0.565847	0.571498	NA
hsa-miR-6740-3p/hsa-mir-6740	0	0	0	NA	NA	NA	NA	NA
hsa-miR-6740-5p/hsa-mir-6740	0.264343	0	0.528686	2.396631	4.924788	0.486646	0.626509	NA
hsa-miR-6741-3p/hsa-mir-6741	0.814768	0.307822	1.321714	1.93264	4.884354	0.39568	0.692341	NA
hsa-miR-6741-5p/hsa-mir-6741	1.617551	0.509196	2.725907	2.456594	4.044713	0.607359	0.543612	NA
hsa-miR-6742-3p/hsa-mir-6742	0.264343	0	0.528686	2.396631	4.924788	0.486646	0.626509	NA
hsa-miR-6742-5p/hsa-mir-6742	0	0	0	NA	NA	NA	NA	NA
hsa-miR-6743-3p/hsa-mir-6743	0	0	0	NA	NA	NA	NA	NA
hsa-miR-6743-5p/hsa-mir-6743	0	0	0	NA	NA	NA	NA	NA
hsa-miR-6744-3p/hsa-mir-6744	0	0	0	NA	NA	NA	NA	NA
hsa-miR-6744-5p/hsa-mir-6744	0	0	0	NA	NA	NA	NA	NA
hsa-miR-6745/hsa-mir-6745	0	0	0	NA	NA	NA	NA	NA
hsa-miR-6746-3p/hsa-mir-6746	0	0	0	NA	NA	NA	NA	NA
hsa-miR-6746-5p/hsa-mir-6746	0	0	0	NA	NA	NA	NA	NA
hsa-miR-6747-3p/hsa-mir-6747	0.990419	0.923467	1.057371	0.353246	4.651503	0.075942	0.939465	NA
hsa-miR-6747-5p/hsa-mir-6747	0	0	0	NA	NA	NA	NA	NA
hsa-miR-6748-3p/hsa-mir-6748	3.407383	0	6.814767	5.498866	3.489578	1.575797	0.115073	NA
hsa-miR-6748-5p/hsa-mir-6748	0.132171	0	0.264343	1.823505	4.944331	0.368807	0.712271	NA
hsa-miR-6749-3p/hsa-mir-6749	0	0	0	NA	NA	NA	NA	NA
hsa-miR-6749-5p/hsa-mir-6749	0	0	0	NA	NA	NA	NA	NA
hsa-miR-6750-3p/hsa-mir-6750	0	0	0	NA	NA	NA	NA	NA
hsa-miR-6750-5p/hsa-mir-6750	1.14433	1.231289	1.057371	− 0.05088	4.392583	− 0.01158	0.990758	NA
hsa-miR-6751-3p/hsa-mir-6751	0	0	0	NA	NA	NA	NA	NA
hsa-miR-6751-5p/hsa-mir-6751	2.02381	0	4.047621	4.770558	3.585675	1.330449	0.18337	NA
hsa-miR-6752-3p/hsa-mir-6752	0	0	0	NA	NA	NA	NA	NA
hsa-miR-6752-5p/hsa-mir-6752	0	0	0	NA	NA	NA	NA	NA
hsa-miR-6753-3p/hsa-mir-6753	0.132171	0	0.264343	1.823505	4.944331	0.368807	0.712271	NA
hsa-miR-6753-5p/hsa-mir-6753	0	0	0	NA	NA	NA	NA	NA
hsa-miR-6754-3p/hsa-mir-6754	0	0	0	NA	NA	NA	NA	NA
hsa-miR-6754-5p/hsa-mir-6754	0	0	0	NA	NA	NA	NA	NA
hsa-miR-6755-3p/hsa-mir-6755	1.891026	2.154756	1.627296	− 0.55566	3.401433	− 0.16336	0.870234	NA
hsa-miR-6755-5p/hsa-mir-6755	3.336909	1.527587	5.146231	1.751366	2.750344	0.636781	0.524268	NA
hsa-miR-6756-3p/hsa-mir-6756	0	0	0	NA	NA	NA	NA	NA
hsa-miR-6756-5p/hsa-mir-6756	0	0	0	NA	NA	NA	NA	NA
hsa-miR-6757-3p/hsa-mir-6757	0	0	0	NA	NA	NA	NA	NA
hsa-miR-6757-5p/hsa-mir-6757	0	0	0	NA	NA	NA	NA	NA
hsa-miR-6758-3p/hsa-mir-6758	0.386769	0.509196	0.264343	0.108828	4.90556	0.022185	0.982301	NA
hsa-miR-6758-5p/hsa-mir-6758	0.773539	1.018392	0.528686	− 0.3819	4.797831	− 0.0796	0.936556	NA
hsa-miR-6759-3p/hsa-mir-6759	0	0	0	NA	NA	NA	NA	NA
hsa-miR-6759-5p/hsa-mir-6759	0.716331	1.432663	0	− 2.70323	4.845928	− 0.55784	0.576957	NA
hsa-miR-6760-3p/hsa-mir-6760	0	0	0	NA	NA	NA	NA	NA
hsa-miR-6760-5p/hsa-mir-6760	0	0	0	NA	NA	NA	NA	NA
hsa-miR-6761-3p/hsa-mir-6761	0.528686	0	1.057371	3.044078	4.909871	0.619992	0.535263	NA
hsa-miR-6761-5p/hsa-mir-6761	3.947912	3.055175	4.840649	0.681756	2.592781	0.262944	0.792594	NA
hsa-miR-6762-3p/hsa-mir-6762	0.418254	0.307822	0.528686	1.050318	4.906666	0.214059	0.830501	NA
hsa-miR-6762-5p/hsa-mir-6762	0.923467	1.846934	0	− 3.07898	4.832001	− 0.63721	0.523991	NA
hsa-miR-6763-3p/hsa-mir-6763	0	0	0	NA	NA	NA	NA	NA
hsa-miR-6763-5p/hsa-mir-6763	0.90571	1.018392	0.793028	− 0.01078	4.788271	− 0.00225	0.998203	NA
hsa-miR-676-3p/hsa-mir-676	4.995529	2.853801	7.137256	1.423018	2.447835	0.581337	0.561013	NA
hsa-miR-6764-3p/hsa-mir-6764	1.651276	0.923467	2.379085	1.411188	3.807356	0.370648	0.7109	NA
hsa-miR-6764-5p/hsa-mir-6764	3.664222	1.12484	6.203603	2.422752	2.411191	1.004794	0.314996	NA
hsa-miR-6765-3p/hsa-mir-6765	0.264343	0	0.528686	2.396631	4.924788	0.486646	0.626509	NA
hsa-miR-6765-5p/hsa-mir-6765	0	0	0	NA	NA	NA	NA	NA
hsa-miR-676-5p/hsa-mir-676	0.254598	0.509196	0	− 1.11434	4.959133	− 0.2247	0.82221	NA
hsa-miR-6766-3p/hsa-mir-6766	0.805023	0.817018	0.793028	0.293348	4.583511	0.064001	0.94897	NA
hsa-miR-6766-5p/hsa-mir-6766	0	0	0	NA	NA	NA	NA	NA
hsa-miR-6767-3p/hsa-mir-6767	0.264343	0	0.528686	2.396631	4.924788	0.486646	0.626509	NA
hsa-miR-6767-5p/hsa-mir-6767	1.517985	0.615645	2.420325	1.893906	3.605705	0.525253	0.599408	NA
hsa-miR-6768-3p/hsa-mir-6768	0	0	0	NA	NA	NA	NA	NA
hsa-miR-6768-5p/hsa-mir-6768	2.221201	0.923467	3.518935	1.811652	3.12682	0.579391	0.562325	NA
hsa-miR-6769a-3p/hsa-mir-6769a	0	0	0	NA	NA	NA	NA	NA
hsa-miR-6769a-5p/hsa-mir-6769a	0	0	0	NA	NA	NA	NA	NA
hsa-miR-6769b-3p/hsa-mir-6769b	0	0	0	NA	NA	NA	NA	NA
hsa-miR-6769b-5p/hsa-mir-6769b	0.254598	0.509196	0	− 1.11434	4.959133	− 0.2247	0.82221	NA
hsa-miR-6770-3p/hsa-mir-6770-1	1.222157	0.817018	1.627296	0.894076	3.656869	0.244492	0.80685	NA
hsa-miR-6770-3p/hsa-mir-6770-2	1.222157	0.817018	1.627296	0.894076	3.656869	0.244492	0.80685	NA
hsa-miR-6770-3p/hsa-mir-6770-3	1.222157	0.817018	1.627296	0.894076	3.656869	0.244492	0.80685	NA
hsa-miR-6770-5p/hsa-mir-6770-1	0	0	0	NA	NA	NA	NA	NA
hsa-miR-6770-5p/hsa-mir-6770-2	0	0	0	NA	NA	NA	NA	NA
hsa-miR-6770-5p/hsa-mir-6770-3	0	0	0	NA	NA	NA	NA	NA
hsa-miR-6771-3p/hsa-mir-6771	0.408509	0.817018	0	− 1.87945	4.892274	− 0.38417	0.700854	NA
hsa-miR-6771-5p/hsa-mir-6771	0	0	0	NA	NA	NA	NA	NA
hsa-miR-6772-5p/hsa-mir-6772	0.307822	0.615645	0	− 1.51932	4.922146	− 0.30867	0.757573	NA
hsa-miR-6773-3p/hsa-mir-6773	0.132171	0	0.264343	1.823505	4.944331	0.368807	0.712271	NA
hsa-miR-6773-5p/hsa-mir-6773	0	0	0	NA	NA	NA	NA	NA
hsa-miR-6774-3p/hsa-mir-6774	0	0	0	NA	NA	NA	NA	NA
hsa-miR-6774-5p/hsa-mir-6774	0	0	0	NA	NA	NA	NA	NA
hsa-miR-6775-3p/hsa-mir-6775	0.286083	0.307822	0.264343	0.477194	4.926281	0.096867	0.922832	NA
hsa-miR-6775-5p/hsa-mir-6775	0.153911	0.307822	0	− 0.74599	4.97963	− 0.14981	0.880917	NA
hsa-miR-6776-3p/hsa-mir-6776	0	0	0	NA	NA	NA	NA	NA
hsa-miR-6776-5p/hsa-mir-6776	0	0	0	NA	NA	NA	NA	NA
hsa-miR-6777-3p/hsa-mir-6777	0.967559	0.307822	1.627296	2.052589	4.384489	0.468148	0.639679	NA
hsa-miR-6777-5p/hsa-mir-6777	2.257789	0.509196	4.006382	3.176749	3.221118	0.986226	0.324022	NA
hsa-miR-6778-3p/hsa-mir-6778	0	0	0	NA	NA	NA	NA	NA
hsa-miR-6778-5p/hsa-mir-6778	0	0	0	NA	NA	NA	NA	NA
hsa-miR-6779-3p/hsa-mir-6779	0	0	0	NA	NA	NA	NA	NA
hsa-miR-6779-5p/hsa-mir-6779	0.509196	1.018392	0	− 2.1782	4.872426	− 0.44705	0.654841	NA
hsa-miR-6780a-3p/hsa-mir-6780a	0	0	0	NA	NA	NA	NA	NA
hsa-miR-6780a-5p/hsa-mir-6780a	0.132171	0	0.264343	1.823505	4.944331	0.368807	0.712271	NA
hsa-miR-6780b-3p/hsa-mir-6780b	1.059621	1.326214	0.793028	− 0.3881	4.101081	− 0.09463	0.924607	NA
hsa-miR-6780b-5p/hsa-mir-6780b	0	0	0	NA	NA	NA	NA	NA
hsa-miR-6781-3p/hsa-mir-6781	0.550425	0.307822	0.793028	1.42144	4.897318	0.290249	0.771626	NA
hsa-miR-6781-5p/hsa-mir-6781	0	0	0	NA	NA	NA	NA	NA
hsa-miR-6782-3p/hsa-mir-6782	0.418254	0.307822	0.528686	1.050318	4.906666	0.214059	0.830501	NA
hsa-miR-6782-5p/hsa-mir-6782	0	0	0	NA	NA	NA	NA	NA
hsa-miR-6783-3p/hsa-mir-6783	0.509196	1.018392	0	− 2.1782	4.872426	− 0.44705	0.654841	NA
hsa-miR-6783-5p/hsa-mir-6783	5.510496	4.594286	6.426706	0.513629	2.014699	0.254941	0.798769	NA
hsa-miR-6784-3p/hsa-mir-6784	0.132171	0	0.264343	1.823505	4.944331	0.368807	0.712271	NA
hsa-miR-6784-5p/hsa-mir-6784	0	0	0	NA	NA	NA	NA	NA
hsa-miR-6785-3p/hsa-mir-6785	0	0	0	NA	NA	NA	NA	NA
hsa-miR-6785-5p/hsa-mir-6785	1.362953	0	2.725907	4.163156	4.684664	0.888677	0.374176	NA
hsa-miR-6786-3p/hsa-mir-6786	4.080591	5.517753	2.643428	− 0.89651	2.447538	− 0.36629	0.714149	NA
hsa-miR-6786-5p/hsa-mir-6786	0	0	0	NA	NA	NA	NA	NA
hsa-miR-6787-3p/hsa-mir-6787	0	0	0	NA	NA	NA	NA	NA
hsa-miR-6787-5p/hsa-mir-6787	0	0	0	NA	NA	NA	NA	NA
hsa-miR-6788-3p/hsa-mir-6788	0	0	0	NA	NA	NA	NA	NA
hsa-miR-6788-5p/hsa-mir-6788	0	0	0	NA	NA	NA	NA	NA
hsa-miR-6789-3p/hsa-mir-6789	0	0	0	NA	NA	NA	NA	NA
hsa-miR-6789-5p/hsa-mir-6789	0.264343	0	0.528686	2.396631	4.924788	0.486646	0.626509	NA
hsa-miR-6790-3p/hsa-mir-6790	0	0	0	NA	NA	NA	NA	NA
hsa-miR-6790-5p/hsa-mir-6790	0.264343	0	0.528686	2.396631	4.924788	0.486646	0.626509	NA
hsa-miR-6791-3p/hsa-mir-6791	0	0	0	NA	NA	NA	NA	NA
hsa-miR-6791-5p/hsa-mir-6791	0	0	0	NA	NA	NA	NA	NA
hsa-miR-6792-3p/hsa-mir-6792	0	0	0	NA	NA	NA	NA	NA
hsa-miR-6792-5p/hsa-mir-6792	0.945819	0	1.891639	3.662379	4.791539	0.764343	0.444663	NA
hsa-miR-6793-3p/hsa-mir-6793	0	0	0	NA	NA	NA	NA	NA
hsa-miR-6793-5p/hsa-mir-6793	1.12484	2.249681	0	− 3.35016	4.41154	− 0.75941	0.447608	NA
hsa-miR-6794-3p/hsa-mir-6794	0.132171	0	0.264343	1.823505	4.944331	0.368807	0.712271	NA
hsa-miR-6794-5p/hsa-mir-6794	0	0	0	NA	NA	NA	NA	NA
hsa-miR-6795-3p/hsa-mir-6795	0	0	0	NA	NA	NA	NA	NA
hsa-miR-6795-5p/hsa-mir-6795	0.681477	0	1.362953	3.012055	4.910469	0.613395	0.539615	NA
hsa-miR-6796-3p/hsa-mir-6796	0	0	0	NA	NA	NA	NA	NA
hsa-miR-6796-5p/hsa-mir-6796	0.396514	0	0.793028	2.767752	4.915475	0.563069	0.573388	NA
hsa-miR-6797-3p/hsa-mir-6797	1.802947	0.615645	2.99025	2.12133	3.469393	0.611441	0.540907	NA
hsa-miR-6797-5p/hsa-mir-6797	0	0	0	NA	NA	NA	NA	NA
hsa-miR-6798-3p/hsa-mir-6798	0.286083	0.307822	0.264343	0.477194	4.926281	0.096867	0.922832	NA
hsa-miR-6798-5p/hsa-mir-6798	0.264343	0	0.528686	2.396631	4.924788	0.486646	0.626509	NA
hsa-miR-6799-3p/hsa-mir-6799	0.396514	0	0.793028	2.767752	4.915475	0.563069	0.573388	NA
hsa-miR-6799-5p/hsa-mir-6799	0.254598	0.509196	0	− 1.11434	4.959133	− 0.2247	0.82221	NA
hsa-miR-6800-3p/hsa-mir-6800	1.385813	0.615645	2.155982	1.71397	3.737301	0.458612	0.646513	NA
hsa-miR-6800-5p/hsa-mir-6800	0.132171	0	0.264343	1.823505	4.944331	0.368807	0.712271	NA
hsa-miR-6801-3p/hsa-mir-6801	0	0	0	NA	NA	NA	NA	NA
hsa-miR-6801-5p/hsa-mir-6801	0	0	0	NA	NA	NA	NA	NA
hsa-miR-6802-3p/hsa-mir-6802	0.672852	0.817018	0.528686	− 0.08316	4.817987	− 0.01726	0.98623	NA
hsa-miR-6802-5p/hsa-mir-6802	0.264343	0	0.528686	2.396631	4.924788	0.486646	0.626509	NA
hsa-miR-6803-3p/hsa-mir-6803	1.496245	0.307822	2.684667	2.849076	3.684969	0.773162	0.439427	NA
hsa-miR-6803-5p/hsa-mir-6803	0	0	0	NA	NA	NA	NA	NA
hsa-miR-6804-3p/hsa-mir-6804	0	0	0	NA	NA	NA	NA	NA
hsa-miR-6804-5p/hsa-mir-6804	0.835388	0.307822	1.362953	1.665763	4.892293	0.340487	0.73349	NA
hsa-miR-6805-3p/hsa-mir-6805	0	0	0	NA	NA	NA	NA	NA
hsa-miR-6805-5p/hsa-mir-6805	0.264343	0	0.528686	2.396631	4.924788	0.486646	0.626509	NA
hsa-miR-6806-3p/hsa-mir-6806	2.674923	0.509196	4.840649	3.424821	3.036521	1.127877	0.259372	NA
hsa-miR-6806-5p/hsa-mir-6806	0	0	0	NA	NA	NA	NA	NA
hsa-miR-6807-3p/hsa-mir-6807	1.607797	0.307822	2.907771	3.041983	3.939011	0.772271	0.439954	NA
hsa-miR-6808-3p/hsa-mir-6808	0.286083	0.307822	0.264343	0.477194	4.926281	0.096867	0.922832	NA
hsa-miR-6808-5p/hsa-mir-6808	0	0	0	NA	NA	NA	NA	NA
hsa-miR-6809-3p/hsa-mir-6809	0	0	0	NA	NA	NA	NA	NA
hsa-miR-6809-5p/hsa-mir-6809	0	0	0	NA	NA	NA	NA	NA
hsa-miR-6810-3p/hsa-mir-6810	0	0	0	NA	NA	NA	NA	NA
hsa-miR-6810-5p/hsa-mir-6810	0	0	0	NA	NA	NA	NA	NA
hsa-miR-6811-3p/hsa-mir-6811	0	0	0	NA	NA	NA	NA	NA
hsa-miR-6811-5p/hsa-mir-6811	0.153911	0.307822	0	− 0.74599	4.97963	− 0.14981	0.880917	NA
hsa-miR-6812-3p/hsa-mir-6812	0.681477	0	1.362953	3.012055	4.910469	0.613395	0.539615	NA
hsa-miR-6812-5p/hsa-mir-6812	0	0	0	NA	NA	NA	NA	NA
hsa-miR-6813-3p/hsa-mir-6813	0	0	0	NA	NA	NA	NA	NA
hsa-miR-6813-5p/hsa-mir-6813	0.132171	0	0.264343	1.823505	4.944331	0.368807	0.712271	NA
hsa-miR-6814-3p/hsa-mir-6814	0.813648	0	1.627296	3.418215	4.892272	0.698697	0.484741	NA
hsa-miR-6814-5p/hsa-mir-6814	1.210162	0	2.420325	4.044592	4.335033	0.933001	0.350819	NA
hsa-miR-6815-3p/hsa-mir-6815	0.763794	1.527587	0	− 2.77189	4.84311	− 0.57234	0.567094	NA
hsa-miR-6815-5p/hsa-mir-6815	0.254598	0.509196	0	− 1.11434	4.959133	− 0.2247	0.82221	NA
hsa-miR-6816-3p/hsa-mir-6816	0	0	0	NA	NA	NA	NA	NA
hsa-miR-6816-5p/hsa-mir-6816	2.04443	0	4.08886	4.753613	4.067207	1.168766	0.242498	NA
hsa-miR-6817-3p/hsa-mir-6817	0.990419	0.923467	1.057371	0.353246	4.651503	0.075942	0.939465	NA
hsa-miR-6817-5p/hsa-mir-6817	0.945819	0	1.891639	3.662379	4.791539	0.764343	0.444663	NA
hsa-miR-6818-3p/hsa-mir-6818	1.160308	1.527587	0.793028	− 0.59382	4.404519	− 0.13482	0.892754	NA
hsa-miR-6818-5p/hsa-mir-6818	0.264343	0	0.528686	2.396631	4.924788	0.486646	0.626509	NA
hsa-miR-6819-3p/hsa-mir-6819	0	0	0	NA	NA	NA	NA	NA
hsa-miR-6819-5p/hsa-mir-6819	0.386769	0.509196	0.264343	0.108828	4.90556	0.022185	0.982301	NA
hsa-miR-6820-3p/hsa-mir-6820	0.615645	1.231289	0	− 2.50085	4.855042	− 0.5151	0.606481	NA
hsa-miR-6820-5p/hsa-mir-6820	0.528686	0	1.057371	3.044078	4.909871	0.619992	0.535263	NA
hsa-miR-6821-3p/hsa-mir-6821	0	0	0	NA	NA	NA	NA	NA
hsa-miR-6821-5p/hsa-mir-6821	0.254598	0.509196	0	− 1.11434	4.959133	− 0.2247	0.82221	NA
hsa-miR-6822-3p/hsa-mir-6822	0	0	0	NA	NA	NA	NA	NA
hsa-miR-6822-5p/hsa-mir-6822	0.153911	0.307822	0	− 0.74599	4.97963	− 0.14981	0.880917	NA
hsa-miR-6823-3p/hsa-mir-6823	0	0	0	NA	NA	NA	NA	NA
hsa-miR-6823-5p/hsa-mir-6823	0.264343	0	0.528686	2.396631	4.924788	0.486646	0.626509	NA
hsa-miR-6824-3p/hsa-mir-6824	0.509196	1.018392	0	− 2.1782	4.872426	− 0.44705	0.654841	NA
hsa-miR-6824-5p/hsa-mir-6824	0	0	0	NA	NA	NA	NA	NA
hsa-miR-6825-3p/hsa-mir-6825	0.396514	0	0.793028	2.767752	4.915475	0.563069	0.573388	NA
hsa-miR-6825-5p/hsa-mir-6825	0.528686	0	1.057371	3.044078	4.909871	0.619992	0.535263	NA
hsa-miR-6826-3p/hsa-mir-6826	0.660857	0	1.321714	3.278941	4.90256	0.668822	0.503609	NA
hsa-miR-6826-5p/hsa-mir-6826	4.405653	3.788792	5.022514	0.492374	2.43655	0.202078	0.839855	NA
hsa-miR-6827-3p/hsa-mir-6827	0.264343	0	0.528686	2.396631	4.924788	0.486646	0.626509	NA
hsa-miR-6827-5p/hsa-mir-6827	1.729103	0.509196	2.94901	2.694291	3.513585	0.766821	0.443188	NA
hsa-miR-6828-3p/hsa-mir-6828	0	0	0	NA	NA	NA	NA	NA
hsa-miR-6828-5p/hsa-mir-6828	0.264343	0	0.528686	2.396631	4.924788	0.486646	0.626509	NA
hsa-miR-6829-3p/hsa-mir-6829	0	0	0	NA	NA	NA	NA	NA
hsa-miR-6829-5p/hsa-mir-6829	0	0	0	NA	NA	NA	NA	NA
hsa-miR-6830-3p/hsa-mir-6830	1.872139	0.307822	3.436457	3.278334	3.740667	0.876404	0.380811	NA
hsa-miR-6830-5p/hsa-mir-6830	0	0	0	NA	NA	NA	NA	NA
hsa-miR-6831-3p/hsa-mir-6831	0.132171	0	0.264343	1.823505	4.944331	0.368807	0.712271	NA
hsa-miR-6831-5p/hsa-mir-6831	0.264343	0	0.528686	2.396631	4.924788	0.486646	0.626509	NA
hsa-miR-6832-3p/hsa-mir-6832	2.399705	0	4.79941	5.071506	3.401346	1.491029	0.135954	NA
hsa-miR-6832-5p/hsa-mir-6832	3.498316	0	6.996631	5.574776	2.994364	1.861757	0.062637	NA
hsa-miR-6833-3p/hsa-mir-6833	0	0	0	NA	NA	NA	NA	NA
hsa-miR-6833-5p/hsa-mir-6833	0.264343	0	0.528686	2.396631	4.924788	0.486646	0.626509	NA
hsa-miR-6834-3p/hsa-mir-6834	0.132171	0	0.264343	1.823505	4.944331	0.368807	0.712271	NA
hsa-miR-6834-5p/hsa-mir-6834	0	0	0	NA	NA	NA	NA	NA
hsa-miR-6835-3p/hsa-mir-6835	0	0	0	NA	NA	NA	NA	NA
hsa-miR-6835-5p/hsa-mir-6835	0	0	0	NA	NA	NA	NA	NA
hsa-miR-6836-3p/hsa-mir-6836	0	0	0	NA	NA	NA	NA	NA
hsa-miR-6836-5p/hsa-mir-6836	0	0	0	NA	NA	NA	NA	NA
hsa-miR-6837-3p/hsa-mir-6837	2.107248	1.83541	2.379085	0.516274	3.168012	0.162965	0.870546	NA
hsa-miR-6837-5p/hsa-mir-6837	0.132171	0	0.264343	1.823505	4.944331	0.368807	0.712271	NA
hsa-miR-6838-3p/hsa-mir-6838	0	0	0	NA	NA	NA	NA	NA
hsa-miR-6838-5p/hsa-mir-6838	0.153911	0.307822	0	− 0.74599	4.97963	− 0.14981	0.880917	NA
hsa-miR-6839-3p/hsa-mir-6839	0.254598	0.509196	0	− 1.11434	4.959133	− 0.2247	0.82221	NA
hsa-miR-6839-5p/hsa-mir-6839	1.48762	1.12484	1.8504	0.835112	3.585059	0.232942	0.815806	NA
hsa-miR-6840-3p/hsa-mir-6840	3.344299	4.309513	2.379085	− 0.74548	3.001329	− 0.24838	0.803837	NA
hsa-miR-6840-5p/hsa-mir-6840	1.362953	0	2.725907	4.163156	4.684664	0.888677	0.374176	NA
hsa-miR-6841-3p/hsa-mir-6841	0	0	0	NA	NA	NA	NA	NA
hsa-miR-6841-5p/hsa-mir-6841	0	0	0	NA	NA	NA	NA	NA
hsa-miR-6842-3p/hsa-mir-6842	4.514822	6.121874	2.907771	− 0.90865	2.397011	− 0.37908	0.704631	NA
hsa-miR-6842-5p/hsa-mir-6842	0	0	0	NA	NA	NA	NA	NA
hsa-miR-6843-3p/hsa-mir-6843	0	0	0	NA	NA	NA	NA	NA
hsa-miR-6845-3p/hsa-mir-6845	0	0	0	NA	NA	NA	NA	NA
hsa-miR-6845-5p/hsa-mir-6845	0	0	0	NA	NA	NA	NA	NA
hsa-miR-6846-3p/hsa-mir-6846	0.264343	0	0.528686	2.396631	4.924788	0.486646	0.626509	NA
hsa-miR-6846-5p/hsa-mir-6846	0.132171	0	0.264343	1.823505	4.944331	0.368807	0.712271	NA
hsa-miR-6847-3p/hsa-mir-6847	3.041072	1.018392	5.063753	2.425857	2.847338	0.851974	0.394229	NA
hsa-miR-6847-5p/hsa-mir-6847	5.237163	0	10.47433	6.165728	2.676662	2.303514	0.02125	NA
hsa-miR-6848-3p/hsa-mir-6848	0	0	0	NA	NA	NA	NA	NA
hsa-miR-6848-5p/hsa-mir-6848	0.396514	0	0.793028	2.767752	4.915475	0.563069	0.573388	NA
hsa-miR-6849-3p/hsa-mir-6849	0	0	0	NA	NA	NA	NA	NA
hsa-miR-6849-5p/hsa-mir-6849	0	0	0	NA	NA	NA	NA	NA
hsa-miR-6850-3p/hsa-mir-6850	0.132171	0	0.264343	1.823505	4.944331	0.368807	0.712271	NA
hsa-miR-6850-5p/hsa-mir-6850	0.439994	0.615645	0.264343	− 0.29614	4.868166	− 0.06083	0.951493	NA
hsa-miR-6851-3p/hsa-mir-6851	0	0	0	NA	NA	NA	NA	NA
hsa-miR-6851-5p/hsa-mir-6851	0.254598	0.509196	0	− 1.11434	4.959133	− 0.2247	0.82221	NA
hsa-miR-6852-3p/hsa-mir-6852	0	0	0	NA	NA	NA	NA	NA
hsa-miR-6852-5p/hsa-mir-6852	4.396682	0.615645	8.17772	3.585634	2.961549	1.21073	0.225999	NA
hsa-miR-6853-3p/hsa-mir-6853	0	0	0	NA	NA	NA	NA	NA
hsa-miR-6853-5p/hsa-mir-6853	0.132171	0	0.264343	1.823505	4.944331	0.368807	0.712271	NA
hsa-miR-6854-3p/hsa-mir-6854	0.153911	0.307822	0	− 0.74599	4.97963	− 0.14981	0.880917	NA
hsa-miR-6854-5p/hsa-mir-6854	5.751979	3.978642	7.525317	0.930595	2.010681	0.462826	0.643489	NA
hsa-miR-6855-3p/hsa-mir-6855	0.682597	0.307822	1.057371	1.697766	4.891693	0.347071	0.728538	NA
hsa-miR-6855-5p/hsa-mir-6855	0.528686	0	1.057371	3.044078	4.909871	0.619992	0.535263	NA
hsa-miR-6856-3p/hsa-mir-6856	1.122591	0.923467	1.321714	0.596069	4.423869	0.134739	0.892818	NA
hsa-miR-6856-5p/hsa-mir-6856	0	0	0	NA	NA	NA	NA	NA
hsa-miR-6857-3p/hsa-mir-6857	0.408509	0.817018	0	− 1.87945	4.892274	− 0.38417	0.700854	NA
hsa-miR-6857-5p/hsa-mir-6857	1.059621	1.326214	0.793028	− 0.3881	4.101081	− 0.09463	0.924607	NA
hsa-miR-6858-3p/hsa-mir-6858	1.342334	0	2.684667	4.202922	4.165197	1.009057	0.312947	NA
hsa-miR-6858-5p/hsa-mir-6858	0	0	0	NA	NA	NA	NA	NA
hsa-miR-6859-3p/hsa-mir-6859-1	1.606677	0	3.213353	4.474732	3.896794	1.148311	0.25084	NA
hsa-miR-6859-3p/hsa-mir-6859-2	1.606677	0	3.213353	4.474732	3.896794	1.148311	0.25084	NA
hsa-miR-6859-3p/hsa-mir-6859-3	1.606677	0	3.213353	4.474732	3.896794	1.148311	0.25084	NA
hsa-miR-6859-3p/hsa-mir-6859-4	1.606677	0	3.213353	4.474732	3.896794	1.148311	0.25084	NA
hsa-miR-6859-5p/hsa-mir-6859-1	1.519105	0.923467	2.114743	1.247611	3.927828	0.317634	0.750763	NA
hsa-miR-6859-5p/hsa-mir-6859-2	1.519105	0.923467	2.114743	1.247611	3.927828	0.317634	0.750763	NA
hsa-miR-6859-5p/hsa-mir-6859-3	1.519105	0.923467	2.114743	1.247611	3.927828	0.317634	0.750763	NA
hsa-miR-6859-5p/hsa-mir-6859-4	1.519105	0.923467	2.114743	1.247611	3.927828	0.317634	0.750763	NA
hsa-miR-6860/hsa-mir-6860	0.681477	0	1.362953	3.012055	4.910469	0.613395	0.539615	NA
hsa-miR-6861-3p/hsa-mir-6861	0	0	0	NA	NA	NA	NA	NA
hsa-miR-6861-5p/hsa-mir-6861	0	0	0	NA	NA	NA	NA	NA
hsa-miR-6862-3p/hsa-mir-6862-1	0	0	0	NA	NA	NA	NA	NA
hsa-miR-6862-3p/hsa-mir-6862-2	0	0	0	NA	NA	NA	NA	NA
hsa-miR-6862-5p/hsa-mir-6862-1	0	0	0	NA	NA	NA	NA	NA
hsa-miR-6862-5p/hsa-mir-6862-2	0	0	0	NA	NA	NA	NA	NA
hsa-miR-6863/hsa-mir-6863	0	0	0	NA	NA	NA	NA	NA
hsa-miR-6864-3p/hsa-mir-6864	0	0	0	NA	NA	NA	NA	NA
hsa-miR-6864-5p/hsa-mir-6864	1.189543	0	2.379085	4.089116	4.831661	0.846317	0.397376	NA
hsa-miR-6865-3p/hsa-mir-6865	0	0	0	NA	NA	NA	NA	NA
hsa-miR-6865-5p/hsa-mir-6865	0	0	0	NA	NA	NA	NA	NA
hsa-miR-6866-3p/hsa-mir-6866	0.264343	0	0.528686	2.396631	4.924788	0.486646	0.626509	NA
hsa-miR-6867-3p/hsa-mir-6867	0	0	0	NA	NA	NA	NA	NA
hsa-miR-6867-5p/hsa-mir-6867	0.651112	0.509196	0.793028	1.053061	4.876474	0.215947	0.829029	NA
hsa-miR-6868-5p/hsa-mir-6868	0	0	0	NA	NA	NA	NA	NA
hsa-miR-6869-3p/hsa-mir-6869	0.615645	1.231289	0	− 2.50085	4.855042	− 0.5151	0.606481	NA
hsa-miR-6869-5p/hsa-mir-6869	0.396514	0	0.793028	2.767752	4.915475	0.563069	0.573388	NA
hsa-miR-6870-3p/hsa-mir-6870	0	0	0	NA	NA	NA	NA	NA
hsa-miR-6870-5p/hsa-mir-6870	0	0	0	NA	NA	NA	NA	NA
hsa-miR-6871-3p/hsa-mir-6871	0	0	0	NA	NA	NA	NA	NA
hsa-miR-6871-5p/hsa-mir-6871	0.54068	0.817018	0.264343	− 0.65628	4.837961	− 0.13565	0.892097	NA
hsa-miR-6872-3p/hsa-mir-6872	0	0	0	NA	NA	NA	NA	NA
hsa-miR-6872-5p/hsa-mir-6872	0.763794	1.527587	0	− 2.77189	4.84311	− 0.57234	0.567094	NA
hsa-miR-6873-5p/hsa-mir-6873	0.681477	0	1.362953	3.012055	4.910469	0.613395	0.539615	NA
hsa-miR-6874-3p/hsa-mir-6874	0.550425	0.307822	0.793028	1.42144	4.897318	0.290249	0.771626	NA
hsa-miR-6874-5p/hsa-mir-6874	0	0	0	NA	NA	NA	NA	NA
hsa-miR-6875-3p/hsa-mir-6875	0	0	0	NA	NA	NA	NA	NA
hsa-miR-6875-5p/hsa-mir-6875	0	0	0	NA	NA	NA	NA	NA
hsa-miR-6876-3p/hsa-mir-6876	0	0	0	NA	NA	NA	NA	NA
hsa-miR-6876-5p/hsa-mir-6876	0.681477	0	1.362953	3.012055	4.910469	0.613395	0.539615	NA
hsa-miR-6877-3p/hsa-mir-6877	0	0	0	NA	NA	NA	NA	NA
hsa-miR-6877-5p/hsa-mir-6877	0.793028	0	1.586057	3.52828	4.884751	0.722305	0.470107	NA
hsa-miR-6878-3p/hsa-mir-6878	0	0	0	NA	NA	NA	NA	NA
hsa-miR-6878-5p/hsa-mir-6878	0.264343	0	0.528686	2.396631	4.924788	0.486646	0.626509	NA
hsa-miR-6879-3p/hsa-mir-6879	0	0	0	NA	NA	NA	NA	NA
hsa-miR-6879-5p/hsa-mir-6879	0	0	0	NA	NA	NA	NA	NA
hsa-miR-6880-3p/hsa-mir-6880	0	0	0	NA	NA	NA	NA	NA
hsa-miR-6880-5p/hsa-mir-6880	0	0	0	NA	NA	NA	NA	NA
hsa-miR-6881-3p/hsa-mir-6881	3.53317	1.432663	5.633678	1.959141	2.40437	0.814825	0.415172	NA
hsa-miR-6881-5p/hsa-mir-6881	0	0	0	NA	NA	NA	NA	NA
hsa-miR-6882-3p/hsa-mir-6882	0	0	0	NA	NA	NA	NA	NA
hsa-miR-6882-5p/hsa-mir-6882	6.201728	4.79566	7.607795	0.593291	2.057093	0.288412	0.773031	NA
hsa-miR-6883-3p/hsa-mir-6883	1.783448	0.923467	2.643428	1.557633	3.701546	0.420806	0.673897	NA
hsa-miR-6883-5p/hsa-mir-6883	0	0	0	NA	NA	NA	NA	NA
hsa-miR-6884-3p/hsa-mir-6884	1.873269	1.326214	2.420325	0.876288	3.043916	0.287882	0.773437	NA
hsa-miR-6884-5p/hsa-mir-6884	1.364073	0.307822	2.420325	2.68904	3.810815	0.705634	0.480416	NA
hsa-miR-6885-3p/hsa-mir-6885	0.461733	0.923467	0	− 2.09228	4.877731	− 0.42895	0.667962	NA
hsa-miR-6885-5p/hsa-mir-6885	0	0	0	NA	NA	NA	NA	NA
hsa-miR-6886-3p/hsa-mir-6886	0.518941	0.509196	0.528686	0.681945	4.885862	0.139575	0.888996	NA
hsa-miR-6886-5p/hsa-mir-6886	3.215603	1.326214	5.104992	1.975359	2.513341	0.78595	0.431897	NA
hsa-miR-6887-3p/hsa-mir-6887	0	0	0	NA	NA	NA	NA	NA
hsa-miR-6887-5p/hsa-mir-6887	0.132171	0	0.264343	1.823505	4.944331	0.368807	0.712271	NA
hsa-miR-6888-3p/hsa-mir-6888	0	0	0	NA	NA	NA	NA	NA
hsa-miR-6888-5p/hsa-mir-6888	0.264343	0	0.528686	2.396631	4.924788	0.486646	0.626509	NA
hsa-miR-6889-3p/hsa-mir-6889	0	0	0	NA	NA	NA	NA	NA
hsa-miR-6889-5p/hsa-mir-6889	0	0	0	NA	NA	NA	NA	NA
hsa-miR-6890-3p/hsa-mir-6890	0	0	0	NA	NA	NA	NA	NA
hsa-miR-6890-5p/hsa-mir-6890	0.615645	1.231289	0	− 2.50085	4.855042	− 0.5151	0.606481	NA
hsa-miR-6891-3p/hsa-mir-6891	0	0	0	NA	NA	NA	NA	NA
hsa-miR-6891-5p/hsa-mir-6891	1.332589	0.509196	2.155982	2.185436	3.862236	0.565847	0.571498	NA
hsa-miR-6892-3p/hsa-mir-6892	0	0	0	NA	NA	NA	NA	NA
hsa-miR-6892-5p/hsa-mir-6892	1.103101	1.941859	0.264343	− 1.91165	4.019321	− 0.47562	0.634348	NA
hsa-miR-6893-3p/hsa-mir-6893	0	0	0	NA	NA	NA	NA	NA
hsa-miR-6893-5p/hsa-mir-6893	0	0	0	NA	NA	NA	NA	NA
hsa-miR-6894-3p/hsa-mir-6894	0.509196	1.018392	0	− 2.1782	4.872426	− 0.44705	0.654841	NA
hsa-miR-6894-5p/hsa-mir-6894	0.593905	0.923467	0.264343	− 0.86911	4.823254	− 0.18019	0.857002	NA
hsa-miR-6895-3p/hsa-mir-6895	0.132171	0	0.264343	1.823505	4.944331	0.368807	0.712271	NA
hsa-miR-6895-5p/hsa-mir-6895	0.132171	0	0.264343	1.823505	4.944331	0.368807	0.712271	NA
hsa-miR-7106-3p/hsa-mir-7106	0	0	0	NA	NA	NA	NA	NA
hsa-miR-7106-5p/hsa-mir-7106	0	0	0	NA	NA	NA	NA	NA
hsa-miR-7107-3p/hsa-mir-7107	0	0	0	NA	NA	NA	NA	NA
hsa-miR-7107-5p/hsa-mir-7107	0	0	0	NA	NA	NA	NA	NA
hsa-miR-7108-3p/hsa-mir-7108	0	0	0	NA	NA	NA	NA	NA
hsa-miR-7108-5p/hsa-mir-7108	0	0	0	NA	NA	NA	NA	NA
hsa-miR-7109-3p/hsa-mir-7109	2.176601	0	4.353203	4.838878	3.607545	1.341322	0.179816	NA
hsa-miR-7109-5p/hsa-mir-7109	0	0	0	NA	NA	NA	NA	NA
hsa-miR-7110-3p/hsa-mir-7110	0.264343	0	0.528686	2.396631	4.924788	0.486646	0.626509	NA
hsa-miR-7110-5p/hsa-mir-7110	0	0	0	NA	NA	NA	NA	NA
hsa-miR-7111-3p/hsa-mir-7111	0.264343	0	0.528686	2.396631	4.924788	0.486646	0.626509	NA
hsa-miR-7111-5p/hsa-mir-7111	0.132171	0	0.264343	1.823505	4.944331	0.368807	0.712271	NA
hsa-miR-7112-3p/hsa-mir-7112	3.467951	0.509196	6.426706	3.875739	2.813342	1.377628	0.168318	NA
hsa-miR-7112-5p/hsa-mir-7112	1.698738	1.018392	2.379085	1.354762	3.802344	0.356296	0.721619	NA
hsa-miR-7113-3p/hsa-mir-7113	0	0	0	NA	NA	NA	NA	NA
hsa-miR-7113-5p/hsa-mir-7113	2.080405	1.740485	2.420325	0.438371	2.902347	0.15104	0.879944	NA
hsa-miR-7114-3p/hsa-mir-7114	0	0	0	NA	NA	NA	NA	NA
hsa-miR-7114-5p/hsa-mir-7114	1.254762	0.923467	1.586057	0.848498	4.229139	0.200631	0.840987	NA
hsa-miR-711/hsa-mir-711	0	0	0	NA	NA	NA	NA	NA
hsa-miR-7150/hsa-mir-7150	0	0	0	NA	NA	NA	NA	NA
hsa-miR-7151-3p/hsa-mir-7151	0	0	0	NA	NA	NA	NA	NA
hsa-miR-7151-5p/hsa-mir-7151	0	0	0	NA	NA	NA	NA	NA
hsa-miR-7152-3p/hsa-mir-7152	0	0	0	NA	NA	NA	NA	NA
hsa-miR-7152-5p/hsa-mir-7152	0	0	0	NA	NA	NA	NA	NA
hsa-miR-7153-3p/hsa-mir-7153	0	0	0	NA	NA	NA	NA	NA
hsa-miR-7153-5p/hsa-mir-7153	0	0	0	NA	NA	NA	NA	NA
hsa-miR-7154-3p/hsa-mir-7154	0	0	0	NA	NA	NA	NA	NA
hsa-miR-7154-5p/hsa-mir-7154	0	0	0	NA	NA	NA	NA	NA
hsa-miR-7155-3p/hsa-mir-7155	0	0	0	NA	NA	NA	NA	NA
hsa-miR-7155-5p/hsa-mir-7155	0	0	0	NA	NA	NA	NA	NA
hsa-miR-7156-3p/hsa-mir-7156	2.725907	0	5.451813	5.173154	3.717745	1.391476	0.164081	NA
hsa-miR-7157-3p/hsa-mir-7157	0	0	0	NA	NA	NA	NA	NA
hsa-miR-7157-5p/hsa-mir-7157	0	0	0	NA	NA	NA	NA	NA
hsa-miR-7158-3p/hsa-mir-7158	0	0	0	NA	NA	NA	NA	NA
hsa-miR-7158-5p/hsa-mir-7158	0	0	0	NA	NA	NA	NA	NA
hsa-miR-7159-3p/hsa-mir-7159	0	0	0	NA	NA	NA	NA	NA
hsa-miR-7159-5p/hsa-mir-7159	0	0	0	NA	NA	NA	NA	NA
hsa-miR-7160-3p/hsa-mir-7160	0	0	0	NA	NA	NA	NA	NA
hsa-miR-7160-5p/hsa-mir-7160	0	0	0	NA	NA	NA	NA	NA
hsa-miR-7161-3p/hsa-mir-7161	0	0	0	NA	NA	NA	NA	NA
hsa-miR-7161-5p/hsa-mir-7161	0	0	0	NA	NA	NA	NA	NA
hsa-miR-7162-3p/hsa-mir-7162	0	0	0	NA	NA	NA	NA	NA
hsa-miR-7162-5p/hsa-mir-7162	0	0	0	NA	NA	NA	NA	NA
hsa-miR-718/hsa-mir-718	0.461733	0.923467	0	− 2.09228	4.877731	− 0.42895	0.667962	NA
hsa-miR-7-2-3p/hsa-mir-7-2	0.153911	0.307822	0	− 0.74599	4.97963	− 0.14981	0.880917	NA
hsa-miR-7515/hsa-mir-7515	0	0	0	NA	NA	NA	NA	NA
hsa-miR-758-3p/hsa-mir-758	1.321714	0	2.643428	4.240271	4.639562	0.913938	0.36075	NA
hsa-miR-758-5p/hsa-mir-758	0.132171	0	0.264343	1.823505	4.944331	0.368807	0.712271	NA
hsa-miR-759/hsa-mir-759	0	0	0	NA	NA	NA	NA	NA
hsa-miR-761/hsa-mir-761	0	0	0	NA	NA	NA	NA	NA
hsa-miR-762/hsa-mir-762	0	0	0	NA	NA	NA	NA	NA
hsa-miR-764/hsa-mir-764	0	0	0	NA	NA	NA	NA	NA
hsa-miR-7702/hsa-mir-7702	0	0	0	NA	NA	NA	NA	NA
hsa-miR-7703/hsa-mir-7703	0.396514	0	0.793028	2.767752	4.915475	0.563069	0.573388	NA
hsa-miR-7704/hsa-mir-7704	0.681477	0	1.362953	3.012055	4.910469	0.613395	0.539615	NA
hsa-miR-770-5p/hsa-mir-770	0	0	0	NA	NA	NA	NA	NA
hsa-miR-7843-3p/hsa-mir-7843	0.153911	0.307822	0	− 0.74599	4.97963	− 0.14981	0.880917	NA
hsa-miR-7843-5p/hsa-mir-7843	0	0	0	NA	NA	NA	NA	NA
hsa-miR-7844-5p/hsa-mir-7844	0.254598	0.509196	0	− 1.11434	4.959133	− 0.2247	0.82221	NA
hsa-miR-7845-5p/hsa-mir-7845	0.408509	0.817018	0	− 1.87945	4.892274	− 0.38417	0.700854	NA
hsa-miR-7846-3p/hsa-mir-7846	0.254598	0.509196	0	− 1.11434	4.959133	− 0.2247	0.82221	NA
hsa-miR-7847-3p/hsa-mir-7847	0	0	0	NA	NA	NA	NA	NA
hsa-miR-7848-3p/hsa-mir-7848	0	0	0	NA	NA	NA	NA	NA
hsa-miR-7849-3p/hsa-mir-7849	0	0	0	NA	NA	NA	NA	NA
hsa-miR-7850-5p/hsa-mir-7850	0.254598	0.509196	0	− 1.11434	4.959133	− 0.2247	0.82221	NA
hsa-miR-7851-3p/hsa-mir-7851	3.250458	2.758877	3.742039	0.481731	2.430371	0.198213	0.842878	NA
hsa-miR-7852-3p/hsa-mir-7852	0	0	0	NA	NA	NA	NA	NA
hsa-miR-7853-5p/hsa-mir-7853	0	0	0	NA	NA	NA	NA	NA
hsa-miR-7854-3p/hsa-mir-7854	3.379259	1.12484	5.633678	2.319979	2.466022	0.940778	0.346819	NA
hsa-miR-7855-5p/hsa-mir-7855	0	0	0	NA	NA	NA	NA	NA
hsa-miR-7856-5p/hsa-mir-7856	0	0	0	NA	NA	NA	NA	NA
hsa-miR-7973/hsa-mir-7973-1	0.254598	0.509196	0	− 1.11434	4.959133	− 0.2247	0.82221	NA
hsa-miR-7973/hsa-mir-7973-2	0.254598	0.509196	0	− 1.11434	4.959133	− 0.2247	0.82221	NA
hsa-miR-7975/hsa-mir-7975	0	0	0	NA	NA	NA	NA	NA
hsa-miR-7978/hsa-mir-7978	0	0	0	NA	NA	NA	NA	NA
hsa-miR-802/hsa-mir-802	0	0	0	NA	NA	NA	NA	NA
hsa-miR-8052/hsa-mir-8052	0	0	0	NA	NA	NA	NA	NA
hsa-miR-8053/hsa-mir-8053	0	0	0	NA	NA	NA	NA	NA
hsa-miR-8054/hsa-mir-8054	0	0	0	NA	NA	NA	NA	NA
hsa-miR-8055/hsa-mir-8055	0	0	0	NA	NA	NA	NA	NA
hsa-miR-8056/hsa-mir-8056	0	0	0	NA	NA	NA	NA	NA
hsa-miR-8057/hsa-mir-8057	0	0	0	NA	NA	NA	NA	NA
hsa-miR-8058/hsa-mir-8058	0	0	0	NA	NA	NA	NA	NA
hsa-miR-8059/hsa-mir-8059	0	0	0	NA	NA	NA	NA	NA
hsa-miR-8060/hsa-mir-8060	0	0	0	NA	NA	NA	NA	NA
hsa-miR-8061/hsa-mir-8061	0	0	0	NA	NA	NA	NA	NA
hsa-miR-8062/hsa-mir-8062	0	0	0	NA	NA	NA	NA	NA
hsa-miR-8063/hsa-mir-8063	0	0	0	NA	NA	NA	NA	NA
hsa-miR-8064/hsa-mir-8064	0	0	0	NA	NA	NA	NA	NA
hsa-miR-8066/hsa-mir-8066	0	0	0	NA	NA	NA	NA	NA
hsa-miR-8067/hsa-mir-8067	0	0	0	NA	NA	NA	NA	NA
hsa-miR-8068/hsa-mir-8068	0	0	0	NA	NA	NA	NA	NA
hsa-miR-8069/hsa-mir-8069-1	0	0	0	NA	NA	NA	NA	NA
hsa-miR-8069/hsa-mir-8069-2	0	0	0	NA	NA	NA	NA	NA
hsa-miR-8070/hsa-mir-8070	0	0	0	NA	NA	NA	NA	NA
hsa-miR-8071/hsa-mir-8071-1	0	0	0	NA	NA	NA	NA	NA
hsa-miR-8071/hsa-mir-8071-2	0	0	0	NA	NA	NA	NA	NA
hsa-miR-8072/hsa-mir-8072	0.264343	0	0.528686	2.396631	4.924788	0.486646	0.626509	NA
hsa-miR-8073/hsa-mir-8073	0	0	0	NA	NA	NA	NA	NA
hsa-miR-8074/hsa-mir-8074	0	0	0	NA	NA	NA	NA	NA
hsa-miR-8075/hsa-mir-8075	0	0	0	NA	NA	NA	NA	NA
hsa-miR-8076/hsa-mir-8076	0	0	0	NA	NA	NA	NA	NA
hsa-miR-8077/hsa-mir-8077	0	0	0	NA	NA	NA	NA	NA
hsa-miR-8078/hsa-mir-8078	0	0	0	NA	NA	NA	NA	NA
hsa-miR-8079/hsa-mir-8079	0	0	0	NA	NA	NA	NA	NA
hsa-miR-8080/hsa-mir-8080	0	0	0	NA	NA	NA	NA	NA
hsa-miR-8081/hsa-mir-8081	0	0	0	NA	NA	NA	NA	NA
hsa-miR-8082/hsa-mir-8082	0	0	0	NA	NA	NA	NA	NA
hsa-miR-8083/hsa-mir-8083	0	0	0	NA	NA	NA	NA	NA
hsa-miR-8084/hsa-mir-8084	0	0	0	NA	NA	NA	NA	NA
hsa-miR-8085/hsa-mir-8085	0	0	0	NA	NA	NA	NA	NA
hsa-miR-8086/hsa-mir-8086	0	0	0	NA	NA	NA	NA	NA
hsa-miR-8087/hsa-mir-8087	0	0	0	NA	NA	NA	NA	NA
hsa-miR-8088/hsa-mir-8088	0	0	0	NA	NA	NA	NA	NA
hsa-miR-8089/hsa-mir-8089	0	0	0	NA	NA	NA	NA	NA
hsa-miR-8485/hsa-mir-8485	1.311969	0.509196	2.114743	2.251359	4.243325	0.530565	0.59572	NA
hsa-miR-874-5p/hsa-mir-874	3.924947	7.056865	0.793028	− 2.7396	2.55159	− 1.07368	0.282965	NA
hsa-miR-875-3p/hsa-mir-875	0	0	0	NA	NA	NA	NA	NA
hsa-miR-875-5p/hsa-mir-875	0	0	0	NA	NA	NA	NA	NA
hsa-miR-876-3p/hsa-mir-876	1.913886	2.770401	1.057371	− 1.18542	3.644382	− 0.32527	0.744973	NA
hsa-miR-876-5p/hsa-mir-876	3.914731	6.772091	1.057371	− 2.42594	2.94169	− 0.82468	0.409556	NA
hsa-miR-885-3p/hsa-mir-885	1.172303	2.344605	0	− 3.39745	4.400056	− 0.77214	0.440032	NA
hsa-miR-885-5p/hsa-mir-885	2.143232	4.286464	0	− 4.27295	3.489871	− 1.22439	0.220807	NA
hsa-miR-887-5p/hsa-mir-887	2.000845	4.00169	0	− 4.19002	4.028931	− 1.03998	0.298348	NA
hsa-miR-888-3p/hsa-mir-888	0	0	0	NA	NA	NA	NA	NA
hsa-miR-888-5p/hsa-mir-888	0.408509	0.817018	0	− 1.87945	4.892274	− 0.38417	0.700854	NA
hsa-miR-889-3p/hsa-mir-889	5.96772	2.154756	9.780684	2.193308	2.542566	0.862636	0.388338	NA
hsa-miR-889-5p/hsa-mir-889	0	0	0	NA	NA	NA	NA	NA
hsa-miR-890/hsa-mir-890	0	0	0	NA	NA	NA	NA	NA
hsa-miR-891a-3p/hsa-mir-891a	0	0	0	NA	NA	NA	NA	NA
hsa-miR-891a-5p/hsa-mir-891a	0.153911	0.307822	0	− 0.74599	4.97963	− 0.14981	0.880917	NA
hsa-miR-891b/hsa-mir-891b	0.307822	0.615645	0	− 1.51932	4.922146	− 0.30867	0.757573	NA
hsa-miR-892a/hsa-mir-892a	0.54068	0.817018	0.264343	− 0.65628	4.837961	− 0.13565	0.892097	NA
hsa-miR-892b/hsa-mir-892b	0.418254	0.307822	0.528686	1.050318	4.906666	0.214059	0.830501	NA
hsa-miR-892c-3p/hsa-mir-892c	0	0	0	NA	NA	NA	NA	NA
hsa-miR-892c-5p/hsa-mir-892c	0	0	0	NA	NA	NA	NA	NA
hsa-miR-920/hsa-mir-920	0	0	0	NA	NA	NA	NA	NA
hsa-miR-921/hsa-mir-921	0	0	0	NA	NA	NA	NA	NA
hsa-miR-922/hsa-mir-922	0	0	0	NA	NA	NA	NA	NA
hsa-miR-924/hsa-mir-924	0	0	0	NA	NA	NA	NA	NA
hsa-miR-92a-2-5p/hsa-mir-92a-2	0.132171	0	0.264343	1.823505	4.944331	0.368807	0.712271	NA
hsa-miR-933/hsa-mir-933	2.847569	4.902109	0.793028	− 2.22911	2.827871	− 0.78826	0.430542	NA
hsa-miR-936/hsa-mir-936	1.739968	0.307822	3.172114	3.165097	3.833738	0.82559	0.409037	NA
hsa-miR-937-5p/hsa-mir-937	2.674923	0.509196	4.840649	3.424821	3.036521	1.127877	0.259372	NA
hsa-miR-938/hsa-mir-938	0.793028	0	1.586057	3.52828	4.884751	0.722305	0.470107	NA
hsa-miR-939-3p/hsa-mir-939	0.937195	0.817018	1.057371	0.579744	4.323721	0.134085	0.893336	NA
hsa-miR-939-5p/hsa-mir-939	2.059785	1.740485	2.379085	0.556172	3.165439	0.175701	0.860529	NA
hsa-miR-940/hsa-mir-940	2.725907	0	5.451813	5.173154	3.717745	1.391476	0.164081	NA
hsa-miR-942-3p/hsa-mir-942	5.176951	4.191539	6.162363	0.569277	2.044583	0.278432	0.780681	NA
hsa-miR-943/hsa-mir-943	4.552069	5.932024	3.172114	− 0.75645	2.363526	− 0.32005	0.748929	NA
hsa-miR-9500/hsa-mir-9500	0	0	0	NA	NA	NA	NA	NA
hsa-miR-9718/hsa-mir-9718	0	0	0	NA	NA	NA	NA	NA
hsa-miR-9851-3p/hsa-mir-9851	0	0	0	NA	NA	NA	NA	NA
hsa-miR-9851-5p/hsa-mir-9851	0	0	0	NA	NA	NA	NA	NA
hsa-miR-9898/hsa-mir-9898	1.749723	0.509196	2.99025	2.61811	3.581313	0.731048	0.46475	NA
hsa-miR-9899/hsa-mir-9899	1.628416	0.307822	2.94901	2.992443	3.577045	0.836568	0.402835	NA
hsa-miR-9900/hsa-mir-9900	0	0	0	NA	NA	NA	NA	NA
hsa-miR-9901/hsa-mir-9901	0	0	0	NA	NA	NA	NA	NA
hsa-miR-9902/hsa-mir-9902-1	4.82115	0.307822	9.334477	4.662047	2.592798	1.798075	0.072165	NA
hsa-miR-9902/hsa-mir-9902-2	4.82115	0.307822	9.334477	4.662047	2.592798	1.798075	0.072165	NA
hsa-miR-9983-3p/hsa-mir-9983	0.264343	0	0.528686	2.396631	4.924788	0.486646	0.626509	NA
hsa-miR-9986/hsa-mir-9986	2.41732	3.777268	1.057371	− 1.56755	2.987659	− 0.52467	0.599809	NA

### miR-214-5P downregulates RAD51 in BRCA wild-type/proficient TNBC cells

**Fig. 3:**
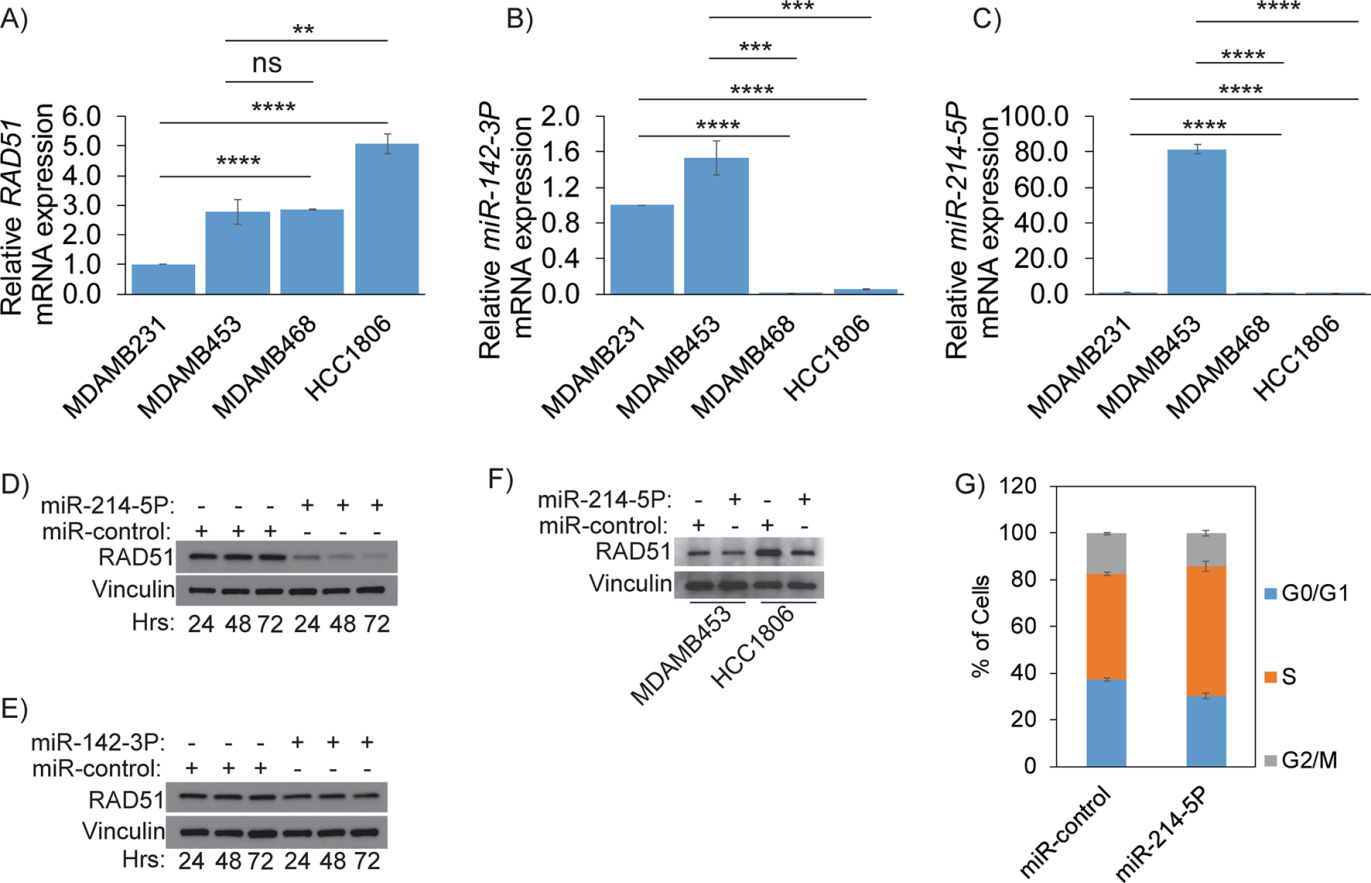
miR-214-5P regulates the expression of RAD51 in TNBC. **A** Comparison of *RAD51* expression in AA (MDAMB468/HCC1806) and EA (MDAMB231/MDAMB453) TNBC cell lines analyzed by RT-PCR in three independent experiments. **B** Comparison of miR-142-3P expression in AA (MDAMB468/HCC1806) and EA (MDAMB231/MDAMB453) TNBC cell lines analyzed by RT-PCR in three independent experiments. **C** Comparison of miR-214-5P expression in AA (MDAMB468/HCC1806) and EA (MDAMB231/MDAMB453) TNBC cell lines analyzed by RT-PCR in three independent experiments. **D** Western blot analysis of RAD51 in MDAMB468 cells transfected with miR-214-5P at time points indicated. **E** Western blot analysis of RAD51 in MDAMB468 cells transfected with miR-142-3P at time points indicated. **F** Western blot analysis of RAD51 in TNBC cells transfected with miR-214-5P. **G** Histogram representation of cell cycle profile in MDAMB468 cells 48 h after transfected with miR-214-5P in three independent experiments. (***p < 0.001) and (****p < 0.0001)

We first determined whether expression levels of miR-214-5P and miR-142-3P negatively correlate with RAD51 expression in racially distinctive TNBC cell lines by performing RT-PCR analysis. As shown in Fig. 3A, the *RAD51* levels were significantly higher in two pairs of AA in TNBC cell lines compared to their respective EA counterparts, except the comparison between MDAMB453 and MDAMB468. On the contrary, miR-142-3P (Fig. 3B) and miR-214-5P (Fig. 3C) were downregulated in AA TNBC cell lines compared to the EA TNBC cell lines. Collectively, these data indicate a negative correlation between specific miRNAs (miR-214-5P and miR-142-3P) and RAD51 levels in two pairs of AA and EA TNBC cell lines (Fig. 3A–C). To investigate whether miR-214-5P and miR-142-3P target RAD51 and downregulate its expression, MDAMB468 cells were transfected with miR-control, miR-214-5P, or miR-142-3P and analyzed for RAD51 protein levels by western blots at different time points after the transfection. As expected, miR-214-5P-transfected cells showed 70% to 90% downregulation of RAD51 in the time points evaluated (Fig. 3D; Additional file 2: S2A). However, miR-142-3P was less efficient in RAD51 downregulation than miR-214-5P (Fig. 3E; Additional file 2: S2B). This could be due to multiple factors, including the presence of binding sites for miR-214-5P in two different regions (1522–1528 and 1702–1708) of RAD51 3’UTR (shown by miRNA Target Base analysis), which might influence efficient targeting. Therefore, further studies were focused only on using miR-214-5P. To rule out that these results were not cell line-specific, we further examined multiple TNBC cell lines. Consistent with MDAMB468 cells’ data (Fig. 3D), miR-214-5P downregulated the expression of RAD51 in MDAMB453 and HCC1806 TNBC cell lines (Fig. 3F). RAD51 is a critical factor in BRCA and associated protein complex mediated repair of DNA double-strand breaks (DSBs) and maintains the integrity of chromosomal DNA in replicating cells [37–40]. Since non-replicating cells tend to have low expression of the HR proteins, it is essential to rule out the contribution of any cell cycle discrepancies in miR-214-5P-transfected cells to RAD51 downregulation. In fact, our flow cytometry results from miR-214-5P-transfected MDAMB468 cells showed increased accumulation of cells in the S-phase compared to the miR-control-transfected cells (Fig. 3G; Additional file 2: S2C and S2D), which rules out the contribution of any cell cycle discrepancies to RAD51 downregulation.

### miR-214-5P targets 3’UTR region of RAD51 and regulates its mRNA levels

**Fig. 4:**
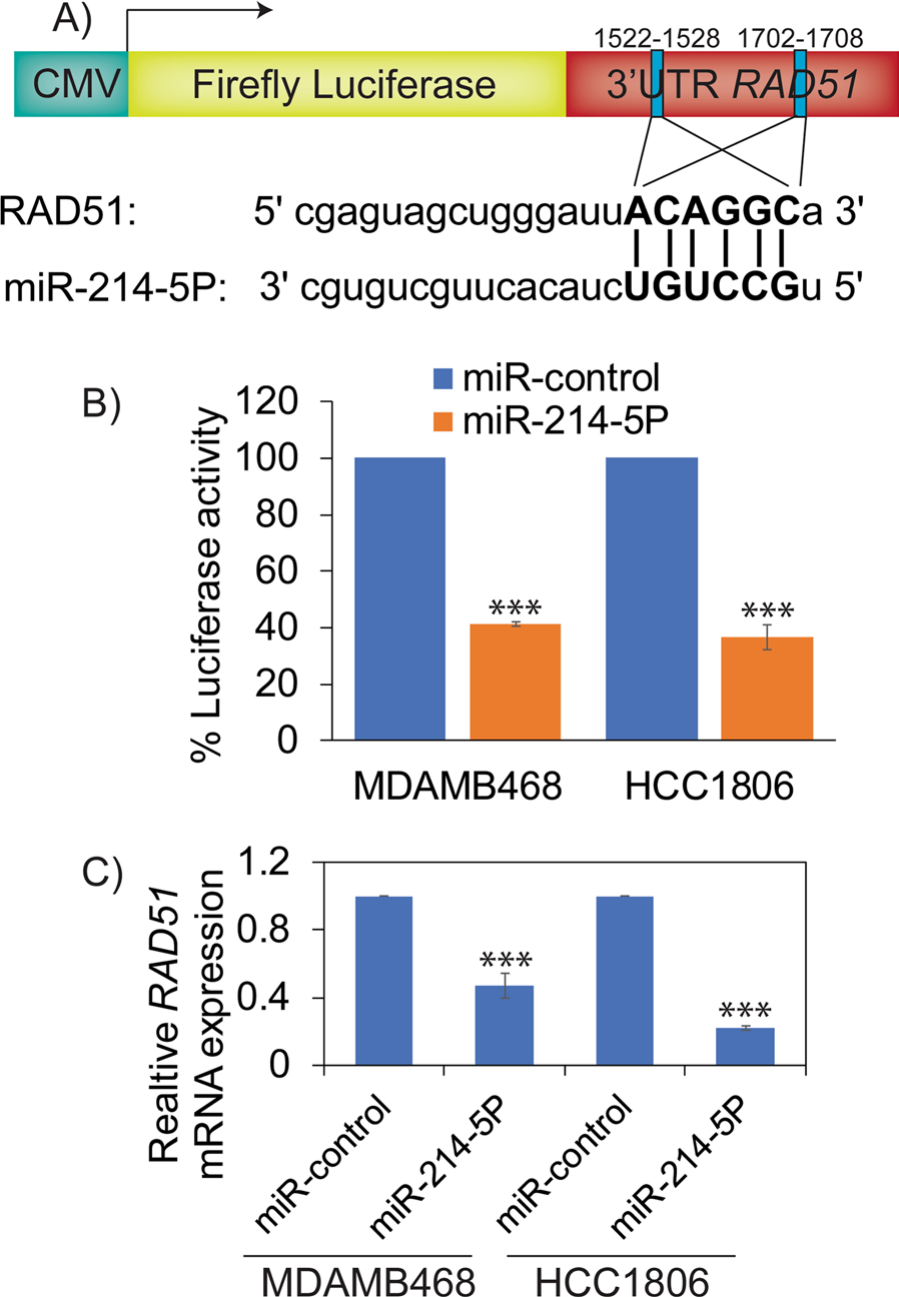
miR-214-5P binds to RAD51 3’UTR region and regulates RAD51 post-transcriptionally. **A** Schematic representation of luciferase reporter plasmid. **B** Histogram representation of luciferase reporter assay performed in MDAMB468 and HCC1806 cells. **C** RT-PCR analysis of *RAD51* expression in miR-214-5P-transfected TNBC cells. Fold-difference with standard deviation is represented as a histogram from three independent experiments. (***p < 0.001)

To confirm that miR-214-5P downregulates RAD51 by targeting the 3’UTR region of the transcript, we performed luciferase assays (Promega). A luciferase reporter was designed to include the luciferase ORF under a CMV promoter, followed by the *RAD51* 3’UTR region, which has the predicted seed sequences (1522–1528 and 1702–1708) for miR-214-5P (Fig. 4A). Co-transfection of the luciferase reporter with either miR-control or miR-214-5P showed > 50% decreased luciferase activity in miR-214-5P-transfected MDAMB468 and HCC1806 TNBC cells compared to the miR-control (Fig. 4B). Consistently, RT-PCR data showed downregulation of RAD51 in miR-214-5P-transfected cells compared to control miRNAs (Fig. 4C), which confirms that miR-214-5P targets the 3’UTR region of *RAD51* mRNA and regulates RAD51 posttranscriptionally. Additionally, this seed sequence is conserved in *RAD51* mRNA (1522–1528) in different primates (Additional file 3: Figure S3A), indicating a conserved mechanism of epigenetic regulation.

### miR-214-5P downregulates RAD51 and induces HR deficiency (HRD) in BRCA wild-type/proficient TNBC cells

**Fig. 5:**
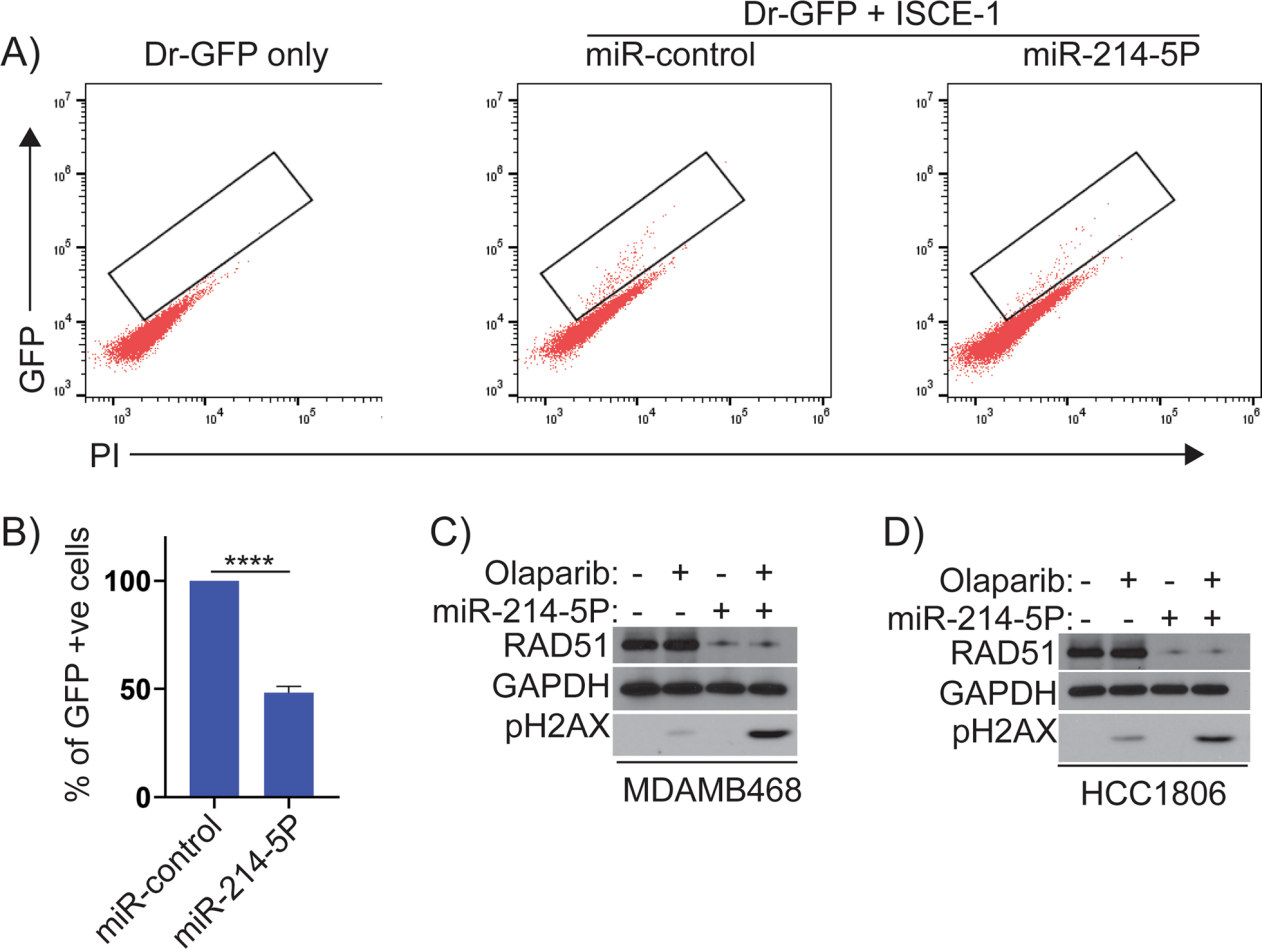
miR-214-5P mimic downregulates RAD51 and induces HRD. **A** MDAMB468 cells were transfected with Dr-GFP and selected using 5 µg/ml puromycin. Stably expressing cells were transfected with ISCE-1 and analyzed for GFP^+^ cells using flow cytometry 48 h after transfection. **B** Histogram representation of GFP^+^ cells from three independent experiments with standard deviation as error bars. **C** MDAMB468 and **D** HCC1806 cells were transfected with miR-control or miR-214-5P and analyzed for protein expression using western blot (****p < 0.0001)

RAD51 is an essential factor of a multi-protein complex of BRCA and related proteins that most accurately repair replication-associated DSBs [37–40]. Thus, deficiency or inhibition of RAD51 affects cells’ ability to repair DSBs due to HR deficiency [21, 40]. We performed Dr-GFP reporter assays to determine whether miR-214-5P-mediated downregulation of RAD51 affects TNBC cells’ efficiency to repair DSB by HR [41, 42]. We used BRCA-proficient MDAMB468 cells to transfect with the Dr-GFP plasmid and selected them in a puromycin selection medium. These Dr-GFP-expressing cells were then co-transfected with an ISCE-1 expression plasmid to induce DSBs and either miR-control or miR-214-5P and then analyzed for GFP expression using a flow cytometer. Consistent with RAD51 downregulation, miR-214-5P-treated MDAMB468 cells showed > 50% reduction in HR efficiency (*p* < 0.001) compared to the miR-control-transfected cells (Fig. 5A, B).

To further examine whether miR-214-5P-induced HRD in DNA repair-proficient TNBC cells causes increased DNA damage in response to PARPi olaparib, we transfected two RAD51 upregulated AA-derived TNBC cell lines (MDAMB468 and HCC1806) with miR-control or miR-214-5P and treated them with vehicle or olaparib. Consistent with the DNA repair deficiency, miR-214-5P-transfected MDAMB468 and HCC1806 cells showed downregulation of RAD51 and increased expression of pH2AX (*p* < 0.0001) protein levels in response to olaparib when compared to their respective miR-control-transfected cells treated with olaparib (Fig. 5C, D).

**Fig. 6:**
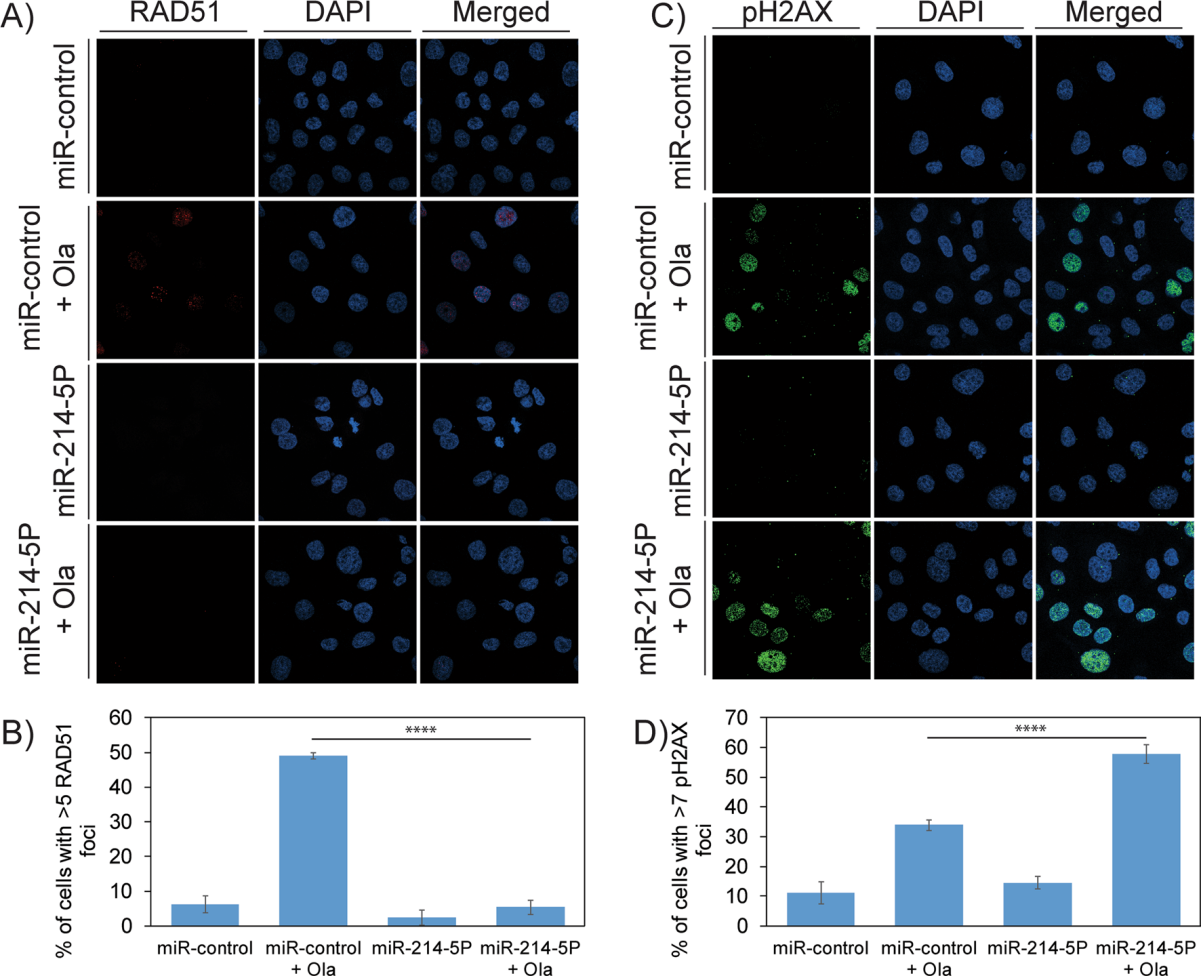
miR-214-5P mimic abrogates olaparib-induced RAD51 foci formation. **A** HCC1806 cells transfected with miR-control or miR-214-5P and treated with or without 25 µM olaparib for 24 h were analyzed for RAD51 foci using immunofluorescence. **B** More than 75 cells from three independent experiments were analyzed for the percentage of cells that shows > 5 RAD51 foci and represented as a histogram with standard error. **C** HCC1806 cells transfected with miR-control or miR-214-5P and treated with or without 25 µM olaparib for 24 h was analyzed for pH2AX foci using immunofluorescence. **D** More than 75 cells from three independent experiments were analyzed for percentage of cells that shows > 7 pH2AX foci and represented as a histogram with standard error. (****p < 0.0001)

To further confirm that miR-214-5P-mediated downregulation of RAD51 also affects its repair foci formation in olaparib-induced DNA damage, we examined for the levels of repair foci of RAD51 and pH2AX in HCC1806 cells (Fig. 6A, C). In miR-control-transfected cells, RAD51 and pH2AX foci were undetectable. Consistent with the western blot data (Fig. 5C, D), olaparib treatment increased the number of RAD51 and pH2AX foci compared to DMSO-treated cells. Furthermore, for the miR-214-5P and olaparib combination, miR-214-5P attenuated olaparib-induced RAD51 foci formation compared to the miR-control and olaparib combination (Fig. 6B). Contrarily, pH2AX foci levels were elevated upon olaparib treatment in control cells and were further elevated by the miR-214-5P and olaparib combination, indicating increased DNA damage in these cells (Fig. 6D). These data confirm that the combination of miR-214-5P and olaparib downregulates RAD51 and increases DNA damage as measured by pH2AX.

### miR-214-5P synergizes with PARPi olaparib in BRCA wild-type/proficient TNBC cells

**Fig. 7:**
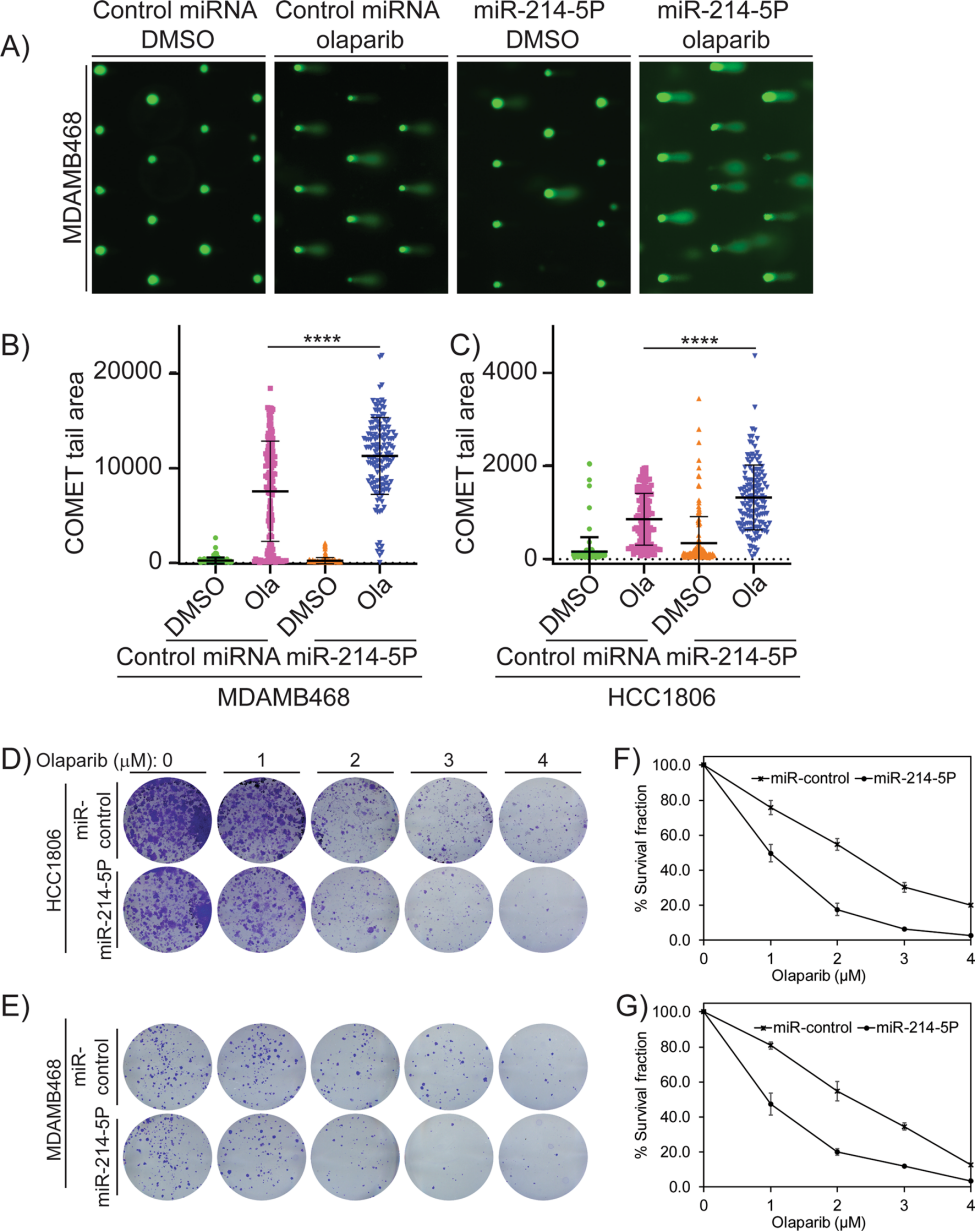
miR-214-5P mimic downregulates RAD51 and synergizes with olaparib. **A** Comet assay representative images of MDAMB468 cells transfected with miR-control or miR-214-5P and treated with or without 25 µM olaparib for 24 h. **B** MDAMB468 and **C** HCC1806 analysis of comet tail area in more than 25 cells from three different experiments with their standard deviation as the error bars. **D** High-density colony assay plates of HCC1806 cells transfected with miR-control or miR-214-5P and treated with different concentrations of olaparib. **E** Low-density colony assay plates of MDAMB468 cells transfected with miR-control or miR-214-5P and treated with varying concentrations of olaparib. **F** Survival fraction of MDAMB468 cells transfected with miR-control or miR-214-5P and treated with varying concentrations of olaparib in three independent experiments. **G** Survival fraction of HCC1806 cells transfected with miR-control or miR-214-5P and treated with varying concentrations of olaparib in three independent experiments

To validate that increased pH2AX is due to increased DNA lesions, we performed alkaline COMET assays in these cell lines (MDAMB468 and HCC1806) (Fig. 7A; Additional file 3: S3B). In agreement with the pH2AX levels, miR-214-5P-transfected MDAMB468 and HCC1806 cells showed significantly elevated levels of COMET tails (*p* < 0.0001) in response to olaparib when compared to their respective miR-control-transfected cells treated with vehicle or olaparib (Fig. 7B, C). Together, these results suggest that miR-214-5P downregulates the HR protein RAD51, causes HR repair deficiency, and potentiates olaparib-induced DNA damage in BRCA-proficient TNBC cells. Furthermore, these data also propose a novel synergistic lethality mechanism involving miR-214-5P and olaparib combination therapy, similar to *BRCA*-mutant or HR-deficient TNBCs.

To confirm that the miR-214-5P-mediated downregulation of RAD51 causes synergistic lethality with the PARPi olaparib, we first performed a high-density colony assay using HCC1806 cells transfected with miR-control or miR-214-5P, and treated them with various concentrations of olaparib. As shown in Fig. 7D, HCC1806 cells transfected with miR-control showed a dose-dependent decrease in the colony-forming ability of these cells. Consistent with the DNA repair deficiency, miR-214-5P-transfected cells showed decreased colony formation capacity relative to miR-control-treated cells in response to olaparib at all the tested drug concentrations. To further confirm, we performed low-density colony assays using MDAMB468 (Fig. 7E) and HCC1806 cells transfected with miR-control or miR-214-5P, treated with or without various concentrations of olaparib and plotted the survival fractions (Fig. 7F, G). The miR-214-5P-transfected cells showed significantly decreased colony formation compared to miR-control-treated cells in response to olaparib at all the tested drug concentrations. Together, these data suggest a novel synergistic lethality combination of miR-214-5P and olaparib to effectively treat *BRCA*-proficient TNBCs that could aid in decreasing disparities in the therapeutic outcomes of TNBCs.

## Discussion

Epidemiological and other population-based studies show the existence of racial disparities relating to BC progression and therapy outcomes in AA ethnic population [43]. In particular, the occurrence of TNBC, the most lethal form of BC, is 2.21 times higher for AA women compared to EA women, and it affects them at a younger age and has a worse prognosis [10, 11, 44, 45]. Several biological and non-biological factors have been identified to influence racial disparities [46]. Biologically, obesity and waist–hip ratio (WHR) have been identified as increased risk for AA TNBC patients. The prevalence of TNBC in overweight and obese AA women is twofold compared to that in normal-weight AA women [47], and these studies are further supported by many studies implicating WHR as a strong risk factor for TNBC in AA women [48]. However, specific endpoints like somatic mutations in prominent genes like *TP53* (46% of AA patients, compared to 27% in EA patients) have been documented as the racial disparity in the overall cancer incidence but not in TNBC [49]. These observations confirm the need to identify more potential mechanistic underpinnings that cause a racial disparity in TNBC.

MiRNAs have been identified as a potential regulator of racial disparity in multiple cancers [50–52], including TNBC [53, 54]. Our present study identifies that the loss of miR-214-5P, which epigenetically regulates RAD51, could be a marker for a poor prognosis in TNBC patients. It could contribute to our understanding of racial disparities in TNBCs. Particularly, miR-214-5P’s role has been documented in proliferation [55], migration, invasion [56], aggressive tumor growth [57] and resistance [58] in multiple cancers. Our studies identified a novel role for miR-214-5P in regulating critical DNA repair gene *RAD51* and suggested that loss of it in AA TNBCs upregulates RAD51 and affects their response to therapeutics. As miR-214-5P is significantly decreased in AA TNBCs compared to EA TNBCs, it indicates its contribution to racial disparities in TNBC therapeutic outcomes and could serve as a biomarker for these patients. Notably, the mechanism identified in our study paves a way to treat BRCA wild-type TNBC patients who do not benefit from single-agent PARPi-targeted therapies.

Upon treatment with PARPi, cells with HRD display extensive DNA lesions and synthetic lethality [59]. PARP inhibitors are a class of agents approved first for *BRCA*-deficient ovarian cancer and BC and later for other cancers [60]. The biochemical effects of deleterious germline mutations in *BRCA1* and *BRCA2* compromise their essential role in maintaining functional, high-fidelity DNA repair via homologous recombination, indicating their potential clinical utility [61, 62]. However, later studies established that not only *BRCA* genes but proteins with loss-of-function mutations involved in HR (including *RAD51*, *FANCD2*, and *PALB2*) also synergize with PARPi [63–65]. However, PARP inhibitors are currently effective only in 15–20% of TNBC patients with genetically compromised HR. Several researchers have identified alternative ways to induce HR non-genetically by targeting various proteins involved in HR, including RAD51 [66–68]. Accordingly, our previous study identified that CHK1 inhibitor prexasertib inhibits RAD51 and synergizes with olaparib [21]. Similarly, inhibition of Myc [69], glycosylase OGG1 [70], nicotinamide phosphoribosyltransferase [71], CDK [72] and topoisomerase 1 [73] have been shown to combine with PARPi in TNBCs synergistically. However, none of these combinations addresses the potential racial disparity observed in AA TNBC patients. Based on our results, we propose a novel synergistic lethality mechanism involving the combination of miR-214-5P and PARPi to effectively treat TNBC, especially in AA TNBC patients, as they express significantly low miR-214-5P and increased RAD51 compared to EA TNBCs. Additionally, a recent clinical study evaluating olaparib against metastatic BCs in patients with BRCA mutations shows promising results [25]. Since olaparib is already FDA-approved, targeting RAD51 either by miRNAs (e.g., miR-214-5P) or by drugs that promote its degradation would be an effective combination to treat HR-proficient TNBCs to overcome therapeutic resistance.

## Conclusions

Our studies provide preclinical evidence for targeting RAD51 by miR-214-5P to induce HR deficiency in *BRCA*-wild-type or HR-proficient TNBCs and shows synergistic lethality with an FDA-approved PARPi olaparib. Since significantly decreased expression of miR-214-5P and compensatory upregulation of RAD51 were observed in AA TNBCs compared to EA TNBCs, these observations could serve as biomarkers for racial disparity in TNBC. Importantly, these results also suggest that a novel combination of miR-214-5P and olaparib could be an effective therapy for *BRCA*-wild-type TNBCs and reduce racial disparities in TNBC outcomes.

## Supplementary Information

**Additional file 1.** Column heat map analysis of miRNA sequencing between EA (MDAMB231 and MDAMB453) and AA (MDAMB468 and HCC1806) cells.

**Additional file 2.** miR-214-5P regulates the expression of RAD51 in a cell cycle independent manner. (A) Densitometry analysis for RAD51 expression in MDAMB468 cells transfected with miR-214-5P in three independent experiments are represented in the histogram with standard deviation as error bars. (B) Densitometry analysis for RAD51 expression in MDAMB468 cells transfected with miR-142-3P in three independent experiments isrepresented in the histogram with standard deviation as error bars. Cell cycle profile of MDAMB468 cells treated with miR-control (C) or miR-214-5P (D).

**Additional file 3.** Bioinformatic analysis of protein sequence conservity. (A) Protein alignment analysis using UNIPORT shows a highly conserved seed sequence 1522- 1528 in RAD51 mRNA. (B) Comet assay representative images in HCC1806 cells transfected with miR-control or miR-214-5P and treated with or without 25M olaparib for 24 h.

## Data Availability

All data generated or analyzed during this study are included in this research article and its supplementary information files.

## Abbreviations

BC: Breast cancer
TNBC: Triple-negative breast cancer
AA: African-American
DNA: Deoxyribonucleic acid
EA: European-American
miRNA: Micro-ribonucleic acid
CA: Caucasian-American
TCGA: The Cancer Genome Atlas
UALCAN: The University of Alabama at Birmingham Cancer Data Analysis Portal
GFP: Green fluorescent protein
HR: Homologous recombination
ER: Estrogen receptor
PR: Progesterone receptor
HER2/EGFR2: Human epidermal growth factor receptor 2
BRCA: Breast cancer susceptibility protein
BRCA1/2: Breast cancer gene 1/2
ATM: Ataxia telangiectasia-mutated
ATR: Ataxia telangiectasia and rad3-related
DSB: DNA double-strand break
FDA: Food and Drug Administration
PARPi: Poly-ADP ribose polymerase (PARP) inhibitors
HRD: Homologous recombination deficiency
RT-PCR: Real-time polymerase chain reaction
DMSO: Dimethyl sulfoxide
ATP: Adenosine triphosphate
GAPDH: Glyceraldehyde-3-phosphate dehydrogenase
PBS: Phosphate-buffered saline
EDTA: Ethylenediamine tetra acetic acid
EGTA: Ethylene glycol-bis(β-aminoethyl ether)-*N*,*N*,*N*′,*N*′-tetra acetic acid
TBST: Tris-buffered saline in 0.1% tween 20
UTR: Untranslated region
CSC: Cancer stem cell
ncRNA: Non-coding RNA
mRNA: Messenger RNA
CHK1: Checkpoint kinase 1
