# Supplementary figures and images for: Racial differences in RAD51 expression are regulated by miRNA-214-5P and its inhibition synergizes with olaparib in triple-negative breast cancer
Source: Breast Cancer Res. 2023 Apr 20;25:44. doi: 10.1186/s13058-023-01615-6 (PMC10120249; doi:10.1186/s13058-023-01615-6)

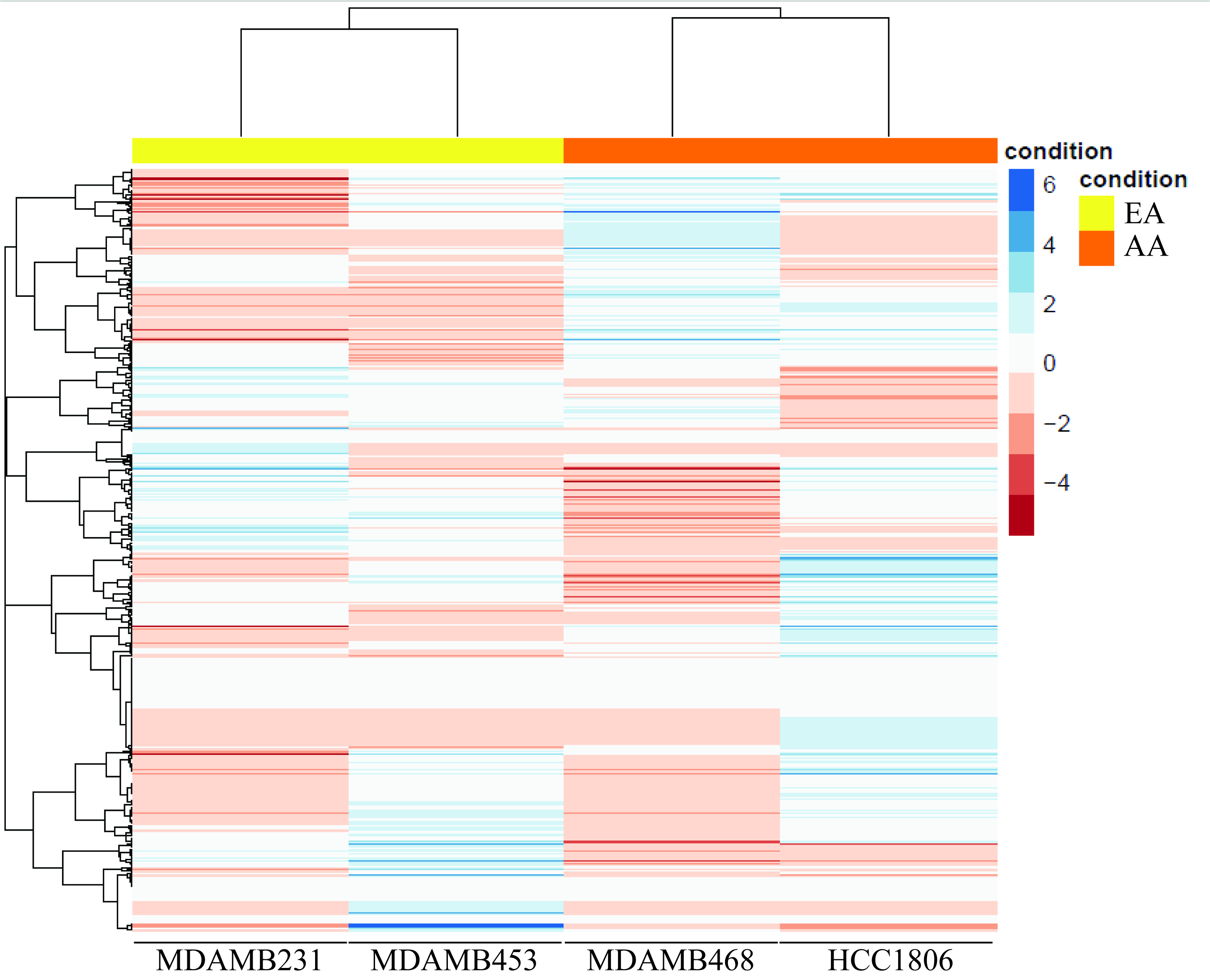

Supplement: Supplementary file 1 — Additional file 1. Column heat map analysis of miRNA sequencing between EA (MDAMB231 and MDAMB453) and AA (MDAMB468 and HCC1806) cells. [file 13058_2023_1615_MOESM1_ESM.tif]

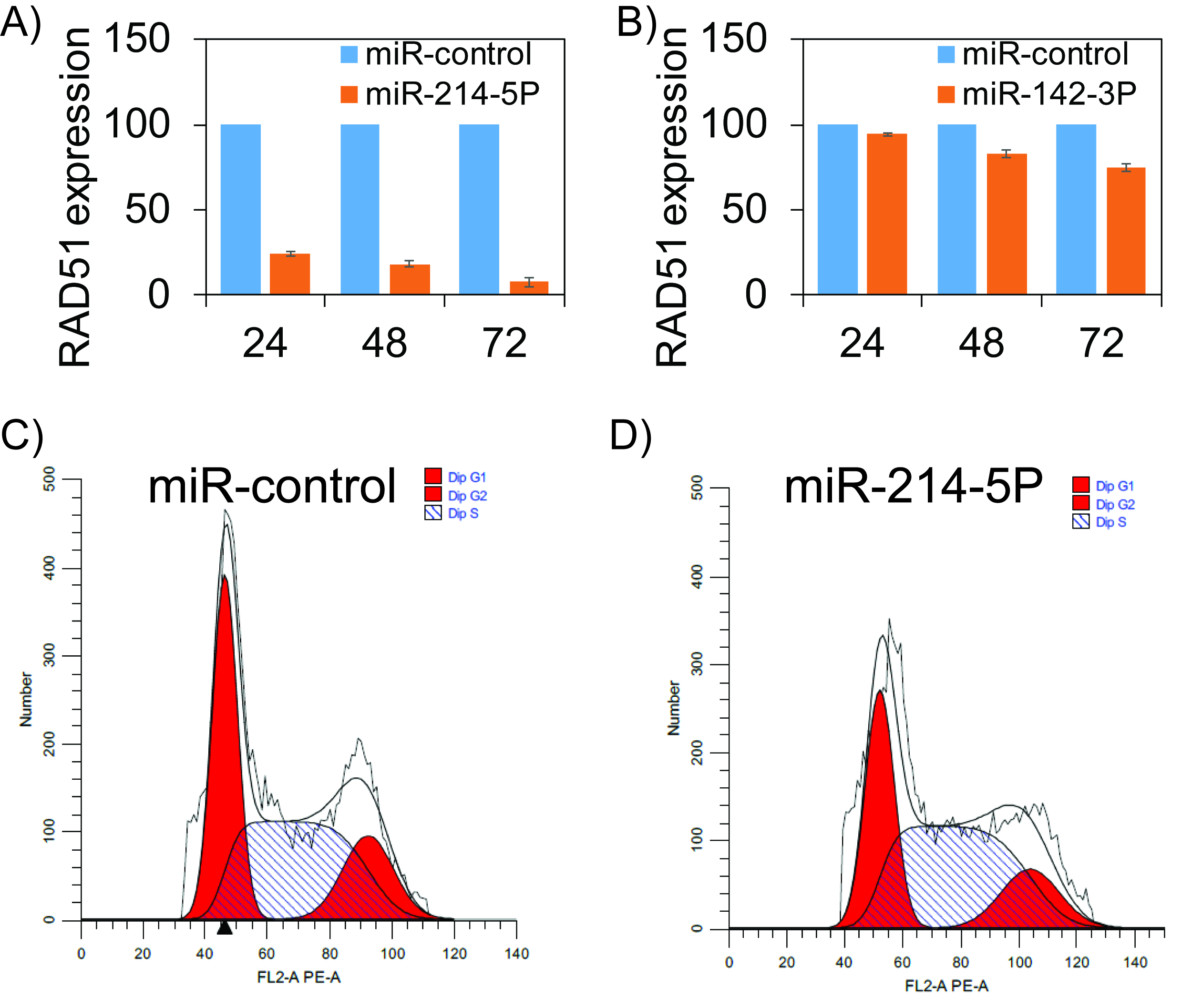

Supplement: Supplementary file 2 — Additional file 2. miR-214-5P regulates the expression of RAD51 in a cell cycle independent manner. (A) Densitometry analysis for RAD51 expression in MDAMB468 cells transfected with miR-214-5P in three independent experiments are represented in the histogram with standard deviation as error bars. (B) Densitometry analysis for RAD51 expression in MDAMB468 cells transfected with miR-142-3P in three independent experiments isrepresented in the histogram with standard deviation as error bars. Cell cycle profile of MDAMB468 cells treated with miR-control (C) or miR-214-5P (D). [file 13058_2023_1615_MOESM2_ESM.tif]

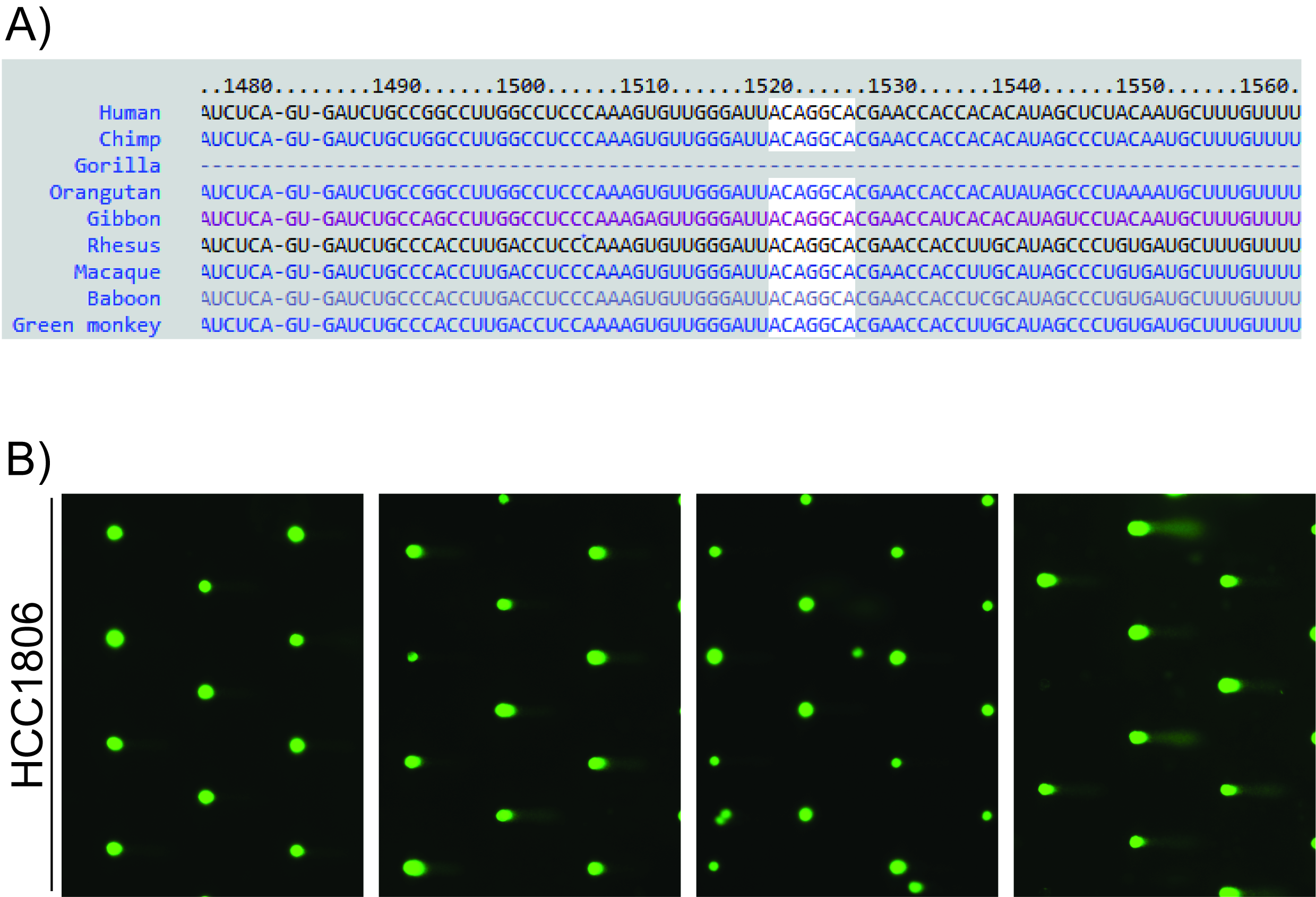

Supplement: Supplementary file 3 — Additional file 3. Bioinformatic analysis of protein sequence conservity. (A) Protein alignment analysis using UNIPORT shows a highly conserved seed sequence 1522- 1528 in RAD51 mRNA. (B) Comet assay representative images in HCC1806 cells transfected with miR-control or miR-214-5P and treated with or without 25M olaparib for 24 h. [file 13058_2023_1615_MOESM3_ESM.tif]
